# Discovery of a Baloxavir‐Inspired Endonuclease Inhibitor That Prevents Herpes Simplex Virus 1 Replication in Cell Culture and In Vivo

**DOI:** 10.1002/advs.202508006

**Published:** 2025-09-15

**Authors:** Sabina Andreu, Kai Tang, Jiahui Zhou, Gabriel Pino‐Peco, Nerea López‐Carrobles, Raquel Bello‐Morales, Daniel Galdo‐Torres, Lina Zhang, Federico Gago, José Antonio López‐Guerrero, Xinyong Liu, Peng Zhan, Luis Menéndez‐Arias

**Affiliations:** ^1^ Centro de Biología Molecular “Severo Ochoa ” Consejo Superior de Investigaciones Científicas and Universidad Autónoma de Madrid c/ Nicolás Cabrera 1, Campus de Cantoblanco‐UAM Madrid 28049 Spain; ^2^ Department of Molecular Biology Universidad Autónoma de Madrid c/ Darwin, 2 Cantoblanco Madrid 28049 Spain; ^3^ Department of Medicinal Chemistry Key Laboratory of Chemical Biology Ministry of Education School of Pharmaceutical Sciences Cheeloo College of Medicine Shandong University Jinan 250012 P. R. China; ^4^ Department of Biomedical Sciences Universidad de Alcalá Alcalá de Henares Madrid 28805 Spain

**Keywords:** antiviral drug, baloxavir, DNA packaging, herpesvirus, HSV, metalloenzymes, nuclease

## Abstract

Herpes simplex viruses (HSV‐1 and HSV‐2) are highly prevalent and contagious, causing lifelong infections that cannot be eradicated with current therapies. Acyclovir and other viral DNA polymerase inhibitors are effective antiviral agents for treating HSV infections. However, despite the recent approval of pritelivir and amenamevir (helicase–primase complex inhibitors), drug resistance is still a major threat to therapeutic success. This research focuses on developing new antiviral strategies against HSV‐1 by targeting the pUL15 endonuclease, a component of the viral packaging motor/terminase complex, using substituted polycyclic pyridones derived from baloxavir acid. Several compounds display low micromolar IC_50_ values in enzymatic assays. Among them, the prioritized compound, **LN‐7**, shows a 50% effective concentration (EC_50_) of 2.8 ± 1.1 µm in antiviral assays and favorable pharmacokinetic properties in rats. **LN‐7** demonstrates antiviral efficacy in infected mice, while exhibiting fewer clinical signs compared to controls. Overall, **LN‐7** emerges as a promising lead for treating herpesvirus infections and is therefore a first‐in‐class drug candidate targeting HSV genome packaging.

## Graphical abstract

Current herpesvirus treatments targeting the viral DNA polymerase are limited by toxicities and drug resistance. Novel compounds inhibiting the nuclease activity of the herpes simplex virus 1 (HSV‐1) terminase complex, required for viral genome packaging, have been identified. Among them, LN‐7 demonstrates efficacy against HSV‐1 in cell culture and animal models (mice), emerging as a “first‐in‐class” antiviral drug candidate.

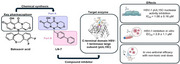

## 1 Introduction

Herpes is a common infection caused by herpes simplex virus (HSV) that can cause painful blisters or ulcers. There are two types of HSV. HSV‐1 is the main causative agent of oral herpes and spreads primarily by skin‐to‐skin contact. Both HSV‐1 and HSV‐2 can infect the genitals, although HSV‐2 is more commonly linked to genital herpes. In addition, they can cause keratitis, encephalitis, and severe neonatal disease. According to WHO's latest estimates obtained in 2016, 67% of the world population under the age of 50 have been infected with HSV‐1,^[^ ^1^ ^]^ while 187 million people between 15 and 49 years old had at least one episode of HSV‐related painful, recurrent genital ulcer disease.^[^ ^2^ ^]^

Acyclovir, valacyclovir (an esterified version of acyclovir), and famciclovir (a prodrug of penciclovir) are commonly prescribed to combat HSV‐1, although penciclovir, ganciclovir, cidofovir, and foscarnet are also used for the treatment of skin lesions caused by HSV‐1 and HSV‐2.^[^ ^3^ ^]^ All of these drugs are DNA polymerase inhibitors, acting as obligate chain terminators (e.g., acyclovir) or delayed chain terminators (e.g., penciclovir and cidofovir)^[^ ^4^ ^]^ or interfering with the polymerization reaction by mimicking the anion pyrophosphate, as in the case of foscarnet.^[^ ^5^, ^6^ ^]^ However, the limited efficacy of these antiviral medications is a major concern, since they only shorten the recovery period of infection by one or two days. In addition, the high prevalence of resistance to antiviral drugs, particularly acyclovir, has also been documented in immunocompromised individuals.^[^ ^3^, ^7^ ^]^

Novel antiherpetic compounds acting on different targets have been developed to overcome resistance while providing effective therapies. Among them, pritelivir and amenamevir are approved inhibitors of the helicase–primase complex that inhibit the replication of both HSV‐1 and HSV‐2, including acyclovir‐resistant viruses.^[^ ^8^ ^]^ These drugs act by blocking the unwinding, priming, and replication of the DNA template. In addition, *n*‐docosanol (also known as behenyl alcohol) is an approved antiviral agent that interacts with cell surface phospholipids, inhibiting the fusion of the HSV envelope with the plasma membrane.^[^ ^9^ ^]^ Additional efforts in the development of antiviral agents against herpesviruses culminated with the approval of drugs such as maribavir and letermovir, specific for the treatment of human cytomegalovirus (HCMV) infections. Maribavir is an inhibitor of the pUL97 serine/threonine protein kinase, an enzyme involved in viral encapsidation and nuclear egress of viral particles from infected cells.^[^ ^10^ ^]^ Letermovir targets the pUL56 subunit in the HCMV terminase complex pUL51/pUL56/pUL89.^[^ ^11^, ^12^ ^]^

The terminase complex is required to package the viral double‐stranded DNA (dsDNA) genome into a preformed protein capsid. It contains an endonuclease activity (located in HCMV pUL89) that cleaves the viral genome units generated in tandem during the DNA replication process.^[^ ^13^ ^]^ In HSV‐1, the terminase complex is composed of pUL15 (an ATPase/endonuclease) and two regulator/fixer proteins, pUL28 and pUL33.^[^ ^14^ ^]^ HSV‐1 *UL15* is the most conserved gene within the family *Herpesviridae*. It encodes a protein of 735 amino acids with five functional domains: a) N‐lasso (residues 1–152), Strut (153–252), ATPase (253–413), regulator (414–478), and nuclease (479–735)^[^ ^14^ ^]^

The nuclease domains of HSV‐1 pUL15^[^ ^15^ ^]^ and HCMV pUL89^[^ ^16^, ^17^ ^]^ show strong structural similarity with ribonucleases H (RNases H) and retroviral integrases.^[^ ^18^, ^19^, ^20^ ^]^ Moreover, their mechanism of action is also shared by a large class of two‐metal ion‐dependent enzymes, including viral nucleases such as the cap‐snatching endonucleases of influenza virus, bunyavirus, and arenavirus^[^ ^21^, ^22^ ^]^ and Holliday junction resolvases of poxviruses.^[^ ^23^ ^]^

α‐Hydroxytropolones are natural products with broad biological activity due to their inhibitory activity against dinuclear metalloenzymes.^[^ ^24^ ^]^ Among them, β‐thujaplicinol was found to inhibit HIV and hepatitis B virus RNases H, and human RNase H1 in enzymatic assays.^[^ ^25^, ^26^, ^27^ ^]^ In cell culture, β‐thujaplicinol and other α‐hydroxytropolones show potent antiviral activity against HSV‐1, HSV‐2, and acyclovir‐resistant mutants.^[^ ^28^, ^29^, ^30^, ^31^ ^]^ These data are consistent with the sub‐micromolar inhibitory activity of β‐thujaplicinol in endonuclease activity assays carried out with the C‐terminal domain of HSV‐1 pUL15 (pUL15C).^[^ ^32^ ^]^ In addition, raltegravir, dolutegravir, and other HIV‐1 integrase inhibitors are also effective against pUL15C^[^ ^32^ ^]^ and show anti‐HSV activity in antiviral assays,^[^ ^33^, ^34^ ^]^ although their potency is largely reduced in comparison with that against HIV‐1.^[^ ^30^ ^]^

Equivalent nucleases such as pUL89C in HCMV or ORF29 in human herpesvirus 8 (HHV8; also known as Kaposi's sarcoma‐associated herpesvirus) are also susceptible to approved HIV‐1 integrase inhibitors.^[^ ^16^, ^17^, ^33^, ^35^ ^]^ In recent years, numerous studies have explored pUL89C inhibition by using different metal‐chelating chemotypes and approaches previously followed in the discovery of HIV‐1 RNase H and integrase inhibitors (for a review, see ref. [36]).

**Figure 1:**
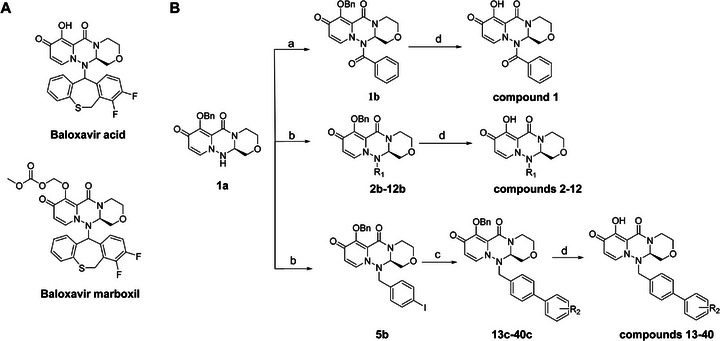
Chemical structures of baloxavir acid and baloxavir marboxil and synthesis of baloxavir‐inspired derivatives. A) Chemical structures of baloxavir acid and baloxavir marboxil. B) Synthesis of substituted polycyclic pyridone derivatives. Reagents and conditions: a) benzoyl chloride, DCM, TEA, 0 °C to r.t., 3 h, yield: 74%; b) iodomethane or iodoethane or different substituted benzyl bromide derivatives, Cs_2_CO_3_, *N*,*N*‐dimethylformamide (DMF), r.t.,12 h, yield: 76–90%; c) various substituted boric acid pinacol esters, Cs_2_CO_3_, Pd(dppf)Cl2, 1,4‐dioxane/H_2_O, 95 °C, 10 h, yield: 69–85%; d) LiCl, DMF, 90 °C, 12 h, yield: 34–56%.

Among them, hydroxypyridinones show an outstanding capacity to coordinate metal ions.^[^ ^37^ ^]^ A 2‐carboxylic acid‐modified 3‐hydroxy‐4‐pyridone pharmacophore is present in baloxavir marboxil^[^ ^38^, ^39^, ^40^ ^]^ (**Figure** **1**), an approved antiviral medication for the treatment of influenza. The active agent (baloxavir acid) acts as an enzyme inhibitor, targeting the influenza virus's cap‐dependent endonuclease activity.^[^ ^41^, ^42^ ^]^ In this study, we obtained a new series of (*R*)‐7‐hydroxy‐3,4,12,12a‐tetrahydro‐1*H*‐[1,4]oxazino[3,4‐*c*]pyrido[2,1‐*f*][1,2,4]triazine‐6,8‐diones, designed by introducing different phenyl and biphenyl substituents at position 2 of the triazinanone ring. Optimization via structure‐based drug design led to the discovery of a lead compound (**LN‐7**) that showed antiviral efficacy against HSV‐1.

## 2 Results

### 2.1 Chemical Synthesis

Metal ion chelating frameworks of RNase H inhibitors are mostly condensed heterocyclic systems with strong planarity, and the side chain dominant groups also contain large π systems, leading to strong rigidity and a large lattice energy of the whole molecule. Therefore, the physical and chemical properties of these inhibitors are poor. Reported HIV‐1 integrase and influenza endonuclease inhibitors have pharmacophore models similar to those of HIV‐1 RNase H inhibitors. Baloxavir acid is an influenza virus endonuclease inhibitor with aliphatic and spiro rings, linked through a triazanone moiety. In this work, we synthesized aryl and biaryl derivatives of baloxavir acid to explore the impact of modifications on the benzothiepine tricyclic system. The synthesis of these compounds is summarized in Figure 1B. (*R*)‐7‐(benzyloxy)‐3,4,12,12a‐tetrahydro‐1*H*‐[1,4]oxazino[3,4‐*c*]pyrido[2,1‐*f*][1,2,4]triazine‐6,8‐dione (**1a**) reacted with benzoyl chloride or different substituted benzyl bromides, respectively, which led to the formation of corresponding intermediates **1b** and **2b**–**12b** through nucleophilic substitution. The key intermediate **5b** was further modified to obtain the corresponding intermediates **13c**–**40c** using various substituted boric acid pinacol esters via the Suzuki reaction. Finally, the target compounds **1**–**40** were obtained by debenzylation of benzyl‐protected intermediates (**2b**–**12b** and **13c**–**40c**) with lithium chloride.

### 2.2 HSV‐1 pUL15C Nuclease Activity Inhibition and Structure–Activity Relationship Studies (SAR)

The polycyclic pyridine skeleton containing the chelating group of baloxavir acid has a key role in endonuclease inhibition. Therefore, we decided to retain the tricyclic moiety and focused our efforts on investigating the influence of the lower hydrophobic region on its activity. The nuclease inhibitory activity of the synthesized compounds was evaluated in enzymatic assays using DNA/DNA hybrids as substrates of the reaction. The substrate contained a 33‐nucleotide DNA labeled at its 5′ end with ^32^P, annealed to a 33‐nucleotide complementary DNA strand. The assays were performed with the C‐terminal domain of the terminase large subunit pUL15 (pUL15C) of HSV‐1.^[^ ^15^ ^]^ We first tested the effects of simplifying the structure of baloxavir acid by directly replacing the lower tricyclic ring with methyl (compound **2**) and ethyl groups (compound **3**). As shown in **Table** **1**, both compounds were more effective inhibitors than baloxavir acid. While the IC_50_ values of compounds **2** and **3** were 2.51 ± 0.22 and 2.20 ± 0.18 µm, respectively, baloxavir acid was found to be a weak inhibitor of HSV‐1 pUL15C with an IC_50_ of 11.5 ± 0.4 µm. These results supported the notion that the upper tricyclic ring is the key pharmacophore in the structure and most relevant to maintaining the inhibitory activity. Encouraged by these results, we synthesized aryl derivatives designated as compounds **1** and **4–10**. Compound **1**, a benzoylated derivative, exhibited ≈3.5‐fold higher potency than baloxavir acid (IC_50_ = 3.28 ± 0.22 µm), while the inhibitory activity of the benzylated derivative compound **4** was reduced (IC_50_ = 8.79 ± 0.61 µm). Notably, other substituted benzyl derivatives (compounds **5**–**10**) demonstrated slightly superior activity in comparison to **4**, with the 3‐F substituted compound 9 showing the highest inhibitory activity (IC_50_ = 2.07 ± 0.28 µm). Moreover, the replacement of the benzene ring in compound **4** with a 1‐naphthalene ring had a minor impact on its inhibitory activity (compound **11**, IC_50_ = 2.56 ± 0.53 µm), although the introduction of a larger pyrene substituent resulted in diminished activity (compound **12**, IC_50_ > 30 µm).

**Table 1:** Inhibitory effects of compounds against HSV‐1 pUL15C.

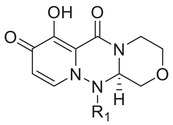		
Compound	R_1_	HSV‐1 pUL15C IC_50_ ^a)^ [µm]
Baloxavir acid	–	11.5 ± 0.4
β‐thujaplicinol	–	0.67 ± 0.10
**1**	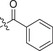	3.28 ± 0.22
**2**	Me	2.51 ± 0.22
**3**	Et	2.20 ± 0.18
**4**	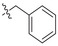	8.79 ± 0.61
**5**	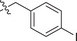	4.87 ± 0.49
**6**	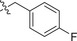	6.69 ± 0.53
**7**	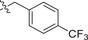	4.49 ± 0.55
**8**	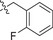	5.67 ± 0.53
**9**	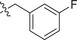	2.07 ± 0.28
**10**	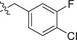	2.47 ± 0.17
**11**	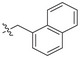	2.56 ± 0.53
**12**	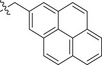	>30 (65.4 ± 6.9)
**13**	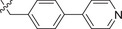	2.66 ± 0.34
**14**	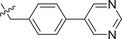	1.74 ± 0.10
**15**	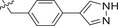	6.35 ± 0.59
**16**	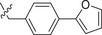	10.6 ± 1.4
**17 (LN‐7)**	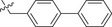	1.56 ± 0.16
**18**	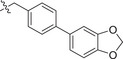	1.69 ± 0.29
**19**	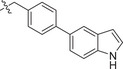	>30 (51.5 ± 6.2)
**20**	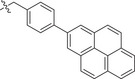	>30 (51.2 ± 7.4)

^a)^ Concentration required to inhibit by 50% the pUL15C nuclease activity in enzymatic assays. Numbers between parentheses indicate percentage inhibition at the highest concentration tested (50 µm). Reported values are averages of at least three independent determinations. Representative inhibition plots for compound **17** (**LN‐7**) and β‐thujaplicinol are shown in Figure S1 (Supporting Information).

Further studies revealed that increasing the hydrophobicity of compound 4 by introducing aromatic or heteroaromatic rings resulted in more potent inhibitors of the nuclease activity of pUL15C. Thus, compounds with pyridine (compound **13**), pyrimidine (compound **14**), and phenyl (compound **17**, also designated as **LN‐7**) substitutions showed IC_50_ values in the range of 1.56–2.66 µm, while those with pyrazole (compound **15**) and furan (compound **16**) substituents were much weaker inhibitors (Table 1). Similar results were observed with compounds having condensed aromatic substituents (i.e., compounds **18**–**20**). Among them, compound **18** showed acceptable inhibitory activity against HSV‐1 pUL15C, while compounds **19** and **20** were not active (IC_50_ > 30 µm), indicating that the pyrene group was not well‐tolerated.

As shown above, the biaryl derivative (compound **17**, **LN‐7**) demonstrated a strong inhibitory activity (IC_50_ = 1.56 ± 0.16 µm). Various substitutions were then introduced at different positions of the distal benzene ring to generate compounds **21**–**40** (**Table** **2**). Specifically, compounds with *ortho*‐hydroxy (**21**, IC_50_ = 6.47 ± 0.60 µm) and *para*‐hydroxy (**23**, IC_50_ = 6.05 ± 0.38 µm) substitutions were tolerated, but the compound with the *meta*‐hydroxy (**22**, IC_50_ = 12.9 ± 1.90 µm) substitution was less active. Similar results were observed with the cyano (compounds **24**–**26**) and amine (**27**–**29**) substituents. As a result, the *para* position of the benzene ring was selected as the optimal site for further exploration. When the *para* substitution contained a polar group (compounds **29**, **35**, **36**, and **37**), the inhibitory activity against pUL15C was much higher than for compounds having nonpolar groups at this position (compounds **30**–**34**). Interestingly, among the compounds having amide groups (i.e., compound **36**), the addition of *N*‐methyl (compound **37**) and *N*‐cyclopentyl (compound **39**) substituents led to significant increases in the IC_50_, while the *N*‐benzene derivative compound **40** was found to be almost inactive in the inhibition assays (IC_50_ > 30 µm).

**Table 2:** Inhibitory effects of compounds against HSV‐1 pUL15C.

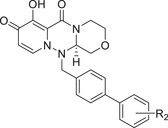		
Compound	R_2_	HSV‐1 pUL15C IC_50_ ^a)^ [µm]
**21**	2‐OH	6.47 ± 0.60
**22**	3‐OH	12.9 ± 1.90
**23**	4‐OH	6.05 ± 0.38
**24**	2‐CN	3.31 ± 0.45
**25**	3‐CN	15.1 ± 1.90
**26**	4‐CN	1.76 ± 0.32
**27**	2‐NH_2_	2.80 ± 0.61
**28**	3‐NH_2_	1.70 ± 0.24
**29**	4‐NH_2_	1.20 ± 0.28
**30**	4‐F	5.40 ± 0.65
**31**	4‐Cl	>30 (48.1 ± 10.4)
**32**	4‐NO_2_	>30 (74.3 ± 2.7)
**33**	4‐OCH_3_	19.4 ± 1.30
**34**	4‐OCF_3_	25.9 ± 8.50
**35**	4‐COOH	1.10 ± 0.10
**36**	4‐CONH_2_	1.11 ± 0.11
**37**	4‐CONHCH_3_	4.68 ± 0.37
**38**	4‐SO_2_CH_3_	5.15 ± 0.62
**39**	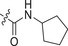	8.22 ± 0.73
**40**	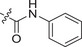	>30 (42.7 ± 13.4)

^a)^ Concentration required to inhibit by 50% the pUL15C nuclease activity in enzymatic assays. Numbers between parentheses indicate percentage inhibition at the highest concentration tested (50 µm). Reported values are averages of at least three independent determinations.

### 2.3 Antiviral Activity Evaluation

All compounds were evaluated in cell culture for their antiviral activity against HSV‐1 in a phenotypic assay using Vero cells and a recombinant HSV‐1 K26 strain containing the green fluorescent protein (*GFP*) gene fused in frame with the viral gene *UL35* encoding the viral capsid protein VP26.^[^ ^43^ ^]^ Five compounds (**7**, **10**, **17**, **30**, and **34**) showed significant inhibitory activity, although compound **17** (**LN‐7**) was the most potent of all. Its activity was slightly improved relative to β‐thujaplicinol, a known inhibitor of HIV‐1 RNase H and other structurally related viral nucleases, although its inhibitory activity was less than that of acyclovir (**Table** **3**). At a concentration of 20 µm, none of the compounds tested were cytotoxic. However, all compounds exhibited CC_50_ values above 100 µm, except for compounds **17**, **30**, and **34**, which showed a higher level of cytotoxicity. Nonetheless, the most potent derivatives had selectivity indices above 13.5.

**Table 3:** Inhibitory and cytotoxic effects, and selectivity indices (SI) of selected baloxavir‐inspired derivatives on HSV‐1 infection in Vero cells.

Compound	EC_50_ ^a)^ [µm]	CC_50_ [µm]	SI
**7**	3.4 ± 2.7	>100	>29.4
**10**	10.3 ± 0.9	>100	>9.7
**16**	>20 (48.5 ± 2.0)	>100	–
**17 (LN‐7)**	2.8 ± 1.1	38.8	13.8
**30**	4.0 ± 1.3	>66.5	>16.6
**34**	8.9 ± 1.0	>52.8	>5.9
Acyclovir (ACV)	0.68 ± 0.11	>100	>147.1
β‐thujaplicinol	3.9 ± 0.9	>100	>25.6
Baloxavir acid	>20 (40.7 ± 1.7)	>100	–
Baloxavir marboxil	>20 (13.7 ± 4.4)	>100	–

^a)^ Numbers in parentheses indicate percentage inhibition at the highest concentration tested (20 µm). Other inhibitors of the series showed less than 5% inhibition at 10 µm and were considered inactive (Figure S2, Supporting Information). Reported values are averages of at least three independent determinations.

Additional experiments revealed that the most effective compound (**LN‐7**) was also active against acyclovir (ACV)‐resistant HSV‐1 and HSV‐2 strains. The antiviral activities of **LN‐7** and ACV were tested against two different ACV‐resistant HSV‐1 strains in Vero cells. **LN‐7** showed a 50% effective concentration (EC_50_) of 3.2 ± 0.3 µm against the resistant strain F‐TKGF, comparable to its effect on wild‐type HSV‐1. By contrast, the antiviral efficacy of ACV against resistant strains F‐TKGF and Δ305 was substantially diminished, with EC_50_ values exceeding 20 µm in the case of F‐TKGF (Figure S3, Supporting Information). The antiviral efficacy of **LN‐7** was also tested against HSV‐2 strains G and Lovelace. In these experiments, we observed a reduction of two orders of magnitude in the viral titer in cultures treated with the compound at 10 µm concentration. Furthermore, the EC_50_ value obtained for compound **17** (**LN‐7**) in antiviral assays carried out with a recombinant HSV‐2 *GFP* strain in Vero cells was 3.6 ± 1.8 µm, similar to that obtained for HSV‐1 (Figure S4, Supporting Information).

### 2.4 Time‐of‐Addition Experiments

These experiments provide information on the target of antiviral compounds and were used to determine how long the addition of a compound can be postponed before it loses its antiviral activity. We determined the inhibitory effects of compounds **7**, **10**, **17**, **30**, and **34** in experiments where viruses were added or removed in the presence or absence of antiviral agents, according to different protocols (Figure S5, Supporting Information). The assays were carried out in Vero cells, using the modified HSV‐1 K26‐*GFP* virus indicated above with a multiplicity of infection (m.o.i.) of 0.1. When used, inhibitors were supplied at 10 µm. In several experiments, cells were treated with the inhibitor for 60 min before adding the virus (i.e., protocols A, B, and C). In all protocols, viral adsorption was allowed for 60 min in the presence or absence of the antiviral compound. After removing the virus by washing the cell cultures, the activity was measured after incubating the infected cells for 24 h, with (protocol A) or without the inhibitor (protocols B, C, and D). The strongest inhibitory effects were observed with protocols A and E that share the postentry incubation in the presence of the antiviral agent (Figure S5, Supporting Information). The behavior of compounds **7**, **10**, **17**, **30**, and **34** in these assays, which was shared by acyclovir and β‐thujaplicinol, is consistent with an inhibitory effect during viral DNA replication.

### 2.5 Synergistic Effect of Compound **17** (**LN‐7**) and Acyclovir

Combinations of antiviral drugs are more likely to be synergistic if they i) are of different classes, ii) have independent mechanisms of action, or iii) act upon different stages of the virus life cycle.^[^ ^44^ ^]^ Available evidence suggests that acyclovir and **LN‐7** act on different steps of HSV‐1 replication (i.e., acyclovir blocking DNA synthesis, and **LN‐7** blocking cleavage of concatemeric dsDNA that will be then packaged in procapsids). We analyzed the synergistic effects of **LN‐7** and acyclovir in HSV‐1‐infected Vero cells using CompuSyn software (ComboSyn, Paramus, NJ) based on the multiple drug effect analysis of Chou and Talalay,^[^ ^45^ ^]^ which provides the theoretical basis for the combination index (CI)–isobologram equation that allows quantitative determination of drug–drug interactions, where CI < 1, = 1, and >1 indicate synergism, additive effect, and antagonism, respectively.

First, Vero cells were infected with HSV‐1 K26‐*GFP* (m.o.i. = 0.1) in the presence of **LN‐7** at 2, 5, 10, and 20 µm, in combination with four doses of acyclovir (0.5, 1, 2, and 4 µm). Under the specified conditions, drug combinations demonstrated a low level of cell cytotoxicity (Figure S6, Supporting Information). Analysis of the viral production 24 h postinfection (p.i.) indicated synergism for most drug combinations according to all parameters, with CI values below 1 (Table S1 and Figure S7, Supporting Information). Robust synergism was detected at the highest dose of **LN‐7** tested (20 µm), with an effect level (Fa) higher than 0.9 (90% of viral inhibition), and a favorable dose reduction index above 1 (Table S1, Supporting Information).

### 2.6 Molecular Modeling and Mechanism of Action of **LN‐7**

To gain insight into the binding mode of the most potent compound **LN‐7** to the C‐terminal domain of the terminase large subunit from HSV‐1 (pUL15C), we carried out molecular dynamics (MD) simulations of the respective complexes with **LN‐7** and other pUL15C inhibitors. The crystal structure of HSV‐1 pUL15C (PDB code 4IOX) was used as the initial model. It is a trimer composed of subunits of 254 amino acids, corresponding to residues 476–730. However, it lacks the Mg^2+^/Mn^2+^ ions in the active site and no electron density is visible for loops 603–613 and 686–704, so these two stretches had to be modeled. The former loop is well conserved among herpesviruses, but the latter is conserved only in HSV‐1 and HSV‐2. Mn^2+^ cations were introduced into the active site as found in the homologous structure of HCMV pUL89C (PDB code 6EY7) containing a bound diketo acid derivative.^[^ ^17^ ^]^

**Figure 2:**
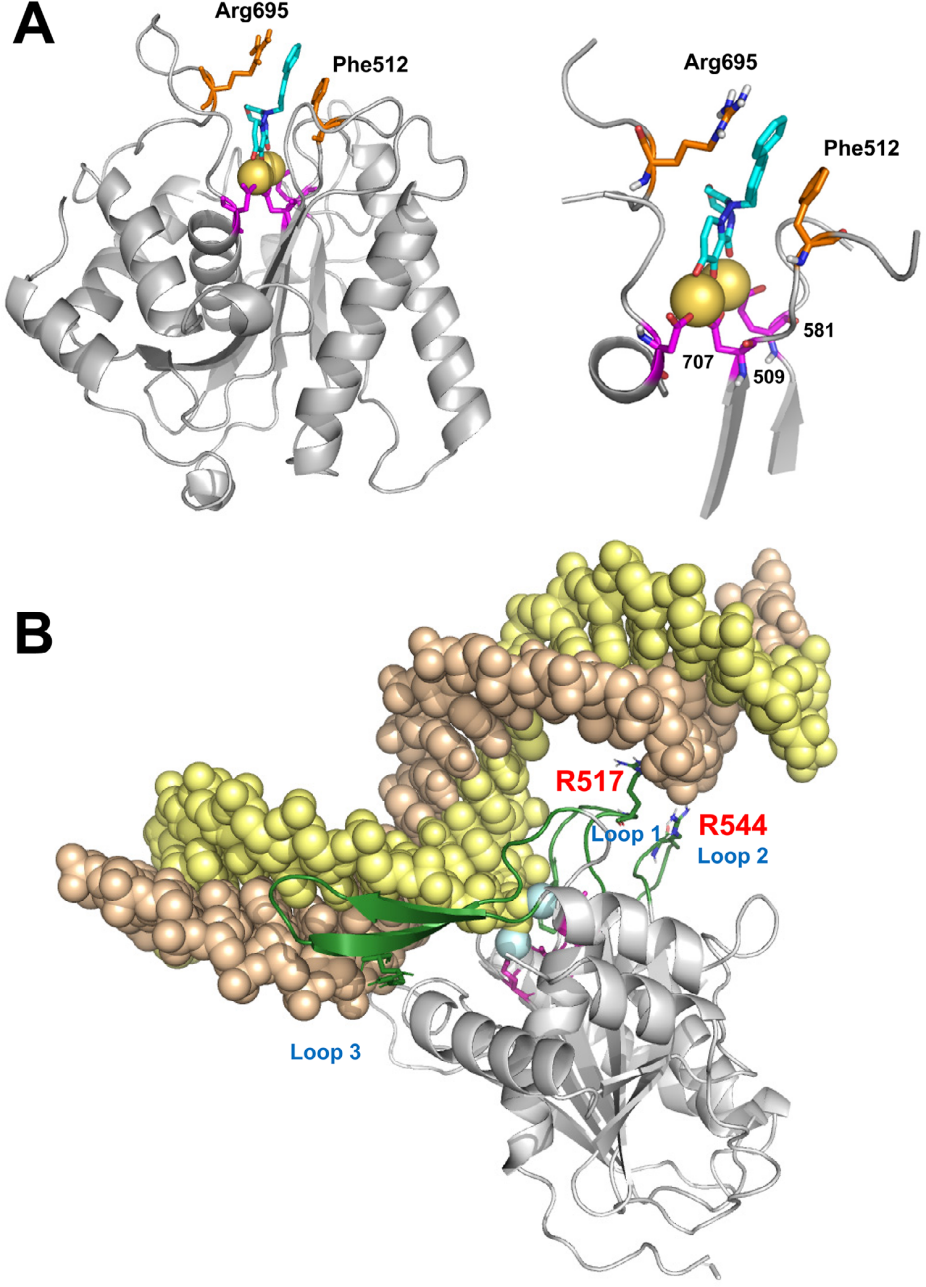
Molecular models of HSV‐1 pUL15C. A) Representative snapshot from the unrestrained MD simulation of the complex formed between **LN‐7** and the nuclease domain of pUL15. The catalytic Mn^2+^ cations within the active site are shown as yellow spheres. The side chains of catalytic residues Asp509, Glu581, and Asp707, together with stabilizing residues Phe512 and Arg695, are shown as sticks. B) Predicted structure of pUL15C bound to a double‐stranded DNA whose nucleotide sequence is identical to that of the substrate used in the inhibition assays. Loops 1, 2, and 3 correspond to residues 509–519, 541–550, and 682–705, respectively. The structure is a representative snapshot obtained after unrestrained MD (300 ns) followed by simulated annealing and energy minimization.

The deprotonated form of compound **17** (**LN‐7**) was docked into the full‐length model and the structure was energy minimized and simulated in aqueous solution using unrestrained MD at 310 K for 300 ns. Throughout the trajectory, the biaryl substituent of **LN‐7** remained sandwiched between the side chains of Phe512 and Arg695 (**Figure** **2**). As expected, the three chelating oxygens in the tricyclic structure that are shared with the “anchor domain” of baloxavir acid interact with the Mn^2+^ cations that are coordinated by the active site residues Asp509, Glu581, and Asp707. The observed stacking and cation–π interactions between the proximal benzyl group of **LN‐7** and the side chains of Phe512 and Arg695, respectively, can account for the inhibitory activity shown by some of the aryl derivatives, as indicated in Table 1. Our predicted structure suggests that **LN‐7** binding could interfere with DNA binding by restricting the accessibility of the cleavable strand to the nuclease active site.

A series of MD simulations of the pUL15C atomic model in complex with different short dsDNA molecules showed that residues 682–705 can form a β‐hairpin that is stabilized upon interaction with the minor groove of the DNA substrate whereas the loop containing Phe512 (residues 509–519) points toward the DNA major groove (Figure 2). Arg517 in the ^513^TANTRA^518^ sequence found in this loop interacts with the template DNA, as does Arg544 in the loop formed by residues 541–550. This arrangement ensures an adequate positioning of the scissile phosphodiester bond for the nucleophilic attack that is required for cleavage. The critical role of Phe512 and Arg695 was confirmed by the negligible catalytic activity shown by F512A, R695A, and F512A/R695A pUL15C variants (Figure S8, Supporting Information). Taken together, our modeling and enzymatic studies strongly suggest that, although Phe512 and Arg695 point away from the nucleic acid substrate, their interaction with the inhibitor promotes conformational changes that are incompatible with access of the DNA to the active site of UL15C.

### 2.7 Pharmacokinetics and Toxicity Studies

**Figure 3:**
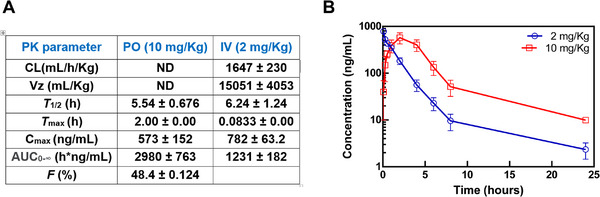
Pharmacokinetics of compound **17** (**LN‐7**) in rats. A) Pharmacokinetics parameters obtained for intravenous (iv) and oral administration (po). B) Plasma concentration–time profiles of **LN‐7** in rats following oral administration (at 2 and 10 mg kg^−1^). Studies were carried out with three animals per group.

Compound **17** (**LN‐7**) was evaluated for pharmacokinetics in male Sprague‐Dawley rats at 2 and 10 mg kg^−1^ for intravenous (iv) and oral administration (po) (**Figure** **3**). **LN‐7** exhibited excellent pharmacokinetic profiles. Among all reported data, the *T*_max_ of **LN‐7** (2 h, po) was consistent with the expected time required for the onset of action, while the analysis revealed slow intrinsic clearance (CL = 1647 ± 230 mL h^−1^ kg^−1^, iv), a favorable half‐life (*T*_1/2_ = 5.54 ± 0.676 h po and 6.24 ± 1.24 h iv), and an optimal volume of distribution (AUC = 2980 ± 763 h × ng mL^−1^ po and 1231 ± 182 h × ng mL^−1^ iv). Furthermore, **LN‐7** exhibited a promising oral bioavailability (*F* = 48.4 ± 0.124%).

The favorable PK profile of the compound warranted its evaluation in safety studies carried out in BALB/c mice. Mice were randomly distributed in different groups and received oral doses of 0 (control), 25, 50, and 75 mg kg^−1^ of **LN‐7**, as well as acyclovir at 50 mg kg^−1^ for five consecutive days (Figure S9, Supporting Information). The mice did not show any toxic symptoms or mortality immediately or during the posttreatment period of 12 days. No abnormal behaviors or significant changes in body weight were observed during the execution of the experiment. White blood cell counts and hematocrit values were similar in all groups of mice, and liver function and renal markers were not affected by the administration of different compounds.

### 2.8 Efficacy of Compound **17** (**LN‐7**) against HSV‐1 in Mice

**Figure 4:**
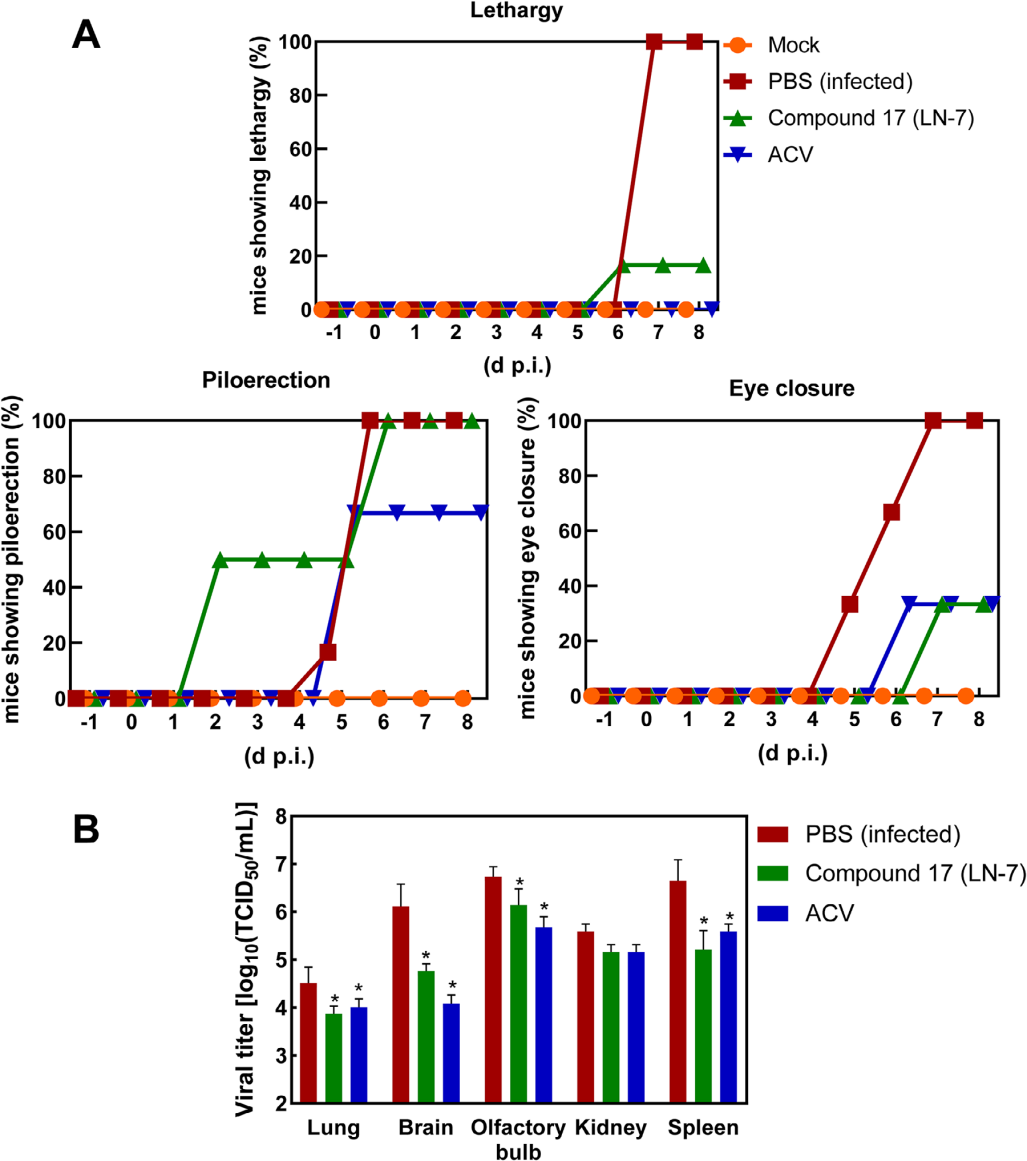
Antiviral effects of compound **17** (**LN‐7**) in infected BALB/c mice. A) Clinical signs (lethargy, piloerection, and eye closure) of BALB/c mice infected with HSV‐1 and orally treated with acyclovir (ACV) and **LN‐7**. Six mice per group were infected after intranasal administration of HSV‐1 (10^5^ PFU), and then treated with PBS, **LN‐7** (75 mg kg^−1^), or ACV (50 mg kg^−1^). Treatment lasted four days, starting one day before the infection, and ending on day 3 postinfection. Changes in body weight and clinical signs were monitored daily. Statistical comparisons were performed using the Gehan–Breslow–Wilcoxon test. B) HSV‐1 titers in the lungs, brain, olfactory bulb, kidneys, and spleen of BALB/c mice. At day 8 p.i., mice were euthanized and relevant organs and tissues were extracted. Progeny virus was titrated in Vero cells to determine the 50% tissue culture infective dose (TCID_50_) mL^−1^. Mean (*n* = 6) ± standard error of the mean (S.E.M.) are shown, and results are compared by two‐way ANOVA with Tukey's post hoc test comparisons (*p < 0.05).

Based on the lack of toxicity at different doses, we tested the in vivo efficacy of **LN‐7** against HSV‐1 in BALB/c mice at the highest dose (i.e., 75 mg kg^−1^). At first, this was done in a prophylactic setting, in which mice were challenged intranasally with the virus one day after the first administration of the compound, and then received equal doses of the compound every day for four consecutive days. All mice were euthanized on day 8 postinfection. All phosphate‐buffered‐saline (PBS)‐treated mice were lethargic and showed piloerection and eye closure at day 7 p.i., in contrast to uninfected mice that remained healthy during the whole experiment (**Figure** **4**). Clinical symptoms of the infection were observed in both groups of treated mice (i.e., mice treated with **LN‐7** at 75 mg kg^−1^ and those treated with acyclovir at 50 mg kg^−1^), although morbidity was higher for the **LN‐7**‐treated animals. Interestingly, the antiviral effects of **LN‐7** were demonstrated after measuring HSV‐1 titers in sacrificed mice. The obtained results showed that viral titers decreased around one to two orders of magnitude in the lung, brain, olfactory bulb, and spleen tissues of mice treated with **LN‐7**, with reductions that were similar to those obtained for acyclovir‐treated mice (Figure 4).

**Figure 5:**
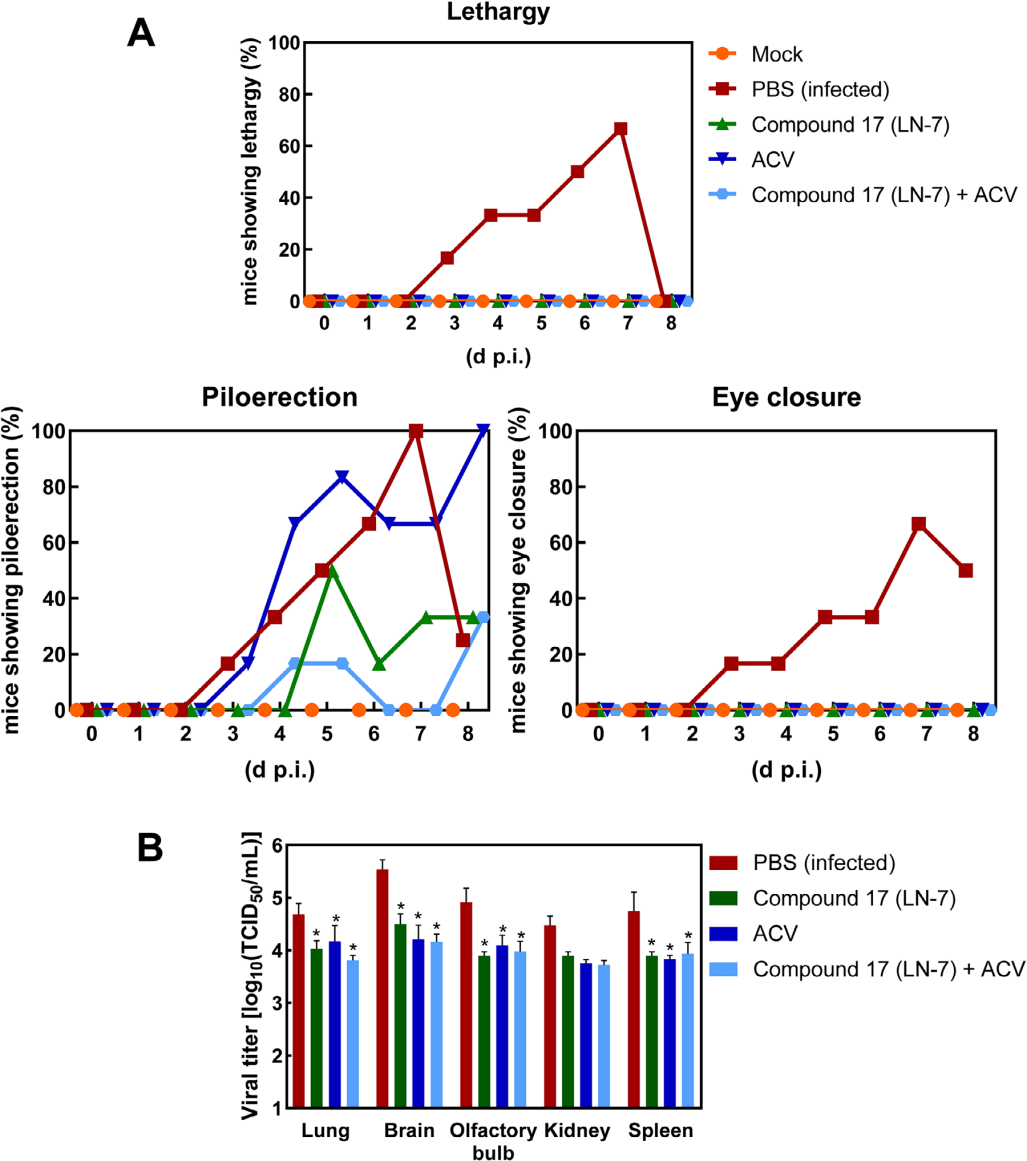
Antiviral effects of compound **17** (**LN‐7**) in infected BALB/c mice. A) Clinical signs of BALB/c mice infected with HSV‐1 and orally treated with ACV, **LN‐7**, or the combination of both drugs. Six mice per group were infected intranasally with HSV‐1 (10^5^ PFU) and treated with PBS, **LN‐7** (75 mg kg^−1^), ACV (50 mg kg^−1^), or the combination **LN‐7** (75 mg kg^−1^)/ACV (50 mg kg^−1^). Mice were treated for four consecutive days (0, 1, 2, and 3 p.i.). Changes in body weight and clinical signs were monitored daily. The analyzed clinical symptoms were piloerection, lethargy and staggering, eye closure, and hunched posture. Individual symptoms are considered as positive when a mouse shows the signs at any time during the day. Mock‐infected mice did not show any symptoms throughout the experiment. Data from the PBS (infected) group on days 7 and 8 p.i. refer to 6 mice (2 mice died on day 6 p.i.). B) HSV‐1 titers in the lungs, brain, olfactory bulb, kidneys, and spleen of BALB/c mice. At day 8 p.i., mice were euthanized and lung, brain, and olfactory bulb were extracted. Progeny virus was titrated in Vero cells to determine the 50% TCID_50_ mL^−1^. Mean (*n* = 6) ± S.E.M. are shown, and statistical comparisons were made by two‐way ANOVA with Tukey's post hoc test comparisons (*p < 0.05).

Considering the positive results of experiments involving the prophylactic administration of the compounds, we also tested the effects of **LN‐7** on BALB/c mice after intranasal infection with HSV‐1. As in the previous experiments, we tested the effects of **LN‐7** at 75 mg kg^−1^ and acyclovir at 50 mg kg^−1^, as well as the combination of **LN‐7** (75 mg kg^−1^) plus acyclovir (50 mg kg^−1^). Drugs were given once a day for four consecutive days. All treated mice survived until the end of the experiment (8 days postinfection), although 2 out of 6 mice in the untreated group of animals died on day six after infection. We did not observe significant weight losses in the treated mice and clinical symptoms were alleviated in all of the treated groups (**Figure** **5**). None of the animals showed signs of lethargy or eye closure, and piloerection was observed in mice treated with acyclovir and **LN‐7**, and was less frequent in animals receiving the combination of both compounds. Interestingly, and in agreement with the results obtained in the prophylactic setting, we observed significant reductions in the viral titers in all analyzed tissues of treated mice, except for kidney tissue. Differences between **LN‐7** at 75 mg kg^−1^, acyclovir at 50 mg kg^−1^, and the combination of both compounds were not significant, and the largest reductions in viral titers were observed in brain (Figure 5B). Taken together, the lack of toxicity at different doses in mice and its good oral bioavailability, combined with its efficacy against HSV‐1 in the mouse model support the development of **LN‐7** as a first‐in‐class antiviral agent against alphaherpesviruses.

## 3 Discussion

Developed in Japan by the pharmaceutical company Shionogi, baloxavir marboxil is an antiviral drug approved for the treatment of seasonal flu and for postexposure prophylaxis of influenza.^[^ ^46^, ^47^ ^]^ The approved compound is an oral prodrug that inside the cell is converted to baloxavir acid (also known as baloxavir). The active molecule targets the endonuclease function of the viral PA polymerase subunit and blocks transcription of the viral mRNA [reviewed in refs. [40, 48]]. Baloxavir acid has potent broad‐spectrum activity in cell culture against different types of influenza viruses,^[^ ^49^, ^50^, ^51^ ^]^ as well as members of the order *Bunyavirales* (e.g., hantavirus, Bunyamwera virus, La Crosse virus, and Crimean–Congo hemorrhagic fever orthonairovirus).^[^ ^52^, ^53^, ^54^ ^]^

The catalytic site of the influenza virus PA endonuclease is a negatively charged pocket with a cluster of three acidic residues (Glu80, Asp108, and Glu119) and two divalent cations (Mg^2+^ or Mn^2+^).^[^ ^55^, ^56^ ^]^ Baloxavir acid chelates the two divalent metal ions at the active site^[^ ^57^ ^]^ and acts as a tight‐binding inhibitor of the endonuclease.^[^ ^58^ ^]^ The metal‐binding pharmacophore of baloxavir acid derives from substituted polycyclic pyridines that were originally designed as potential HIV‐1 integrase inhibitors.^[^ ^46^ ^]^ Leveraging the concept of “privileged structures,”^[^ ^59^ ^]^ we recognized baloxavir acid as a foundational template due to its established metal‐chelating properties and broad‐spectrum antiviral activity.^[^ ^40^ ^]^ By strategically modifying its tricyclic core and introducing hydrophobic substituents (e.g., biaryl moieties at position 2 of the triazinanone ring), we aimed to enhance target specificity for HSV‐1 pUL15C while minimizing off‐target effects. This approach aligns with scaffold re‐evolution principles, where iterative structural refinement of privileged frameworks can unlock novel therapeutic applications.^[^ ^59^ ^]^

The nuclease domain of the terminase complex of herpesviruses such as HSV‐1 shares a similar fold and catalytic site when compared with HIV integrase and RNase H enzymes. In this study, we show that derivatives of the chelating pharmacophore present in baloxavir can be effective inhibitors of HSV‐1 pUL15C in enzymatic assays. The crystal structure of HSV‐1 pUL15C^[^ ^15^ ^]^ and our modeling studies reveal an open conformation that facilitates access of small molecules to the catalytic site, thereby explaining why β‐thujaplicinol and small baloxavir derivatives such as compounds **2** and **3** show good inhibitory activity in enzymatic assays. However, bulkier hydrophobic substituents at position 2 of the triazinanone ring often result in a significant increase of the IC_50_. Nevertheless, the highest inhibitory activity of the diphenyl‐substituted molecules was rationalized by results from molecular modeling studies suggesting that stacking interactions of the proximal phenyl substituent with the side chains of Phe512 and Arg695 impede access of the nucleic acid substrate to the catalytic site of the enzyme. Both residues are highly conserved among the nucleases of herpesviruses and their substitution by alanine leads to the loss of activity, as demonstrated for mutant enzymes F512A, R695A, and F512A/R695A.

Our results provide strong evidence in favor of pUL15C as the primary target of **LN‐7**. However, the inhibitory activity of β‐thujaplicinol, synthetic hydroxytropolone analogs, and other chelating agents has been previously demonstrated for other viral nucleases, most notably HSV‐1 pUL12.^[^ ^28^, ^30^, ^60^ ^]^ This enzyme is an exonuclease required for infectious virus production and cell‐to‐cell spread. Other potential targets of chelating agents in HSV‐1 and other herpesviruses include the recombinase ICP8, the exonuclease activity of the viral DNA polymerase (UL30 in HSV‐1), and host factors such as the flap endonuclease 1 which has been implicated in the reinitiation of stalled viral replication forks during HCMV DNA synthesis.^[^ ^61^ ^]^ Considering their catalytic activities and mechanism of action, we cannot rule out the possibility that **LN‐7** binding to any of those targets could have some additional effect on its antiviral efficacy.

While various compounds exhibit promising inhibitory activity in simple enzymatic assays, only five of them showed a significant antiviral effect in cell cultures infected with HSV‐1. A good activity in enzymatic assays does not always translate into high efficacy in cell culture, due to barriers in cell entry (permeability), metabolic instability, reduced target accessibility, and the need for cellular activation. Interestingly, in our study, we demonstrate that one of the inhibitors (**LN‐7**) shows remarkable antiviral activity against HSV‐1 and HSV‐2 in cell culture. In addition, **LN‐7** demonstrated in vivo favorable pharmacokinetic properties in rats (*F* = 48.4%) and lack of toxicity in experiments in mice when administered at the highest dose (75 mg kg^−1^).

The antiviral activity of **LN‐7** in HSV‐1‐infected mice was further supported after determining viral titers in lung and brain tissues. Although the efficacy of **LN‐7** was not as high as in the case of acyclovir, the good pharmacokinetic and toxicity profiles of the compound leave the door open for improvements in drug administration (e.g., dose, route, etc.). Furthermore, drug combination experiments in vitro suggest a benefit of combining acyclovir with **LN‐7**. By targeting different viral proteins, combined administration of antivirals could reduce the risk of emergence of resistant strains, and possibly lower their toxicity in comparison with high‐dose monotherapies. However, we did not find significant differences in viral titer reduction in mice treated with the compounds alone versus those treated with combinations of acyclovir and **LN‐7**, suggesting that further research will be necessary to determine the optimal doses for combination therapies. Our study provides seminal evidence that it is possible to interfere with the termination of HSV‐1 replication by inhibiting the viral nuclease, and **LN‐7** is therefore a first‐in‐class compound targeting this step in the viral replication cycle.

## 4 Experimental Section

### Ethical Statements

Studies with mice were carried out in strict accordance with the European Commission legislation for the protection of animals used for scientific purposes (directives 86/609/EEC and 2010/63/EU). Mice were maintained under specific pathogen‐free conditions at the Centro de Biología Molecular Severo Ochoa (CBM) (Spanish National Research Council (CSIC)–UAM) animal facility. The protocol for the treatment of the animals was accepted by the ″Comité de Ética del CSIC (Spain) and approved by the “Consejería de Medio Ambiente, Agricultura e Interior de la Comunidad de Madrid” (PROEX 333.7/23). Pharmacokinetics studies with rats were approved by the Institutional Animal Care and Use Committee (IACUC) of Precedo Pharmaceuticals Co. Ltd. (IACUC‐20240311‐1).

### Compound Synthesis and Characterization

Synthesis procedures and compound characterization are described in the Supporting Information. Provided information included ^1^H and ^13^C NMR spectra, and high‐resolution mass spectrometry spectra of compounds **1**–**40**, as well as the high‐performance liquid chromatography purification profile of compound **17** (**LN‐7**).

### Enzymatic Assays

Nuclease inhibition assays were performed with HSV‐1 pUL15C expressed in *Escherichia coli* BL21(DE3) cells and purified as previously described.^[^ ^15^ ^]^ A pET28b plasmid (Novagen, Madison, WI) containing the pUL15C‐encoding residues and N‐terminal His tag was kindly provided by Dr. Sandra K. Weller (University of Connecticut School of Medicine, USA). The substrate was a double‐stranded DNA template containing a ^32^P‐labeled oligonucleotide (5′‐GATGAGACCACCAATAAAAAGGCTCAATTAATG‐3′) annealed to a 33mer complementary DNA strand (5′‐CATTAATTGAGCCTTTTTATTGGTGGTCTCATC‐3′). DNA cleavage kinetics were determined at 37 °C in 20 mm Tris‐HCl (pH 7.0), containing 1 mm MnCl_2_, 10 mm NaCl, and 5% dimethyl sulfoxide (DMSO).^[^ ^32^ ^]^ The concentration of labeled dsDNA substrate was 100 nm, and assays were carried out with an enzyme concentration of 400–600 nm. Nuclease cleavage was triggered by adding the dsDNA to a solution containing the enzyme and the inhibitor (at the appropriate concentration). Reaction aliquots were withdrawn after 15 min and immediately terminated by adding an equal volume of loading buffer [10 mm ethylenediaminetetraacetic acid (EDTA) in 90% formamide containing xylene cyanol FF (3 mg mL^−1^) and bromophenol blue (3 mg mL^−1^)]. Hydrolysis products were fractionated by 20% denaturing urea polyacrylamide electrophoresis [19:1 acrylamide:bisacrylamide] and quantitatively analyzed by phosphorimaging using a BAS1500 scanner (Fuji) and the Tina version 2.09 software (Raytest Isotopenmessgerate Gmbh, Staubenhardt, Germany). Cleavage patterns observed with the 33‐base pair DNA duplex used as substrate are provided in Figure S10 (Supporting Information). IC_50_ values were determined from dose–response curves after calculating the inhibitory activity (i.e., percentage inhibition), in the presence of the tested compounds at concentrations in the range 0.25–50 µm. Reference values for maximum activity were obtained in the absence of inhibitors, in a buffer containing 5% DMSO. Data analyses were performed using the GraphPad Prism version 6.00 software (GraphPad Software, La Jolla, CA, USA).

### Mutant Studies

Site‐directed mutagenesis was carried out using the standard QuickChange protocol (Stratagene). The pET28b plasmid containing the WT HSV‐1 pUL15C was used as template and the mutagenic primers were 5′‐CGTGGATCCCGCGGCCACGGCCAACACCCGAG‐3′ and 5′‐CTCGGGTGTTGGCCGTGGCCGCGGGATCCACG‐3′ for F512A; and 5′‐CCCAACACGGACGTCGCTACGTATTCCGGAAAACG‐3′ and 5′‐CGTTTTCCGGAATACGTAGCGACGTCCGTGTTGGG‐3′ for R695A. The double mutant was prepared with the F512A mutagenic primers, using as template the pET28b plasmid containing the R695A mutation in the pUL15C‐coding region. After mutagenesis, the entire pUL15C‐coding regions were sequenced and if correct, used for expression and purification, as described above for the WT enzyme. DNA cleavage kinetics were carried out at 37 °C in 20 mm Tris‐HCl (pH 7.0), containing 1 mm MnCl_2_, 10 mm NaCl, and 5% DMSO, as described above, but the substrate used was a ^32^P‐labeled DNA oligonucleotide of 42 bases with a hairpin structure (5′‐TATGTATTTAGGATTGGGATTATACCCAATCCTAAATACATA‐3′), similar to the one described by Masaoka et al.^[^ ^32^ ^]^

### Antiviral Assays

The cellular antiviral activities of compounds described above were determined in Vero cells, a cell line derived from the kidney tissue of an adult African green monkey (ATCC CCL‐81). The virus used was a wild‐type HSV‐1 (F strain) (GenBank accession number GU734771) construct obtained by fusing *GFP* to the HSV‐1 capsid protein VP26.^[^ ^43^ ^]^ The construct (designated as HSV‐1 K26‐*GFP*) was a kind gift from Dr. P. Desai (Johns Hopkins University, Baltimore, MD, USA). ACV‐resistant HSV‐1 strains were kindly provided by Dr. Enrique Tabarés (Universidad Autónoma de Madrid, Spain). The recombinant strain F‐TKGF encoded an intact ICP4 protein with an amino terminal deletion of thymidine kinase coding region, which included all of the first 316 codons, followed by the *EGFP* coding sequence.^[^ ^62^ ^]^ Δ305 has a 700‐bp deletion in the *tk* gene, introduced in the HSV‐1 F strain.^[^ ^63^ ^]^ WT HSV‐2‐*GFP* carrying the complete genome of HSV‐2 and the *GFP* ^[^ ^64^ ^]^ was kindly provided by Dr. Y. Kawaguchi (University of Tokyo, Japan). Cells were cultured in low‐glucose Dulbecco's modified Eagle medium (DMEM) supplemented with 5% fetal bovine serum (FBS), penicillin (50 U mL^−1^), and streptomycin (50 µg mL^−1^) at 37 °C in a humidified atmosphere of 5% CO_2_.

Briefly, 3.5 × 10^4^ cells per well were seeded in 48‐well plates and cultured in DMEM media containing 5% FBS to form a monolayer of cells. Compounds were diluted in DMEM with 5% FBS and added to cell monolayers in triplicate for 1 h at different concentrations (2, 5, 10, 20 µm), at 37 °C. Two controls were added in this experiment: i) mock cells, which were subjected to the same conditions as the rest of the samples, but were not infected, and ii) nontreated cells, which were infected with the virus but not treated with any compound. HSV‐1 K26‐*GFP* and F‐TKGF were diluted in DMEM medium without FBS at a m.o.i. of 0.1. Cells were then incubated at 37 °C for 1 h with the mix virus compound. After 1 h of viral adsorption, the virus‐containing inoculum was removed, and wells were washed once in PBS and maintained in a fresh culture medium containing the compounds (at appropriate concentrations). Cells were incubated at 37 °C for an additional 24 h, and plates were then inspected by phase contrast for toxicity. Cells in wells showing no obvious toxicity were processed for flow cytometry. Briefly, cells were dissociated by incubation for 3 min with 0.05% trypsin/0.1% EDTA at room temperature, washed, and fixed in PBS with 1% bovine serum albumin, 1% FBS and 2% paraformaldehyde for 15 min. Then, cells were rinsed, resuspended in PBS, and analyzed using a FACSCalibur Flow Cytometer (BD Biosciences). Data were processed with FlowJo software (BD, version 10.6.2). The EC_50_, which indicated the concentration of the compound that led to a 50% reduction in viral infection was determined from dose–response curves as determined above. The antiviral activity of compound **17** (**LN‐7**) was also tested against ACV‐resistant HSV‐1 strains F‐TKGF and Δ305, and HSV‐2 (wild‐type strains G and Lovelace, and the recombinant HSV‐2 *GFP* strain) in Vero cells, using ACV as a control in the experiments carried out with resistant strains.

In experiments carried out with the ACV‐resistant strain Δ305 or with wild‐type HSV‐1 F, cells were initially treated with **LN‐7** or ACV (at 5 µm concentration) for 1 h before the infection with the virus at a m.o.i. of 0.3. In antiviral assays with HSV‐2 strains, cells were treated with **LN‐7** (at 2, 5, 10, and 20 µm concentration) for 1 h before infection using a m.o.i. of 0.1. In all cases, cells were then incubated with the virus at 37 °C for 1 h, and after removing the virus‐containing inoculum, cultures were maintained at 37 °C for an additional 24 h in the presence of the compound, at the indicated concentrations. After the incubation, cells were treated as above and tissue culture infective doses mL^−1^ (TCID_50_ mL^−1^) were determined using an end‐point dilution assay in the assays with wild‐type strains, or processed for flow cytometry (recombinant HSV‐2 *GFP*).

### Time‐of‐Addition Assays

Time‐of‐addition experiments with HSV‐1 K26 *GFP* were performed to study the phase of infection at which candidate compounds exerted their antiviral activity. Vero cells were grown in 48‐well culture plates at 37 °C as described above. The virus (HSV‐1 K26‐*GFP* at a m.o.i. of 0.1) was added to the cultures and maintained for 1 h in the presence or absence of tested compounds at a concentration of 10 µm. Several protocols were used in which candidate compounds were added before, during, and/or after viral infection (Figure S5, Supporting Information). After 1 h of viral adsorption, the virus was washed and replaced with fresh DMEM medium containing 5% FBS, with or without the tested compounds, according to the protocol. At 24 h postinfection, cells were fixed for flow cytometry analysis and analyzed as described above.

### Analysis of Cell Viability

The cytotoxicity of the compounds was quantified in the Vero cell line using a CellTiter 96 Aqueous NonRadioactive Cell Proliferation Assay Kit (Promega) based on the 3‐(4,5‐dimethylthiazol‐2‐yl)‐5‐(3‐carboxymethoxyphenyl)‐2‐(4‐sulfophenyl)‐2*H*‐tetrazolium (MTT) reagent. Nonconfluent monolayers of cells plated in 96‐well tissue culture plates were grown for 24 h before use. Cells were then treated for 24 h with the compounds at concentrations ranging from 0 to 100 µm, diluted in DMEM supplemented with 5% FBS. Four replicates were performed for each concentration. The cells were then incubated with MTT reactive as indicated by the manufacturer of the kit, and the resulting colored solution was quantified using the scanning multiwell spectrophotometer iMarkTM Microplate Reader (BioRad), measuring the absorbance at 595 nm. The readouts obtained from the MTT assay were further normalized to the value of untreated cells. Finally, CC_50_ values, which indicated the concentration of the compound that led to a 50% reduction in cell viability were calculated with GraphPad Prism software (version 8.0.1) using the four‐parameter variable slope algorithm with the bottom value set to zero.

### Combination Experiments

The antiviral effect produced by the combination of acyclovir with **LN‐7** was studied in Vero cells with HSV‐1 K26‐*GFP*. Vero cells were infected with HSV‐1 K26‐*GFP* (m.o.i. = 0.1) in the presence of **LN‐7** at concentrations of 2, 5, 10, and 20 µm, each one in combination with four doses of acyclovir (0.5, 1, 2, and 4 µm). The antiviral effects were determined 24 h after the infection. Results were analyzed using the method of Chou and Talalay^[^ ^45^ ^]^ to determine if the combination produced synergistic, additive, or antagonistic effects. The resulting data were analyzed using the CompuSyn software (ComboSyn, Paramus, NJ). The assignment of synergy or antagonism was based on the theoretical values obtained for the CI–isobologram equation that facilitated a quantitative determination of drug interactions. The estimated CI value indicated whether the combined effect was synergistic (CI <1), additive (CI = 1), or antagonistic (CI >1).

### Pharmacokinetics Studies in Rats

Six male Sprague‐Dawley rats (180–200 g) were randomly divided into two groups to receive oral administrations of **LN‐7** at 2 mg kg^−1^ (iv administration) or 10 mg kg^−1^ (po administration). **LN‐7** was dissolved in DMSO, and then diluted at a concentration of 1 mg mL^−1^ in 3% DMSO, 10% solutol HS‐15, and 87% double‐distilled water for dosing. Rats were purchased from Beijing Vital River Laboratory Animal Technology Co., Ltd. (Qualification No. SCXK(SH) 2021‐0011). Animals were subjected to 12 h alternating light and dark cycles, ad libitum access to drinking water and food, and fasting for 12 h prior to the initiation of treatments. For the oral dose at 10 mg kg^−1^, blood samples were collected at 0.083, 0.25, 0.5, 1, 2, 4, 6, 8, and 24 h postadministration. For the intravenous dose (at 2 mg kg^−1^), blood samples were collected at 0.083, 0.25, 0.5, 1, 2, 4, 6, 8, and 24 h postadministration. Twenty µL of blood were mixed with a 200 µL solution of methanol:acetonitrile (1:1), vortexed for 5 min, and centrifuged (4000 rpm, 4 °C, 10 min) to obtain plasma within 30 min of sampling. The supernatant was analyzed by liquid chromatography–tandem mass spectrometry. Chromatographic separation was performed using an ACQUITY UPLC HSS T3 column (2.1 × 50 mm, 1.8 µm). The mobile phase was prepared with 0.025% formic acid in water (A) and acetonitrile (B). Gradient elution was carried out at a flow rate of 0.8 mL min^−1^. The samples were analyzed in a gradient from 20% of B to 95% of B in 1.6 min, maintained at 95% for 0.3 min, then decreased back to 20% of B within 0.01 min, and finally maintained at 20% for 0.29 min. Mass spectrometric detection was performed with an electrospray ionization source operating in positive ion mode. Quantification was achieved using multiple reaction monitoring with the following transitions: *m*/*z* 403.15.

Pharmacokinetic parameters such as AUC, *T*_1/2_, and *C*_max_ were calculated with the WinNonlin software. The absolute oral bioavailability in rats was calculated based on the ratio of AUC after oral and intravenous administration, using the equation

(1) $$F\left(\%\right)=100x\left\{\left[\left(\mathrm{AUC},\;\mathrm{po}\right)x\left(\mathrm{Dose},\;\mathrm{iv}\right)\right]/\left[\left(\mathrm{AUC},\;\mathrm{iv}\right)x\left(\mathrm{Dose},\;\mathrm{po}\right)\right]\right\}$$

where *F* represents the absolute bioavailability; AUC represents the area under the concentration–time curve; and Dose refers to the administered amount of test compound.

### Toxicity Assays in Mice

BALB/c mice were purchased from Charles River Laboratories Spain and maintained at the Animal Facility of the CBM (Madrid, Spain). The toxicity of the test compound **LN‐7** was investigated in BALB/c mice dosed as a solution at 25, 50, and 75 mg kg^−1^. The compound was formulated in 3% DMSO + 10% solutol HS‐15 + 87% double‐distilled water (20 mg mL^−1^). Animals had free access to food and water throughout each study. Thirty BALB/c mice were distributed in five different groups (*n* = 6), three of them receiving the specific doses of **LN‐7**, one receiving acyclovir (50 mg kg^−1^), and the fifth one inoculated with PBS. Compounds were orally administered for five consecutive days. Animals were monitored daily for clinical signs (piloerection, lethargy, eye closure), body weight loss, and survival. Oral administration and symptom monitoring was performed by researchers that were aware of the treatment condition (group allocation) for each animal. The general condition of mice was assessed by a predefined score system ranging from 1 to 4: 1 – no indication of pain and distress; 2 – mild or anticipated pain and distress; 3 – moderate pain and distress; and 4 – severe pain and distress. Animals with score 4 were defined as the humane endpoint on which the animal must be euthanized. On day 12, mice were sacrificed and whole blood was obtained by cardiac puncture. Several parameters were analyzed to monitor renal, hepatic, and immunological basic profiles. Body weight was also monitored to exclude weight loss or lack of weight gain that would be indicative of toxicity.

### HSV‐1 Infection Model in BALB/c Mice

BALB/c mice were used to assess the activity of compound **17** (**LN‐7**), after viral titration in specific organs. Mice were maintained in the Biosafety level 2 Animal Facility of the CBM and separated into four groups of six individuals each, and after acclimatization, all of them were infected intranasally with HSV‐1 (10^5^ PFU, 30 µL per mice). Compounds were orally administered. In the prophylactic assay, PBS, **LN‐7** (75 mg kg^−1^), or acyclovir (50 mg kg^−1^) were given at days −1, 0, 1, 2, and 3 postinfection (d.p.i.). In experiments aimed to evaluate the therapeutic effect of the drug candidate, PBS, **LN‐7**, acyclovir, or a combination of **LN‐7** (75 mg kg^−1^) plus acyclovir (50 mg kg^−1^) were administered at days 0, 1, 2, and 3 d.p.i., after intranasal infection with HSV‐1. Animals were monitored daily for clinical signs, body weight loss, and survival. Infection, oral administration, and symptom monitoring were performed by researchers that were aware of the treatment condition (group allocation) for each animal. At day 8 postinfection, animals were euthanized and organs (brain, lungs, olfactory bulb, kidneys, and spleen) were extracted for virus quantitation. Organs were homogenized using TissueLyzer, subjected to three rounds of freezing/thawing, and stored at −80 °C before titration by end‐point dilution and determination of the TCID_50_ mL^−1^.

### Molecular Model of the C‐Terminal Nuclease Domain of HSV pUL15C

The X‐ray crystal structures of the C‐terminal nuclease domain of the terminase large‐subunit pUL15 (pUL15C) from HSV‐1 strain 17, solved at 2.46 Å resolution (PDB code 4IOX, chain A, which included the loop comprising residues ^510^Pro‐Thr^521^)^[^ ^15^ ^]^ and the Mn^2+^‐soaked tripartite terminase subunit 3 (TRM3 also known as UL89) of human herpesvirus 5 strain AD169 in complex with an α,γ‐diketoacid inhibitor (ligand code C3W), solved at 2.9 Å resolution (PDB code 6EY7),^[^ ^17^ ^]^ were employed in the initial molecular modeling studies (Figure S11, Supporting Information). The structural alignment of these two nuclease domains using the Dali web server^[^ ^65^ ^]^ allowed placement of the missing Mn^2+^ ions in the active site of pUL15C as well as the proper reorientation of the side chains of the catalytic residues (Asp509, Glu581, Asp706, and Asp707) to optimize the unique metal ion coordination geometry. The missing protein loop comprising residues 602–614 was obtained from PDB entry 6M5T solved by cryo‐electron microscopy at 3.60 Å.^[^ ^14^ ^]^ For completeness, five models of the full‐length pUL15 protein created with AlphaFold^[^ ^66^ ^]^ were also considered. In addition, the RCD+ server^[^ ^67^ ^]^ was used to generate alternative loop conformations of the intrinsically disordered regions.

### Building of Inhibitors and Placement in the pUL15C Nuclease Active Site

The X‐ray crystal structure of baloxavir marboxil^[^ ^68^ ^]^ deposited in the Cambridge Structural Database with code NAXTEP provided the template for inhibitor model building using the editing tools available in PyMOL v. 2.6 (Schrödinger, LLC. 2024). This molecular graphics program was also employed for molecular visualization and figure preparation. Ab initio geometry optimization of selected hydroxypyridone‐containing ligands in their enolate form, followed by derivation of atom‐centered RESP charges^[^ ^69^ ^]^ was achieved by using the density functional tight‐binding method, a 6‐31G* basis set, and the isoelectric focusing (IEF)‐SCRF continuum solvent model for water,^[^ ^70^ ^]^ as implemented in program Gaussian 09 (Revision D.01).^[^ ^71^ ^]^ The structural superposition of PDB entries 4IOX and 6EY7 also informed about the most plausible ligand binding geometry because best‐fit superpositions of the three exocyclic oxygens of LN7 onto the three equivalent oxygens of the bound α,γ‐diketoacid revealed that only one solution was compatible with the binding site architecture and the need to interact simultaneously with the two active‐site metal ions. This solution was also consistent with i) the binding mode of β‐thujaplicinol at the RNase H active site of HIV‐1 reverse transcriptase (PDB codes 3IG1 and 3K2P),^[^ ^72^ ^]^ ii) the active site arrangement of the nuclease domain of the large terminase subunit gp2 of bacterial virus Sf6 (PDB code 5C2F),^[^ ^73^ ^]^ and iii) cocrystal structures of baloxavir acid and the N‐terminal endonuclease domain of influenza virus polymerase PA (PDB codes 8T5V, 8T5W, 8T5Z, 8T67, 8T6Z, 8T81, 8T94, and 8VGZ).^[^ ^74^ ^]^ 3D models of DNA linear duplexes, DNA hairpins, and their complexes with HSV pUL15C were obtained as described in the Supporting Information, as well as methods used for energy minimization, unrestrained MD simulations, and the analysis of MD trajectories.

### Statistics

All statistical analyses were performed using GraphPad Prism (version 8.0.1, GraphPad Software, Inc.). Data were subjected to either Gehan–Breslow–Wilcoxon tests or two‐way analysis of variance (ANOVA) followed by the Tukey's post hoc test to determine significant differences between groups, and *p* values < 0.05 were considered statistically significant. For the CC_50_ and EC_50_ values, nonlinear fit regression models were used (four parameters).

## Conflict of Interest

The CSIC has applied for a patent (EP25382332) covering HSV‐1 pUL15C nuclease inhibitors reported in this study and related compounds, which has not been published and is pending. S.A., K.T., J.A. L.‐C., X.L., P.Z., and L.M.‐A. are listed as inventors. The remaining authors declare no competing interests.

## Author Contributions

S.A., K.T., and J.Z. contributed equally to this work. Conceptualization: S.A., K.T., J.A.L.‐G., P.Z., L.M.‐A.; methodology: S.A., K.T., J.Z., G.P.‐P., N.L.‐C., R.B.‐M., D.G.‐T., L.Z., F.G., J.A.L.‐G., X.L., P.Z., L.M.‐A.; investigation: S.A., K.T., J.Z., G.P.‐P., N.L.‐C., R.B.‐M., D.G.‐T., L.Z., F.G., J.A.L.‐G., X.L., P.Z., L.M.‐A.; visualization: F.G., L.M.‐A.; supervision: J.A.L.‐G., X.L., P.Z., L.M.‐A.; writing—original draft: S.A., K.T., P.Z., L.M.‐A.; writing—review and editing: S.A., K.T., F.G., J.A.L.‐G., P.Z., L.M.‐A.

## Supporting information



Supporting Information

Supporting Information

## Data Availability

The data that support the findings of this study are available in the Supporting Information of this article.

## References

[1] C. James , M. Harfouche , N. J. Welton , K. M. Turner , L. J. Abu‐Raddad , S. L. Gottlieb , K. J. Looker , Bull. W. H. O. 2020, 98, 315. 32514197 10.2471/BLT.19.237149 PMC7265941

[2] K. J. Looker , C. Johnston , N. J. Welton , C. James , P. Vickerman , K. M. E. Turner , M. C. Boily , S. L. Gottlieb , BMJ Global Health 2020, 5, 1875. 10.1136/bmjgh-2019-001875 PMC7061890 32201620

[3] C. L. Poole , S. H. James , Clin. Ther. 2018, 40, 1282. 30104016 10.1016/j.clinthera.2018.07.006 PMC7728158

[4] J. Deval , Drugs 2009, 69, 151. 19228073 10.2165/00003495-200969020-00002

[5] J. Luczkowiak , M. Álvarez , A. Sebastián‐Martín , L. Menéndez‐Arias , in Viral Polymerases: Structures, Functions and Roles as Antiviral Drug Targets, (Ed: S. P. Gupta ), Elsevier‐Academic Press, London, UK 2019, pp. 95–134.

[6] J. Piret , G. Boivin , Enzymes 2021, 50, 79. 34861944 10.1016/bs.enz.2021.07.003

[7] R. Duan , R. D. de Vries , A. D. Osterhaus , L. Remeijer , G. M. Verjans , J. Infect. Dis. 2008, 198, 659. 18627246 10.1086/590668

[8] J. Piret , G. Boivin , Adv. Exp. Med. Biol. 2021, 1322, 1. 34258735 10.1007/978-981-16-0267-2_1

[9] L. E. Pope , J. F. Marcelletti , L. R. Katz , J. Y. Lin , D. H. Katz , M. L. Parish , P. G. Spear , Antiviral Res. 1998, 40, 85. 9864049 10.1016/s0166-3542(98)00048-5

[10] K. K. Biron , R. J. Harvey , S. C. Chamberlain , S. S. Good , A. A. Smith 3rd , M. G. Davis , C. L. Talarico , W. H. Miller , R. Ferris , R. E. Dornsife , S. C. Stanat , J. C. Drach , L. B. Townsend , G. W. Koszalka , Antimicrob. Agents Chemother. 2002, 46, 2365. 12121906 10.1128/AAC.46.8.2365-2372.2002 PMC127361

[11] T. Goldner , G. Hewlett , N. Ettischer , H. Ruebsamen‐Schaeff , H. Zimmermann , P. Lischka , J. Virol. 2011, 85, 10884. 21752907 10.1128/JVI.05265-11 PMC3187482

[12] T. Goldner , G. Hewlett , H. Ruebsamen‐Schaeff , H. Zimmermann , P. Lischka , Antimicrob. Agents Chemother. 2014, 58, 610. 24189264 10.1128/AAC.01794-13 PMC3910730

[13] E. M. Borst , J. Kleine‐Albers , I. Gabaev , M. Babic , K. Wagner , A. Binz , I. Degenhardt , M. Kalesse , S. Jonjic , R. Bauerfeind , M. Messerle , J. Virol. 2013, 87, 1720. 23175377 10.1128/JVI.01955-12 PMC3554196

[14] Y. Yang , P. Yang , N. Wang , Z. Chen , D. Su , Z. H. Zhou , Z. Rao , X. Wang , Protein Cell 2020, 11, 339. 32328903 10.1007/s13238-020-00710-0 PMC7196598

[15] S. Selvajaran Sigamani , H. Zhao , Y. N. Kamau , J. D. Baines , L. Tang , J. Virol. 2013, 87, 7140. 23596306 10.1128/JVI.00311-13 PMC3676077

[16] M. Nadal , P. J. Mas , A. G. Blanco , C. Arnan , M. Solà , D. J. Hart , M. Coll , Proc. Natl. Acad. Sci. USA 2010, 107, 16078. 20805464 10.1073/pnas.1007144107 PMC2941324

[17] S. Bongarzone , M. Nadal , Z. Kaczmarska , C. Machón , M. Álvarez , F. Albericio , M. Coll , ACS Omega 2018, 3, 8497. 31458978 10.1021/acsomega.8b01472 PMC6645139

[18] W. Yang , Q. Rev. Biophys. 2011, 44, 1. 20854710 10.1017/S0033583510000181 PMC6320257

[19] K. A. Majorek , S. Dunin‐Horkawicz , K. Steczkiewicz , A. Muszewska , M. Nowotny , K. Ginalski , J. M. Bujnicki , Nucleic Acids Res. 2014, 42, 4160. 24464998 10.1093/nar/gkt1414 PMC3985635

[20] G. N. Maertens , A. N. Engelman , P. Cherepanov , Nat. Rev. Microbiol. 2022, 20, 20. 34244677 10.1038/s41579-021-00586-9 PMC8671357

[21] S. Olschewski , S. Cusack , M. Rosenthal , Trends Microbiol. 2020, 28, 293. 31948728 10.1016/j.tim.2019.12.006

[22] A. B. Russell , Cell 2020, 181, 1450. 32589954 10.1016/j.cell.2020.05.044

[23] H. Li , Y. Hwang , K. Perry , F. Bushman , G. D. Van Duyne , J. Biol. Chem. 2016, 291, 11094. 27013661 10.1074/jbc.M115.709139 PMC4900259

[24] C. Meck , M. P. D'Erasmo , D. R. Hirsch , R. P. Murelli , MedChemComm 2014, 5, 842. 25089179 10.1039/C4MD00055B PMC4114738

[25] S. R. Budihas , I. Gorshkova , S. Gaidamakov , A. Wamiru , M. K. Bona , M. A. Parniak , R. J. Crouch , J. B. McMahon , J. A. Beutler , S. F. Le Grice , Nucleic Acids Res. 2015, 33, 1249. 10.1093/nar/gki268 PMC552956 15741178

[26] Y. Hu , X. Cheng , F. Cao , A. Huang , J. E. Tavis , Antiviral Res. 2013, 99, 221. 23796982 10.1016/j.antiviral.2013.06.007

[27] N. L. Ponzar , R. Tajwar , N. Pozzi , J. E. Tavis , J. Biol. Chem. 2022, 298, 101790. 35247386 10.1016/j.jbc.2022.101790 PMC9034292

[28] J. E. Tavis , H. Wang , A. E. Tollefson , B. Ying , M. Korom , X. Cheng , F. Cao , K. L. Davis , W. S. Wold , L. A. Morrison , Antimicrob. Agents Chemother. 2014, 58, 7451. 25267681 10.1128/AAC.03875-14 PMC4249532

[29] P. J. Ireland , J. E. Tavis , M. P. D'Erasmo , D. R. Hirsch , R. P. Murelli , M. M. Cadiz , B. S. Patel , A. K. Gupta , T. C. Edwards , M. Korom , E. A. Moran , L. A. Morrison , Antimicrob. Agents Chemother. 2016, 60, 2140. 26787704 10.1128/AAC.02675-15 PMC4808205

[30] K. A. DiScipio , S. Weerasooriya , R. Szczepaniak , A. Hazeen , L. R. Wright , D. L. Wright , S. K. Weller , mBio 2022, 13, 0322621. 10.1128/mbio.03226-21 PMC8787488 35073739

[31] A. Gazquez Casals , A. J. Berkowitz , A. J. Yu , H. E. Waters , D. V. Schiavone , D. M. Kapkayeva , L. A. Morrison , R. P. Murelli , RSC Adv. 2023, 13, 8743. 36936842 10.1039/d2ra06749h PMC10016935

[32] T. Masaoka , H. Zhao , D. R. Hirsch , M. P. D'Erasmo , C. Meck , B. Varnado , A. Gupta , M. J. Meyers , J. Baines , J. A. Beutler , R. P. Murelli , L. Tang , S. F. Le Grice , Biochemistry 2016, 55, 809. 26829613 10.1021/acs.biochem.5b01254 PMC4819167

[33] Z. Yan , K. F. Bryant , S. M. Gregory , M. Angelova , D. H. Dreyfus , X. Z. Zhao , D. M. Coen , T. R. Burke Jr , D. M. Knipe , mBio 2014, 5, e01318. 24987091 10.1128/mBio.01318-14 PMC4161245

[34] M. R. Pennington , I. E. H. Voorhees , H. M. Callaway , S. D. Dehghanpir , J. D. Baines , C. R. Parrish , G. R. Van de Walle , J. Virol. 2018, 92, e00994. 30045987 10.1128/JVI.00994-18 PMC6158441

[35] J. T. Miller , H. Zhao , T. Masaoka , B. Varnado , E. M. Cornejo Castro , V. A. Marshall , K. Kouhestani , A. Y. Lynn , K. E. Aron , A. Xia , J. A. Beutler , D. R. Hirsch , L. Tang , D. Whitby , R. P. Murelli , S. F. J. Le Grice , Antimicrob. Agents Chemother. 2018, 62, e00233. 30061278 10.1128/AAC.00233-18 PMC6153795

[36] A. D. Jagtap , R. J. Geraghty , Z. Wang , J. Med. Chem. 2023, 66, 13874. 37827528 10.1021/acs.jmedchem.3c01280 PMC11793932

[37] A. Cilibrizzi , V. Abbate , Y. L. Chen , Y. Ma , T. Zhou , R. C. Hider , Chem. Rev. 2018, 118, 7657. 30033720 10.1021/acs.chemrev.8b00254

[38] K. Morimoto , M. Kawai , T. Akiyama , H. Mikamiyama , Y. Taoda , K. Tomita , N. Suzuki , K. Takaya , M. Mikamiyama , C. Kageyama , WO2010147068A1, 2010.

[39] M. Kawai , T. Akiyama , H. Mikamiyama , Y. Taoda , K. Tomita , N. Suzuki , K. Anan , C. Kageyama , M. Miyagawa , WO2012039414A1, 2012.

[40] R. O'Hanlon , M. L. Shaw , Curr. Opin. Virol. 2019, 35, 14. 30852344 10.1016/j.coviro.2019.01.006

[41] E. J. Mifsud , F. G. Hayden , A. C. Hurt , Antiviral Res. 2019, 169, 104545. 31247246 10.1016/j.antiviral.2019.104545

[42] E. Takashita , Cold Spring Harb. Perspect. Med. 2021, 11, a038687. 32122918 10.1101/cshperspect.a038687 PMC8091960

[43] P. Desai , S. Person , J. Virol. 1998, 72, 7563. 9696854 10.1128/jvi.72.9.7563-7568.1998 PMC110002

[44] X. Sun , S. Vilar , N. P. Tatonetti , Sci. Transl. Med. 2013, 5, 205rv1. 10.1126/scitranslmed.3006667 24089409

[45] T. C. Chou , Cancer Res. 2010, 70, 440. 20068163 10.1158/0008-5472.CAN-09-1947

[46] M. Kawai , K. Tomita , T. Akiyama , A. Okano , M. Miyagawa , Jpn. Tokkyo Koho JP5971830, 2016.

[47] T. Shishido , T. Noshi , A. Yamamoto , M. Kitano , PCT Int. Appl. 201803246, 2017.

[48] H. Ju , J. Zhang , B. Huang , D. Kang , B. Huang , X. Liu , P. Zhan , J. Med. Chem. 2017, 60, 3533. 28118010 10.1021/acs.jmedchem.6b01227

[49] T. Noshi , M. Kitano , K. Taniguchi , A. Yamamoto , S. Omoto , K. Baba , T. Hashimoto , K. Ishida , Y. Kushima , K. Hattori , M. Kawai , R. Yoshida , M. Kobayashi , T. Yoshinaga , A. Sato , M. Okamatsu , Y. Sakoda , H. Kida , T. Shishido , A. Naito , Antiviral Res. 2018, 160, 109. 30316915 10.1016/j.antiviral.2018.10.008

[50] E. Takashita , H. Morita , R. Ogawa , K. Nakamura , S. Fujisaki , M. Shirakura , T. Kuwahara , N. Kishida , S. Watanabe , T. Odagiri , Front. Microbiol. 2018, 9, 3026. 30574137 10.3389/fmicb.2018.03026 PMC6291754

[51] V. P. Mishin , M. C. Patel , A. Chesnokov , J. De La Cruz , H. T. Nguyen , L. Lollis , E. Hodges , Y. Jang , J. Barnes , T. Uyeki , C. T. Davis , D. E. Wentworth , L. V. Gubareva , Emerging Infect. Dis. 2019, 25, 1969. 10.3201/eid2510.190607 PMC6759234 31287050

[52] S. Ter Horst , Y. Fernandez‐Garcia , M. Bassetto , S. Günther , A. Brancale , J. Neyts , J. Rocha‐Pereira , J. Antimicrob. Chemother. 2020, 75, 3189. 32766680 10.1093/jac/dkaa337

[53] S. Toba , A. Sato , M. Kawai , Y. Taoda , Y. Unoh , S. Kusakabe , H. Nobori , S. Uehara , K. Uemura , K. Taniguchi , M. Kobayashi , T. Noshi , R. Yoshida , A. Naito , T. Shishido , J. Maruyama , S. Paessler , M. J. Carr , W. W. Hall , K. Yoshimatsu , J. Arikawa , K. Matsuno , Y. Sakoda , M. Sasaki , Y. Orba , H. Sawa , H. Kida , Proc. Natl. Acad. Sci. USA 2022, 119, e2206104119. 36037386 10.1073/pnas.2206104119 PMC9457168

[54] K. Liu , L. Li , Y. Liu , X. Wang , J. Liu , J. Li , F. Deng , R. Zhang , Y. Zhou , Z. Hu , W. Zhong , M. Wang , C. Guo , Antiviral Res. 2024, 227, 105890. 38657838 10.1016/j.antiviral.2024.105890

[55] A. Dias , D. Bouvier , T. Crépin , A. A. McCarthy , D. J. Hart , F. Baudin , S. Cusack , R. W. Ruigrok , Nature 2009, 458, 914. 19194459 10.1038/nature07745

[56] P. Yuan , M. Bartlam , Z. Lou , S. Chen , J. Zhou , X. He , Z. Lv , R. Ge , X. Li , T. Deng , E. Fodor , Z. Rao , Y. Liu , Nature 2009, 458, 909. 19194458 10.1038/nature07720

[57] S. Omoto , V. Speranzini , T. Hashimoto , T. Noshi , H. Yamaguchi , M. Kawai , K. Kawaguchi , T. Uehara , T. Shishido , A. Naito , S. Cusack , Sci. Rep. 2018, 8, 9633. 29941893 10.1038/s41598-018-27890-4 PMC6018108

[58] B. Todd , E. P. Tchesnokov , M. Götte , J. Biol. Chem. 2021, 296, 100486. 33647314 10.1016/j.jbc.2021.100486 PMC8065212

[59] Y. Song , W. Chen , D. Kang , Q. Zhang , P. Zhan , X. Liu , Comb. Chem. High Throughput Screening 2014, 17, 536. 10.2174/1386207317666140122101631 24446784

[60] L. M. Grady , P. Szczepaniak , R. P. Murelli , T. Masaoka , S. F. J. Le Grice , D. L. Wright , S. K. Weller , J. Virol. 2017, 91, e01380 28956767 10.1128/JVI.01380-17 PMC5686714

[61] E. M. Schilling , M. Scherer , F. Rothemund , T. Stamminger , PLoS Pathog. 2021, 17, e1009460. 33770148 10.1371/journal.ppat.1009460 PMC8026080

[62] L. Lerma , A. L. Muñoz , R. García Utrilla , B. Sainz , F. Lim , E. Tabarés , S. Gómez‐Sebastián , Virus Res. 2020, 279, 197896. 32045631 10.1016/j.virusres.2020.197896

[63] L. E. Post , S. Mackem , B. Roizman , Cell 1981, 24, 555. 6263501 10.1016/0092-8674(81)90346-9

[64] Y. Liu , X. Guan , C. Li , F. Ni , S. Luo , J. Wang , D. Zhang , M. Zhang , Q. Hu , Virology 2018, 525, 83. 30248525 10.1016/j.virol.2018.09.004

[65] L. Holm , Nucleic Acids Res. 2022, 50, W210. 35610055 10.1093/nar/gkac387 PMC9252788

[66] J. Jumper , R. Evans , A. Pritzel , T. Green , M. Figurnov , O. Ronneberger , K. Tunyasuvunakool , R. Bates , A. Žídek , A. Potapenko , A. Bridgland , C. Meyer , S. A. A. Kohl , A. J. Ballard , A. Cowie , B. Romera‐Paredes , S. Nikolov , R. Jain , J. Adler , T. Back , S. Petersen , D. Reiman , E. Clancy , M. Zielinski , M. Steinegger , M. Pacholska , T. Berghammer , S. Bodenstein , D. Silver , O. Vinyals , et al., Nature 2021, 596, 583. 34265844 10.1038/s41586-021-03819-2 PMC8371605

[67] J. R. López‐Blanco , A. J. Canosa‐Valls , Y. Li , P. Chacón , Nucleic Acids Res. 2016, 44, W395 . 27151199 10.1093/nar/gkw395 PMC4987936

[68] X. Zhou , K. Yu , J. Liu , Z. Jin , X. Hu , Crystals 2022, 12, 550.

[69] J. Wang , P. Cieplak , P. Kollman , J. Comput. Chem. 2000, 21, 1049.

[70] G. Scalmani , M. J. Frisch , J. Chem. Phys. 2010, 132, 114110. 20331284 10.1063/1.3359469

[71] M. J. Frisch , G. W. Trucks , G. E. Schlegel , M. A. R. Scuseria , J. R. Cheeseman , G. Scalmani , V. Barone , G. A. Petersson , H. Nakatsuji , X. Li , M. Caricato , A. Marenich , J. Bloino , B. G. Janesko , R. Gomperts , B. Mennucci , H. P. Hratchian , J. V. Ortiz , A. F. Izmaylov , J. L. Sonnenberg , D. Williams‐Young , F. Ding , F. Lipparini , F. Egidi , J. Goings , B. Peng , A. Petrone , T. Henderson , D. Ranasinghe , V. G. Zakrzewski , et al., Gaussian, Inc., Wallingford CT 2016, https://gaussian.com/g09citation/.

[72] D. M. Himmel , K. A. Maegley , T. A. Pauly , J. D. Bauman , K. Das , C. Dharia , A. D. Clark Jr. , K. Ryan , M. J. Hickey , R. A. Love , S. H. Hughes , S. Bergqvist , E. Arnold , Structure 2009, 17, 1625. 20004166 10.1016/j.str.2009.09.016 PMC3365588

[73] H. Zhao , Z. Lin , A. Y. Lynn , B. Varnado , J. A. Beutler , R. P. Murelli , S. F. Le Grice , L. Tang , Nucleic Acids Res. 2015, 43, 11003. 26450964 10.1093/nar/gkv1018 PMC4678813

[74] A. J. Kohlbrand , R. W. Stokes , B. Sankaran , S. M. Cohen , Biochemistry 2024, 63, 264. 38190441 10.1021/acs.biochem.3c00536 PMC10851415

