# Supplementary material for: Discovery of a Baloxavir‐Inspired Endonuclease Inhibitor That Prevents Herpes Simplex Virus 1 Replication in Cell Culture and In Vivo
Source: Adv Sci (Weinh). 2025 Sep 15;12(42):e08006. doi: 10.1002/advs.202508006 (PMC12622528; doi:10.1002/advs.202508006)
Supplement: Supplementary file 1 — Supporting Information [file ADVS-12-e08006-s001.pdf]

Supplementary Information for

Discovery of a Baloxavir-Inspired Endonuclease Inhibitor that Prevents Herpes Simplex Virus 1 Replication in Cell Culture and *In Vivo*

Sabina Andreu,[1,2]† Kai Tang[3]†, Jiahui Zhou[3]†, Gabriel Pino-Peco,[1] Nerea López-Carrobles,[1] Raquel Bello-Morales,[2] Daniel Galdo-Torres,[2] Lina Zhang,[3] Federico Gago,[4] José Antonio López-Guerrero,[1,2]* Xinyong Liu,[3]* Peng Zhan[3]* & Luis Menéndez-Arias[1]*

[1] *Centro de Biología Molecular "Severo Ochoa" (Consejo Superior de Investigaciones Científicas and Universidad Autónoma de Madrid), c/ Nicolás Cabrera 1, Campus de Cantoblanco-UAM, 28049 Madrid, Spain*

[2] *Department of Molecular Biology, Universidad Autónoma de Madrid, c/ Darwin, 2 Cantoblanco, 28049 Madrid, Spain.*

[3] *Department of Medicinal Chemistry, Key Laboratory of Chemical Biology, Ministry of Education, School of Pharmaceutical Sciences, Cheeloo College of Medicine, Shandong University, Jinan, 250012 P.R. China*

[4] *Department of Biomedical Sciences, Universidad de Alcalá, Alcalá de Henares, 28805 Madrid, Spain.*

† Joint first authorship

* *Correspondence to Luis Menéndez-Arias*: Centro de Biología Molecular "Severo Ochoa" (Consejo Superior de Investigaciones Científicas and Universidad Autónoma de Madrid), c/ Nicolás Cabrera 1, Campus de Cantoblanco-UAM, 28049 Madrid, Spain; E-mail: lmenendez@cbm.csic.es

E-mails of co-corresponding authors: zhanpeng1982@sdu.edu.cn (Zhan P.); xinyongl@sdu.edu.cn (Liu X.); ja.lopez@uam.es (López-Guerrero J.A.)

This PDF file includes:

Figs. S1 to S11
Table S1
Supplementary Methods
References

Other Supplementary Materials for this manuscript include the following:

Data S1 to S2

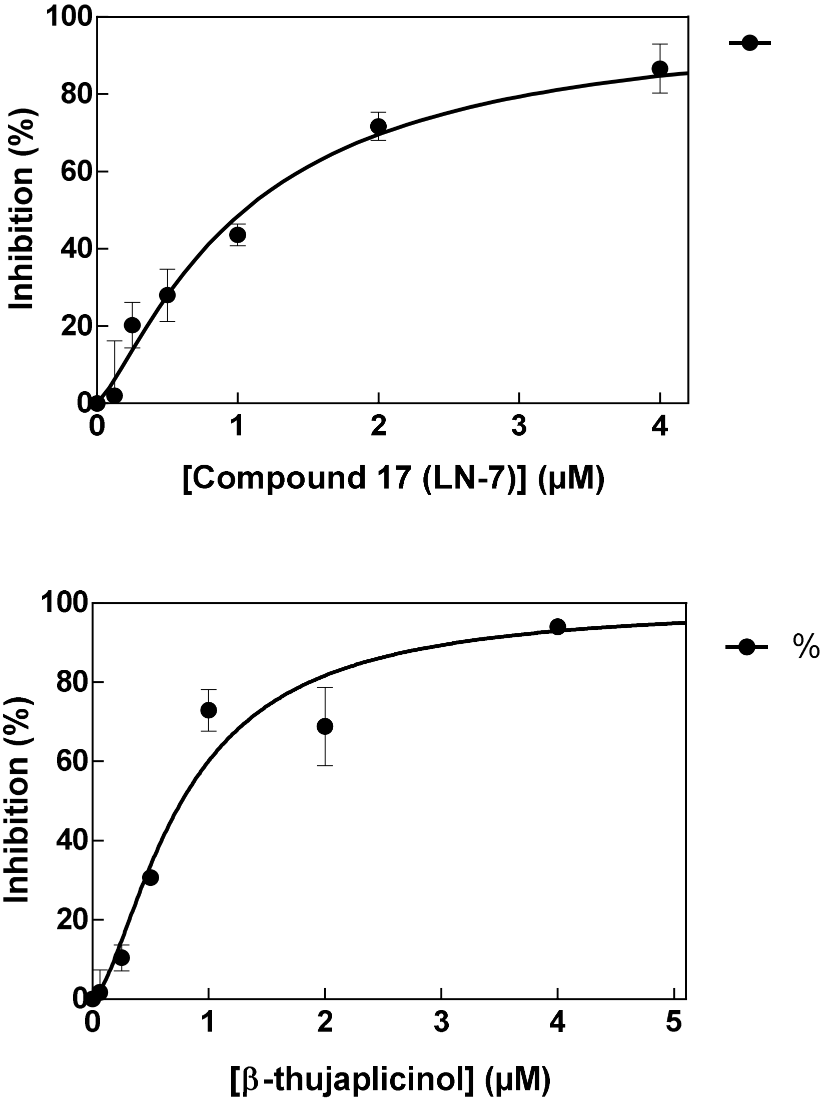

Supplementary Figure S1. Inhibition of HSV-1 pUL15C nuclease activity by compound 17 (LN-7) and β-thujaplicinol. Representative inhibition plots are shown for both compounds tested at concentrations ranging from 0.25 to 16 μM. The data represent the mean and standard deviation of three independent measurements.

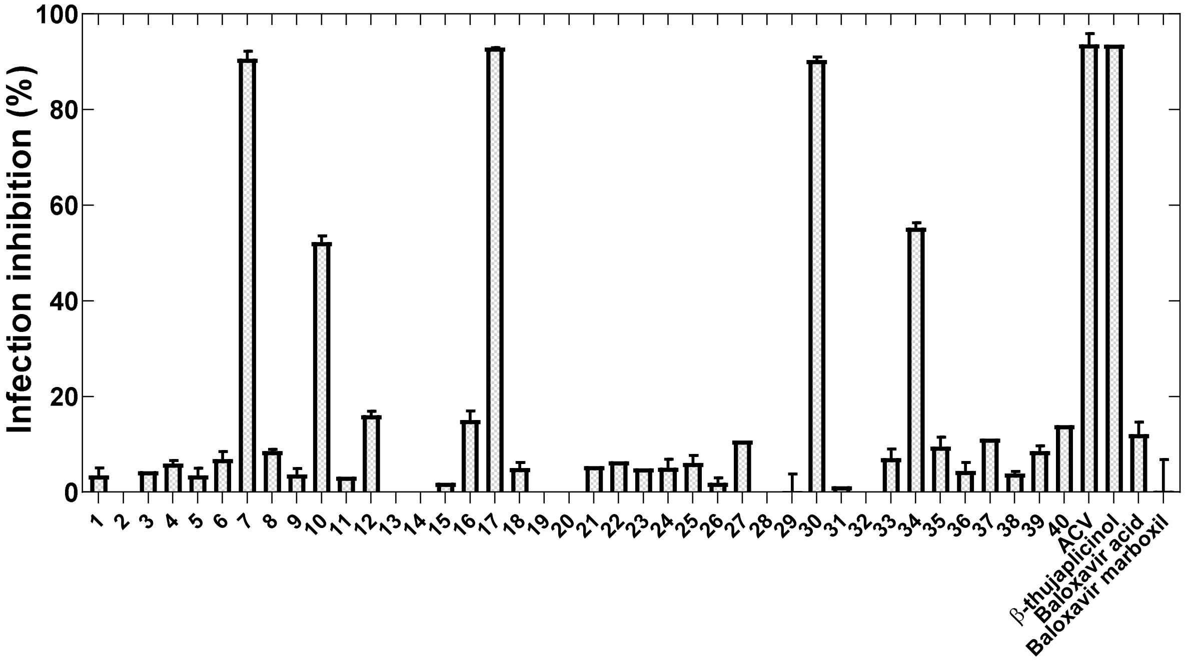

Supplementary Figure S2. Screening of antiviral compounds. Bars represent percentage inhibition obtained for each compound at a concentration of 10 μM in Vero cells infected with HSV-1 K26-GFP. After 24 h, the percentage of GFP positive cells was determined by flow cytometry and compared with the values obtained in the absence of inhibitor. The data represent the mean and standard deviation of three independent experiments.

A

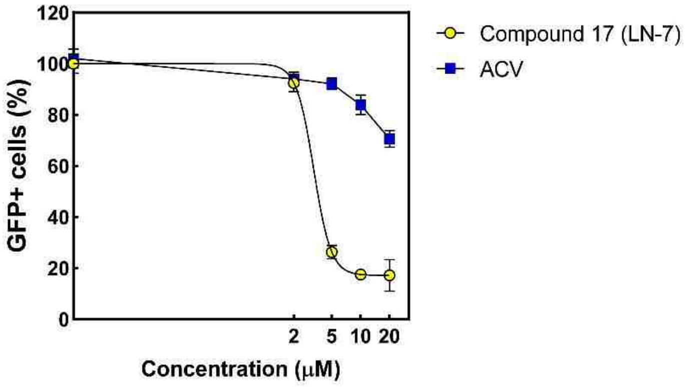

B

Compound	EC_{50} (μM)
**17 (LN-7)**	3.2 ± 0.3
**ACV**	>20 (31.4 ± 3.3)

C

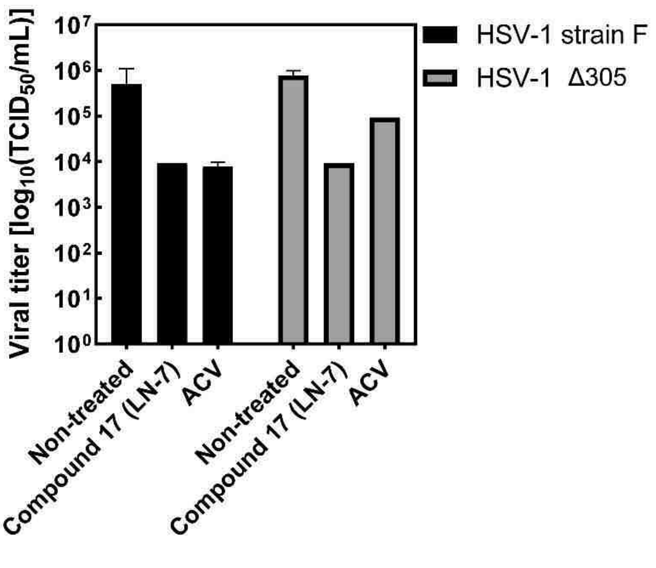

Supplementary Figure S3. Antiviral activity of Compound 17 (LN-7) against HSV-1 ACV-resistant strains. (**A**) EC_{50} values of LN-7 and ACV against F-TKGF. Dose-response curves for EC_{50} values were determined by a nonlinear fit model with variable response curve (four parameters). HSV-1 K26-GFP (ACV-sensitive) was used as control. Experiments were performed in triplicate for each data point (n = 3), and values are represented as mean percentages of infection/viability ± S.E.M. For some conditions, S.E.M. values are so small that they are not visible on the plot. (**B**) Inhibitory effects of ACV and LN-7 on F-TKGF infection in Vero cells. Numbers in parentheses indicate percentage inhibition at the highest concentration tested (20 μM). (**C**) Antiviral activity of LN-7 and ACV against HSV-1(F)Δ305 (ACV-resistant). Compounds were tested at 5 μM concentration. HSV-1 strain F (ACV-sensitive) was used as control. Progeny virus was titrated in Vero cells at 24 h post-infection to determine the 50% tissue culture infective dose per mL ($TCID_{50}$/mL) (n=2).

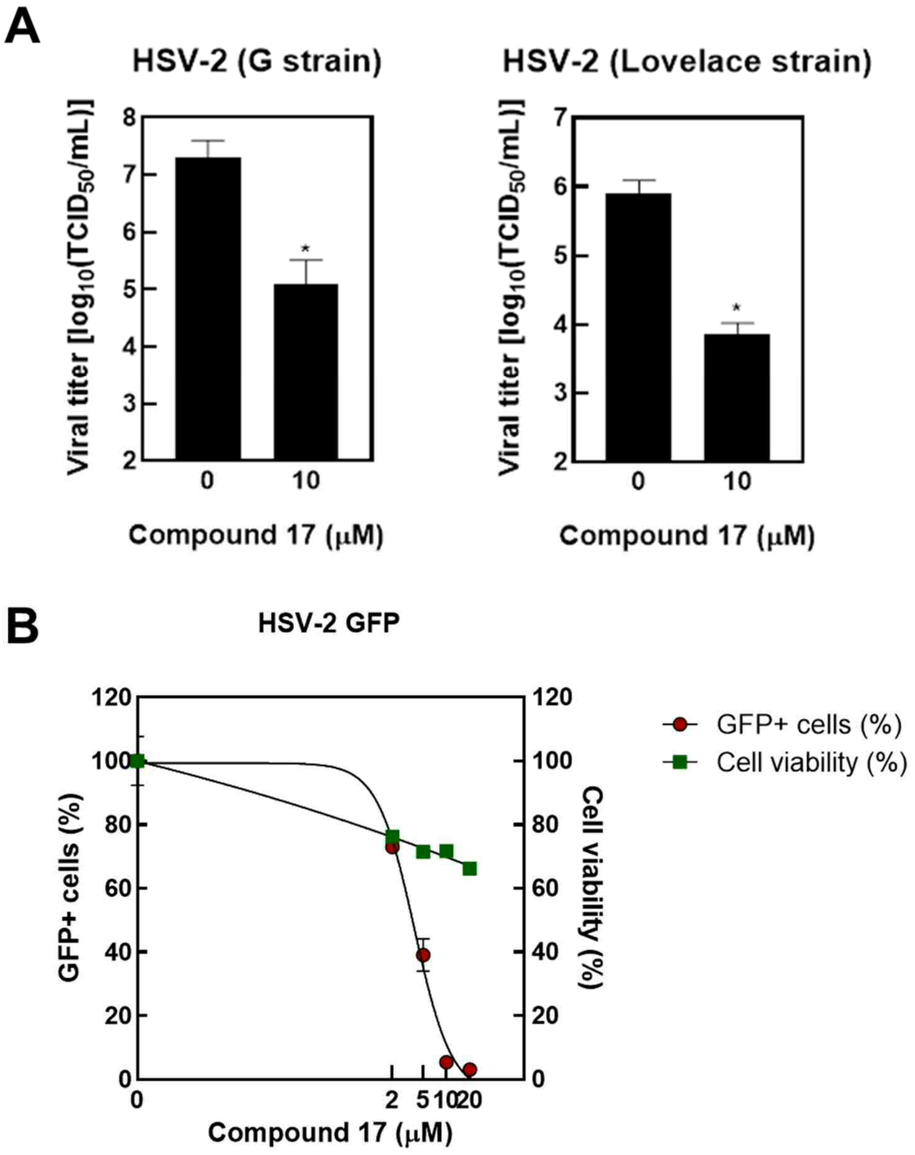

Supplementary Figure S4. Antiviral activity of compound 17 (LN-7) against HSV-2 strains G and Lovelace. (**A**) Antiviral activity against HSV-2 strains G and Lovelace at 10 μM concentration of inhibitor. Progeny virus was titrated in Vero cells at 24 h post-infection to determine the 50% tissue culture infective dose per mL (TCID50/mL) (n=3). (**B**) Antiviral activity against HSV-2 GFP. Dose-response curves for EC_{50} and CC_{50} values were determined by a non-linear fit model with variable response curve (four parameters). The percentages of GFP positive cells (infection) and cell viabilities compared to untreated cultures are represented by red dots and green squares, respectively. Experiments were performed in triplicate for each data point (n = 3), and values are represented as mean percentages of infection/viability ± S.E.M. For some conditions, S.E.M. values are so small that they are not visible on the plot.

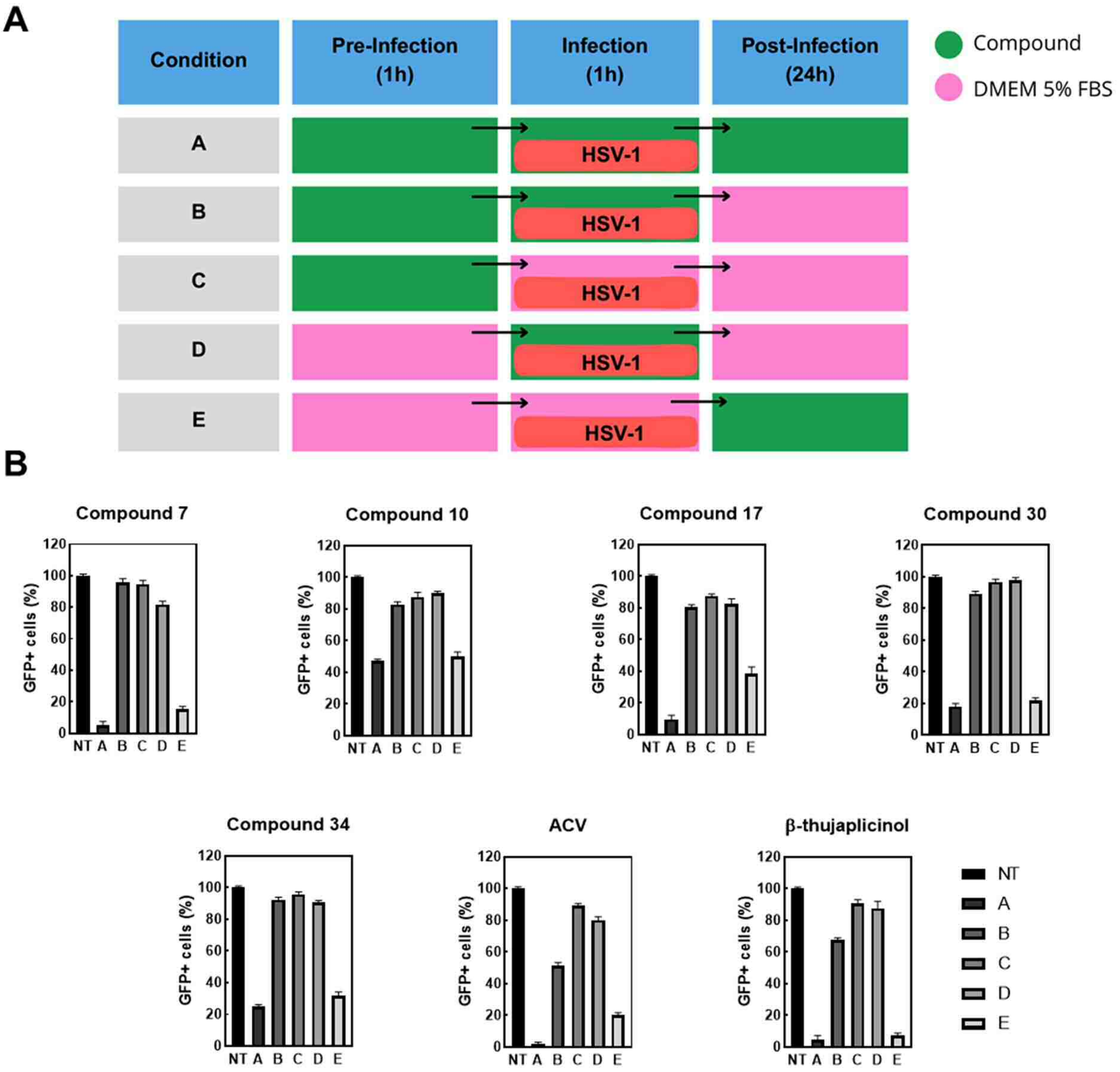

Supplementary Figure S5. Time-of-addition experiments. Vero cell cultures were grown in 48-well culture plates and inoculated at a m.o.i. of 0.1 with HSV-1 K26-GFP, for 1 h in the presence or absence of compounds at 37°C. (**A**) Schematic diagrams showing the times where the com-pound was present in the assay. Pink bars are used to indicate that cells were exposed to culture medium without inhibitor. (**B**) Percentage of infection (GFP positive cells) normalized to the values obtained with untreated cells, for each protocol and derivatives with antiviral activity, as well as acyclovir and β-thujaplicinol as reference compounds. Triplicate experiments were performed for each data point (n = 4), and the value is presented as mean of the percentage of normalized infection ± S.E.M. **p* < 0.05 and ***p* < 0.0001 were considered significant as determined using the two-tailed Student's *t*-test.

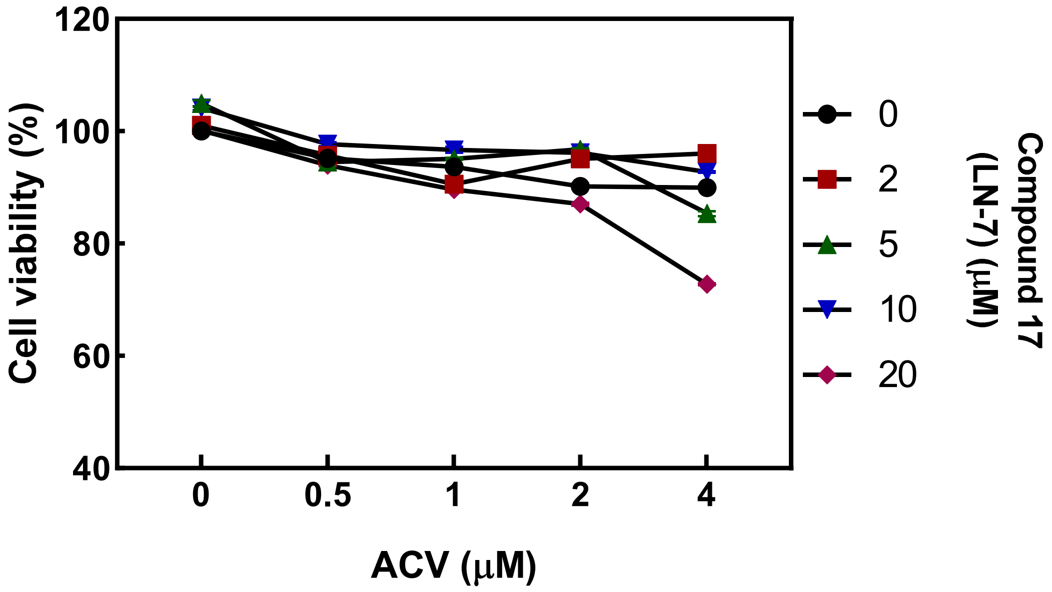

Supplementary Figure S6. Cytotoxic effects of Compound 17 (LN-7) and acyclovir (ACV) added in combination to Vero cell cultures. Cells were treated with Compound 17 and ACV for 24 h. Cell viability was measured using the MTT tetrazolium salt assay and calculated as the percentage of cell viability compared to that obtained in untreated cells. Represented values are averages ± S.D. (n = 4).

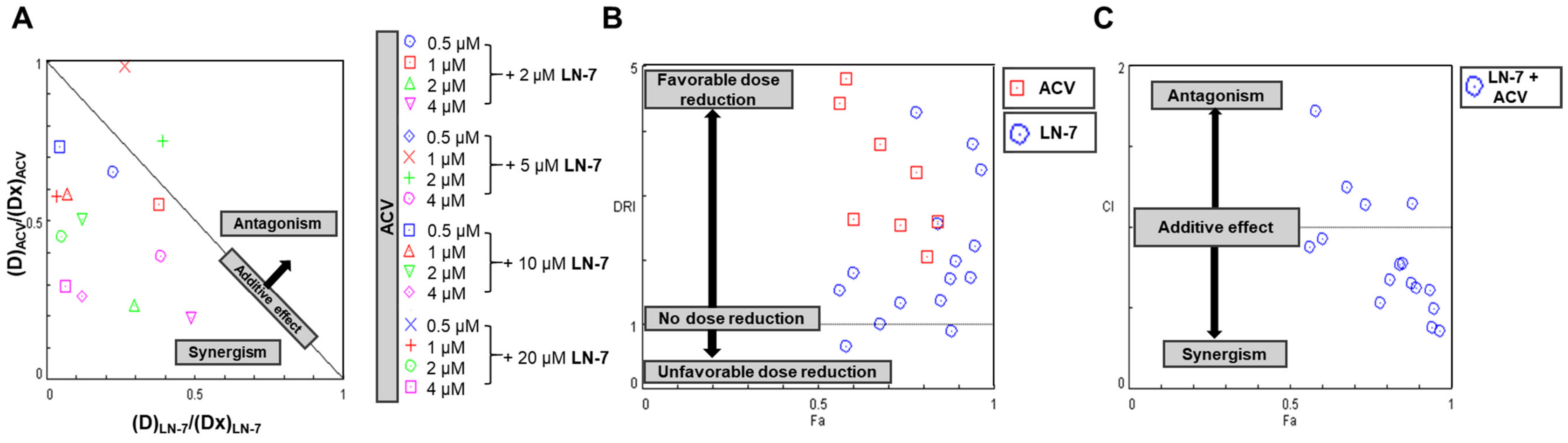

Supplementary Fig. S7. Synergistic activity of acyclovir (ACV) and compound 17 (LN-7) against HSV-1. (A) Dose-normalized isobologram for the non-constant ratio combinations. Data points on the hypotenuse indicate an additive effect, those on the lower left indicate synergism, and those on the upper right indicate antagonism. D, dose; Dx, dose of a single drug that exhibits a given percent inhibition. **(B)** Dose reduction index (DRI) for each drug for a given effect (Fa). DRI of >1, 1, and <1 indicate a favorable dose reduction, no dose reduction, and a negative dose reduction, respectively. Values larger than 5 are not represented in the plot. **(C)** Combination index (CI) values as a function of the effect levels (Fa), where CI values of <1, 1, and >1 indicate synergism, additive effect, and antagonism, respectively. More specifically, robust and moderate synergism are characterized by CI values in the range of 0.3 – 0.7 and 0.7 – 0.95, respectively. Graphs were generated through analysis using CompuSyn software, and the specific procedures are detailed in the Materials and Methods section of the paper.

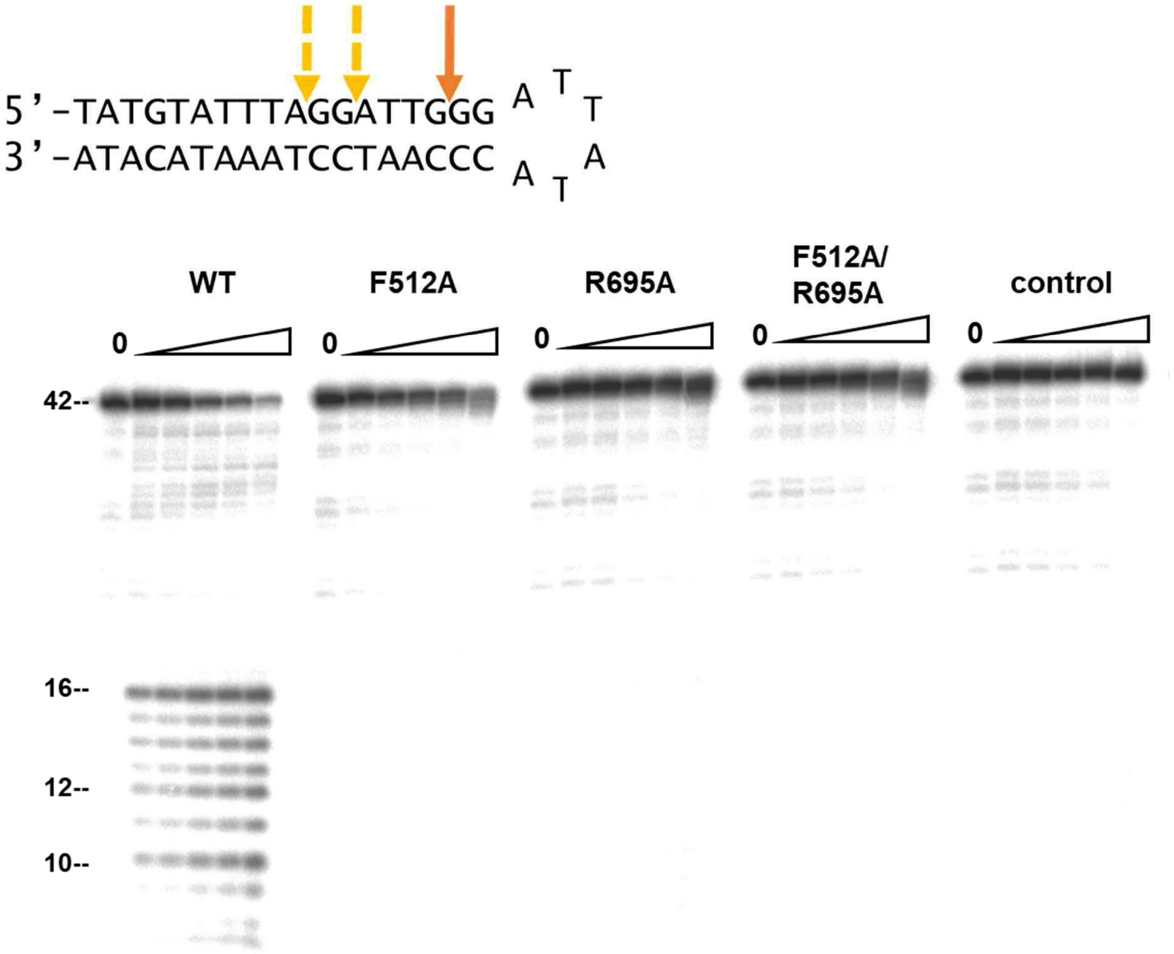

Supplementary Figure S8. DNA cleavage kinetics of a ^{32}P-labeled hairpin oligonucleotide by WT and pUL15C variants F512A, R695A and the double-mutant F512A/R695A. Cleavage reactions were carried out for 1, 2, 5, 10 and 30 min at 37ºC in the presence of 100 nM oligonucleotide and the corresponding enzymes at a concentration of 900 nM. Control reactions were carried out in the absence of enzyme. The nucleotide sequence of the substrate is shown above with major cleavage sites indicated with red and orange arrows. A representative gel is shown.

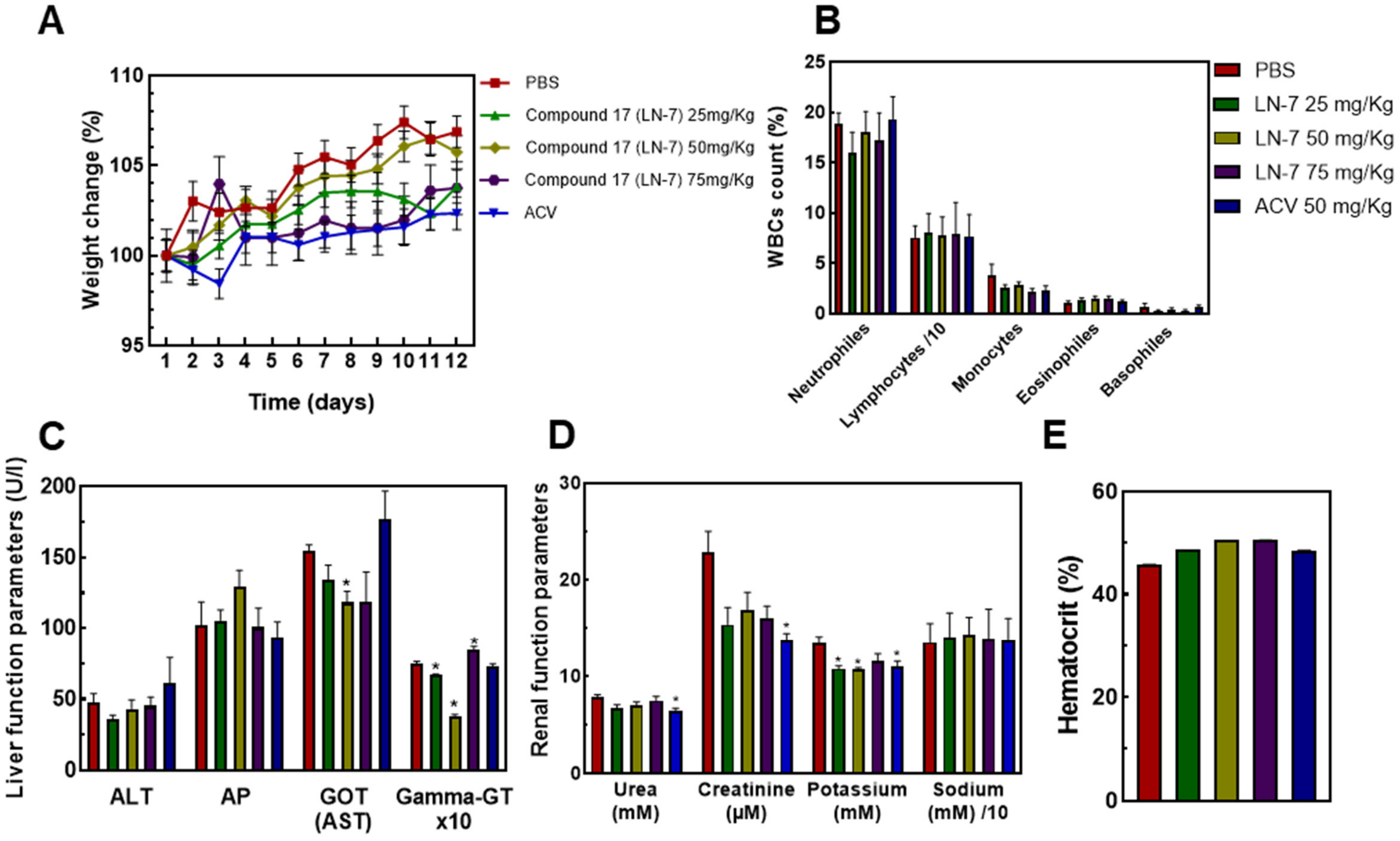

Supplementary Fig. S9. LN-7 (compound 17) shows no toxic effects in an *in vivo* mouse model. Thirty BALB/c mice were distributed in five different groups (n = 6) and were mock-inoculated (PBS) or inoculated (LN-7 at 25, 50, and 75 mg/Kg, or 50 mg/mL ACV) by oral administration for 5 consecutive days. On day 12, mice were sacrificed and whole blood was obtained by cardiac puncture. **(A)** Percentage of body weight change, **(B)** WBC count, **(C)** liver markers alanine transaminase (ALT), alkaline phosphatase (AP), aspartate aminotransferase (AST), and γ-glutamyl transferase (Gamma-GT), and **(D)** renal markers urea, creatinine, potassium, and sodium levels were analyzed. Hematocrit levels are shown in panel **(E)** for the five groups of mice. Mean (n = 6) ± S.E.M are shown. A two-way ANOVA followed by Tukey's post-hoc test comparisons were used to analyze data and identify significant differences between the means of different groups (*$p < 0.05$).

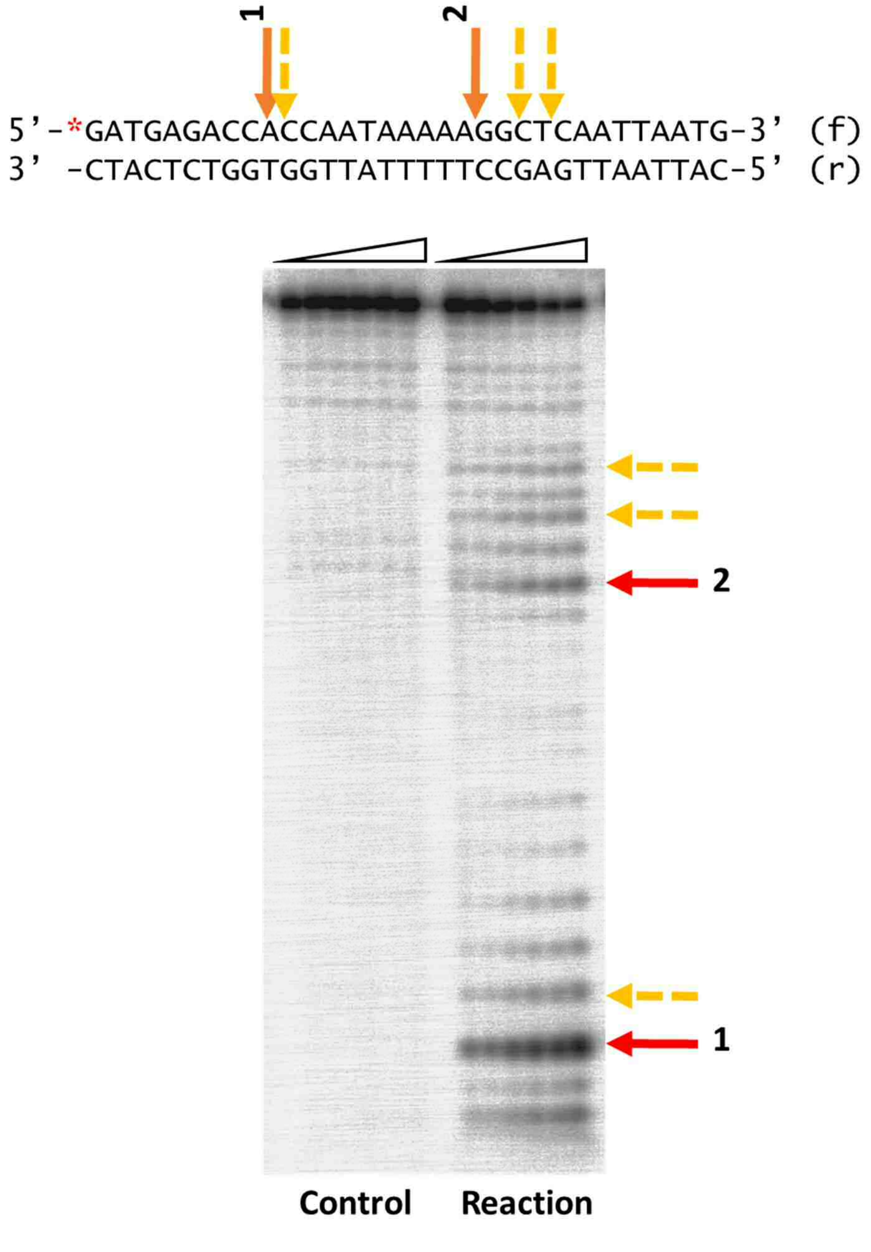

Supplementary Figure S10. pUL15C cleavage of the 33-base pair DNA duplex used in nuclease inhibition assays. Asterisk (red) indicates the position of the ^{32}P label. Cleavage reactions were carried out for 1, 2, 5, 10, 20 and 30 min at 37ºC in the presence of 100 nM duplex DNA with or without pUL15C (at 600 nM). Red arrows indicate major cleavage sites and orange arrows indicate secondary cleavages.

```
4IOX  467  GSHMTGDDRPVLTKSAGERFLLYRPST-TTNS-GLMAPDLYVYVDPAFTANTRASGTGVA  524
           G++    +  ++T  + E F + R ST  TN+       LYVY+DPAFT N +ASGTGVA
6EY7  419  GTNKISQNTVLITDQSREEFDILRYSTLNTNAYDYFGKTLYVYLDPAFTTNRKASGTGVA  478

4IOX  525  VVGRYRDDYIIFALEHFFLRALTGSAPADIARCVVHSLTQVLALHPGAFRGVRVAVEGNS  584
            VG YR  ++I+ LEHFFLR L+ S+   IA C  H +  VL+LHP     +R+AVEGN+
6EY7  479  AVGAYRHQFLIYGLEHFFLRDLSESSEVAIAECAAHMIISVLSLHP-YLDELRIAVEGNT  537

4IOX  585  SQDSAVAIATHVHTEMHRLLASEGADAGSGPELLFYHCEPPGSAVLYPFFLLNKQKTPAF  644
           +Q +AV IA          L  +   + +   +LFYH  P  + +  PF+L+ + K  A
6EY7  538  NQAAAVRIAC---------LIRQSVQSSTLIRVLFYHT-PDQNHIEQPFYLMGRDKALAV  587

4IOX  645  EHFIKKFNSGGVMASQEIVSATVRLQTDPVEYLLEQLNNLTE-TVSPNTDVRTYSGKRNG  703
           E FI +FNSG + ASQE+VS T++L  DP+EYLLEQ+ NL   T++  T  R  + ++N
6EY7  588  EQFISRFNSGYIKASQELVSYTIKLSHDPIEYLLEQIQNLHRVTLAEGTTARYSAKRQNR  647

4IOX  704  ASDDLMVAVIMAIYLAAQAGPPHTFAPITRVS  (D509, E581, D706, D707)     HSV-1
            SDDL++AVIMA YL
6EY7  648  ISDDLIIAVIMATYLCDDIHAIRF-----RVS  (D463, E534, D650, D651)     HCMV
```

Supplementary Figure S11. Structure-based sequence alignment of C-terminal nuclease domains of the large subunits of HSV-1 and HCMV terminase complexes, pUL15 (PDB code 4IOX) and UL89 (PDB code 6EY7), respectively. Catalytic residues are highlighted in yellow.

Supplementary Table S1. Synergy parameters generated by CompuSyn software for LN-7 (compound 17) and ACV drug interactions against infection of HSV-1 K26 GFP in Vero cells. Synergy is determined by the combination index value (CI), where CI < 1 indicates synergism, CI > 1 indicates antagonism, and CI = 1 indicates that both drugs have an additive effect. More specifically, robust and moderate synergism are characterized by CI values in the range of 0.3 – 0.7 and 0.7 – 0.95, respectively. Fa values (0 to 1) correspond to 0 to 100% of viral infection inhibition.

Dose LN-7 (μM)	Dose ACV (μM)	Effect (Fa)	CI value	Interaction
2	0.5	0.561	0.87875	Moderate synergism
	1	0.601	0.93184	Moderate synergism
	2	0.781	0.53090	Robust synergism
	4	0.8115	0.67871	Robust synergism
5	0.5	0.58	1.71754	Antagonism
	1	0.678	1.25220	Antagonism
	2	0.7645	1.14393	Antagonism
	4	0.84198	0.77526	Moderate synergism
10	0.5	0.85	0.77783	Moderate synergism
	1	0.8767	0.65412	Robust synergism
	2	0.8916	0.62477	Robust synergism
	4	0.9402	0.38283	Robust synergism
20	0.5	0.90177	0.93006	Moderate synergism
	1	0.9346	0.61266	Robust synergism
	2	0.94848	0.4999	Robust synergism
	4	0.96565	0.35772	Robust synergism

SUPPLEMENTARY METHODS

Compound Synthesis and Characterization. All reagents and solvents employed were gained from standard suppliers and directly used without further purification. Liquid chromatography-mass spectrometry (LC-MS) and thin-layer chromatography (TLC) were used to monitor the reactions. Purification of intermediates and final products was carried out by silica gel column chromatography (200-300 mesh). The compounds' melting points were determined by WRS-1A micromelting apparatus and these data were uncorrected. All the ^{1}H and ^{13}C NMR spectra were recorded with Bruker AVANCE III 400 M spectrometer. Chemical shifts were reported in ppm relative to tetramethylsilane (TMS).

General procedure for the synthesis of intermediate 1b. (*R*)-7-(benzyloxy)-3,4,12,12a-tetrahydro-1*H*-[1,4]oxazino[3,4-*c*]pyrido[2,1-*f*][1,2,4]triazine-6,8-dione **1a** (0.5 g, 1.53 mmol, 1 eq) and triethylamine (TEA, 232 mg, 2.30 mmol, 1.5 eq) were added to dichloromethane (DCM, 25 mL), stirred for 10 minutes, then benzoyl chloride (325 mg, 1.84 mmol, 1.2 eq)was added dropwise in an ice bath, and the reaction was carried out at room temperature. After 3 hours, the TLC detection reaction was completed. The solvent was concentrated under reduced pressure, added to water, and extracted in ethyl acetate. The organic phase was combined and dried with anhydrous sodium sulfate. The solvent was concentrated after suction filtration. Finally, the concentrated residue was purified by flash column chromatography (DCM/MeOH = 20:1) to give the corresponding intermediate **1b**. Brown solid, yield 77.5%. ESI-MS: m/z 432.88 $[M+1]^{+}$. $C_{24}H_{21}N_3O_5$ (431.15).

General procedure for the synthesis of intermediates 2b-12b. A mixture of **1a** (0.5 g, 1.53 mmol, 1 eq.), Iodomethane (1.836 mmol, 1.2 eq.), and Cs_2CO_3 (0.99 g, 3.06 mmol, 2 eq.) in DMF (25 mL) was stirred for 12 h at room temperature. The reaction was monitored by TLC until it was complete, then concentrated under reduced pressure. The residue was added to a saturated sodium chloride solution, extracted with ethyl acetate (3×50 mL), and combined with the organic phase, which was dried with anhydrous sodium sulfate, and then filtered and concentrated. Finally, the concentrated residue was purified by flash column chromatography (DCM/MeOH = 20:1) to give the intermediate **2b**.

*(R)-7-(benzyloxy)-12-methyl-3,4,12,12a-tetrahydro-1H-[1,4]oxazino[3,4-c]pyrido[2,1-f][1,2,4]triazine-6,8-dione (**2b**)*. ESI-MS: m/z 342.15 $[M+1]^{+}$. $C_{18}H_{19}IN_3O_4$ (342.47).

*(R)-7-(benzyloxy)-12-ethyl-3,4,12,12a-tetrahydro-1H-[1,4]oxazino[3,4-c]pyrido[2,1-f][1,2,4]triazine-6,8-dione (**3b**)*. Intermediate **3b** is synthesized following the procedure used for the preparation of Intermediate **2b**, with ethane iodide substituted for iodomethane. ESI-MS: m/z 356.16 $[M+1]^{+}$. $C_{19}H_{21}IN_3O_4$ (356.08).

*(R)-12-benzyl-7-(benzyloxy)-3,4,12,12a-tetrahydro-1H-[1,4]oxazino[3,4-c]pyrido[2,1-f][1,2,4]triazine-6,8-dione (**4b**)*. Intermediate **4b** is synthesized following the procedure used for the preparation of Intermediate **2b**, with benzyl bromide substituted for iodomethane. ESI-MS: m/z 417.17 $[M+1]^{+}$. $C_{24}H_{23}N_3O_4$ (417.47).

(R)-7-(benzyloxy)-12-(4-iodobenzyl)-3,4,12,12a-tetrahydro-1H-[1,4]oxazino[3,4-c]pyrido[2,1-f][1,2,4]triazine-6,8-dione ***(5b).*** Intermediate **5b** is synthesized following the procedure used for the preparation of Intermediate **2b**, with 4-iodobenzyl bromide substituted for iodomethane. ^{1}H NMR (400 MHz, DMSO-d_6) δ 7.67 (d, J = 8.2 Hz, 2H), 7.53 (d, J = 6.7 Hz, 2H), 7.40 – 7.28 (m, 3H), 7.06 (s, 1H), 7.00 (d, J = 8.2 Hz, 2H), 5.92 (d, J = 7.7 Hz, 1H), 5.14 (s, 2H), 4.99 (s, 1H), 4.35 – 4.14 (m, 2H), 3.99 – 3.76 (m, 3H), 3.60 (s, 2H), 3.19 (d, J = 10.2 Hz, 1H). ESI-MS: m/z 543.80 [M+1]$^+$. $C_{24}H_{22}IN_3O_4$ (543.07).

*(R)-7-(benzyloxy)-12-(4-fluorobenzyl)-3,4,12,12a-tetrahydro-1H-[1,4]oxazino[3,4-c]pyrido[2,1-f][1,2,4]triazine-6,8-dione (**6b**).* Intermediate **6b** is synthesized following the procedure used for the preparation of Intermediate **2b**, with 4-fluorobenzyl bromide substituted for iodomethane. ESI-MS: m/z 435.16 [M+1]$^+$. $C_{24}H_{22}FN_3O_4$ (435.10).

*(R)-7-(benzyloxy)-12-(4-(trifluoromethyl)benzyl)-3,4,12,12a-tetrahydro-1H-[1,4]oxazino[3,4-c]pyrido[2,1-f][1,2,4]triazine-6,8-dione (**7b**).* Intermediate **7b** is synthesized following the procedure used for the preparation of Intermediate **2b**, with 4-trifluoromethylbenzyl bromide substituted for iodomethane. ESI-MS: m/z 485.16 [M+1]$^+$. $C_{25}H_{22}FN_3O_4$ (485.64).

*(R)-7-(benzyloxy)-12-(2-fluorobenzyl)-3,4,12,12a-tetrahydro-1H-[1,4]oxazino[3,4-c]pyrido[2,1-f][1,2,4]triazine-6,8-dione (**8b**).* Intermediate **8b** is synthesized following the procedure used for the preparation of Intermediate **2b**, with 2-fluorobenzyl bromide substituted for iodomethane. ESI-MS: m/z 435.16 [M+1]$^+$. $C_{24}H_{22}FN_3O_4$ (435.87).

*(R)-7-(benzyloxy)-12-(3-fluorobenzyl)-3,4,12,12a-tetrahydro-1H-[1,4]oxazino[3,4-c]pyrido[2,1-f][1,2,4]triazine-6,8-dione (**9b**).* Intermediate **9b** is synthesized following the procedure used for the preparation of Intermediate **2b**, with 3-fluorobenzyl bromide substituted for iodomethane. ESI-MS: m/z 435.16 [M+1]$^+$. $C_{24}H_{22}FN_3O_4$ (435.55).

*(R)-7-(benzyloxy)-12-(4-chloro-3-fluorobenzyl)-3,4,12,12a-tetrahydro-1H-[1,4]oxazino[3,4-c]pyrido[2,1-f][1,2,4]triazine-6,8-dione (**10b**).* Intermediate **10b** is synthesized following the procedure used for the preparation of Intermediate **2b**, with 4-chloro-3-fluorobenzyl bromide substituted for iodomethane. ESI-MS: m/z 469.12 [M+1]$^+$. $C_{24}H_{21}FClN_3O_4$ (469.90).

*(R)-7-(benzyloxy)-12-(naphthalen-1-ylmethyl)-3,4,12,12a-tetrahydro-1H-[1,4]oxazino[3,4-c]pyrido[2,1-f][1,2,4]triazine-6,8-dione (**11b**).* Intermediate **11b** is synthesized following the procedure used for the preparation of Intermediate **2b**, with 1-chloromethyl naphthalene substituted for iodomethane. ESI-MS: m/z 467.18 [M+1]$^+$. $C_{28}H_{25}N_3O_4$ (469.53).

*(R)-7-(benzyloxy)-12-(pyren-4-ylmethyl)-3,4,12,12a-tetrahydro-1H-[1,4]oxazino[3,4-c]pyrido[2,1-f][1,2,4]triazine-6,8-dione (**12b**).* Intermediate **12b** is synthesized following the procedure used for the preparation of Intermediate **2b**, with 1-chloromethylpyrene substituted for iodomethane. ESI-MS: m/z 541.20 [M+1]$^+$. $C_{34}H_{27}N_3O_4$ (541.67).

General procedure for the synthesis of intermediates 13c-40c. A mixture of **5b** (0.2 g, 0.36 mmol, 1 eq.), pyridine-4-boronic acid (0.72 mmol, 2 eq.), Cs_2CO_3 (0.18 g, 0.55 mmol, 1.5 eq.), and $PdCl_2$ (dppf) DCM adduct (0.032 g, 0.039 mmol, 0.1 eq.) was weighed into the 50 ml bottle, and a mixed solution of 12 mL dioxane and 2 mL water (dioxane: water = 6:1) was added, and the reaction was heated at 95 °C under nitrogen protection for 11 h. After the reaction was completed, the mixture

was filtered with diatomite, and the filtrate was transferred to the separating funnel, extracted with ethyl acetate (3×20 mL), combined with the organic phase, added anhydrous sodium sulfate to the organic phase for drying, filtration, concentration, finally the residue was purified by column chromatography (DCM: MeOH = 20:1) to obtain the intermediate **13c**. *(R)-7-(benzyloxy)-12-(4-(pyridin-4-yl)benzyl)-3,4,12,12a-tetrahydro-1H-[1,4]oxazino[3,4-c]pyrido[2,1-f][1,2,4]triazine-6,8-dione (**13c**).* ^{1}H NMR (400 MHz, DMSO-d_6) δ 8.64 (d, J = 5.1 Hz, 2H), 7.76 (dd, J = 23.6, 6.5 Hz, 4H), 7.54 (d, J = 7.4 Hz, 2H), 7.43 – 7.27 (m, 5H), 7.12 (s, 1H), 5.93 (d, J = 7.7 Hz, 1H), 5.15 (s, 2H), 5.04 (s, 1H), 4.28 (d, J = 83.0 Hz, 3H), 3.90 (d, J = 56.1 Hz, 4H), 3.30 – 3.14 (m, 1H). ESI-MS: m/z 495.57 [M+1]$^+$. $C_{29}H_{26}N_4O_4$ (494.20).

*(R)-7-(benzyloxy)-12-(4-(pyrimidin-5-yl)benzyl)-3,4,12,12a-tetrahydro-1H-[1,4]oxazino[3,4-c]pyrido[2,1-f][1,2,4]triazine-6,8-dione (**14c**).* Intermediate **14c** is synthesized following the procedure used for the preparation of Intermediate **13c**, with 5-pyrimidinylboronic acid substituted for pyridine-4-boronic acid. ESI-MS: m/z 496.20 [M+1]$^+$. $C_{28}H_{26}N_5O_4$ (496.55).

*(R)-12-(4-(1H-pyrazol-4-yl)benzyl)-7-(benzyloxy)-3,4,12,12a-tetrahydro-1H-[1,4]oxazino[3,4-c]pyrido[2,1-f][1,2,4]triazine-6,8-dione (**15c**).* Intermediate **15c** is synthesized following the procedure used for the preparation of Intermediate **13c**, with 1*H*-pyrazole-4-boronic acid substituted for pyridine-4-boronic acid. ESI-MS: m/z 484.20 [M+1]$^+$. $C_{27}H_{26}N_5O_4$ (484.54).

*(R)-7-(benzyloxy)-12-(4-(furan-2-yl)benzyl)-3,4,12,12a-tetrahydro-1H-[1,4]oxazino[3,4-c]pyrido[2,1-f][1,2,4]triazine-6,8-dione (**16c**).* Intermediate **16c** is synthesized following the procedure used for the preparation of Intermediate **13c**, with 2-furanboronic acid substituted for pyridine-4-boronic acid. ESI-MS: m/z 483.18 [M+1]$^+$. $C_{28}H_{25}N_3O_5$ (483.26).

*(R)-12-([1,1'-biphenyl]-4-ylmethyl)-7-(benzyloxy)-3,4,12,12a-tetrahydro-1H-[1,4]oxazino[3,4-c]pyrido[2,1-f][1,2,4]triazine-6,8-dione (**17c**).* Intermediate **17c** is synthesized following the procedure used for the preparation of Intermediate **13c**, with phenylboronic acid substituted for pyridine-4-boronic acid. ESI-MS: m/z 483.18 [M+1]$^+$. $C_{30}H_{27}N_3O_4$ (483.26).

*(R)-12-(4-(benzo[d][1,3]dioxol-5-yl)benzyl)-7-(benzyloxy)-3,4,12,12a-tetrahydro-1H-[1,4]oxazino[3,4-c]pyrido[2,1-f][1,2,4]triazine-6,8-dione (**18c**).* Intermediate **18c** is synthesized following the procedure used for the preparation of Intermediate **13c**, with 2-(benzo[*d*][1,3]dioxol-5-yl)-4,4,5,5-tetramethyl-1,3,2-dioxaborolane substituted for pyridine-4-boronic acid. ESI-MS: m/z 538.20 [M+1]$^+$. $C_{31}H_{28}N_3O_6$ (538.66).

*(R)-12-(4-(1H-indol-5-yl)benzyl)-7-(benzyloxy)-3,4,12,12a-tetrahydro-1H-[1,4]oxazino[3,4-c]pyrido[2,1-f][1,2,4]triazine-6,8-dione (**19c**).* Intermediate **19c** is synthesized following the procedure used for the preparation of Intermediate **13c**, with 5-Indoleboronic acid pinacol ester substituted for pyridine-4-boronic acid. ESI-MS: m/z 533.22 [M+1]$^+$. $C_{32}H_{29}N_4O_4$ (538.66).

*(R)-7-(benzyloxy)-12-(4-(pyren-2-yl)benzyl)-3,4,12,12a-tetrahydro-1H-[1,4]oxazino[3,4-c]pyrido[2,1-f][1,2,4]triazine-6,8-dione (**20c**).* Intermediate **20c** is synthesized following the procedure used for the preparation of Intermediate **13c**, with pyren-1-ylboronic acid substituted for pyridine-4-boronic acid. ESI-MS: m/z 618.24 [M+1]$^+$. $C_{40}H_{32}N_3O_4$ (618.71).

(R)-7-(benzyloxy*)-12-((2'-hydroxy-[1,1'-biphenyl]-4-yl)methyl)-3,4,12,12a-tetrahydro-1H-[1,4]oxazino[3,4-c]pyrido[2,1-f][1,2,4]triazine-6,8-dione (**21c**).* ^{1}H NMR (400 MHz, DMSO-d_6)

δ 9.55 (s, 1H), 7.52 (dd, J = 12.8, 7.7 Hz, 4H), 7.41 – 7.28 (m, 3H), 7.27 – 7.10 (m, 5H), 6.94 (d, J = 8.0 Hz, 1H), 6.87 (t, J = 7.4 Hz, 1H), 5.94 (d, J = 7.7 Hz, 1H), 5.15 (s, 2H), 5.00 (s, 1H), 4.50 – 3.73 (m, 7H), 3.28 – 3.18 (m, 1H). ESI-MS: m/z 510.45 $[M+1]^+$. $C_{30}H_{27}N_3O_5$ (509.20).

*(R)-7-(benzyloxy)-12-((3'-hydroxy-[1,1'-biphenyl]-4-yl)methyl)-3,4,12,12a-tetrahydro-1H-[1,4]oxazino[3,4-c]pyrido[2,1-f][1,2,4]triazine-6,8-dione (**22c**)*. Intermediate **22c** is synthesized following the procedure used for the preparation of Intermediate **13c**, with 3-hydroxyphenylboronic acid substituted for pyridine-4-boronic acid. ESI-MS: m/z 510.20 $[M+1]^+$. $C_{30}H_{28}N_3O_5$ (510.57).

*(R)-7-(benzyloxy)-12-((4'-hydroxy-[1,1'-biphenyl]-4-yl)methyl)-3,4,12,12a-tetrahydro-1H-[1,4]oxazino[3,4-c]pyrido[2,1-f][1,2,4]triazine-6,8-dione (**23c**)*. Intermediate **23c** is synthesized following the procedure used for the preparation of Intermediate **13c**, with 4-hydroxyphenylboronic acid substituted for pyridine-4-boronic acid. ESI-MS: m/z 510.20 $[M+1]^+$. $C_{30}H_{28}N_3O_5$ (510.83).

*(R)-4'-((7-(benzyloxy)-6,8-dioxo-1,3,4,6,8,12a-hexahydro-12H-[1,4]oxazino[3,4-c]pyrido[2,1-f][1,2,4]triazin-12-yl)methyl)-[1,1'-biphenyl]-2-carbonitrile (**24c**)*. Intermediate **24c** is synthesized following the procedure used for the preparation of Intermediate **13c**, with 2-cyanophenylboronic acid substituted for pyridine-4-boronic acid. ESI-MS: m/z 519.20 $[M+1]^+$. $C_{31}H_{27}N_4O_4$ (519.58).

*(R)-4'-((7-(benzyloxy)-6,8-dioxo-1,3,4,6,8,12a-hexahydro-12H-[1,4]oxazino[3,4-c]pyrido[2,1-f][1,2,4]triazin-12-yl)methyl)-[1,1'-biphenyl]-3-carbonitrile (**25c**)*. Intermediate **25c** is synthesized following the procedure used for the preparation of Intermediate **13c**, with 3-cyanophenylboronic acid substituted for pyridine-4-boronic acid. ESI-MS: m/z 519.20 $[M+1]^+$. $C_{31}H_{27}N_4O_4$ (518.96).

*(R)-4'-((7-(benzyloxy)-6,8-dioxo-1,3,4,6,8,12a-hexahydro-12H-[1,4]oxazino[3,4-c]pyrido[2,1-f][1,2,4]triazin-12-yl)methyl)-[1,1'-biphenyl]-4-carbonitrile (**26c**)*. Intermediate **26c** is synthesized following the procedure used for the preparation of Intermediate **13c**, with 4-cyanophenylboronic acid substituted for pyridine-4-boronic acid. ESI-MS: m/z 519.20 $[M+1]^+$. $C_{31}H_{27}N_4O_4$ (519.77).

*(R)-12-((2'-amino-[1,1'-biphenyl]-4-yl)methyl)-7-(benzyloxy)-3,4,12,12a-tetrahydro-1H-[1,4]oxazino[3,4-c]pyrido[2,1-f][1,2,4]triazine-6,8-dione (**27c**)*. Intermediate **27c** is synthesized following the procedure used for the preparation of Intermediate **13c**, with 2-aminophenylboronic acid substituted for pyridine-4-boronic acid. ESI-MS: m/z 508.21 $[M+1]^+$. $C_{30}H_{28}N_4O_4$ (508.64).

*(R)-12-((3'-amino-[1,1'-biphenyl]-4-yl)methyl)-7-(benzyloxy)-3,4,12,12a-tetrahydro-1H-[1,4]oxazino[3,4-c]pyrido[2,1-f][1,2,4]triazine-6,8-dione (**28c**)*. Intermediate **28c** is synthesized following the procedure used for the preparation of Intermediate **13c**, with 3-aminophenylboronic acid substituted for pyridine-4-boronic acid. ESI-MS: m/z 508.21 $[M+1]^+$. $C_{30}H_{28}N_4O_4$ (510.02).

*(R)-12-((4'-amino-[1,1'-biphenyl]-4-yl)methyl)-7-(benzyloxy)-3,4,12,12a-tetrahydro-1H-[1,4]oxazino[3,4-c]pyrido[2,1-f][1,2,4]triazine-6,8-dione (**29c**)*. Intermediate **29c** is synthesized following the procedure used for the preparation of Intermediate **13c**, with 4-aminophenylboronic acid substituted for pyridine-4-boronic acid. ESI-MS: m/z 508.21 $[M+1]^+$. $C_{30}H_{28}N_4O_4$ (509.10).

*(R)-7-(benzyloxy)-12-((4'-fluoro-[1,1'-biphenyl]-4-yl)methyl)-3,4,12,12a-tetrahydro-1H-[1,4]oxazino[3,4-c]pyrido[2,1-f][1,2,4]triazine-6,8-dione (**30c**)*. Intermediate **30c** is synthesized following the procedure used for the preparation of Intermediate **13c**, with 4-fluorobenzeneboronic acid substituted for pyridine-4-boronic acid. ESI-MS: m/z 511.19 $[M+1]^+$. $C_{30}H_{26}FN_3O_4$ (511.55).

*(R)-7-(benzyloxy)-12-((4'-chloro-[1,1'-biphenyl]-4-yl)methyl)-3,4,12,12a-tetrahydro-1H-[1,4]oxazino[3,4-c]pyrido[2,1-f][1,2,4]triazine-6,8-dione (**31c**)*. Intermediate **31c** is synthesized following the procedure used for the preparation of Intermediate **13c**, with 4-chlorophenylboronic acid substituted for pyridine-4-boronic acid. ESI-MS: m/z 528.17 $[M+1]^+$. $C_{30}H_{26}ClN_3O_4$ (529.01).

*(R)-7-(benzyloxy)-12-((4'-nitro-[1,1'-biphenyl]-4-yl)methyl)-3,4,12,12a-tetrahydro-1H-[1,4]oxazino[3,4-c]pyrido[2,1-f][1,2,4]triazine-6,8-dione (**32c**)*. Intermediate **32c** is synthesized following the procedure used for the preparation of Intermediate **13c**, with 4-nitrophenylboronic acid substituted for pyridine-4-boronic acid. ESI-MS: m/z 539.19 $[M+1]^+$. $C_{30}H_{27}N_4O_6$ (540.06).

*(R)-7-(benzyloxy)-12-((4'-methoxy-[1,1'-biphenyl]-4-yl)methyl)-3,4,12,12a-tetrahydro-1H-[1,4]oxazino[3,4-c]pyrido[2,1-f][1,2,4]triazine-6,8-dione (**33c**)*. Intermediate **33c** is synthesized following the procedure used for the preparation of Intermediate **13c**, with 4-methoxyphenylboronic acid substituted for pyridine-4-boronic acid. ESI-MS: m/z 524.22 $[M+1]^+$. $C_{31}H_{30}N_3O_5$ (525.01).

*(R)-7-(benzyloxy)-12-((4'-(trifluoromethoxy)-[1,1'-biphenyl]-4-yl)methyl)-3,4,12,12a-tetrahydro-1H-[1,4]oxazino[3,4-c]pyrido[2,1-f][1,2,4]triazine-6,8-dione (**34c**)*. Intermediate **34c** is synthesized following the procedure used for the preparation of Intermediate **13c**, with 4-trifluoromethoxyphenylboronic acid substituted for pyridine-4-boronic acid. ESI-MS: m/z 578.19 $[M+1]^+$. $C_{31}H_{27}F_3N_3O_5$ (578.96).

*(R)-4'-((7-(benzyloxy)-6,8-dioxo-1,3,4,6,8,12a-hexahydro-12H-[1,4]oxazino[3,4-c]pyrido[2,1-f][1,2,4]triazin-12-yl)methyl)-[1,1'-biphenyl]-4-carboxylic acid (**35c**)*. Intermediate **35c** is synthesized following the procedure used for the preparation of Intermediate **13c**, with 4-carboxyphenylboronic acid substituted for pyridine-4-boronic acid. ESI-MS: m/z 538.20 $[M+1]^+$. $C_{31}H_{28}N_3O_6$ (538.84).

*(R)-4'-((7-(benzyloxy)-6,8-dioxo-1,3,4,6,8,12a-hexahydro-12H-[1,4]oxazino[3,4-c]pyrido[2,1-f][1,2,4]triazin-12-yl)methyl)-[1,1'-biphenyl]-4-carboxamide (**36c**)*. Intermediate **36c** is synthesized following the procedure used for the preparation of Intermediate **13c**, with (4-carbamoylphenyl)boronic acid substituted for pyridine-4-boronic acid. ESI-MS: m/z 537.21 $[M+1]^+$. $C_{32}H_{29}N_4O_5$ (537.60).

*(R)-4'-((7-(benzyloxy)-6,8-dioxo-1,3,4,6,8,12a-hexahydro-12H-[1,4]oxazino[3,4-c]pyrido[2,1-f][1,2,4]triazin-12-yl)methyl)-N-methyl-[1,1'-biphenyl]-4-carboxamide (**37c**)*. Intermediate **37c** is synthesized following the procedure used for the preparation of Intermediate **13c**, with (4-(methylcarbamoyl)phenyl)boronic acid substituted for pyridine-4-boronic acid. ESI-MS: m/z 551.23 $[M+1]^+$. $C_{32}H_{31}N_4O_5$ (551.62).

*(R)-7-(benzyloxy)-12-((4'-(methylsulfonyl)-[1,1'-biphenyl]-4-yl)methyl)-3,4,12,12a-tetrahydro-1H-[1,4]oxazino[3,4-c]pyrido[2,1-f][1,2,4]triazine-6,8-dione (**38c**)*. Intermediate **38c** is

synthesized following the procedure used for the preparation of Intermediate **13c**, with (4-(methylcarbamoyl)phenyl)boronic acid substituted for pyridine-4-boronic acid. ESI-MS: m/z 551.23 $[M+1]^+$. $C_{32}H_{31}N_4O_5$ (551.62).

*(R)-4'-((7-(benzyloxy)-6,8-dioxo-1,3,4,6,8,12a-hexahydro-12H-[1,4]oxazino[3,4-c]pyrido[2,1-f][1,2,4]triazin-12-yl)methyl)-N-cyclopentyl-[1,1'-biphenyl]-4-carboxamide (**39c**).* Intermediate **39c** is synthesized following the procedure used for the preparation of Intermediate **13c**, with (4-(cyclopentylcarbamoyl)phenyl)boronic acid substituted for pyridine-4-boronic acid. ESI-MS: m/z 605.28 $[M+1]^+$. $C_{36}H_{37}N_4O_5$ (605.72).

*(R)-4'-((7-(benzyloxy)-6,8-dioxo-1,3,4,6,8,12a-hexahydro-12H-[1,4]oxazino[3,4-c]pyrido[2,1-f][1,2,4]triazin-12-yl)methyl)-N-phenyl-[1,1'-biphenyl]-4-carboxamide (**40c**).* Intermediate **40c** is synthesized following the procedure used for the preparation of Intermediate **13c**, with (4-(phenylcarbamoyl)phenyl)boronic acid substituted for pyridine-4-boronic acid. ESI-MS: m/z 613.25 $[M+1]^+$. $C_{36}H_{37}N_4O_5$ (613.69).

General procedure for the synthesis of compounds 1-40. The intermediates **1b-12b** or **13c-40c** were weighed and placed in 25 mL nightshade bottles respectively and dissolved with 10 mL *N,N*-dimethylformamide (DMF). Then lithium chloride (10 eq.) was added at room temperature, and the reaction was heated at 95 °C for 12 h. After the reaction was over, the solvent DMF was removed under pressure, 10 mL of water was added to the concentrated residue, and the solid was then precipitated, and filtered. The filter cake was dissolved by methanol chromatography and purified by HPLC to obtain the corresponding target compounds **1-40**.

*(R)-12-benzoyl-7-hydroxy-3,4,12,12a-tetrahydro-1H-[1,4]oxazino[3,4-c]pyrido[2,1-f][1,2,4]triazine-6,8-dione **(1)**.* Yellow solid, m.p.186-188 °C, yield 73.8%. ^1H NMR (400 MHz, DMSO-d_6) δ 7.83 – 7.73 (m, 3H, 3 × Ph-H), 7.62 (t, *J* = 7.8 Hz, 2H, 2 × Ph-H), 7.45 – 7.30 (m, 1H, CH=CH), 6.21 (d, *J* = 7.7 Hz, 1H, CH=CH), 5.47 (d, *J* = 10.1 Hz, 1H, CH), 4.32 (t, *J* = 14.4 Hz, 3H, 3 × CH), 3.93 (t, *J* = 10.7 Hz, 1H, CH), 3.68 (d, *J* = 11.5 Hz, 1H, CH), 3.24 (d, *J* = 12.3 Hz, 1H, CH). ^{13}C NMR (100 MHz, DMSO-d_6) δ 173.58, 172.18, 160.73, 152.23, 140.12, 133.96, 130.81, 129.70, 129.46, 129.05, 128.74, 127.35, 117.98, 112.78, 70.29, 67.20, 65.70. HRMS (ESI): m/z calcd for $C_{17}H_{15}N_3O_5$ $[M+H]^+$ 342.1090; found 342.1090.

*(R)-7-hydroxy-12-methyl-3,4,12,12a-tetrahydro-1H-[1,4]oxazino[3,4-c]pyrido[2,1-f][1,2,4]triazine-6,8-dione **(2)**.* Yellow solid, m.p.130-132 °C, yield 20.3%. ^1H NMR (400 MHz, DMSO-d_6) δ 7.70 (d, *J* = 7.5 Hz, 1H, CH=CH), 6.12 (d, *J* = 7.3 Hz, 1H, CH=CH), 5.03 (dd, *J* = 10.4, 4.0 Hz, 1H, CH), 4.17 (d, *J* = 13.3 Hz, 1H, CH), 4.01 – 3.85 (m, 2H, 2 × CH), 3.62 – 3.45 (m, 2H, 2 × CH), 3.18 (dt, *J* = 13.0, 6.0 Hz, 1H, CH), 2.79 (s, 3H, NCH_3). ^{13}C NMR (100 MHz, DMSO-d_6) δ 171.41, 162.47, 153.22, 137.67, 116.21, 113.27, 71.21, 66.70, 65.85, 42.79, 42.58. HRMS (ESI): m/z calcd for $C_{11}H_{13}N_3O_4$ $[M+H]^+$ 252.0984; found 252.0983.

*(R)-12-ethyl-7-hydroxy-3,4,12,12a-tetrahydro-1H-[1,4]oxazino[3,4-c]pyrido[2,1-f][1,2,4]triazine-6,8-dione **(3)**.* Yellow solid, m.p.135-136 °C, yield 25.4%. ^1H NMR (400 MHz, DMSO-d_6) δ 7.35 (s, 1H, CH=CH), 5.69 (s, 1H, CH=CH,), 4.75 (s, 1H, CH), 4.26 (s, 1H, CH), 3.90 – 3.68 (m, 2H, 2 × CH), 3.06 (s, 3H, 3 × CH), 2.95 (s, 1H, CH), 2.79 (s, 1H, CH), 0.94 (t, *J* = 7.1 Hz, 3H, CH_3). ^{13}C NMR (100 MHz, DMSO-d_6) δ 171.36, 161.50, 136.72, 114.06, 71.58,

66.08, 35.13, 21.57, 13.03. HRMS (ESI): m/z calcd for $C_{12}H_{15}N_3O_4$ $[M+H]^+$ 266.1141; found 266.1141.

(R)-12-benzyl-7-hydroxy-3,4,12,12a-tetrahydro-1H-[1,4]oxazino[3,4-c]pyrido[2,1-f][1,2,4]triazine-6,8-dione ***(4)***. Red brown solid, m.p. 223-225°C, yield 28.4%. ^{1}H NMR (400 MHz, DMSO-d_6) δ 7.35 – 7.30 (m, 3H, 3 × Ph-H), 7.29 – 7.23 (m, 2H, 2 × Ph-H), 7.03 (d, J = 7.6 Hz, 1H, CH=CH), 5.76 (d, J = 7.5 Hz, 1H, CH=CH), 5.12 – 4.98 (m, 1H, CH), 4.28 (s, 3H, 3 × CH), 3.98 – 3.75 (m, 3H, 3 × CH), 3.68 – 3.64 (m, 1H, CH), 3.18 (d, J = 12.5 Hz, 1H, CH). ^{13}C NMR (100 MHz, DMSO-d_6) δ 171.47, 153.34, 138.36, 135.08, 130.36, 129.00, 128.89, 116.21, 111.65, 65.98. HRMS (ESI): m/z calcd for $C_{17}H_{17}N_3O_4$ $[M+H]^+$ 328.1297; found 328.1298.

(R)-7-hydroxy-12-(4-iodobenzyl)-3,4,12,12a-tetrahydro-1H-[1,4]oxazino[3,4-c]pyrido[2,1-f][1,2,4]triazine-6,8-dione ***(5)***. Yellow solid, m.p. 240-242°C, yield 52.3%. ^{1}H NMR (400 MHz, DMSO-d_6) δ 7.69 (d, J = 8.0 Hz, 2H, 2 × Ph-H), 7.08 (d, J = 8.1 Hz, 3H, 2 × Ph-H, CH=CH), 5.77 (s, 1H, CH=CH), 5.02 (s, 1H, CH), 4.25 (s, 3H, 3 × CH), 3.98 – 3.75 (m, 2H, CH_2), 3.71 – 3.49 (m, 2H, 2 × CH), 3.17 (s, 1H, CH). ^{13}C NMR (100 MHz, DMSO-d_6) δ 171.73, 153.26, 138.55, 137.80, 134.73, 132.59, 95.25, 65.97. HRMS (ESI): m/z calcd for $C_{17}H_{16}IN_3O_4$ $[M+H]^+$ 454.0263; found 454.0260.

(R)-12-(4-fluorobenzyl)-7-hydroxy-3,4,12,12a-tetrahydro-1H-[1,4]oxazino[3,4-c]pyrido[2,1-f][1,2,4]triazine-6,8-dione ***(6)***. Light orange solid, m.p. 253–256 °C, yield 49.4%. ^{1}H NMR (400 MHz, DMSO-d_6) δ 11.72 (s, 1H, OH), 7.30 (dd, J = 8.4, 5.7 Hz, 2H, 2 × Ph-H), 7.15 (t, J = 8.8 Hz, 2H, 2 × Ph-H), 7.02 (d, J = 7.4 Hz, 1H, CH=CH), 5.81 – 5.75 (m, 1H, CH=CH), 5.05 (d, J = 9.7 Hz, 1H, CH), 4.29 (d, J = 9.1 Hz, 3H, 3 × CH), 3.87 (dd, J = 34.9, 9.8 Hz, 2H, CH_2), 3.66 (t, J = 10.5 Hz, 1H, CH), 3.51 (t, J = 11.7 Hz, 1H, CH), 3.24 (td, J = 12.5, 3.8 Hz, 1H, CH). ^{13}C NMR (100 MHz, DMSO-d_6) δ 171.48, 163.76, 161.33, 153.36, 138.41, 132.62, 132.54, 131.43, 131.40, 116.20, 115.93, 115.72, 111.62, 65.97, 55.39. ^{13}C NMR (100 MHz, DMSO-d_6) δ 171.48, 163.76, 161.33, 153.36, 138.41, 132.62, 132.54, 131.43, 131.40, 116.20, 115.93, 115.72, 111.62, 65.97, 55.39. HRMS (ESI): m/z calcd for $C_{17}H_{16}FN_3O_4$ $[M+H]^+$ 346.1203; found 346.1201.

(R)-7-hydroxy-12-(4-(trifluoromethyl)benzyl)-3,4,12,12a-tetrahydro-1H-[1,4]oxazino[3,4-c]pyrido[2,1-f][1,2,4]triazine-6,8-dione ***(7)***. Light orange solid, m.p. 239–242 °C, yield 52.1%. ^{1}H NMR (400 MHz, DMSO-d_6) δ 11.68 (s, 1H, OH), 7.71 (d, J = 7.9 Hz, 2H, 2 × Ph-H), 7.54 (d, J = 7.9 Hz, 2H, 2 × Ph-H), 7.11 (d, J = 7.6 Hz, 1H, CH=CH), 5.79 (d, J = 7.6 Hz, 1H, CH=CH), 5.07 (d, J = 9.7 Hz, 1H, CH), 4.49 – 4.21 (m, 3H, 3 × CH), 3.89 (dd, J = 44.6, 10.0 Hz, 2H, CH_2), 3.65 (t, J = 10.6 Hz, 1H, CH), 3.58 – 3.45 (m, 1H, CH), 3.25 (t, J = 12.6 Hz, 1H, CH). ^{13}C NMR (100 MHz, DMSO-d_6) δ 171.53, 153.41, 140.20, 138.30, 131.03, 129.73, 129.41, 129.10, 128.79, 128.69, 125.98, 125.88, 125.84, 125.80, 125.76, 123.28, 116.22, 111.77, 65.92. HRMS (ESI): m/z calcd for $C_{18}H_{16}F_3N_3O_4$ $[M+H]^+$ 396.1171; found 396.1166.

(R)-12-(2-fluorobenzyl)-7-hydroxy-3,4,12,12a-tetrahydro-1H-[1,4]oxazino[3,4-c]pyrido[2,1-f][1,2,4]triazine-6,8-dione ***(8)***. Yellow solid, m.p. 121-124°C, yield 45.8%. ^{1}H NMR (400 MHz, DMSO-d_6) δ 7.41 (q, J = 7.2 Hz, 1H, Ph-H), 7.28 (t, J = 7.8 Hz, 1H, Ph-H), 7.21 – 7.09 (m, 2H, 2 × Ph-H), 6.98 (s, 1H, CH=CH), 5.77 (s, 1H, CH=CH), 5.12 (s, 1H, CH), 4.35 (s, 3H, 3 × CH), 3.99 – 3.76 (m, 2H, CH_2), 3.67 (s, 1H, CH), 3.51 (s, 1H, CH), 3.22 (t, J = 11.9 Hz, 1H, CH). ^{13}C NMR (100 MHz, DMSO-d_6) δ 171.53, 162.82, 160.36, 153.28, 138.19, 133.20, 133.17, 131.66, 131.58,

125.08, 125.04, 121.96, 121.81, 116.22, 116.00, 115.79, 111.76, 65.93. HRMS (ESI): m/z calcd for $C_{17}H_{16}FN_3O_4$ $[M+H]^+$ 346.1203; found 346.1201.

(R)-12-(3-fluorobenzyl)-7-hydroxy-3,4,12,12a-tetrahydro-1H-[1,4]oxazino[3,4-c]pyrido[2,1-f][1,2,4]triazine-6,8-dione ***(9)***. Yellow solid, m.p.120-123 °C, yield 45.6%. ^{1}H NMR (400 MHz, DMSO-d_6) δ 7.37 (q, J = 7.4 Hz, 1H, Ph-H), 7.17 (t, J = 8.5 Hz, 3H, 3 × Ph-H), 7.10 (d, J = 7.6 Hz, 2H, Ph-H, CH=CH), 5.77 (s, 1H, CH=CH), 5.02 (s, 1H, CH), 4.30 (s, 3H, 3 × CH), 3.95 – 3.79 (m, 2H, CH_2), 3.61 (s, 2H, 2 × CH), 3.17 (s, 1H, CH). ^{13}C NMR (100 MHz, DMSO-d_6)δ 171.76, 164.23, 163.71, 161.29, 153.22, 138.54, 137.73, 131.07, 130.99, 126.41, 117.09, 116.88, 115.90, 115.69, 111.17, 65.96. HRMS (ESI): m/z calcd for $C_{17}H_{16}FN_3O_4$ $[M+Na]^+$ 368.1023; found 368.1023.

(R)-12-(4-chloro-3-fluorobenzyl)-7-hydroxy-3,4,12,12a-tetrahydro-1H-[1,4]oxazino[3,4-c]pyrido[2,1-f][1,2,4]triazine-6,8-dione ***(10)***. Light orange solid, m.p. 241–244 °C, yield 49.7%. ^{1}H NMR (400 MHz, DMSO-d_6) δ 11.75 (s, 1H, OH), 7.55 (t, J = 8.0 Hz, 1H, Ph-H), 7.43 (d, J = 10.1 Hz, 1H, CH=CH), 7.16 (t, J = 7.7 Hz, 2H, 2 × Ph-H), 5.83 (d, J = 7.5 Hz, 1H, CH=CH), 5.06 (s, 1H, CH), 4.38 – 4.20 (m, 3H, 3 × CH), 3.88 (dd, J = 41.8, 12.0 Hz, 2H, CH_2), 3.63 (t, J = 10.5 Hz, 1H, CH), 3.49 (d, J = 12.2 Hz, 1H, CH), 3.26 (d, J = 12.4 Hz, 1H, CH). ^{13}C NMR (100 MHz, DMSO-d_6) δ 171.53, 158.74, 156.29, 153.39, 138.44, 137.24, 131.14, 127.57, 119.93, 119.76, 118.74, 118.52, 116.22, 111.82, 65.92. HRMS (ESI): m/z calcd for $C_{17}H_{15}ClFN_3O_4$ $[M+H]^+$ 380.0813; found 380.0810.

(R)-7-hydroxy-12-(naphthalen-1-ylmethyl)-3,4,12,12a-tetrahydro-1H-[1,4]oxazino[3,4-c]pyrido[2,1-f][1,2,4]triazine-6,8-dione ***(11)***. Light orange solid, m.p. 279–282 °C, yield 45.2%. ^{1}H NMR (400 MHz, DMSO-d_6) δ 11.86 (s, 1H, OH), 8.26 (s, 1H, Ph-H), 7.95 (dd, J = 20.1, 8.2 Hz, 2H, 2 × Ph-H), 7.75 – 7.51 (m, 2H, 2 × Ph-H), 7.35 (t, J = 7.5 Hz, 1H, Ph-H), 7.26 – 7.11 (m, 1H, Ph-H), 6.40 (s, 1H, CH=CH), 5.52 (s, 1H, CH=CH), 5.29 (s, 1H, CH), 4.73 (s, 2H, CH_2), 4.38 (s, 1H, CH), 3.95 (d, J = 64.5 Hz, 2H, CH_2), 3.53 (d, J = 13.6 Hz, 2H, CH_2), 3.20 (d, J = 57.7 Hz, 1H, CH). ^{13}C NMR (150 MHz, DMSO-d_6) δ 171.36, 153.49, 138.01, 133.79, 132.01, 130.32, 129.98, 129.80, 129.08, 127.21, 126.55, 125.73, 124.62, 115.97, 111.07, 66.02, 55.33, 53.88, 42.14. HRMS (ESI): m/z calcd for $C_{21}H_{19}N_3O_4$ $[M+H]^+$ 378.1454; found 378.1455.

(R)-7-hydroxy-12-(pyren-1-ylmethyl)-3,4,12,12a-tetrahydro-1H-[1,4]oxazino[3,4-c]pyrido[2,1-f][1,2,4]triazine-6,8-dione ***(12)***. Yellow solid, m.p. 243-245°C, yield 60.4%. ^{1}H NMR (400 MHz, DMSO-d_6) δ 8.53 – 8.40 (m, 1H, Ph-H), 8.38 – 8.30 (m, 3H, 3 × Ph-H), 8.29 – 8.05 (m, 5H, 5 × Ph-H), 7.71 (d, J = 7.8 Hz, 1H, CH=CH), 6.43 (s, 1H, CH=CH), 5.46 – 4.83 (m, 4H), 4.42 (s, 2H, CH_2), 3.32 (d, J = 12.6 Hz, 2H, 2 × CH), 3.17 (s, 1H, CH). ^{13}C NMR (100 MHz, DMSO-d_6) δ 131.77, 131.15, 130.69, 130.10, 129.81, 128.70, 128.32, 127.94, 127.83, 126.99, 126.16, 126.12, 125.14, 124.44, 124.18, 123.88, 66.09. HRMS (ESI): m/z calcd for $C_{21}H_{19}N_3O_4$ $[M+H]^+$ 452.1610; found 452.1610.

(R)-7-hydroxy-12-(4-(pyridin-4-yl)benzyl)-3,4,12,12a-tetrahydro-1H-[1,4]oxazino[3,4-c]pyrido[2,1-f][1,2,4]triazine-6,8-dione ***(13)***. Light orange solid, m.p. 260–263 °C, yield 49.7%. ^{1}H NMR (400 MHz, DMSO-d_6) δ 11.75 (s, 1H, OH), 8.64 (d, J = 5.0 Hz, 2H, 2 × Ph-H), 7.80 (d, J = 7.9 Hz, 2H, 2 × Ph-H), 7.73 (d, J = 5.0 Hz, 2H, 2 × Ph-H), 7.44 (d, J = 7.8 Hz, 2H, 2 × Ph-H), 7.13 (d, J = 7.6 Hz, 1H, CH=CH), 5.80 (d, J = 7.6 Hz, 1H, CH=CH), 5.07 (d, J = 10.1 Hz, 1H,

CH), 4.44 – 4.24 (m, 3H, 3 × CH), 3.89 (dd, *J* = 41.2, 10.7 Hz, 2H, CH_2), 3.67 (t, *J* = 9.7 Hz, 1H, CH), 3.50 (d, *J* = 11.4 Hz, 1H, CH), 3.26 (t, *J* = 12.6 Hz, 1H, CH). ^{13}C NMR (100 MHz, DMSO-d_6) δ 171.53, 153.41, 150.76, 146.72, 138.41, 137.36, 136.47, 131.15, 127.33, 121.55, 116.24, 111.80, 65.97. HRMS (ESI): m/z calcd for $C_{22}H_{20}N_4O_4$ $[M+H]^+$ 405.1563; found 405.1560.

(R)-7-hydroxy-12-(4-(pyrimidin-5-yl)benzyl)-3,4,12,12a-tetrahydro-1H-[1,4]oxazino[3,4-c]pyrido[2,1-f][1,2,4]triazine-6,8-dione ***(14)***. Light orange solid, m.p. 242–244 ºC, yield 37%. ^{1}H NMR (400 MHz, DMSO-d_6) δ 11.75 (s, 1H, OH), 9.18 (d, *J* = 10.2 Hz, 3H, 3 × Ph-H), 7.93 – 7.72 (m, 2H, 2 × Ph-H), 7.47 (d, *J* = 7.1 Hz, 2H, 2 × Ph-H), 7.15 (d, *J* = 7.6 Hz, 1H, CH=CH), 5.86 – 5.74 (m, 1H, CH=CH), 5.06 (s, 1H, CH), 4.34 (d, *J* = 18.4 Hz, 3H, 3 × CH), 4.03 – 3.76 (m, 2H, CH_2), 3.68 (d, *J* = 9.5 Hz, 1H, CH), 3.58 – 3.44 (m, 1H, CH), 3.27 (d, *J* = 13.3 Hz, 1H, CH). ^{13}C NMR (100 MHz, DMSO-d_6) δ 157.85, 155.13, 138.37, 136.18, 134.07, 133.02, 131.20, 127.40, 116.20, 111.79, 65.98, 55.39. HRMS (ESI): m/z calcd for $C_{21}H_{19}N_5O_4$ $[M+H]^+$ 406.1515; found 406.1512.

(R)-12-(4-(1H-pyrazol-4-yl)benzyl)-7-hydroxy-3,4,12,12a-tetrahydro-1H-[1,4]oxazino[3,4-c]pyrido[2,1-f][1,2,4]triazine-6,8-dione ***(15)***. Light orange solid, m.p. 291–294 ºC, yield 52%. ^{1}H NMR (400 MHz, DMSO-d_6) δ 12.96 (s, 1H, NH), 11.77 (s, 1H, OH), 8.08 (s, 2H, 2CH=CH), 7.57 (d, *J* = 7.7 Hz, 2H, 2 × Ph-H), 7.21 (d, *J* = 7.8 Hz, 2H, 2 × Ph-H), 7.08 (d, *J* = 7.6 Hz, 1H, CH=CH), 5.78 (d, *J* = 7.7 Hz, 1H, CH=CH), 5.07 (s, 1H, CH), 4.26 (s, 3H, 3 × CH), 4.00 – 3.75 (m, 2H, CH_2), 3.66 (t, *J* = 9.8 Hz, 1H, CH), 3.49 (d, *J* = 12.8 Hz, 1H, CH), 3.26 (d, *J* = 10.3 Hz, 1H, CH). ^{13}C NMR (100 MHz, DMSO-d_6) δ 171.49, 153.36, 138.52, 133.47, 132.31, 130.92, 125.50, 121.10, 116.22, 111.68, 65.98, 55.39. HRMS (ESI): m/z calcd for $C_{20}H_{19}N_5O_4$ $[M+H]^+$ 394.1515; found 394.1515.

(R)-12-(4-(furan-2-yl)benzyl)-7-hydroxy-3,4,12,12a-tetrahydro-1H-[1,4]oxazino[3,4-c]pyrido[2,1-f][1,2,4]triazine-6,8-dione ***(16)***. Light orange solid, m.p. 225–228 ºC, yield 45.8%. ^{1}H NMR (400 MHz, DMSO-d_6) δ 11.71 (s, 1H, OH), 7.76 (d, *J* = 1.8 Hz, 1H, CH=CH), 7.65 (d, *J* = 8.0 Hz, 2H, 2 × Ph-H), 7.30 (d, *J* = 8.0 Hz, 2H, 2 × Ph-H), 7.06 (d, *J* = 7.7 Hz, 1H, CH=CH), 6.98 (d, *J* = 3.3 Hz, 1H, CH=CH), 6.60 (dd, *J* = 3.5, 1.8 Hz, 1H, CH=CH), 5.77 (d, *J* = 7.3 Hz, 1H, CH=CH), 5.07 (d, *J* = 8.1 Hz, 1H, CH), 4.40 – 4.19 (m, 3H, 3 × CH), 3.89 (dd, *J* = 39.9, 9.4 Hz, 2H, CH_2), 3.68 (d, *J* = 11.7 Hz, 1H, CH), 3.51 (t, *J* = 18.1 Hz, 1H, CH), 3.24 (td, *J* = 12.9, 3.8 Hz, 1H, CH). ^{13}C NMR (100 MHz, DMSO-d_6) δ 171.48, 153.37, 153.06, 143.59, 138.46, 134.13, 131.00, 130.74, 123.89, 116.21, 112.63, 111.67, 106.84, 65.98. HRMS (ESI): m/z calcd for $C_{21}H_{19}N_3O_5$ $[M+H]^+$ 394.1403; found 394.1402.

(R)-12-([1,1'-biphenyl]-4-ylmethyl)-7-hydroxy-3,4,12,12a-tetrahydro-1H-[1,4]oxazino[3,4-c]pyrido[2,1-f][1,2,4]triazine-6,8-dione ***(17)***. Light orange solid, m.p. 258–261 ºC, yield 50%. ^{1}H NMR (400 MHz, DMSO-d_6) δ 11.64 (s, 1H, OH), 7.66 (dd, *J* = 13.1, 7.8 Hz, 4H, 4 × Ph-H), 7.47 (t, *J* = 7.5 Hz, 2H, 2 × Ph-H), 7.36 (d, *J* = 8.0 Hz, 3H, 3 × Ph-H), 7.12 (d, *J* = 7.6 Hz, 1H, CH=CH), 5.80 (d, *J* = 7.5 Hz, 1H, CH=CH), 5.06 (d, *J* = 10.3 Hz, 1H, CH), 4.32 (s, 3H, 3 × CH), 3.88 (dd, *J* = 40.4, 9.9 Hz, 2H, CH_2), 3.66 (t, *J* = 10.1 Hz, 1H, CH), 3.50 (d, *J* = 11.9 Hz, 1H, CH), 3.25 (t, *J* = 10.8 Hz, 1H, CH). ^{13}C NMR (100 MHz, DMSO-d_6) δ 171.54, 153.44, 140.44, 139.89, 138.44, 134.36, 130.94, 129.45, 128.12, 127.15, 127.08, 116.22, 111.74, 65.98, 55.39. HRMS (ESI): m/z calcd for $C_{23}H_{21}N_3O_4$ $[M+H]^+$ 404.1610; found 404.1609.

(R)-12-(4-(benzo[d][1,3]dioxol-5-yl)benzyl)-7-hydroxy-3,4,12,12a-tetrahydro-1H-[1,4]oxazino[3,4-c]pyrido[2,1-f][1,2,4]triazine-6,8-dione ***(18)***. Light orange solid, m.p. 239–242 ºC, yield 47.5%. ^{1}H NMR (400 MHz, DMSO-d_6) δ 11.83 (s, 1H, OH), 7.58 (d, *J* = 7.9 Hz, 2H, 2 × Ph-H), 7.35 – 7.23 (m, 3H, 3 × Ph-H), 7.20 – 7.08 (m, 2H, 2 × Ph-H), 6.99 (d, *J* = 8.1 Hz, 1H, CH=CH), 6.06 (s, 2H, CH2), 5.80 (d, *J* = 7.6 Hz, 1H, CH=CH), 5.05 (s, 1H, CH), 4.31 (d, *J* = 12.3 Hz, 3H, 3 × CH), 3.98 – 3.79 (m, 2H, CH_2), 3.65 (d, *J* = 10.4 Hz, 1H, CH), 3.51 (t, *J* = 12.8 Hz, 1H, CH), 3.24 (td, *J* = 12.5, 3.6 Hz, 1H, CH). ^{13}C NMR (100 MHz, DMSO-d_6) δ 171.53, 153.43, 148.49, 147.45, 140.16, 138.43, 134.16, 133.84, 130.83, 126.85, 120.72, 116.19, 111.77, 109.15, 107.47, 101.65, 65.98. HRMS (ESI): m/z calcd for $C_{24}H_{21}N_3O_6$ $[M+H]^+$ 448.1508; found 448.1507.

(R)-12-(4-(1H-indol-6-yl)benzyl)-7-hydroxy-3,4,12,12a-tetrahydro-1H-[1,4]oxazino[3,4-c]pyrido[2,1-f][1,2,4]triazine-6,8-dione ***(19)***. Light orange solid, m.p. 243–246 ºC, yield 45%. ^{1}H NMR (400 MHz, DMSO-d_6) δ 11.82 (s, 1H, OH), 11.17 (s, 1H, NH), 7.83 (s, 1H, Ph-H), 7.63 (d, *J* = 7.5 Hz, 2H, 2 × Ph-H), 7.47 (d, *J* = 8.2 Hz, 1H, CH=CH), 7.43 – 7.36 (m, 2H, 2 × Ph-H), 7.31 (d, *J* = 7.6 Hz, 2H, 2 × Ph-H), 7.13 (d, *J* = 7.6 Hz, 1H, CH=CH), 6.49 (s, 1H, CH=CH), 5.81 (d, *J* = 7.4 Hz, 1H, CH=CH), 5.06 (s, 1H, CH), 4.30 (s, 3H, 3 × CH), 3.89 (dd, *J* = 39.5, 9.8 Hz, 2H, CH_2), 3.68 (d, *J* = 10.2 Hz, 1H, CH), 3.51 (t, *J* = 11.7 Hz, 1H, CH), 3.27 (d, *J* = 10.5 Hz, 1H, CH). ^{13}C NMR (100 MHz, DMSO-d_6) δ 171.55, 153.47, 142.26, 138.47, 136.06, 132.85, 131.03, 130.82, 128.72, 127.05, 126.61, 120.70, 118.61, 116.20, 112.29, 111.75, 102.04, 66.00, 49.07. HRMS (ESI): m/z calcd for $C_{25}H_{22}N_4O_4$ $[M+H]^+$ 443.1719; found 443.1716.

(R)-7-hydroxy-12-(4-(pyren-4-yl)benzyl)-3,4,12,12a-tetrahydro-1H-[1,4]oxazino[3,4-c]pyrido[2,1-f][1,2,4]triazine-6,8-dione ***(20)***. Red brown solid, m.p. 260-262ºC, yield 35.6%. ^{1}H NMR (400 MHz, DMSO-d_6) δ 8.44 – 8.28 (m, 3H, 3 × Ph-H), 8.28 – 8.16 (m, 3H, 3 × Ph-H), 8.12 (d, *J* = 7.6 Hz, 1H, Ph-H), 8.08 – 7.95 (m, 2H, 2 × Ph-H), 7.58 (d, *J* = 7.5 Hz, 2H, 2 × Ph-H), 7.49 (d, *J* = 7.8 Hz, 2H, 2 × Ph-H), 7.27 (s, 1H, CH=CH), 5.95 – 5.74 (m, 1H, CH=CH), 5.17 (s, 1H,CH), 4.55 – 4.26 (m, 3H, 3 × CH), 4.03 – 3.85 (m, 2H, CH_2), 3.82 – 3.67 (m, 2H, 2 × CH), 3.17 (s, 1H, CH). ^{13}C NMR (100 MHz, DMSO-d_6) δ 166.67, 142.27, 133.90, 130.87, 128.22, 127.31, 126.88, 124.60. HRMS (ESI): m/z calcd for $C_{33}H_{25}N_3O_4$ $[M+H]^+$ 528.1923; found 528.1927.

(R)-7-hydroxy-12-((2'-hydroxy-[1,1'-biphenyl]-4-yl)methyl)-3,4,12,12a-tetrahydro-1H-[1,4]oxazino[3,4-c]pyrido[2,1-f][1,2,4]triazine-6,8-dione ***(21)***. Light orange solid, m.p. 289–291 ºC, yield 42.6%. ^{1}H NMR (600 MHz, DMSO-d_6) δ 11.84 (s, 1H, OH), 9.56 (s, 1H, OH), 7.52 (d, *J* = 8.0 Hz, 2H, 2 × Ph-H), 7.30 (d, *J* = 7.8 Hz, 2H, 2 × Ph-H), 7.25 (dd, *J* = 7.6, 1.7 Hz, 1H, Ph-H), 7.16 (td, *J* = 7.7, 1.8 Hz, 2H, 2 × Ph-H), 6.94 (d, *J* = 8.1 Hz, 1H, CH=CH), 6.87 (t, *J* = 7.4 Hz, 1H, Ph-H), 5.83 (d, *J* = 7.6 Hz, 1H, CH=CH), 5.03 (s, 1H, CH), 4.29 (s, 3H, 3 × CH), 3.97 – 3.78 (m, 2H, CH_2), 3.67 (d, *J* = 10.7 Hz, 1H, CH), 3.52 (d, *J* = 12.1 Hz, 1H, CH), 3.29 – 3.22 (m, 1H, CH). ^{13}C NMR (100 MHz, DMSO-d_6) δ 171.53, 154.78, 153.41, 139.04, 138.45, 133.30, 130.77, 129.83, 129.59, 129.13, 127.48, 119.93, 116.56, 116.21, 111.91, 65.99. HRMS (ESI): m/z calcd for $C_{23}H_{21}N_3O_5$ $[M+H]^+$ 420.1559; found 420.1562.

(R)-7-hydroxy-12-((3'-hydroxy-[1,1'-biphenyl]-4-yl)methyl)-3,4,12,12a-tetrahydro-1H-[1,4]oxazino[3,4-c]pyrido[2,1-f][1,2,4]triazine-6,8-dione ***(22)***. Light orange solid, m.p. 271–274

°C, yield 41%. ^1H NMR (400 MHz, DMSO-d_6) δ 11.66 (s, 1H, OH), 9.53 (s, 1H, OH), 7.56 (d, *J* = 7.8 Hz, 2H, 2 × Ph-H), 7.33 (d, *J* = 7.8 Hz, 2H, 2 × Ph-H), 7.25 (t, *J* = 7.9 Hz, 1H, Ph-H), 7.09 (dd, *J* = 15.6, 7.7 Hz, 2H, 2 × Ph-H), 7.03 (s, 1H, Ph-H), 6.77 (d, *J* = 8.0 Hz, 1H, CH=CH), 5.80 (d, *J* = 7.6 Hz, 1H, CH=CH), 5.06 (d, *J* = 9.8 Hz, 1H, CH), 4.30 (d, *J* = 10.3 Hz, 3H, 3 × CH), 3.89 (dd, *J* = 39.6, 11.3 Hz, 2H, CH_2), 3.66 (t, *J* = 10.7 Hz, 1H, CH), 3.51 (t, *J* = 12.0 Hz, 1H, CH), 3.31 – 3.22 (m, 1H, CH). ^{13}C NMR (101 MHz, DMSO-d_6) δ 171.52, 158.31, 153.40, 141.35, 140.63, 138.44, 134.27, 130.87, 130.44, 127.06, 117.84, 116.22, 115.11, 113.88, 111.77, 65.98. HRMS (ESI): m/z calcd for $C_{23}H_{21}N_3O_5$ $[M+H]^+$ 420.1559; found 420.1559.

(R)-7-hydroxy-12-((4'-hydroxy-[1,1'-biphenyl]-4-yl)methyl)-3,4,12,12a-tetrahydro-1H-[1,4]oxazino[3,4-c]pyrido[2,1-f][1,2,4]triazine-6,8-dione ***(23)***. Light orange solid, m.p. 280–283 °C, yield 51.8%. ^1H NMR (600 MHz, DMSO-d_6) δ 11.71 (s, 1H, OH), 9.61 (s, 1H, OH), 7.55 (d, *J* = 7.8 Hz, 2H, 2 × Ph-H), 7.50 (d, *J* = 8.2 Hz, 2H, 2 × Ph-H), 7.28 (d, *J* = 7.8 Hz, 2H, 2 × Ph-H), 7.14 – 7.06 (m, 1H, CH=CH), 6.84 (d, *J* = 8.3 Hz, 2H, 2 × Ph-H), 5.79 (d, *J* = 7.6 Hz, 1H, CH=CH), 5.04 (s, 1H, CH), 4.29 (s, 3H, 3 × CH), 3.88 (d, *J* = 57.5 Hz, 2H, CH_2), 3.66 (s, 1H, CH), 3.50 (s, 1H, CH), 3.28 – 3.20 (m, 1H, CH). ^{13}C NMR (100 MHz, DMSO-d_6) δ 171.50, 157.77, 152.15, 140.48, 138.41, 133.09, 130.85, 130.56, 128.15, 126.31, 116.22, 111.61, 65.99, 55.48. HRMS (ESI): m/z calcd for $C_{23}H_{21}N_3O_5$ $[M+H]^+$ 420.1559; found 420.1556.

(R)-4'-((7-hydroxy-6,8-dioxo-1,3,4,6,8,12a-hexahydro-12H-[1,4]oxazino[3,4-c]pyrido[2,1-f][1,2,4]triazin-12-yl)methyl)-[1,1'-biphenyl]-2-carbonitrile ***(24)***. Light orange solid, m.p. 249–252 °C, yield 56%. ^1H NMR (400 MHz, DMSO-d_6) δ 11.71 (s, 1H, OH), 7.95 (d, *J* = 7.6 Hz, 1H, Ph-H), 7.80 (t, *J* = 7.6 Hz, 1H, Ph-H), 7.64 – 7.53 (m, 4H, 4 × Ph-H), 7.46 (d, *J* = 7.8 Hz, 2H, 2 × Ph-H), 7.14 (s, 1H, CH=CH), 5.82 (d, *J* = 7.6 Hz, 1H, CH=CH), 5.07 (d, *J* = 7.5 Hz, 1H, CH), 4.38 (s, 3H, 3 × CH), 3.90 (dd, *J* = 44.4, 10.9 Hz, 2H, CH_2), 3.67 (t, *J* = 10.6 Hz, 1H, CH), 3.51 (d, *J* = 8.8 Hz, 1H, CH), 3.31 – 3.21 (m, 1H, CH). ^{13}C NMR (100 MHz, DMSO-d_6) δ 171.52, 153.41, 144.53, 138.29, 135.98, 134.32, 134.01, 130.56, 130.54, 129.33, 128.82, 118.91, 116.25, 111.85, 110.72, 65.98. HRMS (ESI): m/z calcd for $C_{24}H_{20}N_4O_4$ $[M+H]^+$ 429.1563; found 429.1562.

(R)-4'-((7-hydroxy-6,8-dioxo-1,3,4,6,8,12a-hexahydro-12H-[1,4]oxazino[3,4-c]pyrido[2,1-f][1,2,4]triazin-12-yl)methyl)-[1,1'-biphenyl]-3-carbonitrile ***(25)***. Light orange solid, m.p. 217–220 °C, yield 43%. ^1H NMR (400 MHz, DMSO-d_6) δ 11.73 (s, 1H, OH), 8.18 (s, 1H, Ph-H), 8.05 (d, *J* = 8.7 Hz, 1H, Ph-H), 7.84 (d, *J* = 7.6 Hz, 1H, Ph-H), 7.75 (d, *J* = 7.9 Hz, 2H, 2 × Ph-H), 7.67 (t, *J* = 7.8 Hz, 1H, Ph-H), 7.41 (d, *J* = 7.9 Hz, 2H, 2 × Ph-H), 7.13 (d, *J* = 7.6 Hz, 1H, CH=CH), 5.81 (d, *J* = 7.7 Hz, 1H, CH=CH), 5.07 (s, 1H, CH), 4.33 (t, *J* = 14.6 Hz, 3H, 3 × CH), 3.89 (dd, *J* = 41.1, 10.1 Hz, 2H, CH_2), 3.66 (d, *J* = 10.0 Hz, 1H, CH), 3.51 (d, *J* = 11.6 Hz, 1H, CH), 3.25 (td, *J* = 12.5, 3.7 Hz, 1H, CH). ^{13}C NMR (100 MHz, DMSO-d_6) δ 171.52, 153.42, 140.98, 138.40, 138.23, 135.51, 131.86, 131.70, 131.07, 130.65, 127.42, 119.24, 116.22, 112.62, 111.81, 65.98. HRMS (ESI): m/z calcd for $C_{24}H_{20}N_4O_4$ $[M+H]^+$ 429.1563; found 429.1563.

(R)-4'-((7-hydroxy-6,8-dioxo-1,3,4,6,8,12a-hexahydro-12H-[1,4]oxazino[3,4-c]pyrido[2,1-f][1,2,4]triazin-12-yl)methyl)-[1,1'-biphenyl]-4-carbonitrile ***(26)***. Light orange solid, m.p. 237–240 °C, yield 51%. ^1H NMR (400 MHz, DMSO-d_6) δ 11.75 (s, 1H, OH), 7.97 – 7.88 (m, 4H, 4 × Ph-H), 7.74 (d, *J* = 7.8 Hz, 2H, 2 × Ph-H), 7.42 (d, *J* = 7.7 Hz, 2H, 2 × Ph-H), 7.13 (d, *J* = 7.6 Hz, 1H, CH=CH), 5.80 (d, *J* = 7.6 Hz, 1H, CH=CH), 5.06 (d, *J* = 8.0 Hz, 1H, CH), 4.31 (d, *J* = 29.2

Hz, 3H, 3 × CH), 3.89 (dd, *J* = 39.9, 10.3 Hz, 2H, CH_2), 3.67 (t, *J* = 10.3 Hz, 1H, CH), 3.51 (d, *J* = 10.0 Hz, 1H, CH), 3.25 (t, *J* = 10.8 Hz, 1H, CH). ^{13}C NMR (100 MHz, DMSO-d_6) δ 171.52, 153.41, 144.35, 138.46, 135.93, 133.36, 131.11, 127.96, 127.59, 119.31, 116.23, 111.82, 110.65, 65.97. HRMS (ESI): m/z calcd for $C_{24}H_{20}N_4O_4$ $[M+H]^+$ 429.1563; found 429.1562.

(R)-12-((2'-amino-[1,1'-biphenyl]-4-yl)methyl)-7-hydroxy-3,4,12,12a-tetrahydro-1H-[1,4]oxazino[3,4-c]pyrido[2,1-f][1,2,4]triazine-6,8-dione ***(27)***. Light orange solid, m.p. 214–217 °C, yield 39.9%. ^{1}H NMR (400 MHz, DMSO-d_6) δ 11.82 (s, 1H, OH), 7.33 (q, *J* = 7.9 Hz, 4H, 4 × Ph-H), 7.16 (d, *J* = 7.3 Hz, 1H, Ph-H), 7.05 (t, *J* = 7.7 Hz, 1H, Ph-H), 6.96 (d, J = 7.5 Hz, 1H, CH=CH), 6.76 (d, J = 8.0 Hz, 1H, Ph-H), 6.63 (t, J = 7.4 Hz, 1H, Ph-H), 5.81 (d, J = 7.6 Hz, 1H, CH=CH), 5.08 (d, J = 7.4 Hz, 1H, CH), 4.73 (s, 2H, NH_2), 4.31 (s, 3H, 3 × CH), 3.89 (dd, J = 39.6, 10.2 Hz, 2H, CH_2), 3.68 (d, J = 10.7 Hz, 1H, CH), 3.51 (t, J = 9.9 Hz, 1H, CH), 3.25 (t, J = 12.7 Hz, 1H, CH). ^{13}C NMR (100 MHz, DMSO-d6) δ 171.50, 153.30, 145.40, 140.19, 138.53, 133.44, 130.78, 130.37, 129.21, 128.80, 125.69, 125.66, 117.19, 116.33, 115.69, 111.77, 65.99, 55.39. HRMS (ESI): m/z calcd for $C_{23}H_{22}N_4O_4$ $[M+H]^+$ 419.1719; found 419.1716.

(R)-12-((3'-amino-[1,1'-biphenyl]-4-yl)methyl)-7-hydroxy-3,4,12,12a-tetrahydro-1H-[1,4]oxazino[3,4-c]pyrido[2,1-f][1,2,4]triazine-6,8-dione ***(28)***. Yellow solid, m.p. 180-182°C, yield 34.2%. ^{1}H NMR (400 MHz, DMSO-d_6) δ 7.52 (d, *J* = 7.8 Hz, 2H, 2× Ph-H), 7.30 (d, *J* = 7.8 Hz, 2H, 2× Ph-H), 7.09 (t, *J* = 7.5 Hz, 2H, Ph-H, CH=CH), 6.85 (s, 1H, Ph-H), 6.78 (d, *J* = 7.7 Hz, 1H, Ph-H), 6.57 (d, *J* = 8.0 Hz, 1H, Ph-H), 5.80 (d, *J* = 7.6 Hz, 1H, CH=CH), 5.20 (s, 2H, NH_2), 5.07 (s, 1H, CH), 4.31 (s, 3H, 3× CH), 3.96 – 3.81 (m, 2H, CH_2), 3.67 (s, 2H, 2× CH), 3.29 (s, 1H, CH). ^{13}C NMR (100 MHz, DMSO-d_6) δ 171.51, 153.39, 149.62, 141.39, 140.62, 138.46, 133.88, 130.80, 129.92, 126.90, 114.69, 113.79. HRMS (ESI): m/z calcd for $C_{23}H_{22}N_4O_4$ $[M+H]^+$ 419.1719; found 419.1719.

(R)-12-((4'-amino-[1,1'-biphenyl]-4-yl)methyl)-7-hydroxy-3,4,12,12a-tetrahydro-1H-[1,4]oxazino[3,4-c]pyrido[2,1-f][1,2,4]triazine-6,8-dione ***(29)***. Yellow solid, m.p. 278-280 °C, yield 33.5%. ^{1}H NMR (400 MHz, DMSO-d_6) δ 7.51 (s, 2H, 2× Ph-H), 7.37 (s, 2H, 2× Ph-H), 7.23 (s, 2H, 2× Ph-H), 7.08 (s, 1H, CH=CH), 6.63 (d, *J* = 8.1 Hz, 2H, 2× Ph-H), 5.77 (s, 1H, CH=CH), 5.28 (s, 2H, NH_2), 5.03 (s, 1H, CH), 4.27 (s, 3H, 3× CH), 3.97 – 3.73 (m, 3H, 3× CH), 3.71 – 3.63 (m, 1H, CH), 3.17 (s, 1H, CH). ^{13}C NMR (100 MHz, DMSO-d_6) δ 171.55, 152.91, 148.98, 139.87, 132.30, 130.70, 127.57, 127.07, 125.66, 114.71, 66.05. HRMS (ESI): m/z calcd for $C_{23}H_{22}N_4O_4$ $[M+H]^+$ 419.1719; found 419.1720.

(R)-12-((4'-fluoro-[1,1'-biphenyl]-4-yl)methyl)-7-hydroxy-3,4,12,12a-tetrahydro-1H-[1,4]oxazino[3,4-c]pyrido[2,1-f][1,2,4]triazine-6,8-dione ***(30)***. Light orange solid, m.p. 267–270 °C, yield 51%. ^{1}H NMR (400 MHz, DMSO-d_6) δ 11.77 (s, 1H, OH), 7.72 (dd, *J* = 8.4, 5.5 Hz, 2H, 2 × Ph-H), 7.63 (d, *J* = 7.8 Hz, 2H, 2 × Ph-H), 7.46 – 7.25 (m, 4H, 4 × Ph-H), 7.12 (d, *J* = 7.7 Hz, 1H, CH=CH), 5.80 (d, *J* = 7.6 Hz, 1H, CH=CH), 5.06 (d, *J* = 9.0 Hz, 1H, CH), 4.32 (s, 3H, 3 × CH), 3.89 (dd, *J* = 38.3, 10.2 Hz, 2H, CH_2), 3.67 (t, *J* = 10.1 Hz, 1H, CH), 3.51 (t, *J* = 8.7 Hz, 1H, CH), 3.32 – 3.20 (m, 1H, CH). ^{13}C NMR (100 MHz, DMSO-d_6) δ 171.50, 163.66, 161.22, 153.40, 139.42, 138.41, 136.40, 136.37, 134.37, 130.95, 129.13, 129.05, 127.09, 116.32, 116.22, 116.11, 111.79, 65.98. HRMS (ESI): m/z calcd for $C_{23}H_{20}FN_3O_4$ $[M+H]^+$ 422.1516; found 422.1513.

(R)-12-((4'-chloro-[1,1'-biphenyl]-4-yl)methyl)-7-hydroxy-3,4,12,12a-tetrahydro-1H-[1,4]oxazino[3,4-c]pyrido[2,1-f][1,2,4]triazine-6,8-dione ***(31)***. Light orange solid, m.p. 215–218 °C, yield 46.5%. ^{1}H NMR (400 MHz, DMSO-d_6) δ 11.63 (s, 1H, OH), 7.68 (dd, J = 23.8, 8.0 Hz, 4H, 4 × Ph-H), 7.52 (d, J = 8.1 Hz, 2H, 2 × Ph-H), 7.37 (d, J = 7.8 Hz, 2H, 2 × Ph-H), 7.12 (d, J = 7.7 Hz, 1H, CH=CH), 5.80 (d, J = 7.6 Hz, 1H, CH=CH), 5.06 (d, J = 11.4 Hz, 1H, CH), 4.46 – 4.24 (m, 3H, 3 × CH), 3.88 (dd, J = 39.0, 10.6 Hz, 2H, CH_2), 3.68 (d, J = 10.7 Hz, 1H, CH), 3.51 (t, J = 10.1 Hz, 1H, CH), 3.32 – 3.21 (m, 1H, CH). ^{13}C NMR (100 MHz, DMSO-d_6) δ 171.51, 153.42, 139.07, 138.70, 138.43, 134.81, 132.98, 131.02, 129.39, 128.86, 127.11, 116.22, 111.79, 65.98, 55.40. HRMS (ESI): m/z calcd for $C_{23}H_{20}ClN_3O_4$ [M+H]$^+$ 438.1220; found 438.1220.

(R)-7-hydroxy-12-((4'-nitro-[1,1'-biphenyl]-4-yl)methyl)-3,4,12,12a-tetrahydro-1H-[1,4]oxazino[3,4-c]pyrido[2,1-f][1,2,4]triazine-6,8-dione ***(32)***. Light orange solid, m.p. 214–217 °C, yield 34.7%. ^{1}H NMR (400 MHz, DMSO-d_6) δ 11.96 (s, 1H, OH), 8.30 (d, J = 8.5 Hz, 2H, 2 × Ph-H), 7.98 (d, J = 8.5 Hz, 2H, 2 × Ph-H), 7.78 (d, J = 7.9 Hz, 2H, 2 × Ph-H), 7.45 (d, J = 7.9 Hz, 2H, 2 × Ph-H), 7.14 (d, J = 7.6 Hz, 1H, CH=CH), 5.81 (d, J = 7.6 Hz, 1H, CH=CH), 5.07 (d, J = 8.9 Hz, 1H, CH), 4.37 (d, J = 9.8 Hz, 3H, 3 × CH), 3.90 (dd, J = 40.1, 10.8 Hz, 2H, CH_2), 3.67 (t, J = 10.6 Hz, 1H, CH), 3.53 (t, J = 10.9 Hz, 1H, CH), 3.29 – 3.22 (m, 1H, CH). ^{13}C NMR (100 MHz, DMSO-d_6) δ 171.53, 153.41, 147.19, 146.35, 138.40, 138.04, 136.25, 131.17, 128.25, 127.80, 124.58, 116.23, 111.82, 65.97. HRMS (ESI): m/z calcd for $C_{23}H_{20}N_4O_6$ [M+H]$^+$ 449.1461; found 449.1460.

(R)-7-hydroxy-12-((4'-methoxy-[1,1'-biphenyl]-4-yl)methyl)-3,4,12,12a-tetrahydro-1H-[1,4]oxazino[3,4-c]pyrido[2,1-f][1,2,4]triazine-6,8-dione ***(33)***. Light orange solid, m.p. 198–201 °C, yield 53.1%. ^{1}H NMR (400 MHz, DMSO-d_6) δ 11.81 (s, 1H, OH), 7.61 (dd, J = 12.3, 8.3 Hz, 4H, 4 × Ph-H), 7.31 (d, J = 8.0 Hz, 2H, 2 × Ph-H), 7.12 (d, J = 7.6 Hz, 1H, CH=CH), 7.02 (d, J = 8.7 Hz, 2H, 2 × Ph-H), 5.80 (d, J = 7.6 Hz, 1H, CH=CH), 5.05 (d, J = 9.4 Hz, 1H, CH), 4.30 (s, 3H, 3 × CH), 3.98 – 3.82 (m, 2H, CH_2), 3.80 (s, 3H, CH_3), 3.66 (t, J = 10.2 Hz, 1H, CH), 3.51 (t, J = 12.5 Hz, 1H, CH), 3.25 (td, J = 13.0, 12.2, 3.6 Hz, 1H, CH). ^{13}C NMR (100 MHz, DMSO-d_6) δ 171.51, 159.51, 153.41, 140.11, 138.45, 133.50, 132.21, 130.90, 128.18, 126.56, 116.20, 114.85, 111.76, 65.98, 55.65, 55.39. HRMS (ESI): m/z calcd for $C_{24}H_{23}N_3O_5$ [M+H]$^+$ 434.1716; found 434.1711.

(R)-7-hydroxy-12-((4'-(trifluoromethoxy)-[1,1'-biphenyl]-4-yl)methyl)-3,4,12,12a-tetrahydro-1H-[1,4]oxazino[3,4-c]pyrido[2,1-f][1,2,4]triazine-6,8-dione ***(34)***. Light orange solid, m.p. 219–221 °C, yield 39%. ^{1}H NMR (400 MHz, DMSO-d_6) δ 11.79 (s, 1H, OH), 7.81 (d, J = 7.9 Hz, 2H, 2 × Ph-H), 7.67 (d, J = 7.5 Hz, 2H, 2 × Ph-H), 7.45 (d, J = 8.2 Hz, 2H, 2 × Ph-H), 7.38 (d, J = 7.7 Hz, 2H, 2 × Ph-H), 7.13 (d, J = 7.0 Hz, 1H, CH=CH), 5.80 (d, J = 7.2 Hz, 1H, CH=CH), 5.07 (s, 1H, CH), 4.33 (s, 3H, 3 × CH), 3.89 (dd, J = 40.4, 9.9 Hz, 2H, CH_2), 3.68 (d, J = 10.2 Hz, 1H, CH), 3.52 (t, J = 9.4 Hz, 1H, CH), 3.26 (t, J = 10.8 Hz, 1H, CH). ^{13}C NMR (100 MHz, DMSO-d_6) δ 171.51, 153.40, 148.42, 139.23, 138.98, 138.41, 134.90, 131.01, 129.00, 127.30, 121.94, 121.86, 119.31, 116.22, 111.78, 65.98. HRMS (ESI): m/z calcd for $C_{24}H_{20}F_3N_3O_5$ [M+H]$^+$ 488.1433; found 488.1431.

(R)-4'-((7-hydroxy-6,8-dioxo-1,3,4,6,8,12a-hexahydro-12H-[1,4]oxazino[3,4-c]pyrido[2,1-f][1,2,4]triazin-12-yl)methyl)-[1,1'-biphenyl]-4-carboxylic acid ***(35)***. White solid, m.p. 283-

285°C, yield 85.6%. ^{1}H NMR (400 MHz, DMSO-d_6) δ 8.02 (d, *J* = 8.1 Hz, 2H, 2× Ph-H), 7.82 (d, *J* = 8.1 Hz, 2H, 2× Ph-H), 7.73 (d, *J* = 7.9 Hz, 2H, 2× Ph-H), 7.40 (d, *J* = 7.9 Hz, 2H, 2× Ph-H), 7.13 (d, *J* = 7.7 Hz, 1H, CH=CH), 5.81 (d, *J* = 7.6 Hz, 1H, CH=CH), 5.07 (s, 1H, CH), 4.41 – 4.19 (m, 3H, 3× CH), 3.99 – 3.78 (m, 2H, 2× CH), 3.68 (s, 2H, 2× CH), 3.17 (s, 1H, CH). ^{13}C NMR (100 MHz, DMSO-d_6) δ 171.53, 167.59, 153.39, 144.00, 139.29, 138.44, 135.32, 131.05, 130.45, 130.30, 127.46, 127.20, 116.24, 111.79, 65.97. HRMS (ESI): m/z calcd for $C_{24}H_{21}N_3O_6$ $[M+H]^+$ 448.1508; found 448.1508.

(R)-4'-((7-hydroxy-6,8-dioxo-1,3,4,6,8,12a-hexahydro-12H-[1,4]oxazino[3,4-c]pyrido[2,1-f][1,2,4]triazin-12-yl)methyl)-[1,1'-biphenyl]-4-carboxamide ***(36)***. Light orange solid, m.p. 236–239 °C, yield 54.4%. ^{1}H NMR (400 MHz, DMSO-d_6) δ 11.81 (s, 1H, OH), 8.03 (s, 1H, Ph-H), 7.97 (d, *J* = 8.0 Hz, 2H, 2 × Ph-H), 7.77 (d, *J* = 8.0 Hz, 2H, 2 × Ph-H), 7.72 (d, *J* = 7.8 Hz, 2H, NH_2), 7.39 (d, *J* = 7.1 Hz, 3H, 3 × Ph-H), 7.13 (d, *J* = 7.0 Hz, 1H, CH=CH), 5.81 (d, *J* = 7.5 Hz, 1H, CH=CH), 5.07 (s, 1H, CH), 4.42 – 4.21 (m, 3H, 3 × CH), 3.89 (dd, *J* = 40.0, 10.0 Hz, 2H, CH_2), 3.68 (d, *J* = 10.5 Hz, 1H, CH), 3.58 – 3.46 (m, 1H, CH), 3.25 (t, *J* = 10.9 Hz, 1H, CH). ^{13}C NMR (100 MHz, DMSO-d_6) δ 171.55, 167.96, 153.45, 142.45, 139.46, 138.43, 135.03, 133.69, 131.00, 128.67, 127.35, 126.83, 116.22, 111.78, 65.98, 60.23, 21.23, 14.55. HRMS (ESI): m/z calcd for $C_{24}H_{22}N_4O_5$ $[M+H]^+$ 447.1668; found 447.1669.

(R)-4'-((7-hydroxy-6,8-dioxo-1,3,4,6,8,12a-hexahydro-12H-[1,4]oxazino[3,4-c]pyrido[2,1-f][1,2,4]triazin-12-yl)methyl)-N-methyl-[1,1'-biphenyl]-4-carboxamide ***(37)***. Red brown solid, m.p.242-245 °C, yield 68.9%. ^{1}H NMR (400 MHz, DMSO-d_6) δ 8.57 – 8.49 (m, 1H, CONH), 7.93 (q, *J* = 8.6 Hz, 3H, 3 × Ph-H), 7.78 (d, *J* = 8.2 Hz, 2H, 2 × Ph-H), 7.72 (d, *J* = 8.2 Hz, 2H, 2 × Ph-H), 7.38 (d, *J* = 7.7 Hz, 2H, Ph-H, CH=CH), 5.77 (s, 1H, CH=CH), 5.04 (s, 1H, CH), 4.34 (s, 3H, 3 × CH), 3.99 – 3.78 (m, 2H, CH_2), 3.67 (s, 2H, 2 × CH), 3.17 (s, 1H, CH), 2.80 (d, *J* = 4.3 Hz, 3H, $CONCH_3$). ^{13}C NMR (100MHz, DMSO-d_6) δ 166.69, 142.29, 139.36, 134.26, 133.90, 130.89, 128.25, 128.22, 127.32, 127.16, 126.89, 66.00, 26.74. HRMS (ESI): m/z calcd for $C_{25}H_{24}N_4O_5$ $[M+H]^+$ 461.1825; found 461.1828.

(R)-7-hydroxy-12-((4'-(methylsulfonyl)-[1,1'-biphenyl]-4-yl)methyl)-3,4,12,12a-tetrahydro-1H-[1,4]oxazino[3,4-c]pyrido[2,1-f][1,2,4]triazine-6,8-dione ***(38)***. Light orange solid, m.p. 215–218 °C, yield 43%. ^{1}H NMR (400 MHz, DMSO-d_6) δ 11.75 (s, 1H, OH), 8.02 – 7.93 (m, 4H, 4 × Ph-H), 7.74 (d, *J* = 7.8 Hz, 2H, 2 × Ph-H), 7.43 (d, *J* = 7.9 Hz, 2H, 2 × Ph-H), 7.14 (d, *J* = 7.6 Hz, 1H, CH=CH), 5.81 (d, *J* = 7.6 Hz, 1H, CH=CH), 5.07 (d, *J* = 8.3 Hz, 1H, CH), 4.43 – 4.24 (m, 3H, 3 × CH), 3.89 (dd, *J* = 41.8, 11.1 Hz, 2H, CH_2), 3.67 (t, *J* = 10.5 Hz, 1H, CH), 3.52 (t, *J* = 10.5 Hz, 1H, CH), 3.29 (d, *J* = 3.7 Hz, 1H, CH), 3.25 (s, 3H, CH_3). ^{13}C NMR (100 MHz, DMSO-d_6) δ 171.52, 153.40, 144.83, 140.17, 138.64, 138.41, 135.81, 131.12, 128.13, 127.99, 127.70, 116.24, 111.81, 65.98, 55.40, 44.05. HRMS (ESI): m/z calcd for $C_{24}H_{23}N_3O_6S$ $[M+H]^+$ 482.1386; found 482.1386.

(R)-N-cyclopentyl-4'-((7-hydroxy-6,8-dioxo-1,3,4,6,8,12a-hexahydro-12H-[1,4]oxazino[3,4-c]pyrido[2,1-f][1,2,4]triazin-12-yl)methyl)-[1,1'-biphenyl]-4-carboxamide ***(39)***. Yellow solid, m.p. 165-167°C, yield 54.3%. ^{1}H NMR (400 MHz, DMSO-d_6) δ 8.36 (d, *J* = 7.3 Hz, 1H, CONH), 7.94 (d, *J* = 8.1 Hz, 2H, 2 × Ph-H), 7.77 (d, *J* = 8.1 Hz, 2H, 2 × Ph-H), 7.72 (d, *J* = 8.0 Hz, 2H, 2 × Ph-H), 7.39 (d, *J* = 7.9 Hz, 2H, 2 × Ph-H), 7.13 (d, *J* = 7.6 Hz, 1H, CH=CH), 5.81 (d, *J* = 7.6

Hz, 1H, CH=CH), 5.07 (s, 1H, CH), 4.41 – 4.27 (m, 3H, 3 × CH), 4.28 – 4.22 (m, 1H, CH), 3.97 – 3.81 (m, 2H, CH_2), 3.68 (s, 1H, CH), 3.52 (s, 1H, CH), 3.27 – 3.21 (m, 1H, CH), 1.94 – 1.86 (m, 2H, CH_2), 1.74 – 1.67 (m, 2H, CH_2), 1.58 – 1.51 (m, 4H, 2 × CH_2).^{13}C NMR (100 MHz, DMSO-d_6) δ 171.52, 165.92, 153.41, 142.15, 139.46, 138.47, 134.99, 134.17, 131.03, 128.48, 127.32, 126.73, 116.23, 111.77, 65.98, 51.42, 32.60, 24.14. HRMS (ESI): m/z calcd for $C_{29}H_{30}N_4O_5$ $[M+H]^+$ 515.2294; found 515.2297.

(R)-4'-((7-hydroxy-6,8-dioxo-1,3,4,6,8,12a-hexahydro-12H-[1,4]oxazino[3,4-c]pyrido[2,1-f][1,2,4]triazin-12-yl)methyl)-N-phenyl-[1,1'-biphenyl]-4-carboxamide ***(40)***. White solid, m.p. 290-293°C, yield 45.7%. ^{1}H NMR (400 MHz, DMSO-d_6) δ 10.32 (s, 1H, CONH), 8.07 (d, J = 8.1 Hz, 2H, 2 × Ph-H), 7.86 (d, J = 8.1 Hz, 2H, 2 × Ph-H), 7.81 (d, J = 8.0 Hz, 2H, 2 × Ph-H), 7.76 (d, J = 7.9 Hz, 2H, 2 × Ph-H), 7.41 (d, J = 7.9 Hz, 2H, 2 × Ph-H), 7.37 (t, J = 7.7 Hz, 2H, 2 × Ph-H), 7.13 (q, J = 7.8 Hz, 2H, Ph-H, CH=CH), 5.82 (d, J = 7.6 Hz, 1H, CH=CH), 5.07 (s, 1H, CH), 4.35 (s, 3H, 3 × CH), 3.98 – 3.81 (m, 2H, CH_2), 3.68 (s, 2H, 2 × CH), 3.26 (d, J = 3.4 Hz, 1H, CH). ^{13}C NMR (100 MHz, DMSO-d_6) δ 171.54, 165.53, 153.42, 142.80, 139.63, 139.32, 138.47, 135.18, 134.26, 131.06, 129.10, 128.88, 127.41, 126.98. HRMS (ESI): m/z calcd for $C_{30}H_{26}N_4O_5$ $[M+H]^+$ 523.1981; found 523.1982.

^{1}H and ^{13}C NMR spectra of compound **1**

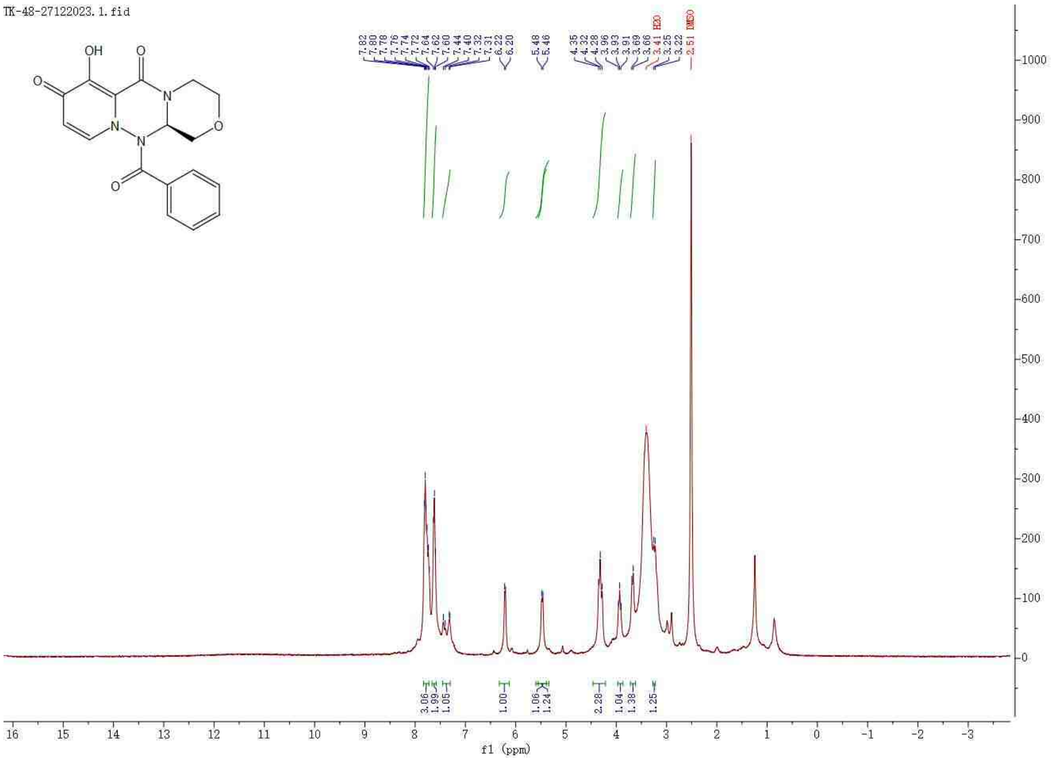

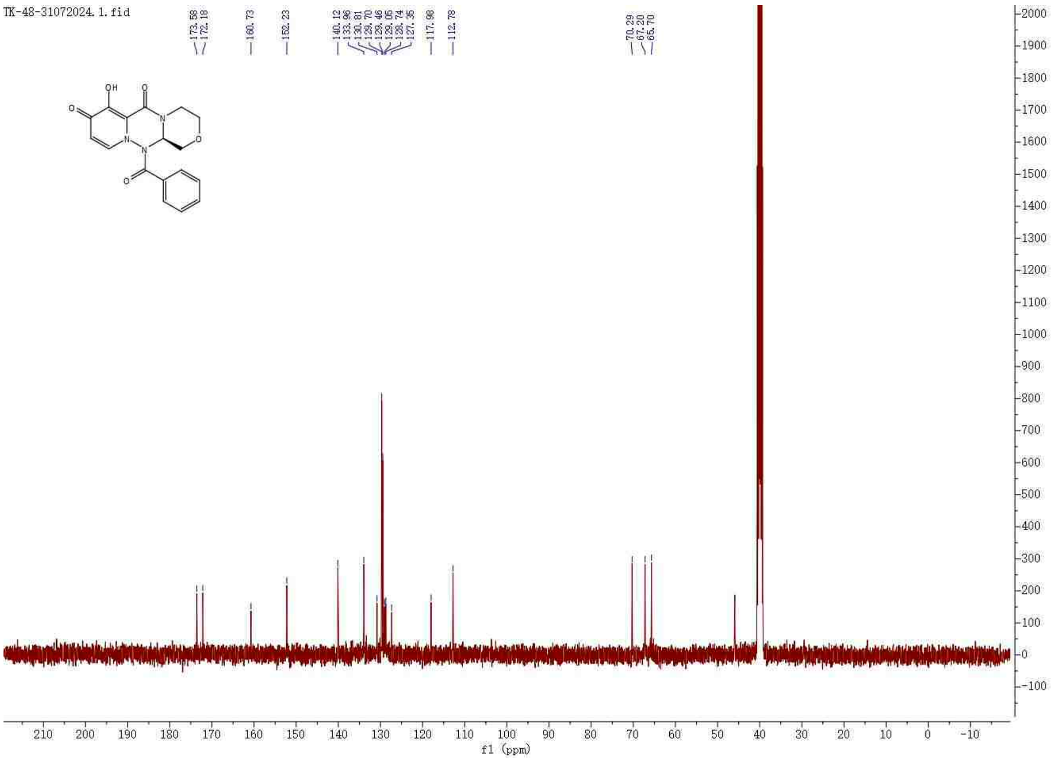

^{1}H and ^{13}C NMR spectra of compound **2**

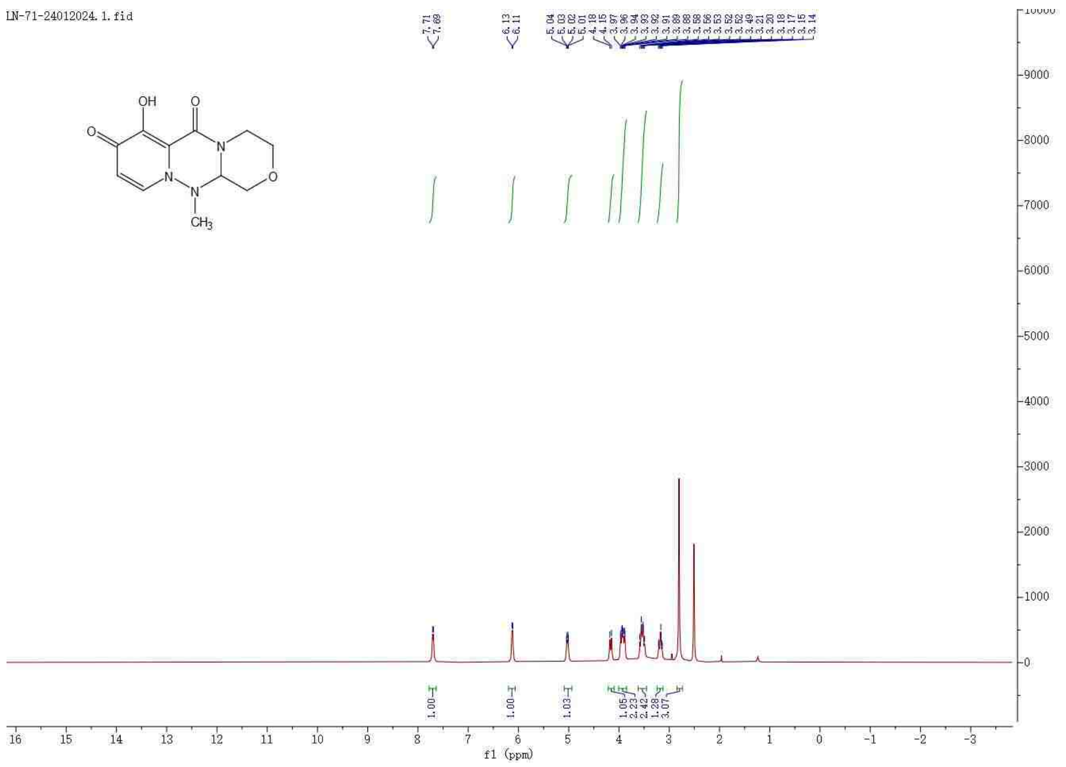

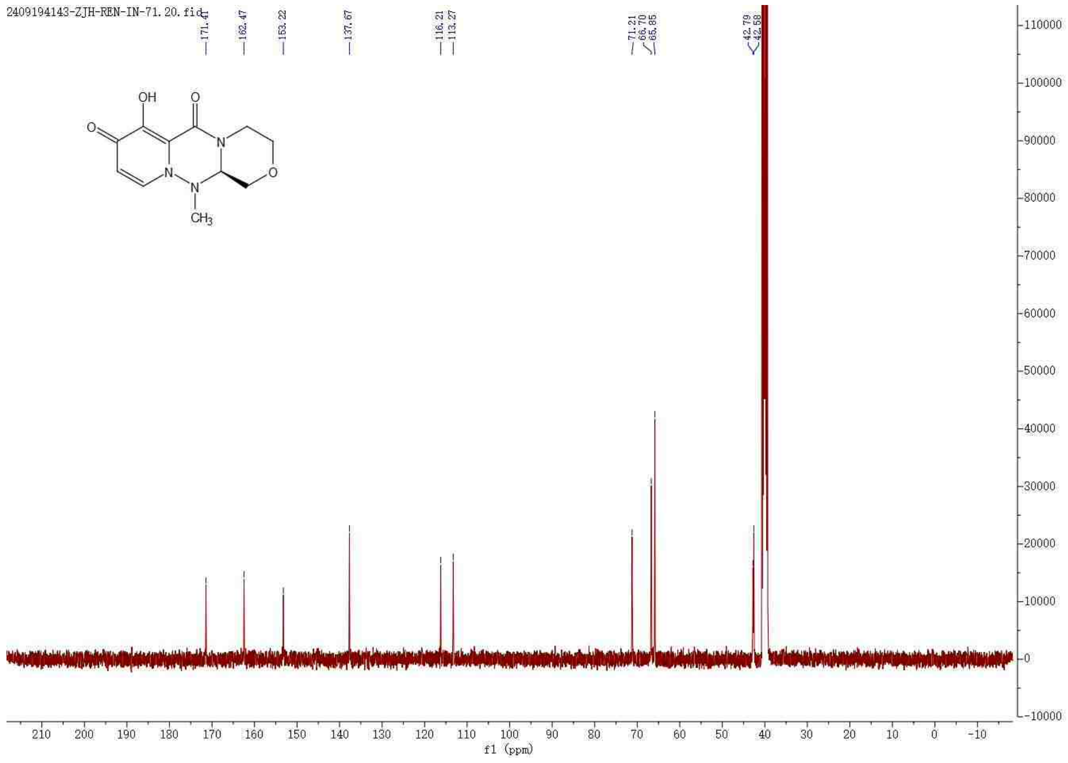

^{1}H and ^{13}C NMR spectra of compound **3**

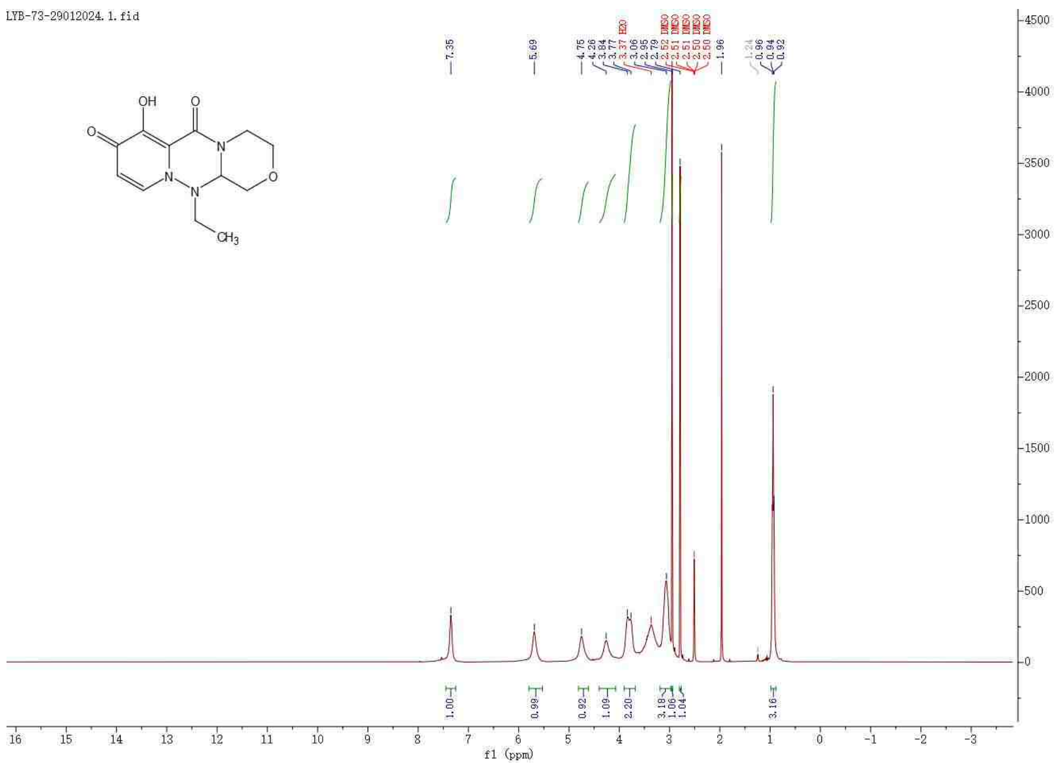

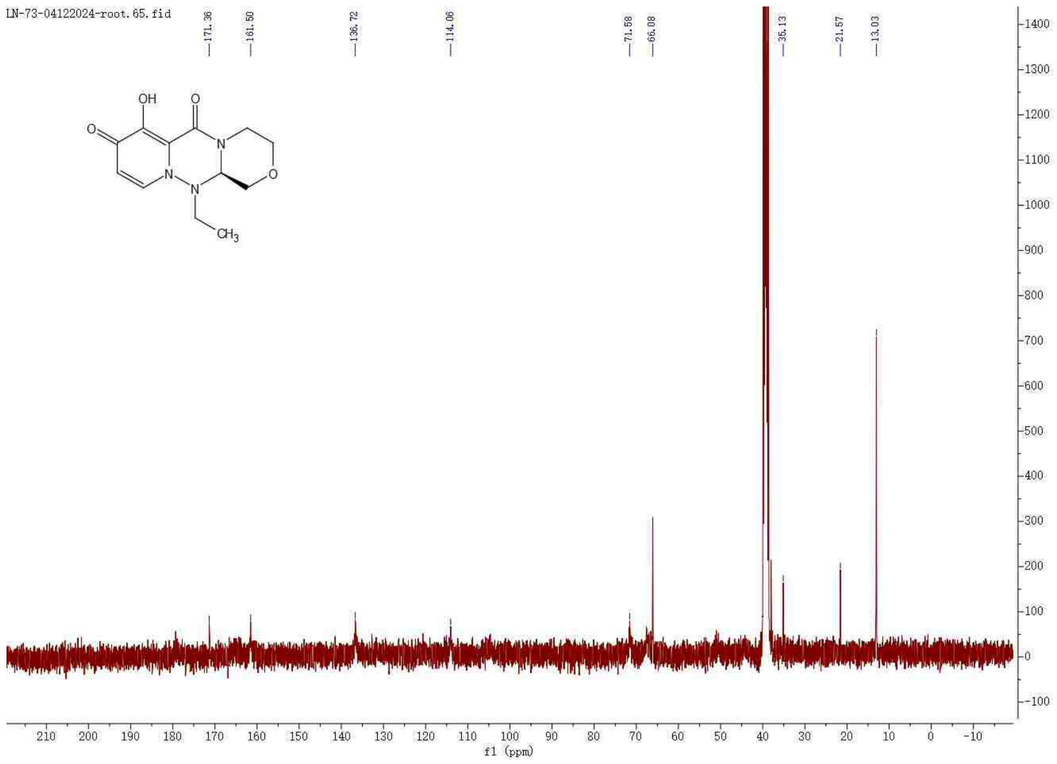

^{1}H and ^{13}C NMR spectra of compound **4**

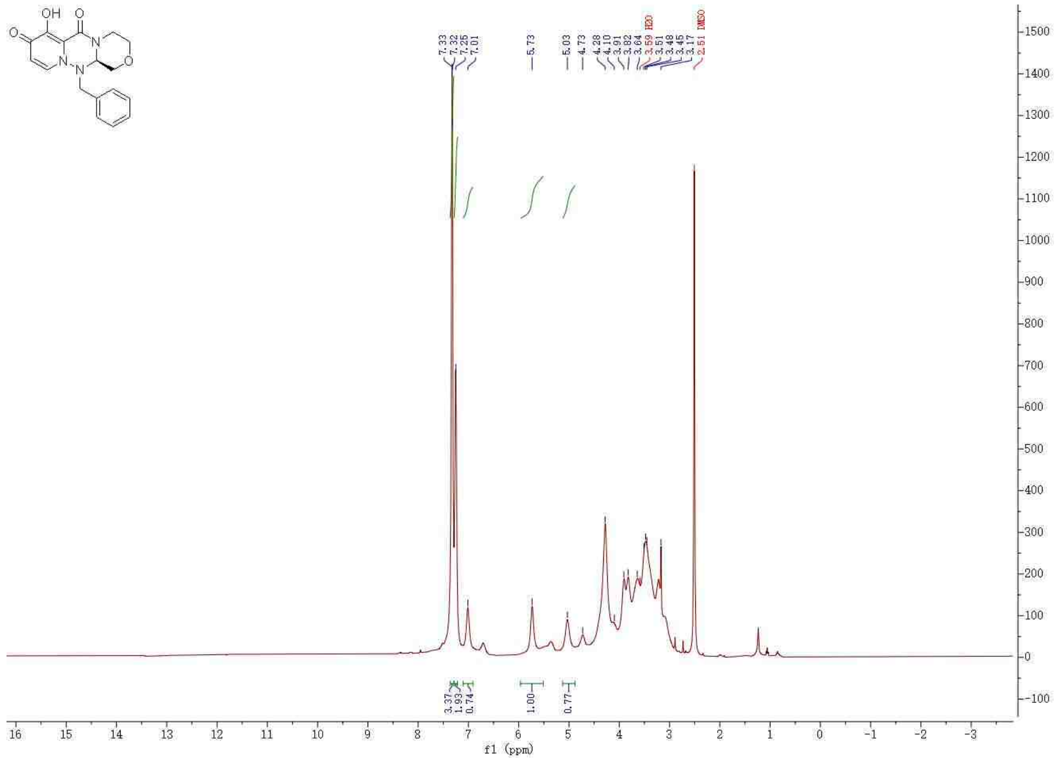

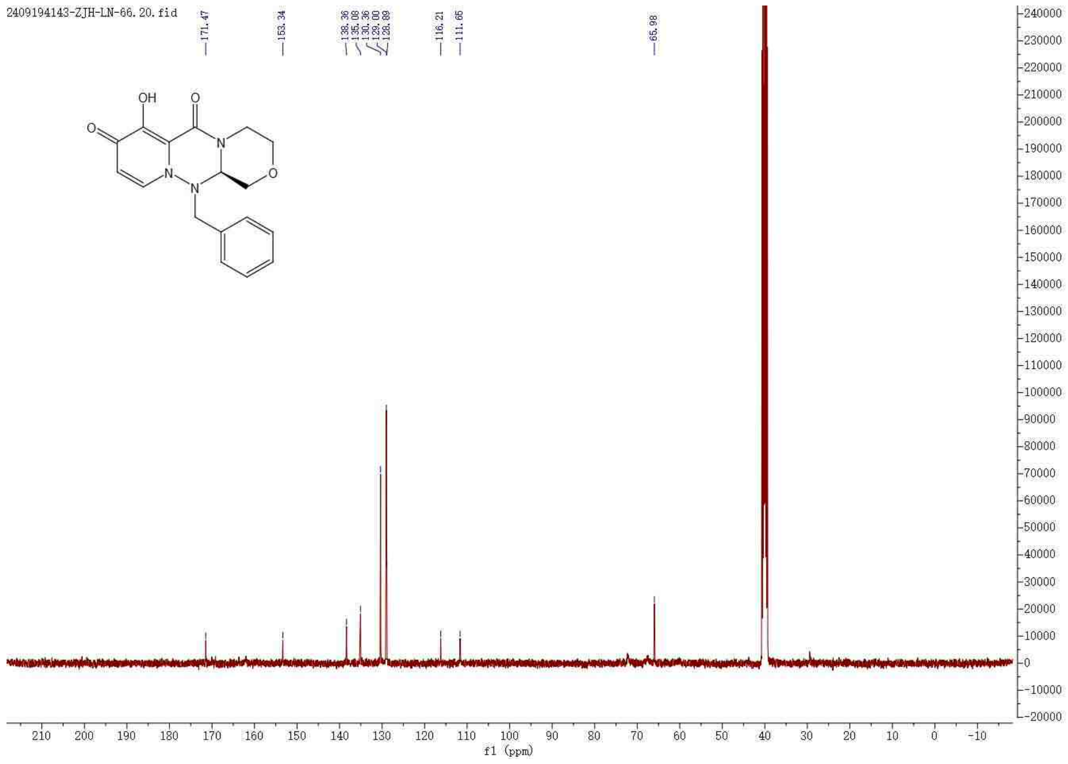

^{1}H and ^{13}C NMR spectra of compound **5**

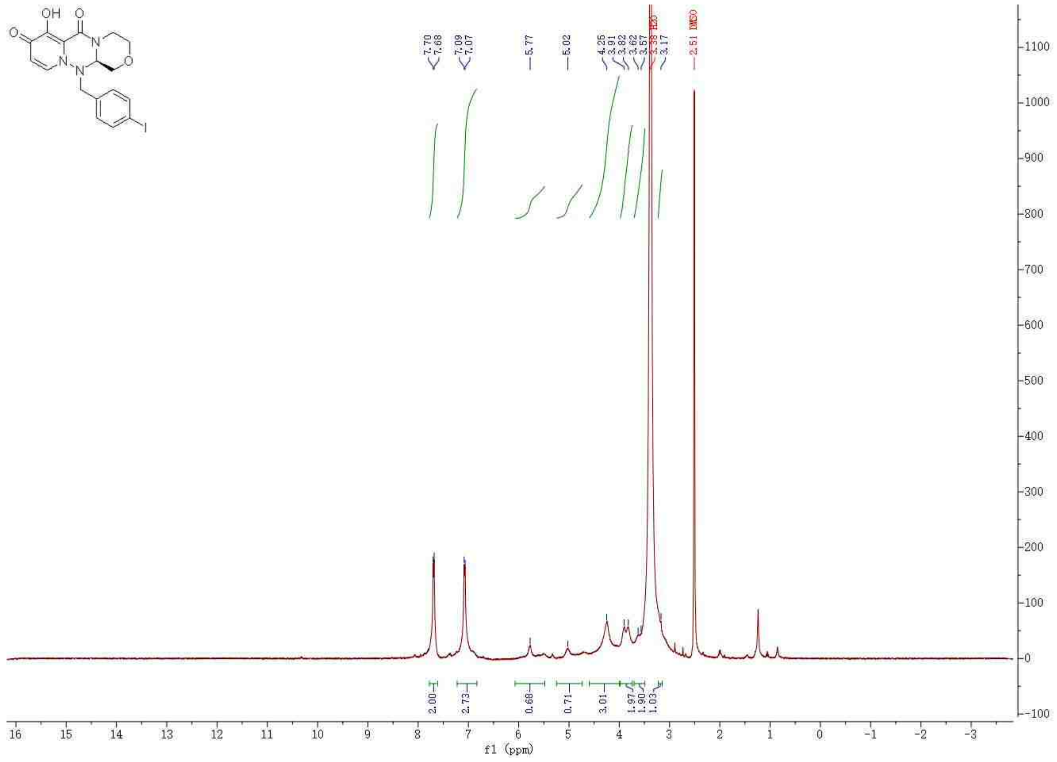

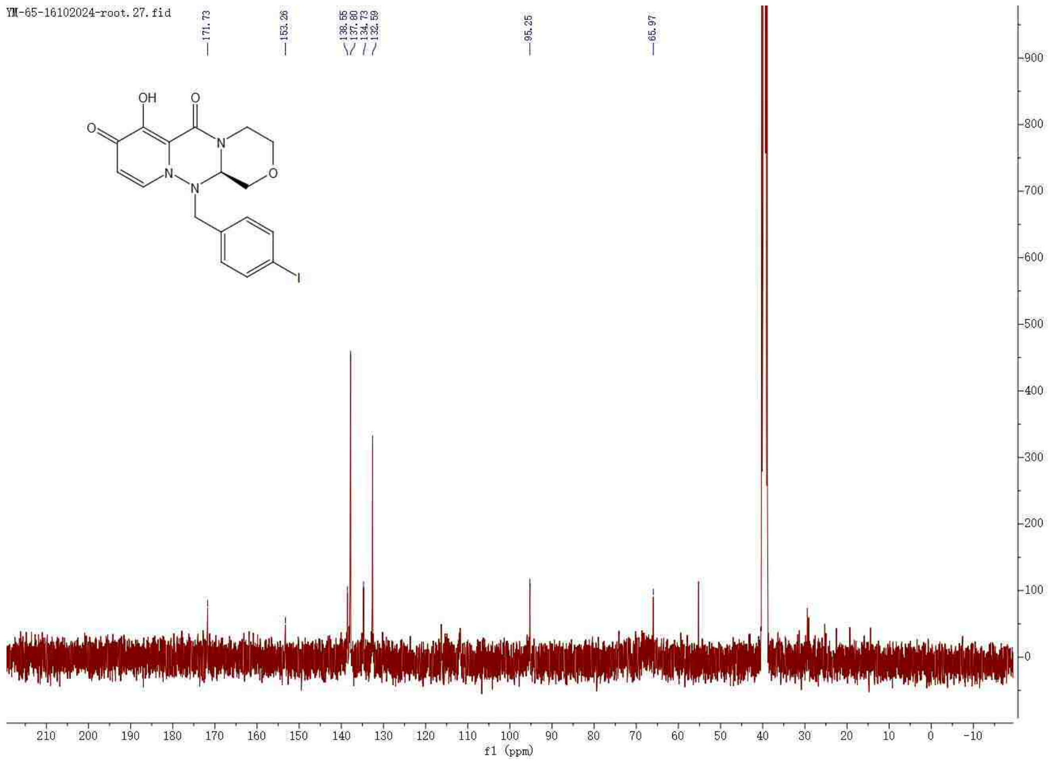

^1H and ^{13}C NMR spectra of compound **6**

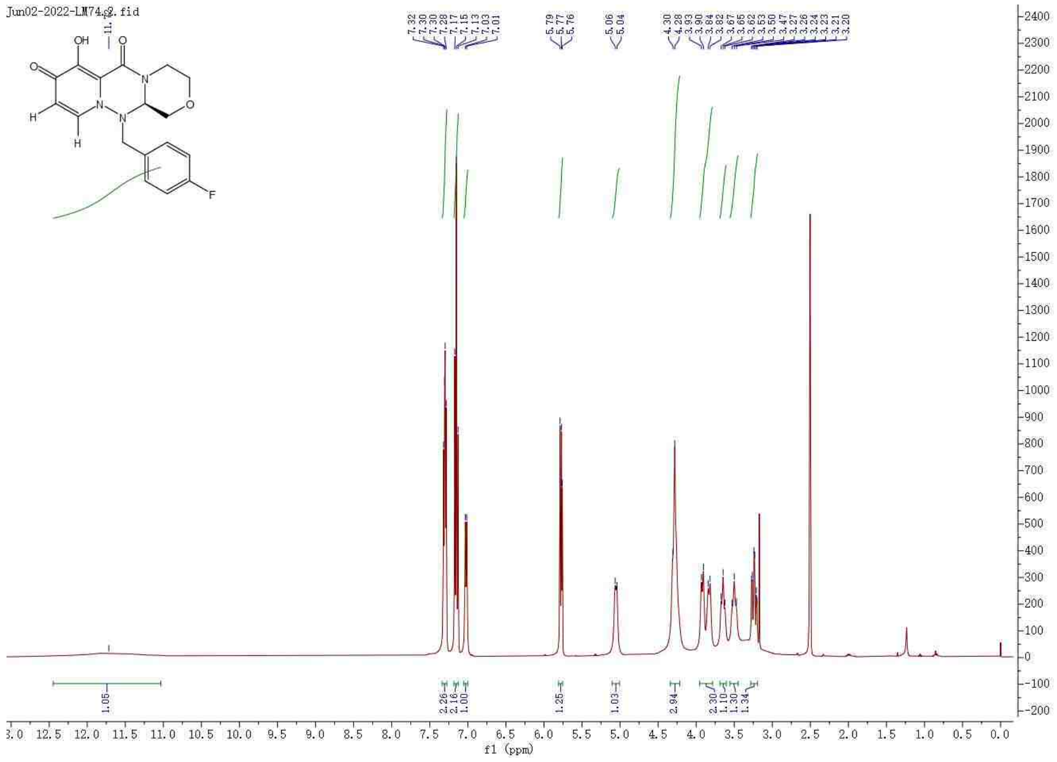

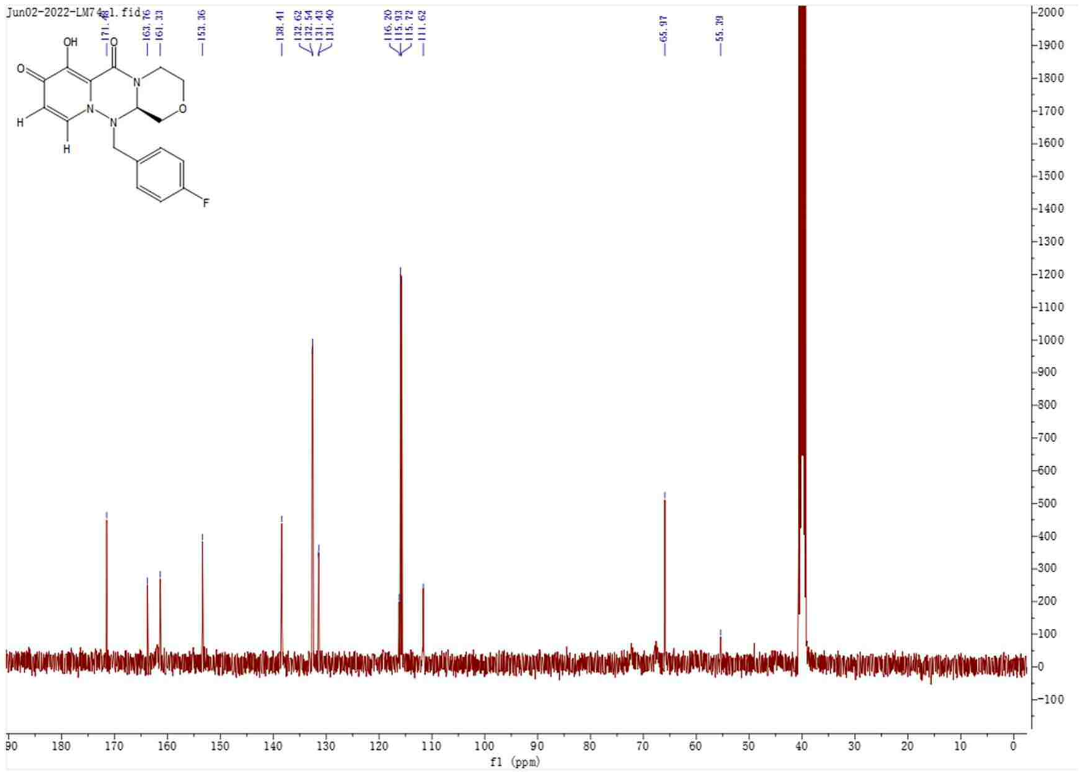

^{1}H and ^{13}C NMR spectra of compound **7**

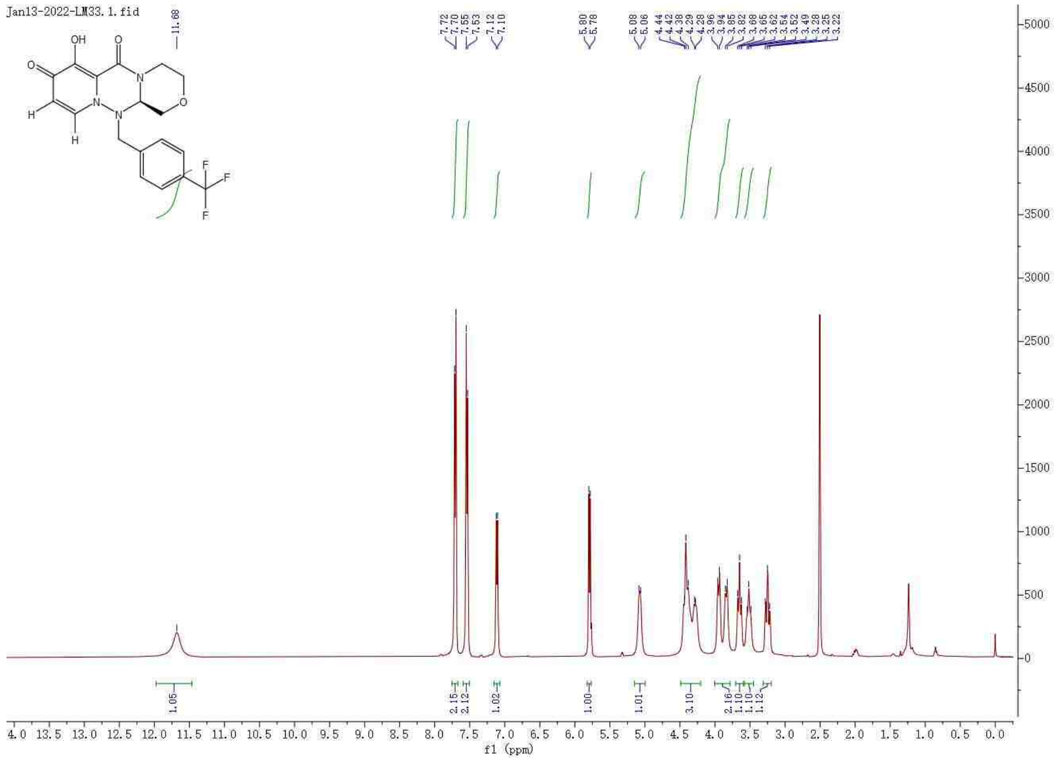

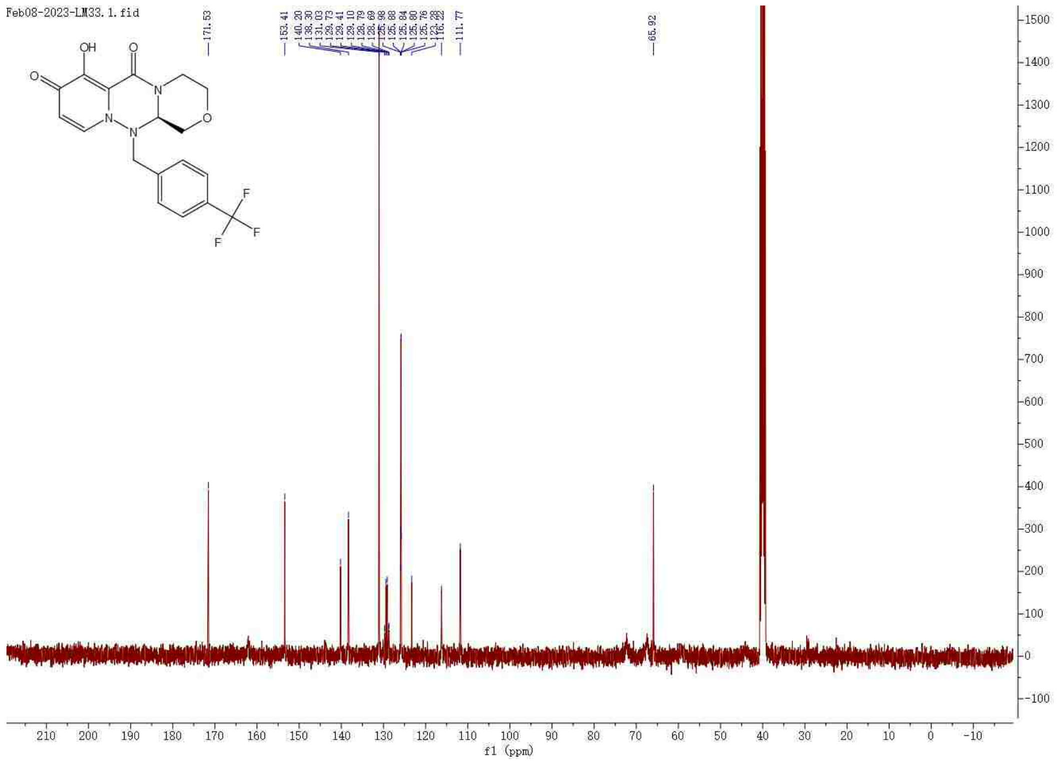

^{1}H and ^{13}C NMR spectra of compound **8**

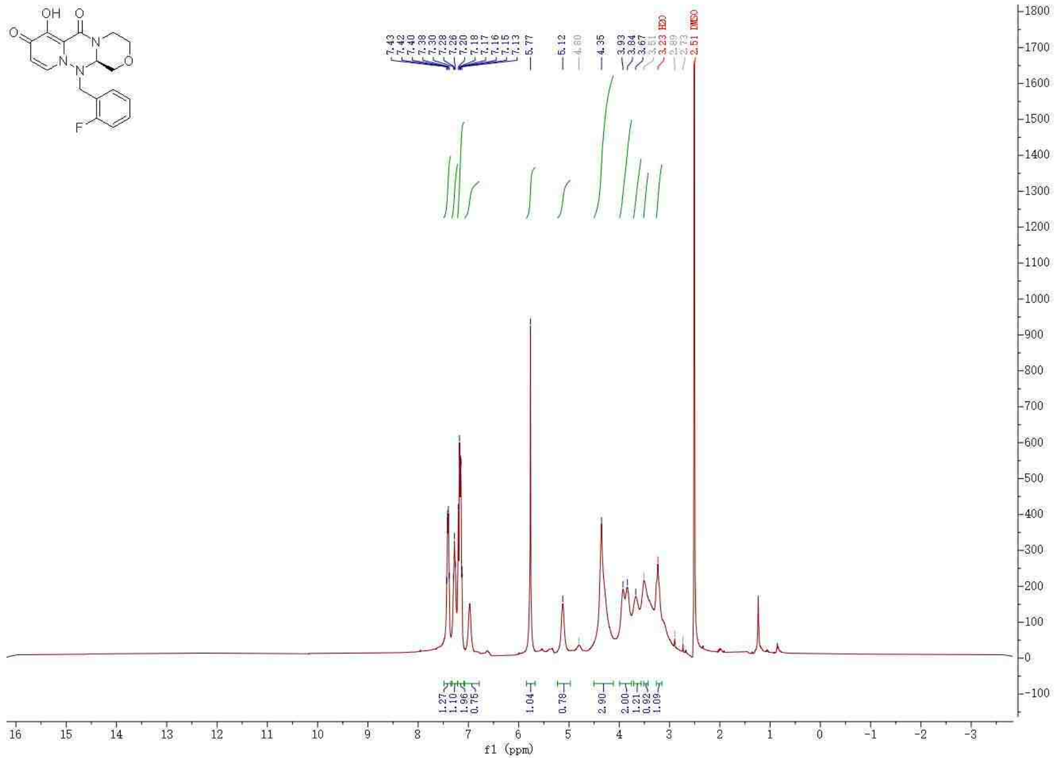

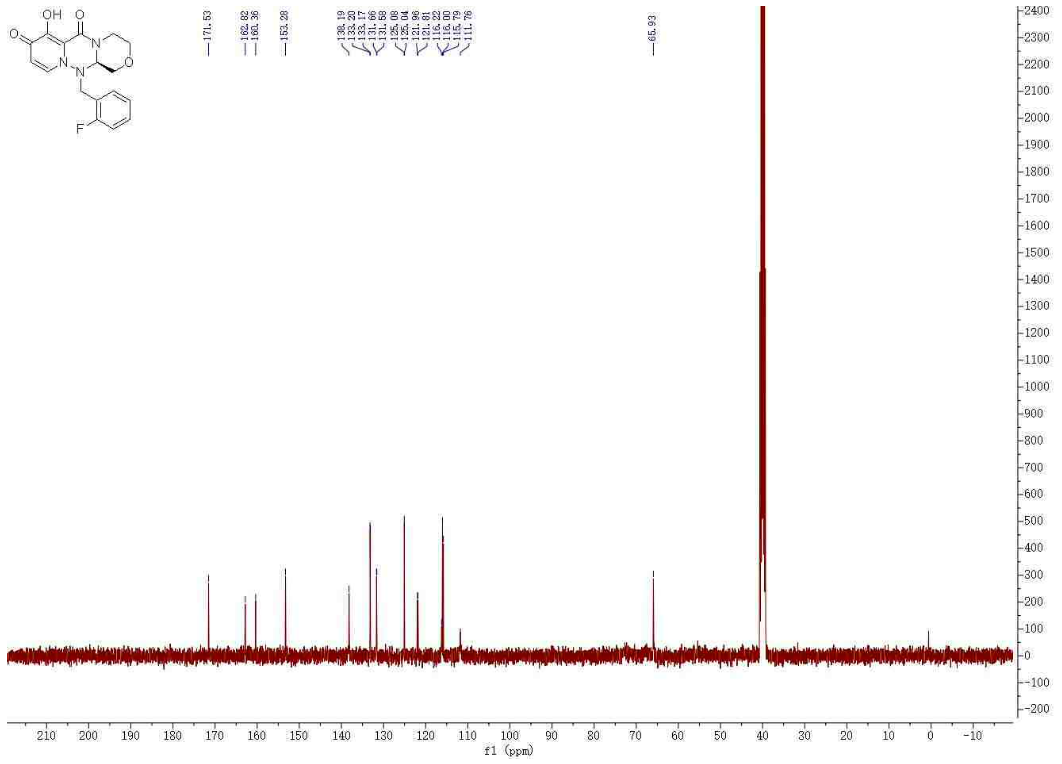

^{1}H and ^{13}C NMR spectra of compound **9**

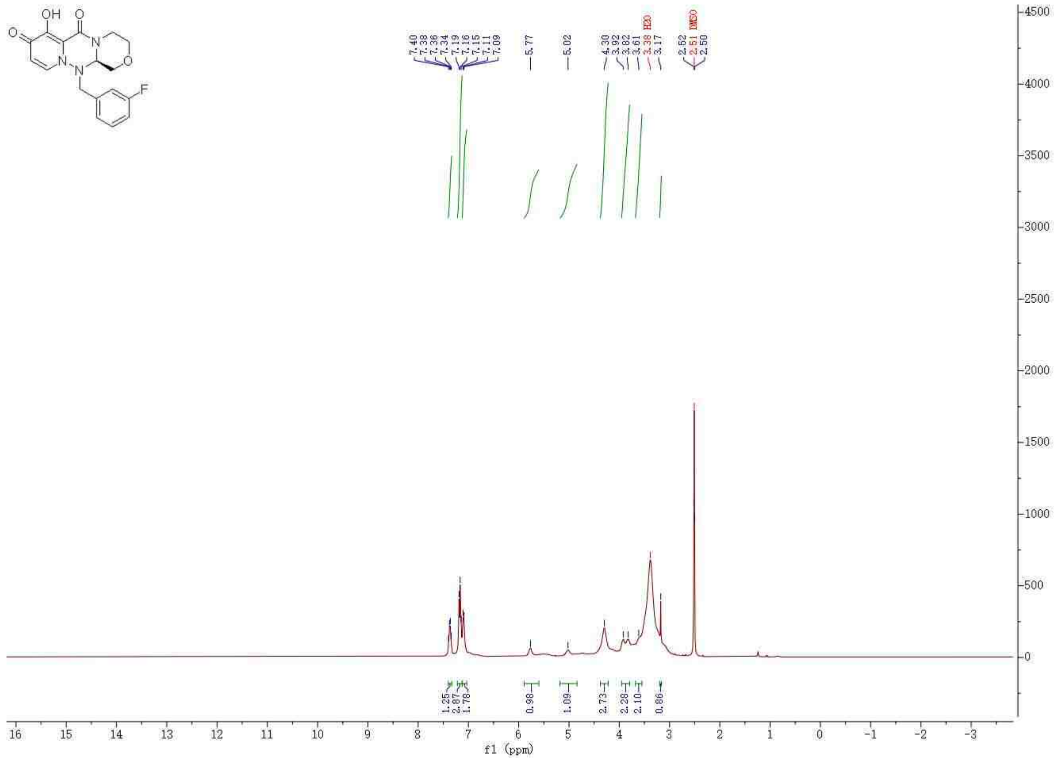

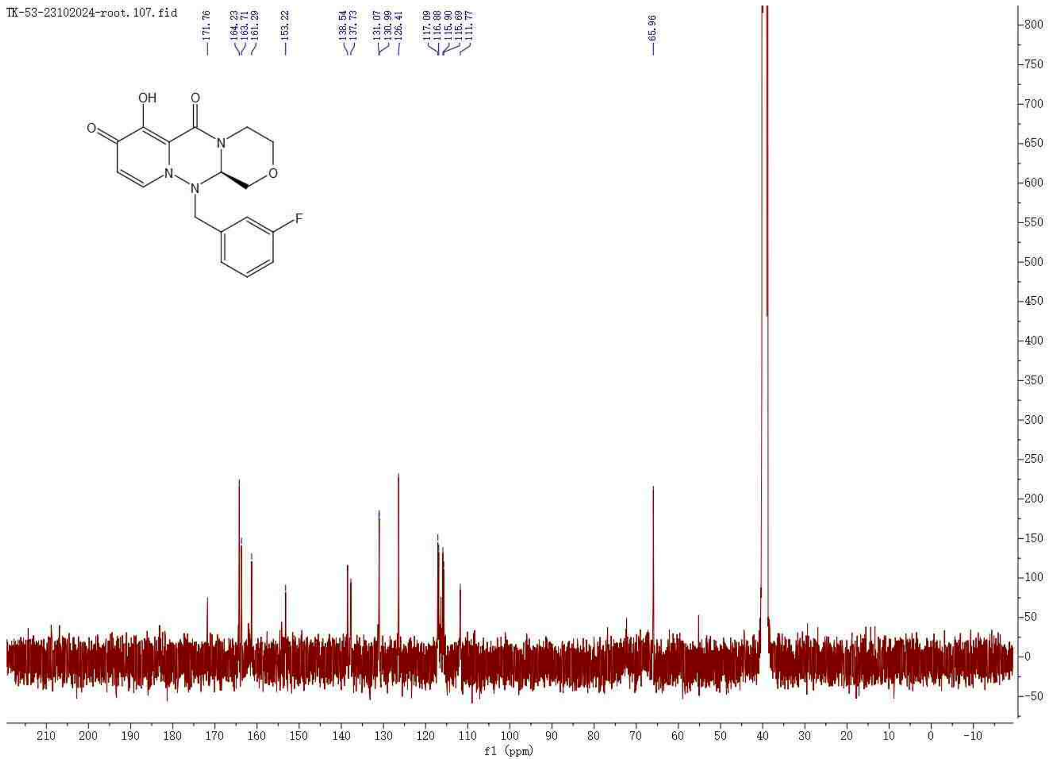

^{1}H and ^{13}C NMR spectra of compound **10**

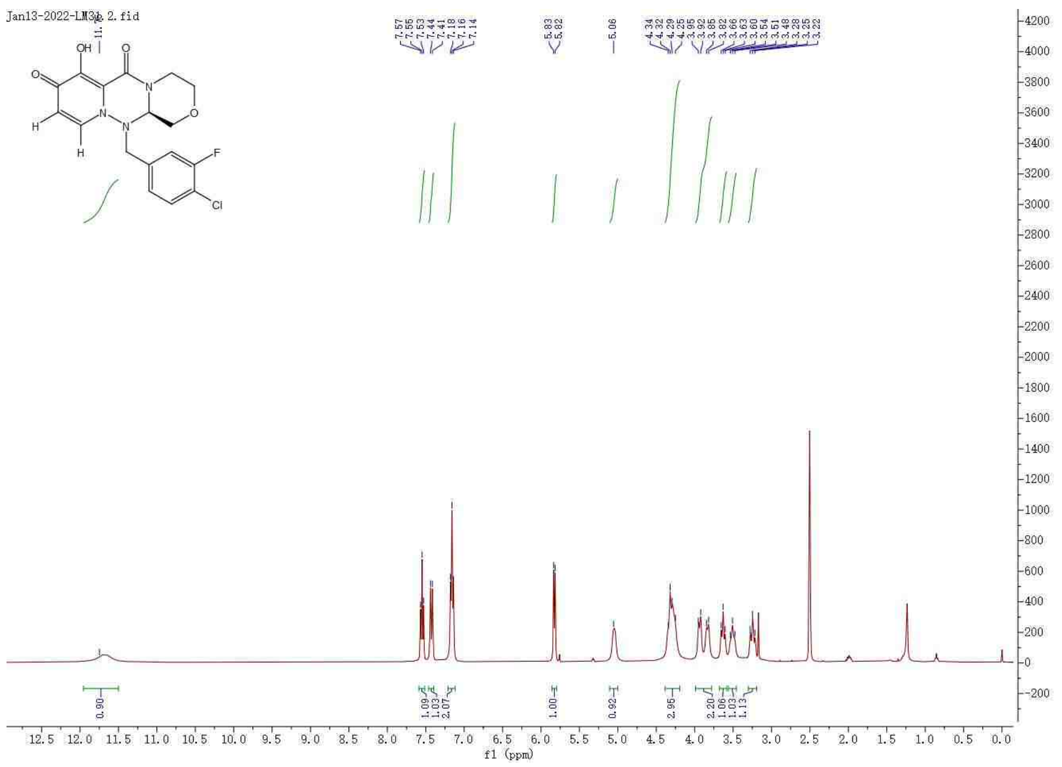

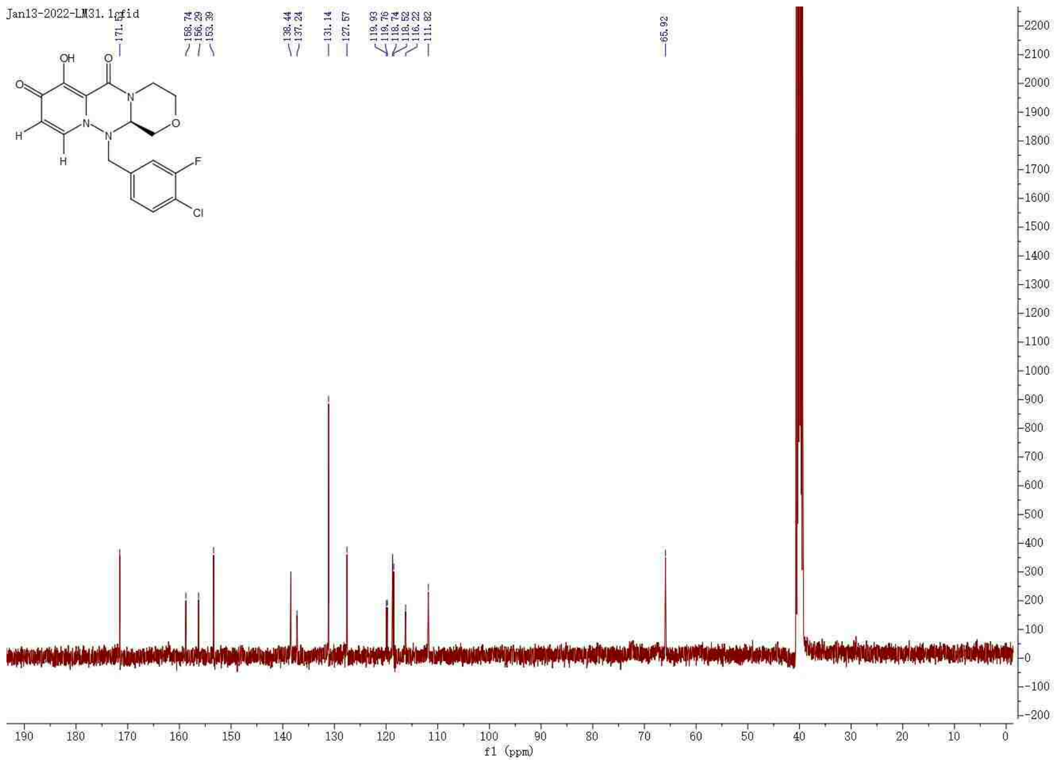

^{1}H and ^{13}C NMR spectra of compound **11**

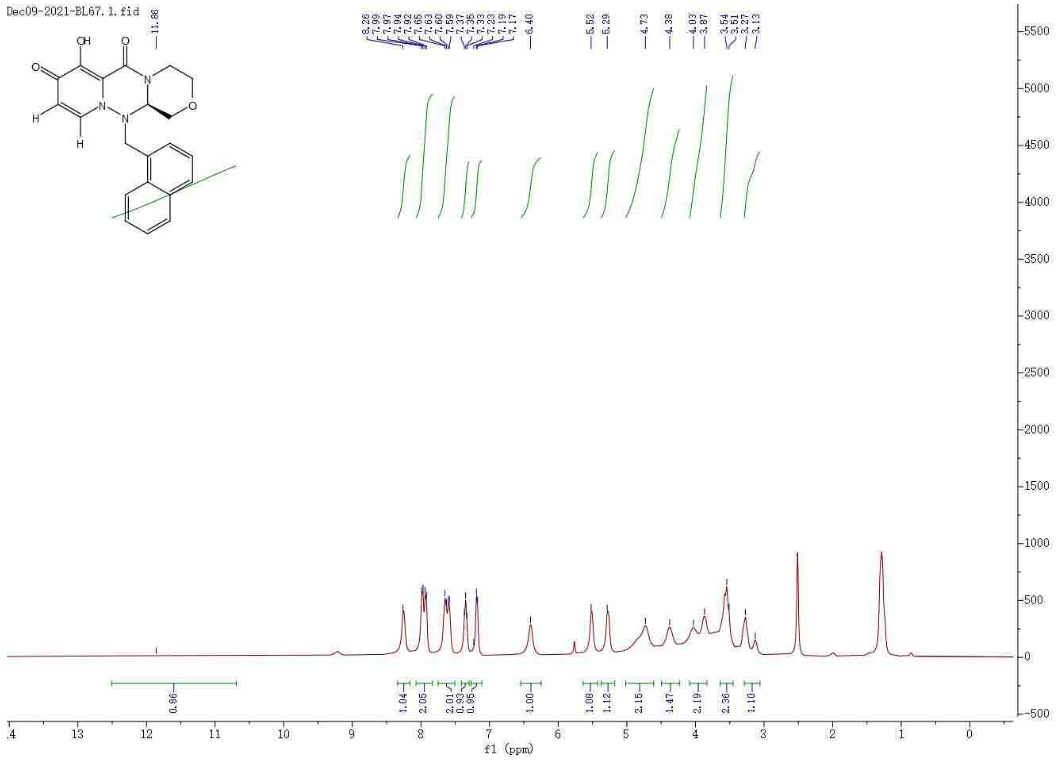

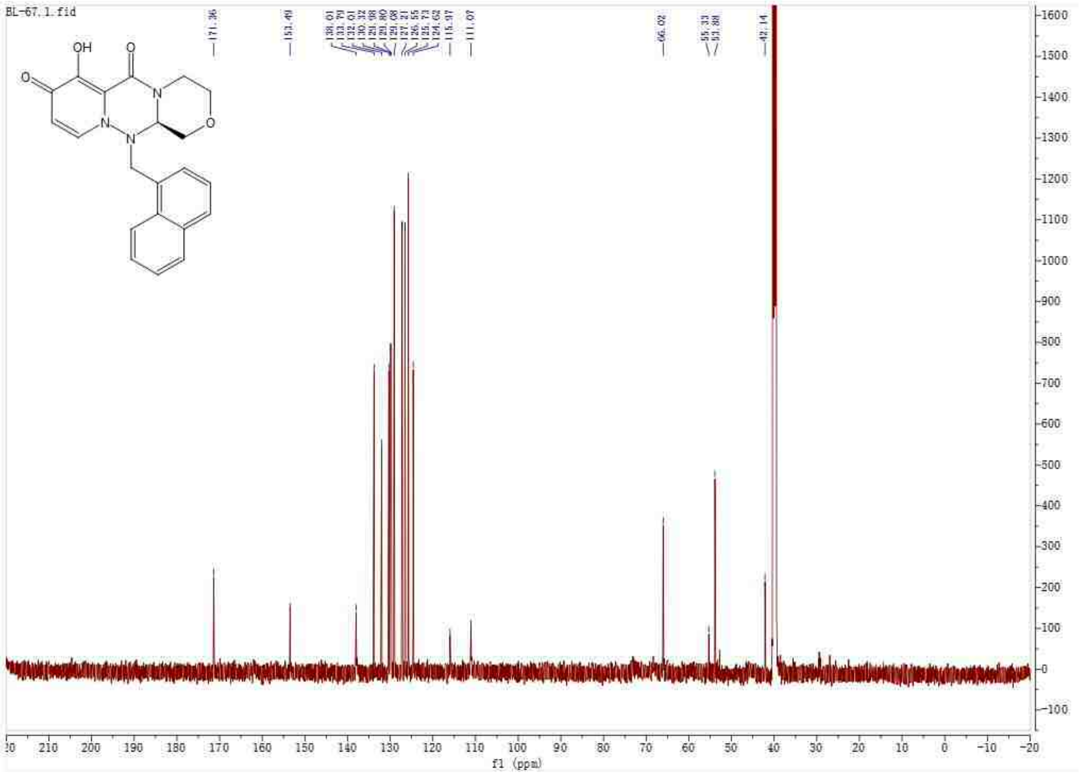

^{1}H and ^{13}C NMR spectra of compound **12**

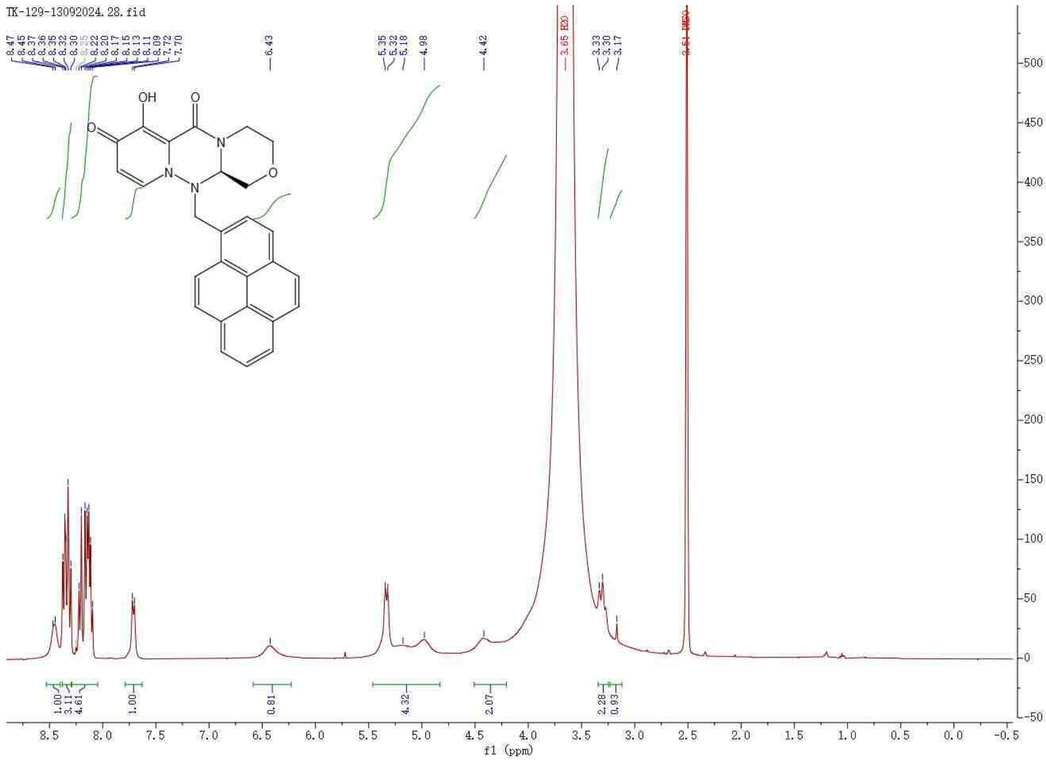

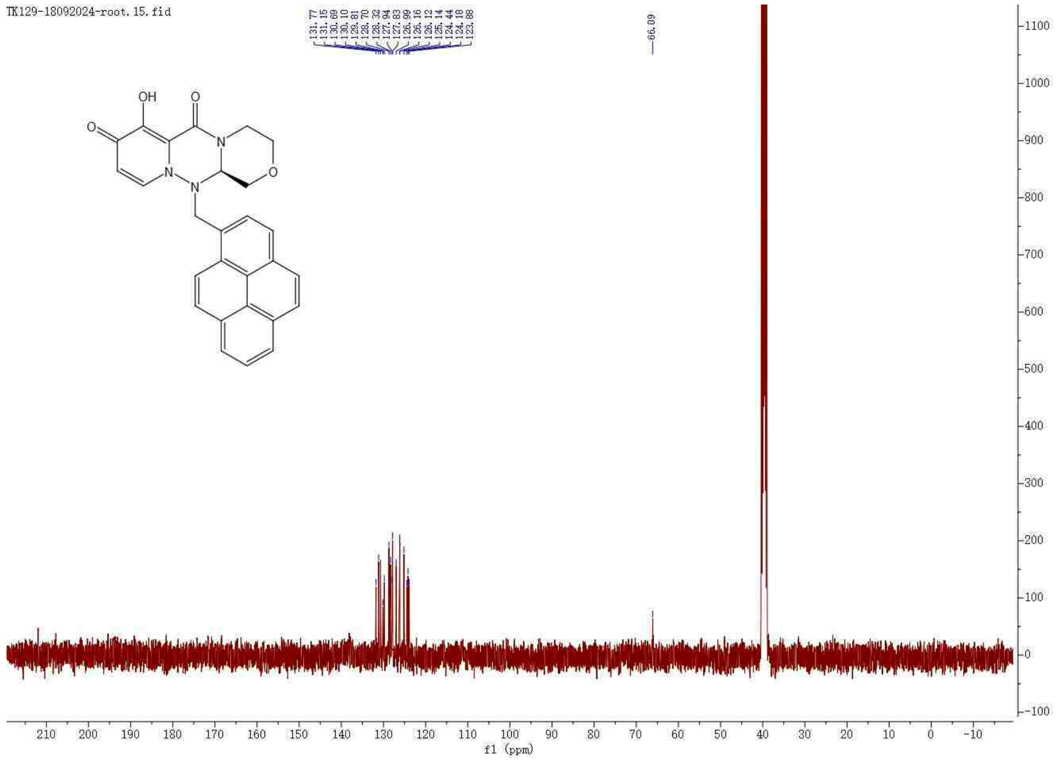

^{1}H and ^{13}C NMR spectra of compound **13**

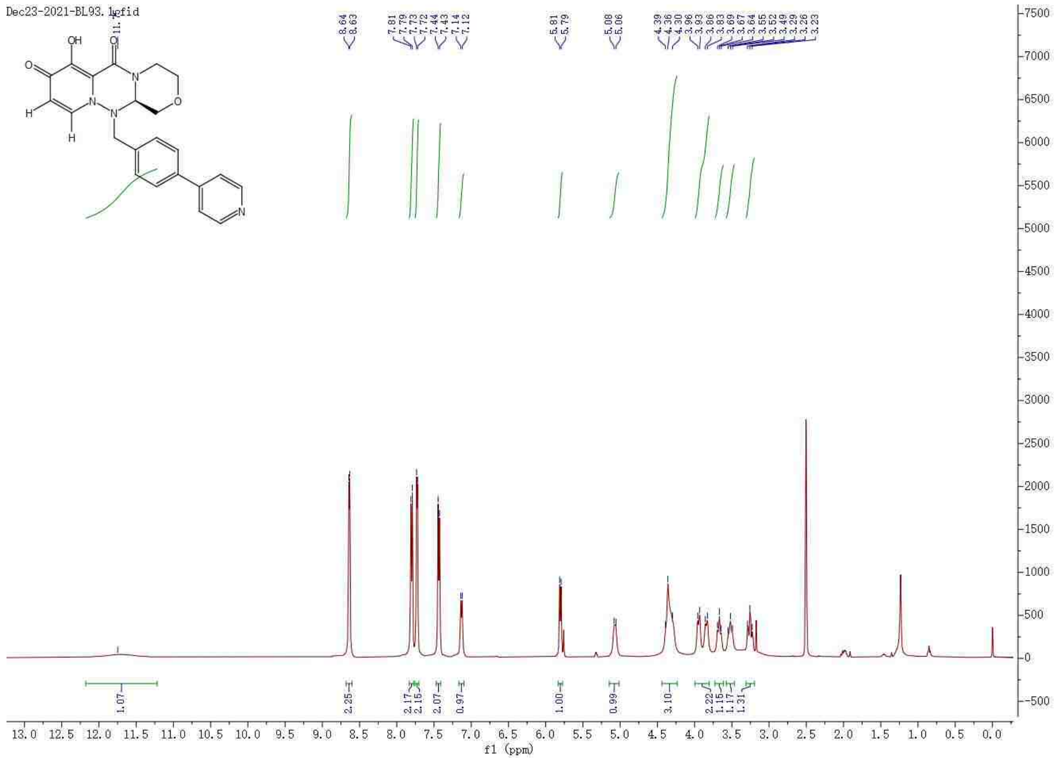

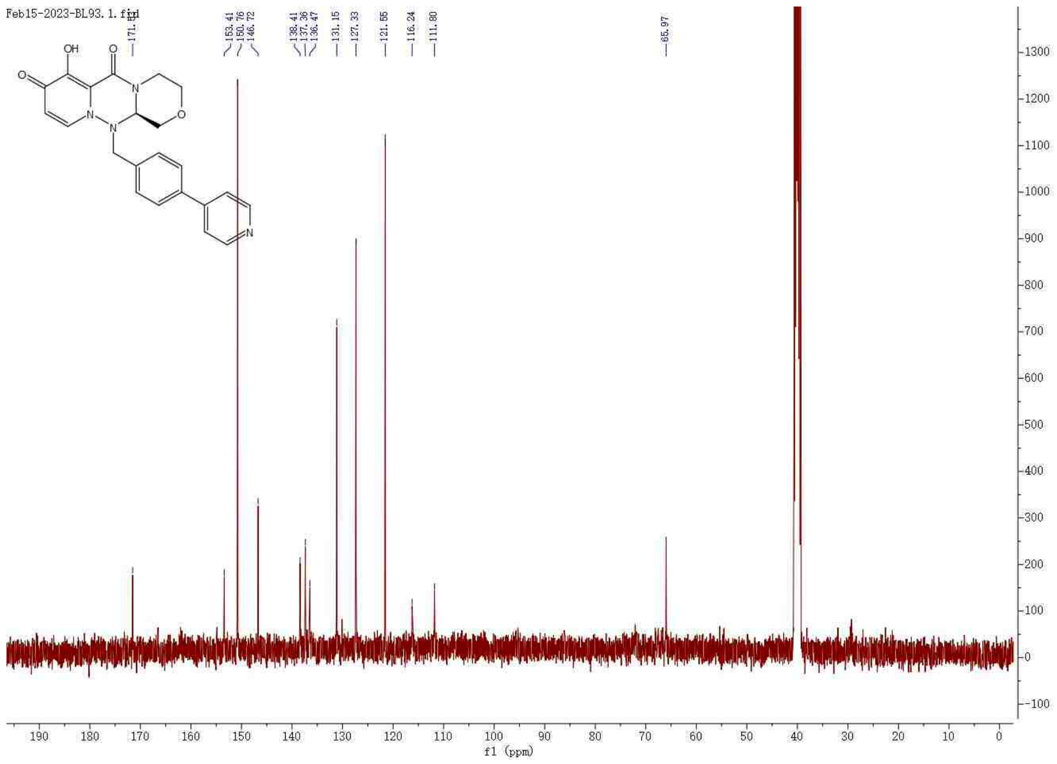

^{1}H and ^{13}C NMR spectra of compound **14**

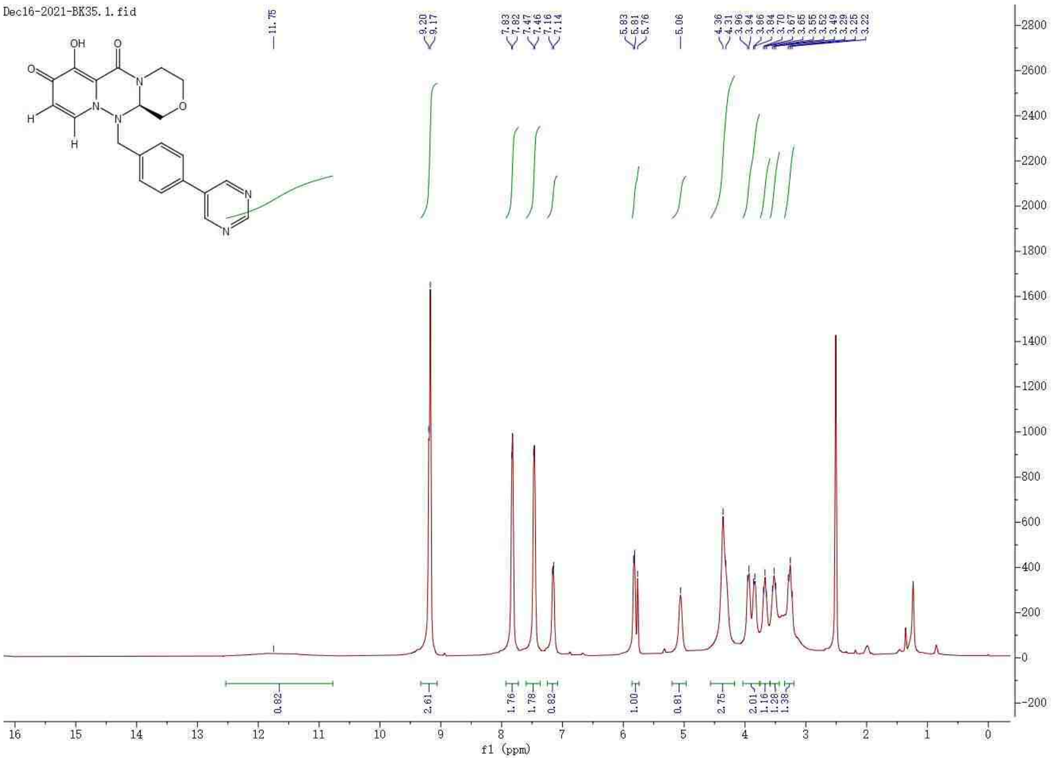

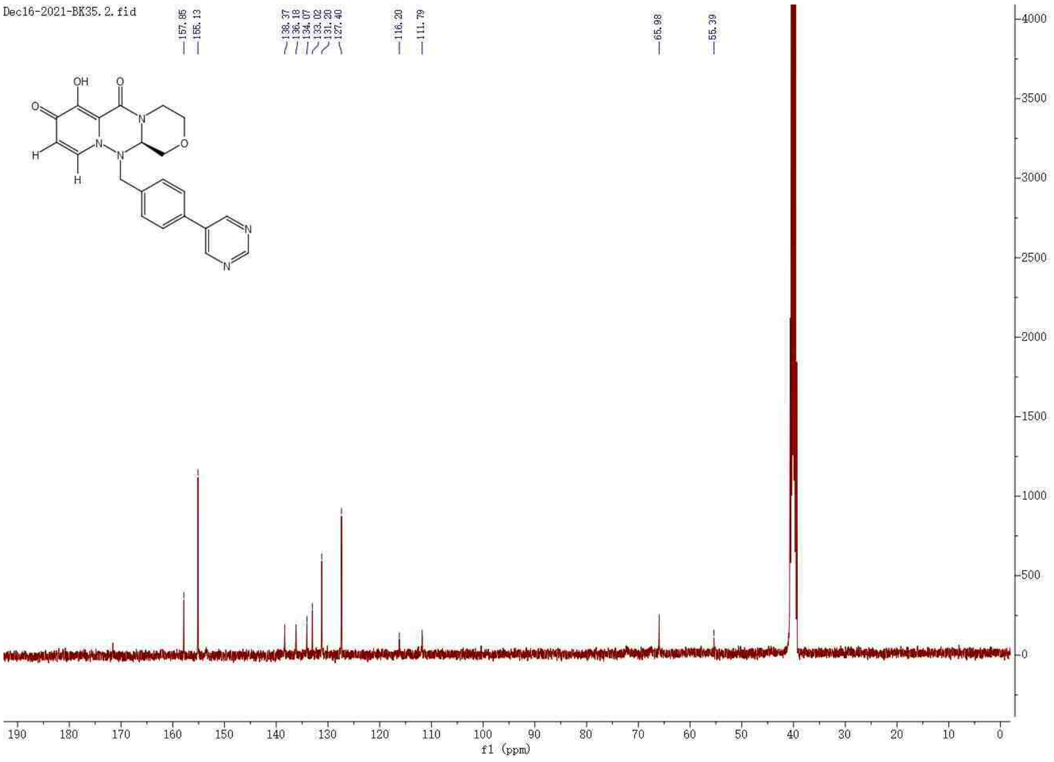

^{1}H and ^{13}C NMR spectra of compound **15**

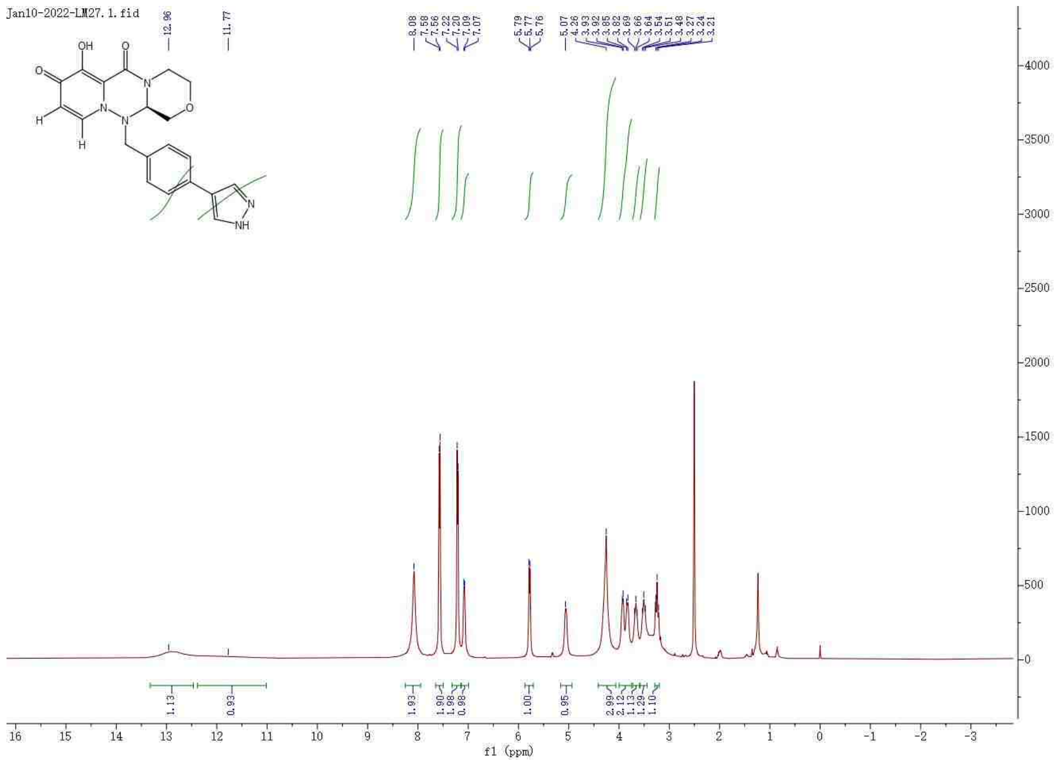

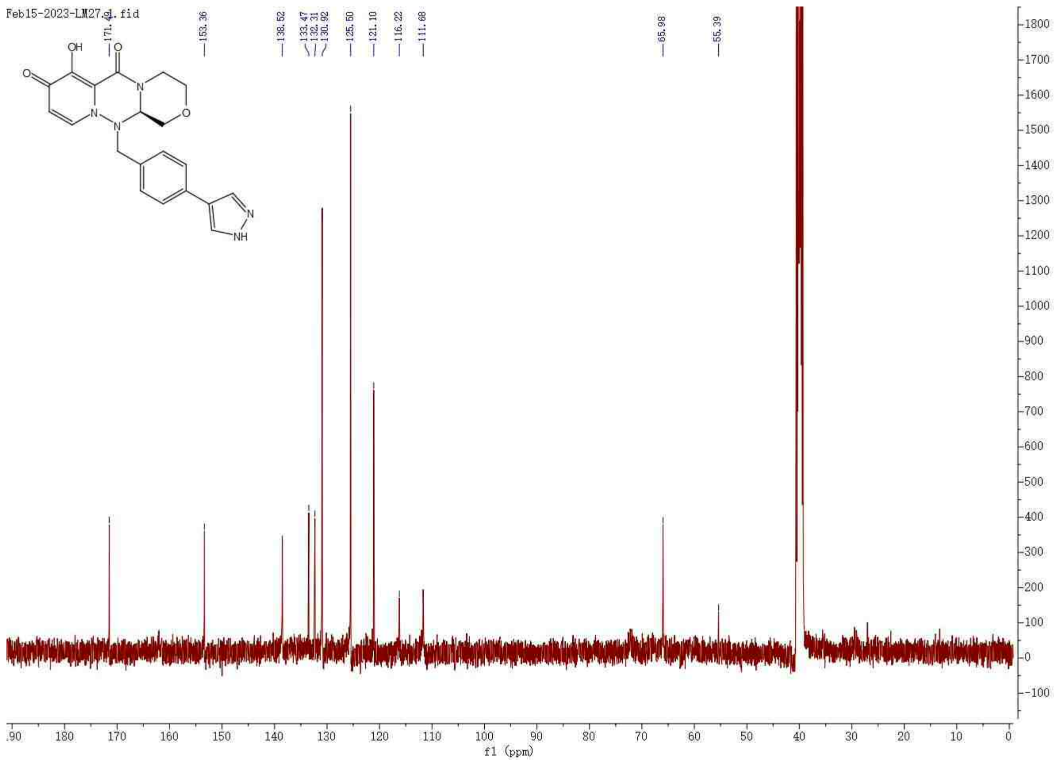

^{1}H and ^{13}C NMR spectra of compound **16**

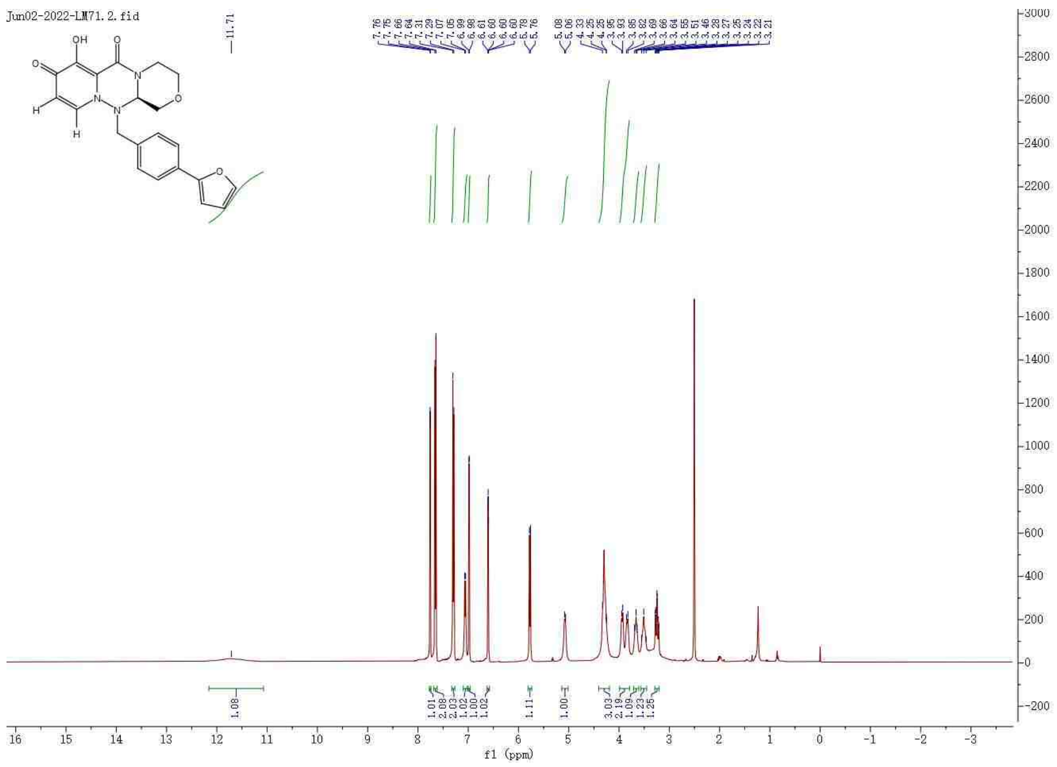

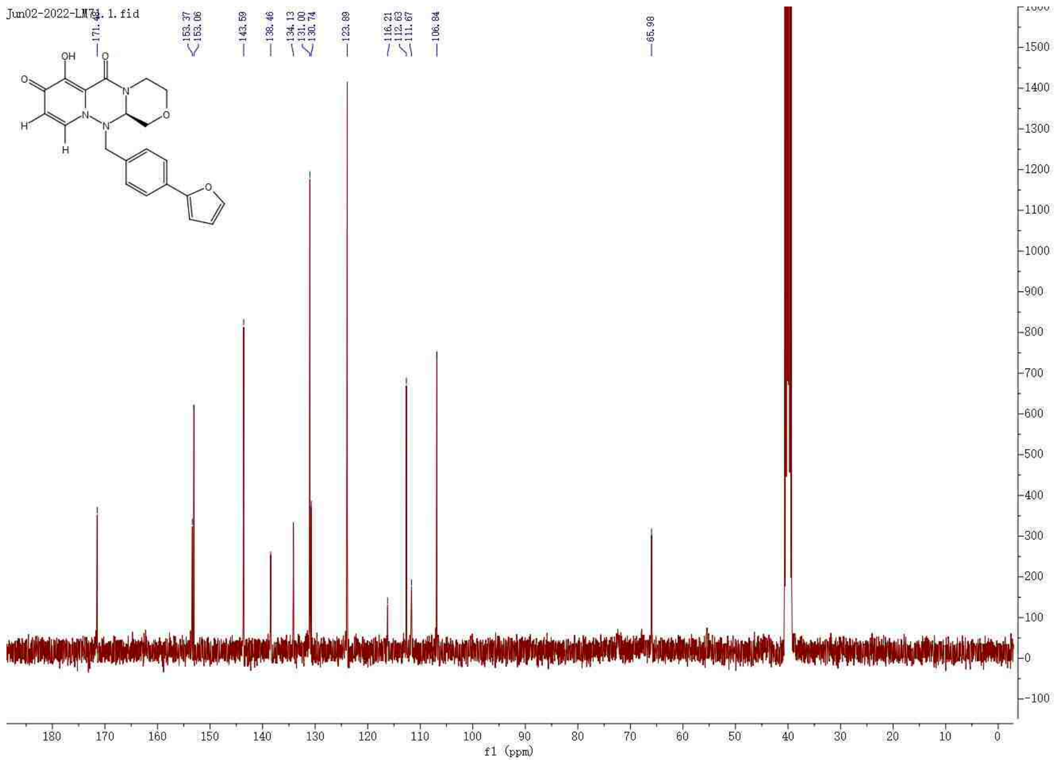

^{1}H and ^{13}C NMR spectra of compound **17**

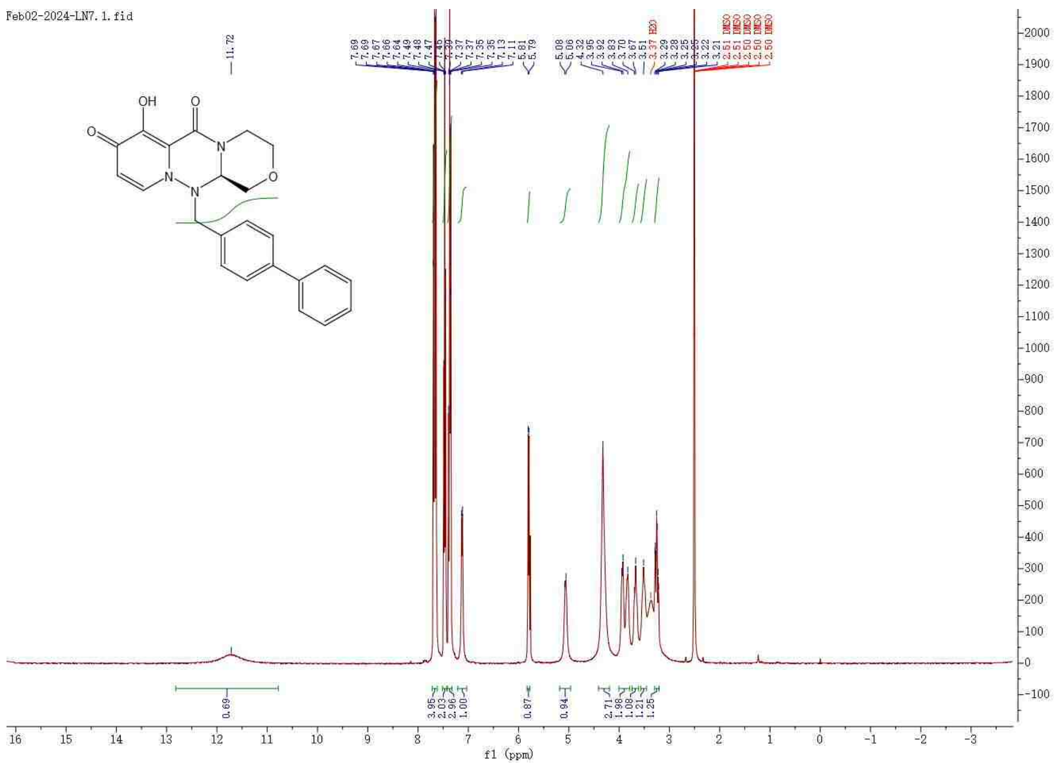

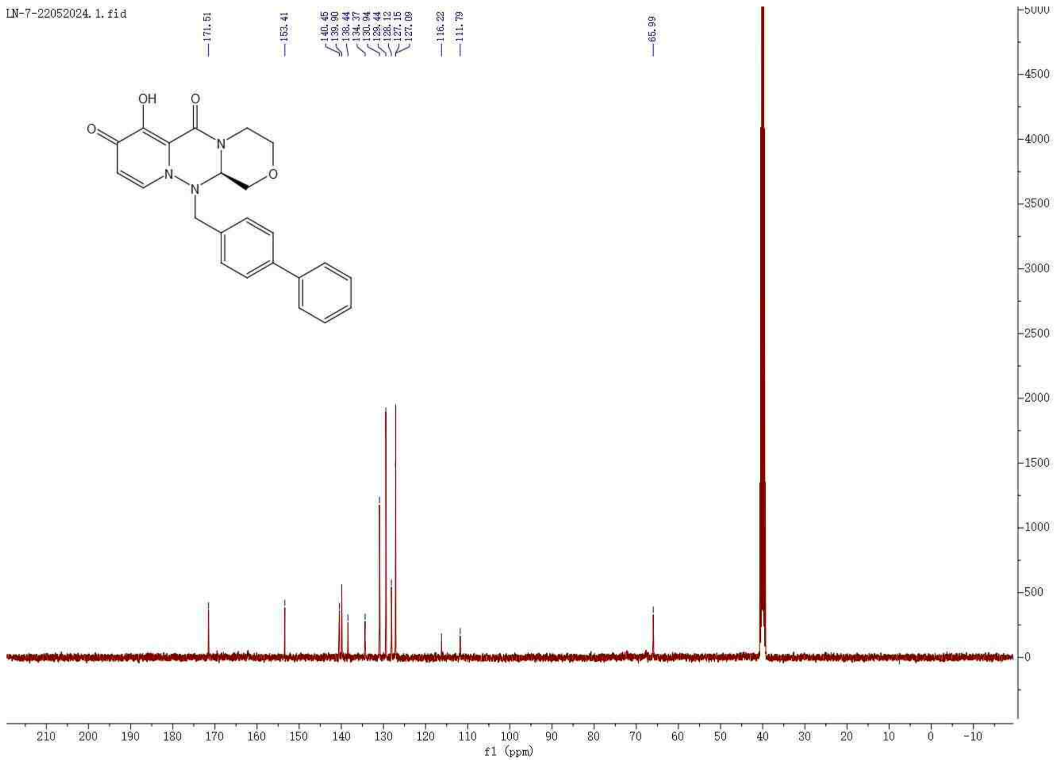

^{1}H and ^{13}C NMR spectra of compound **18**

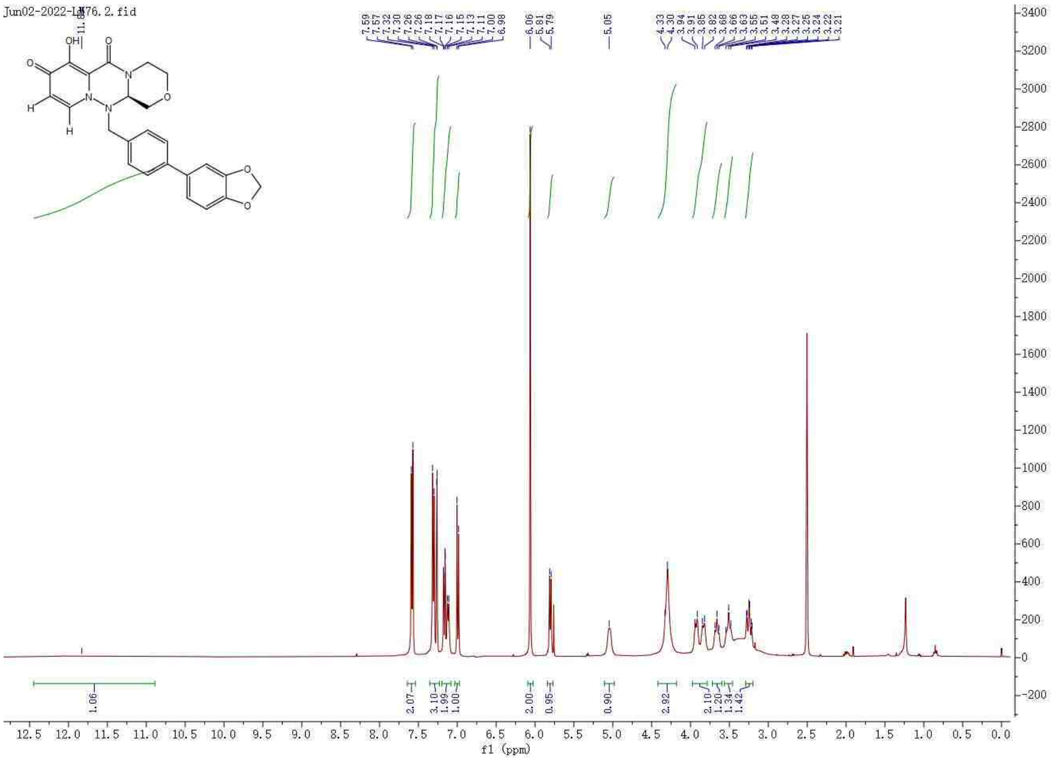

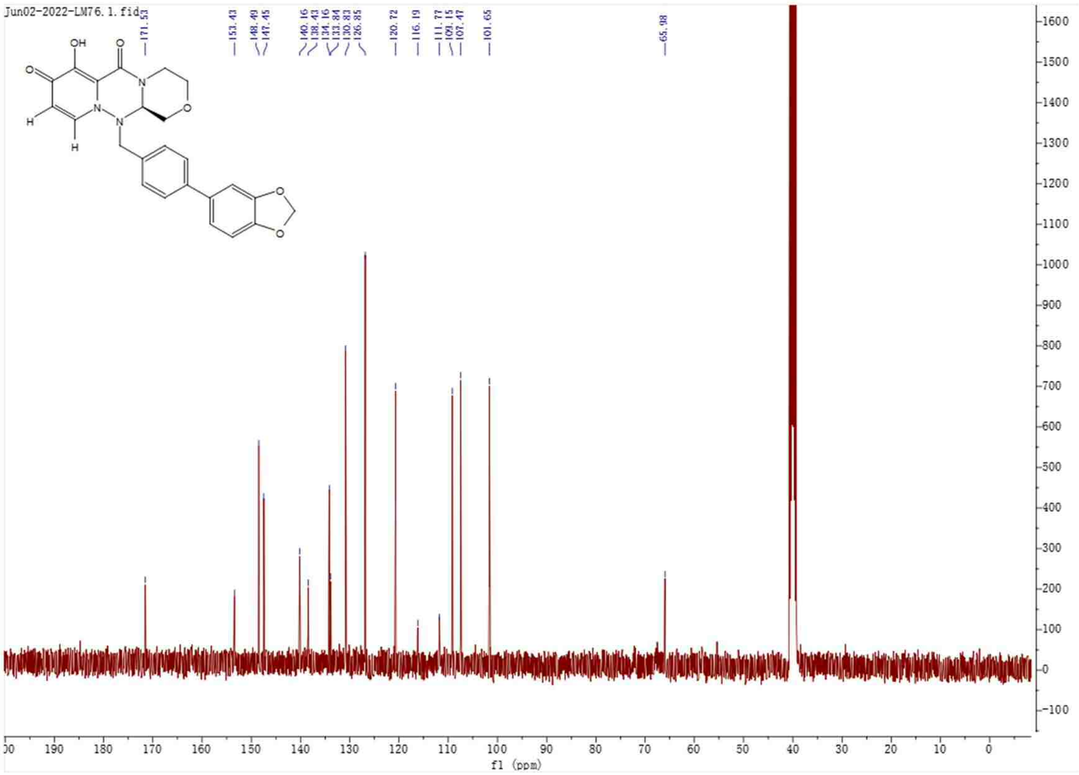

^{1}H and ^{13}C NMR spectra of compound **19**

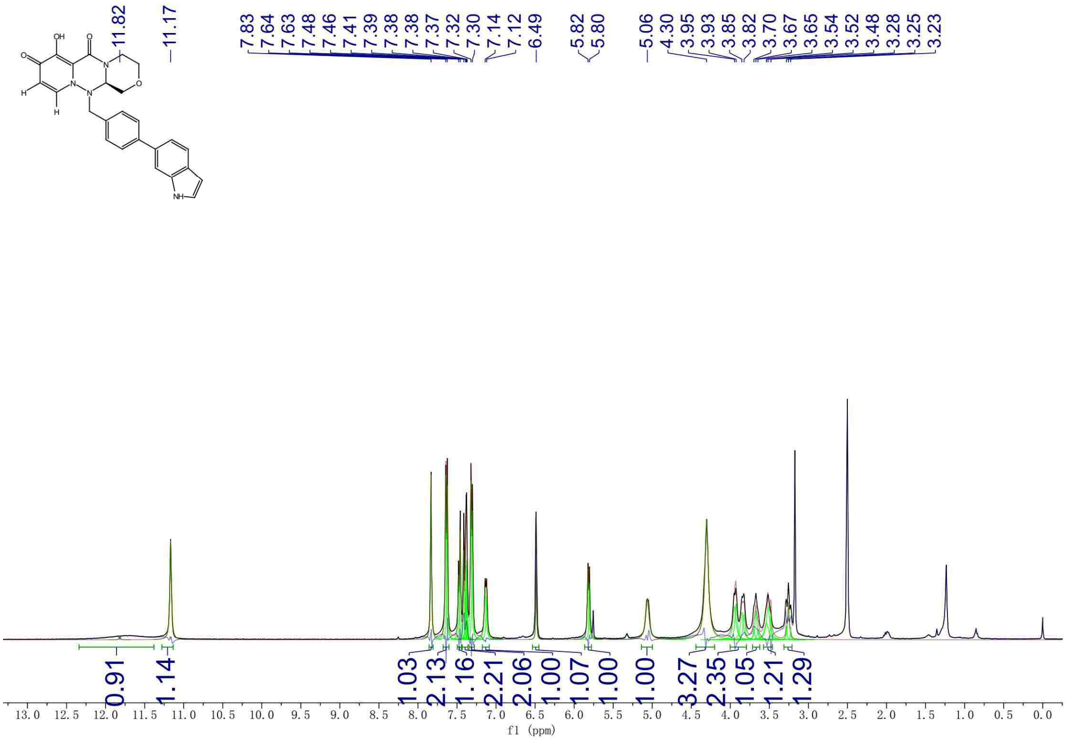

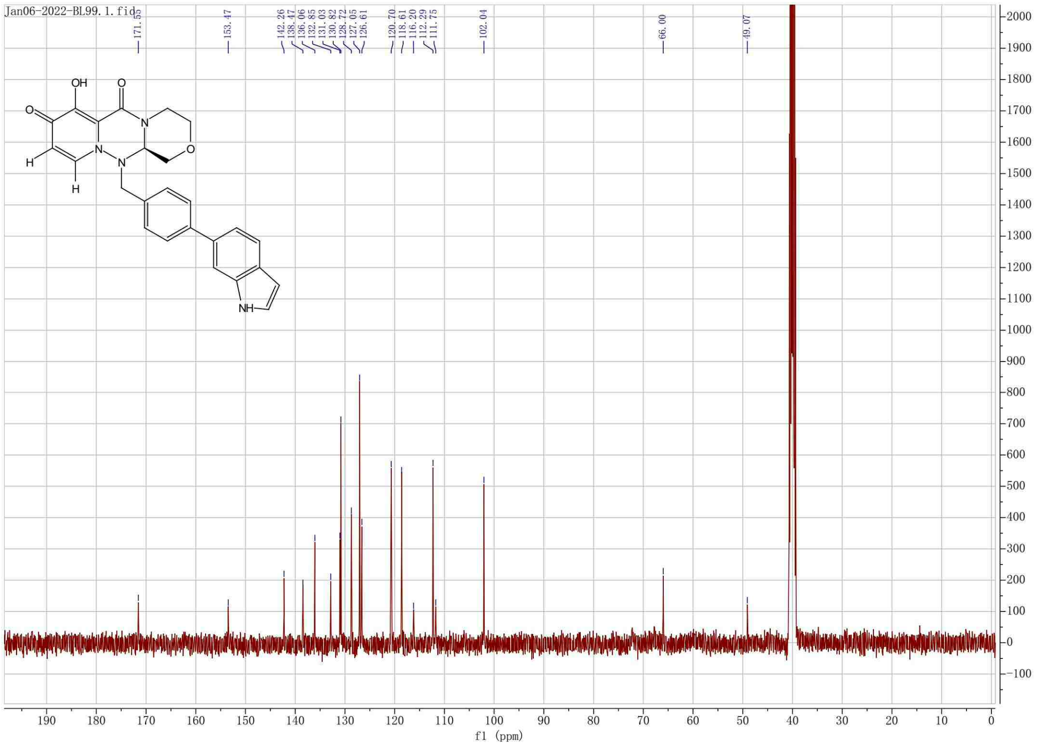

^{1}H and ^{13}C NMR spectra of compound **20**

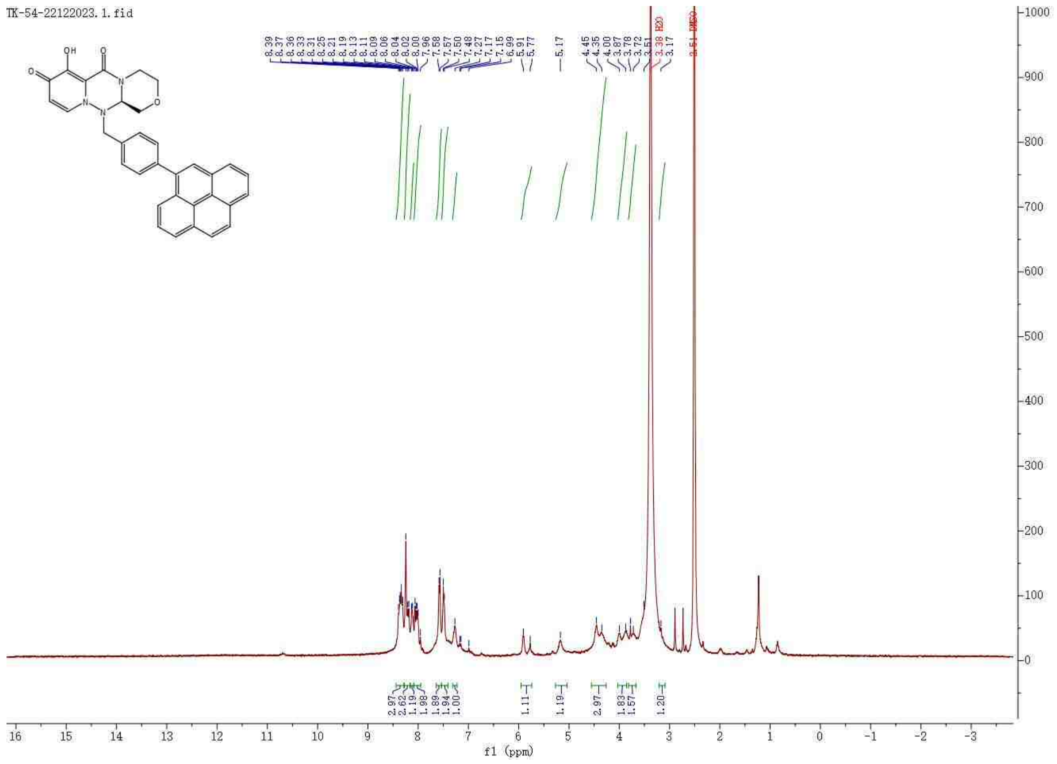

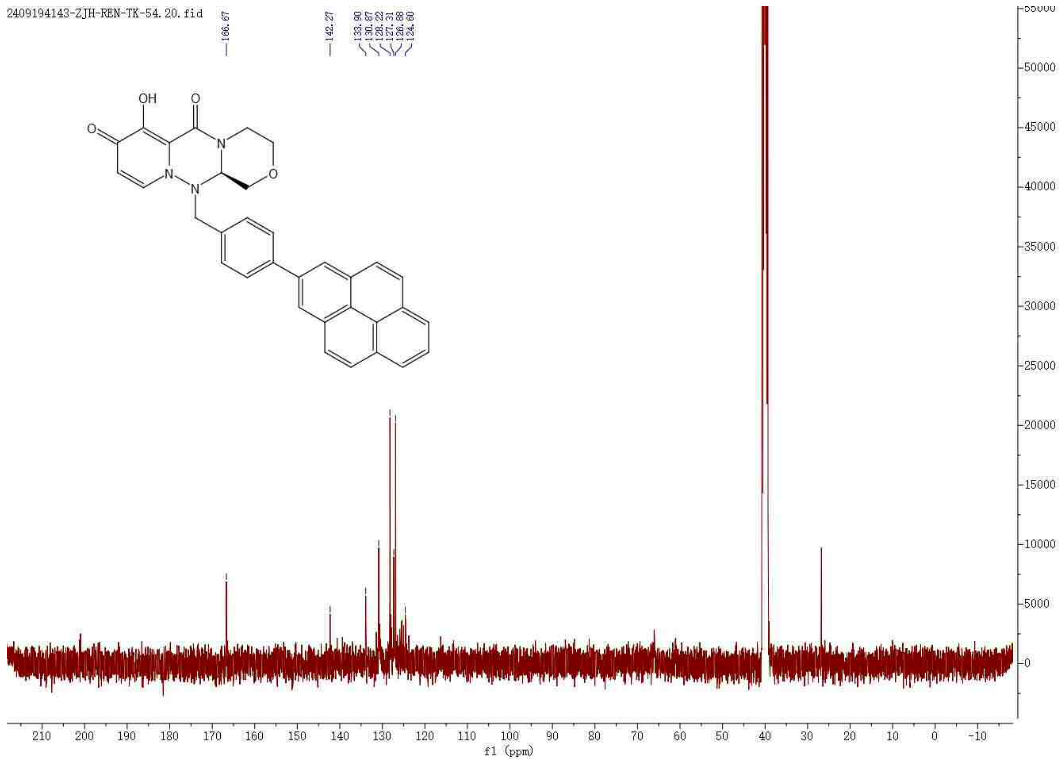

^{1}H and ^{13}C NMR spectra of compound **21**

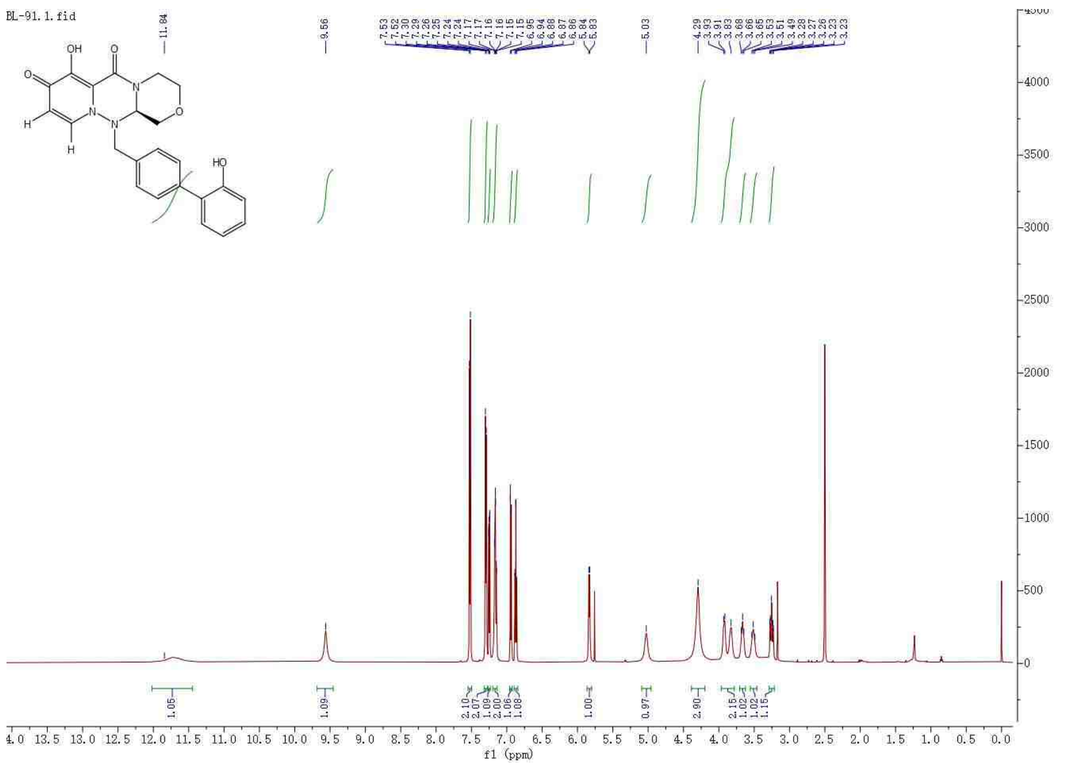

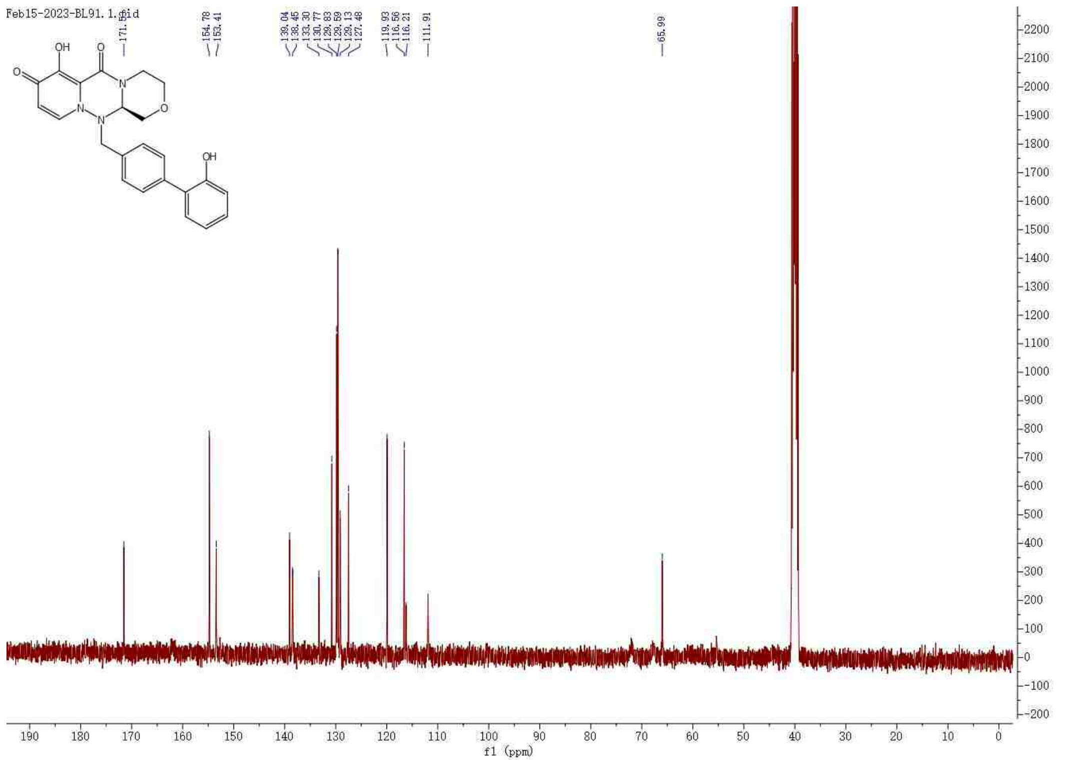

^{1}H and ^{13}C NMR spectra of compound **22**

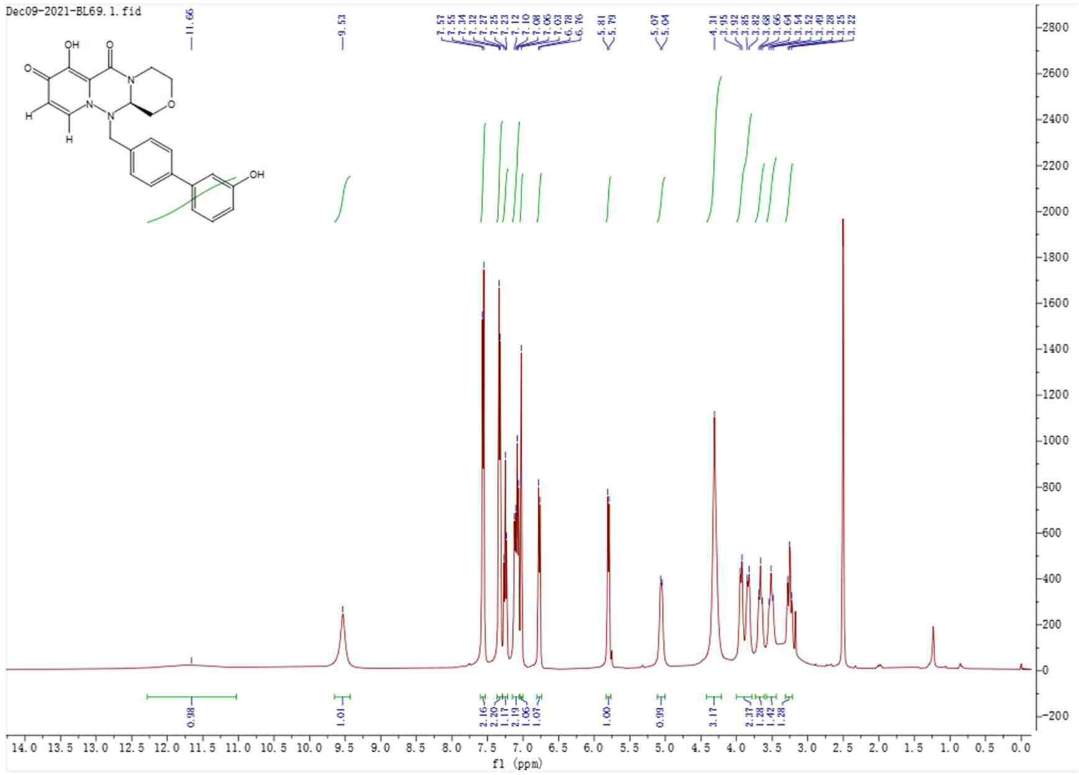

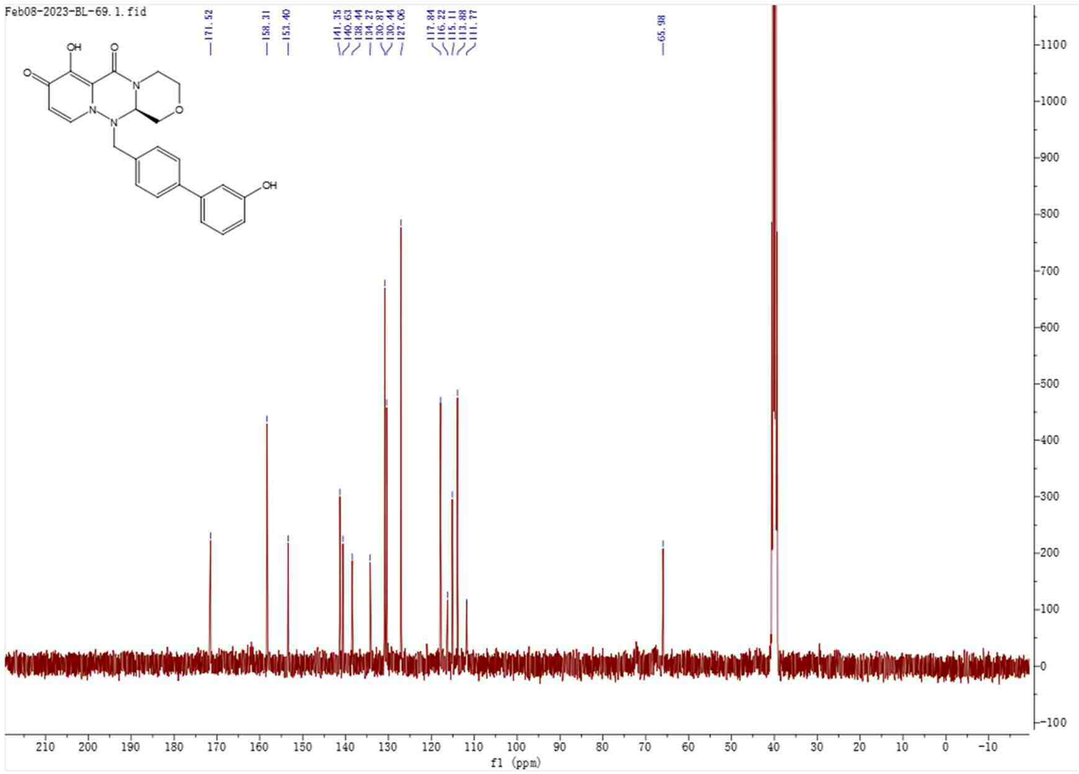

^{1}H and ^{13}C NMR spectra of compound **23**

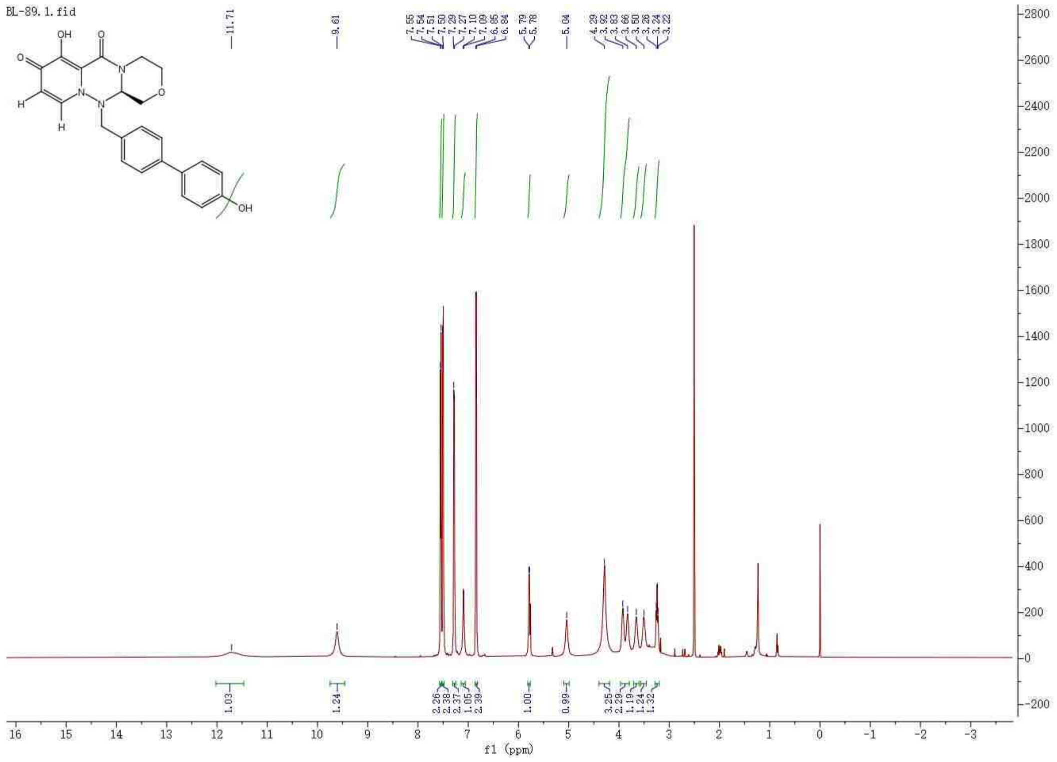

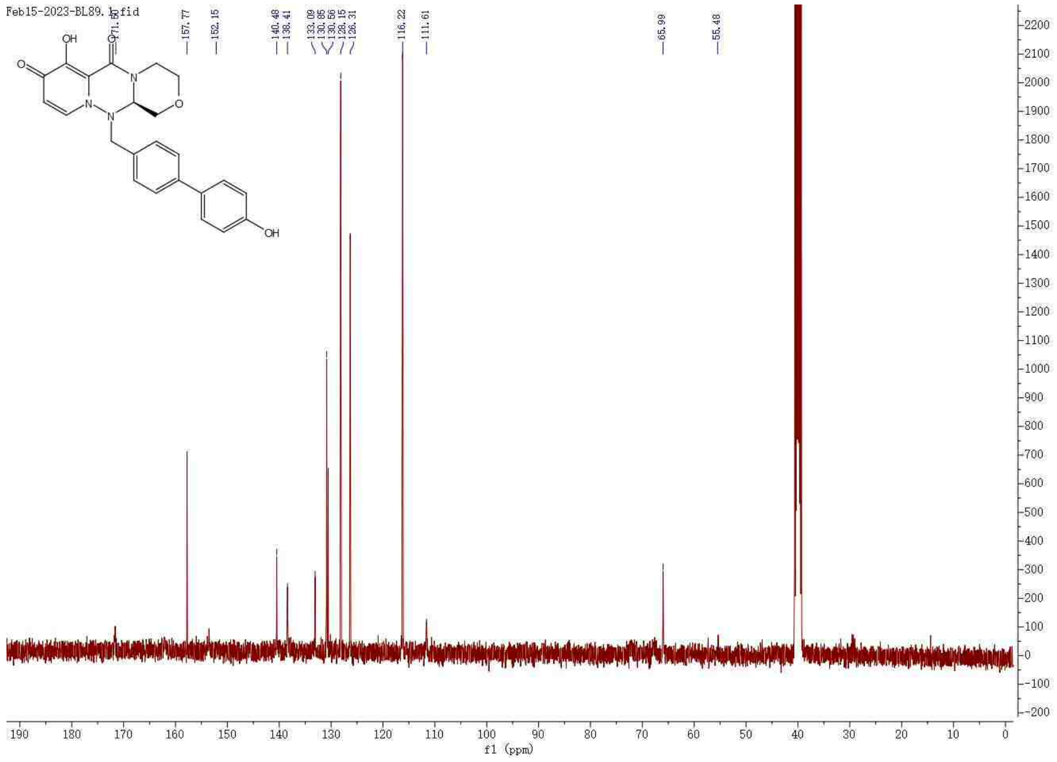

^{1}H and ^{13}C NMR spectra of compound **24**

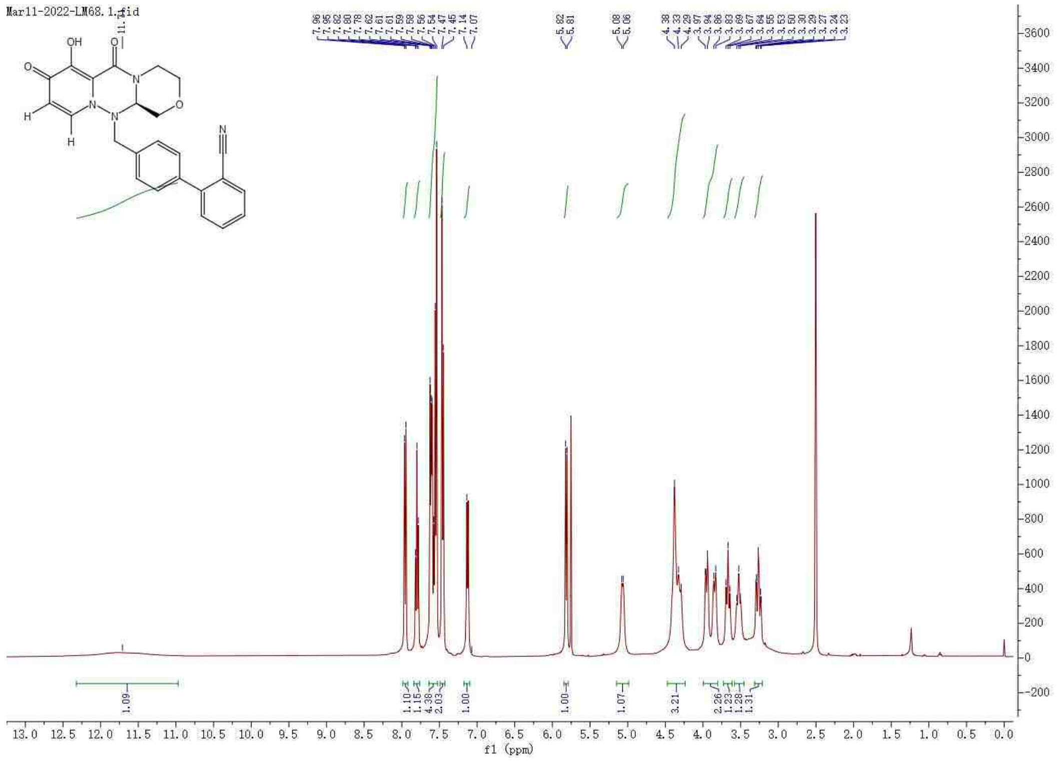

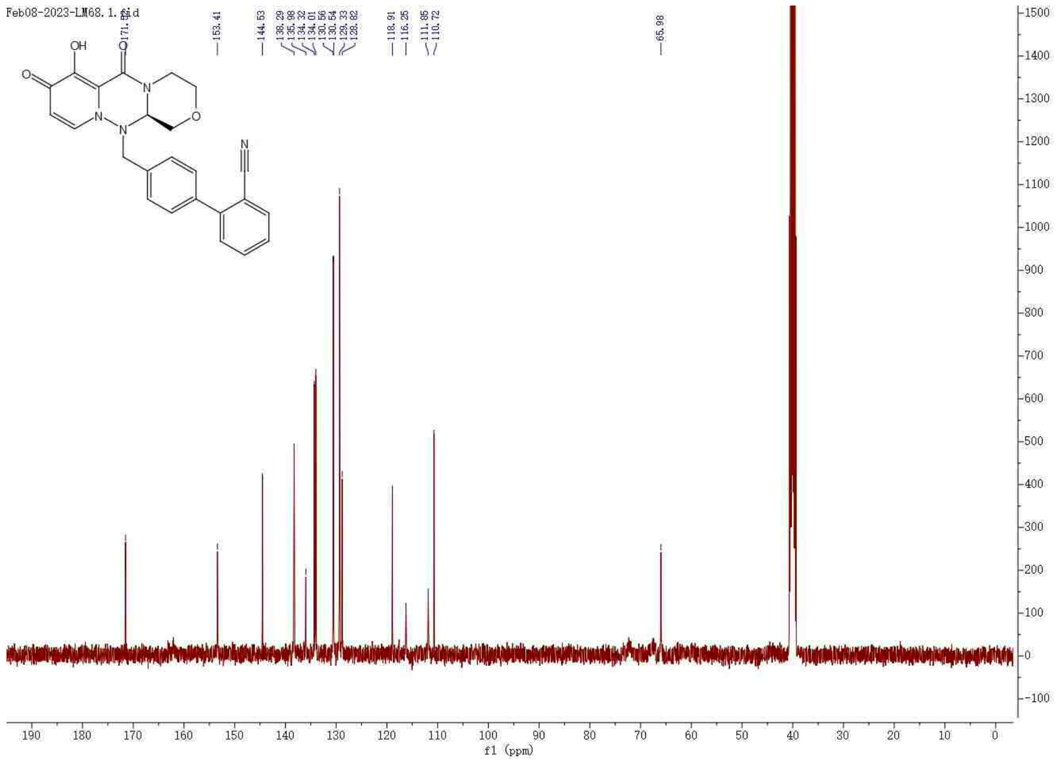

^{1}H and ^{13}C NMR spectra of compound **25**

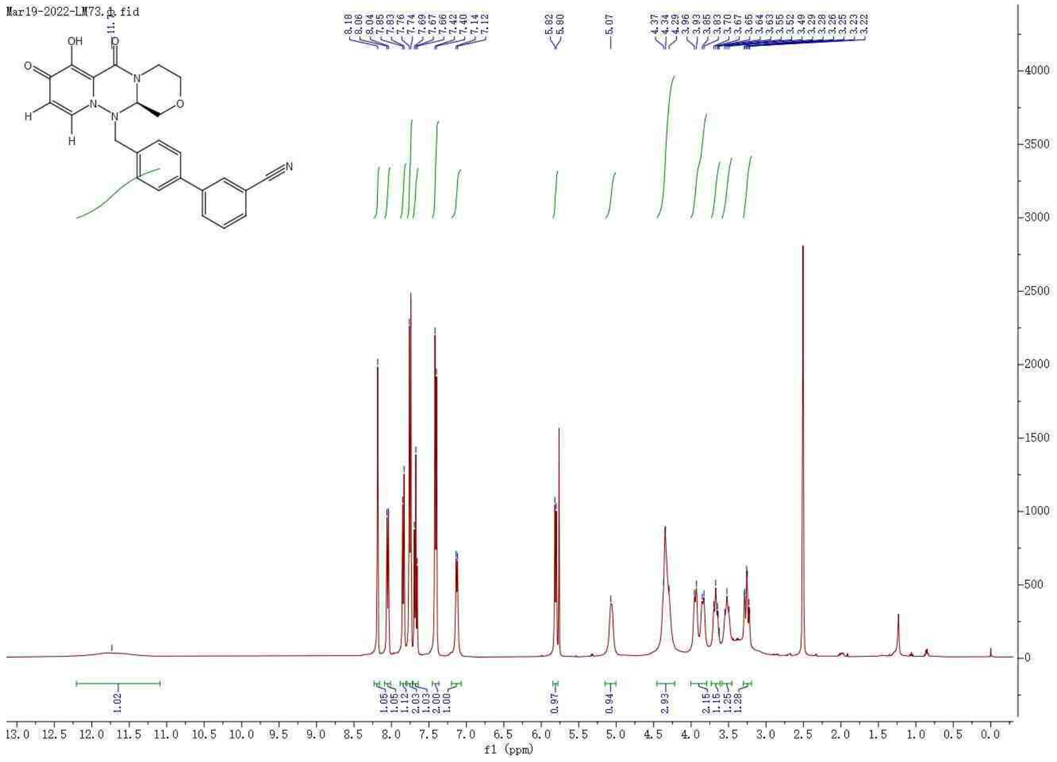

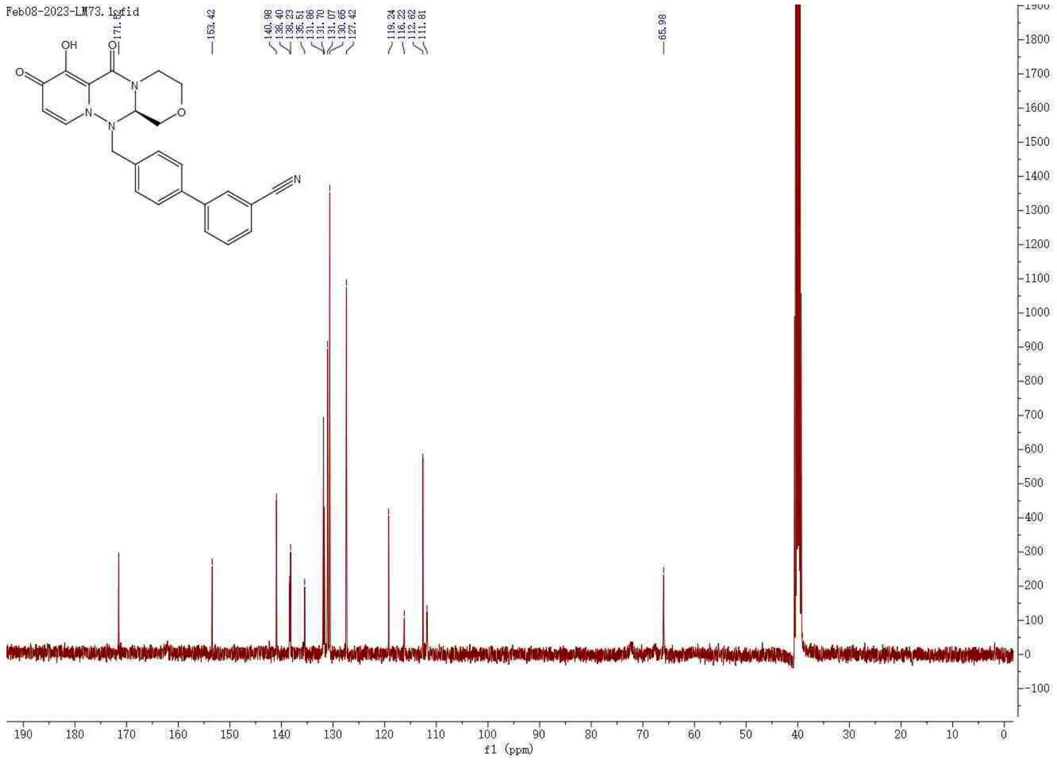

^{1}H and ^{13}C NMR spectra of compound **26**

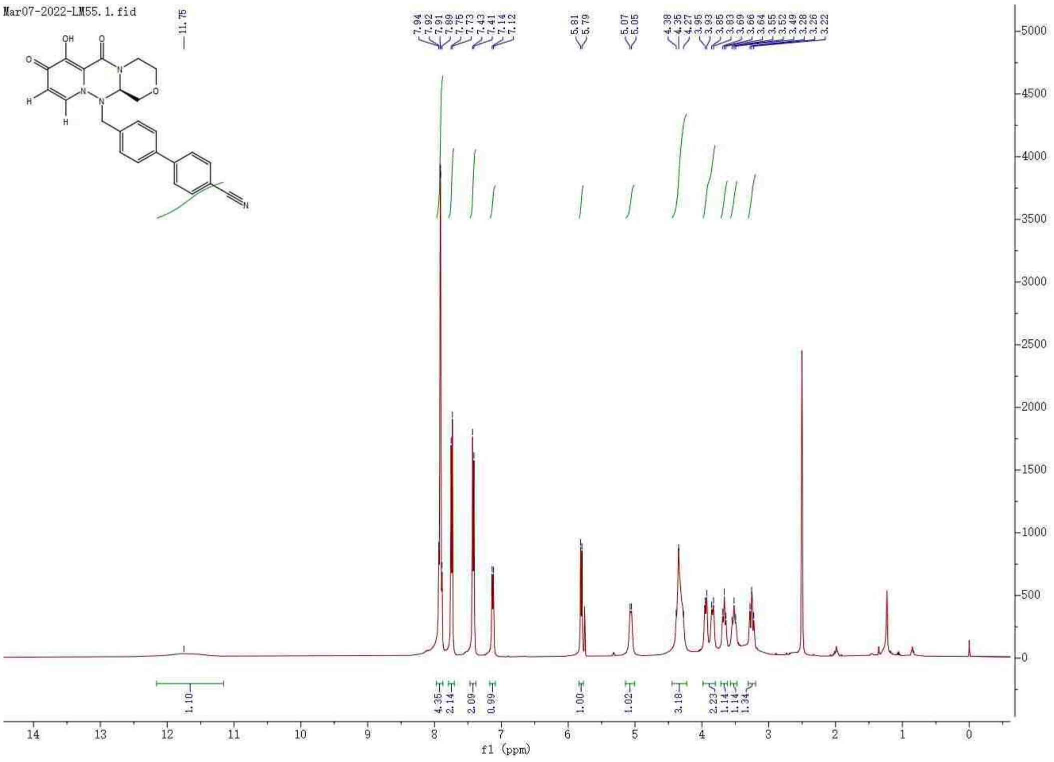

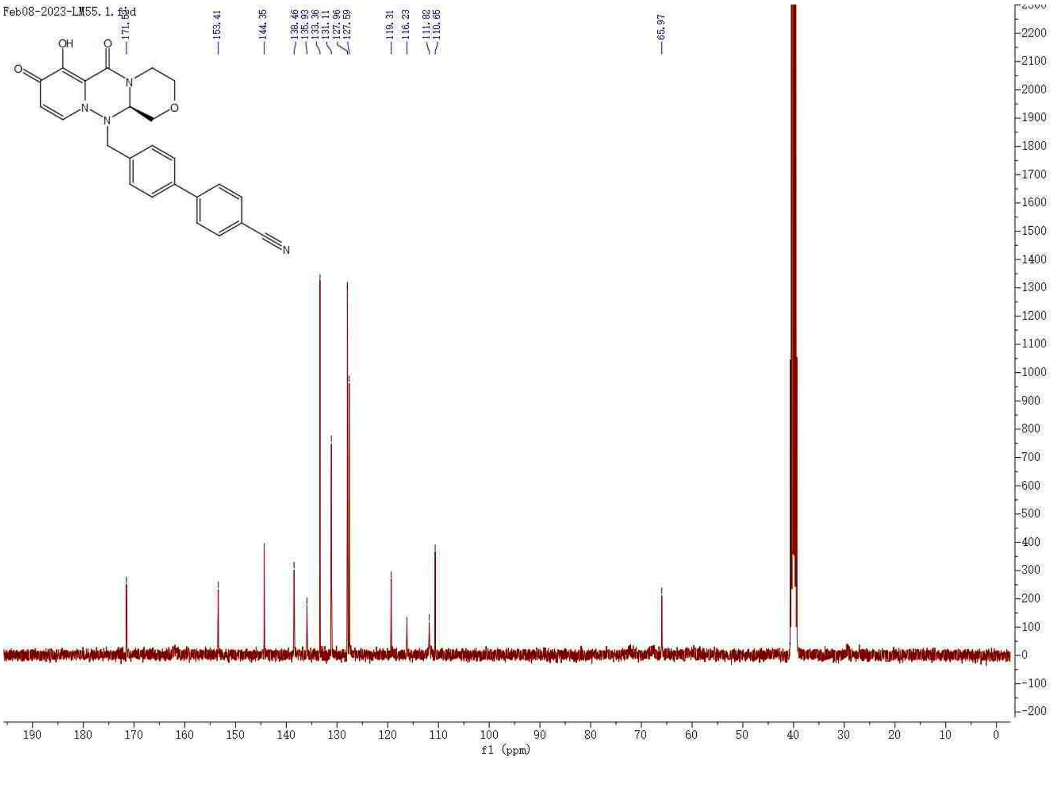

^{1}H and ^{13}C NMR spectra of compound **27**

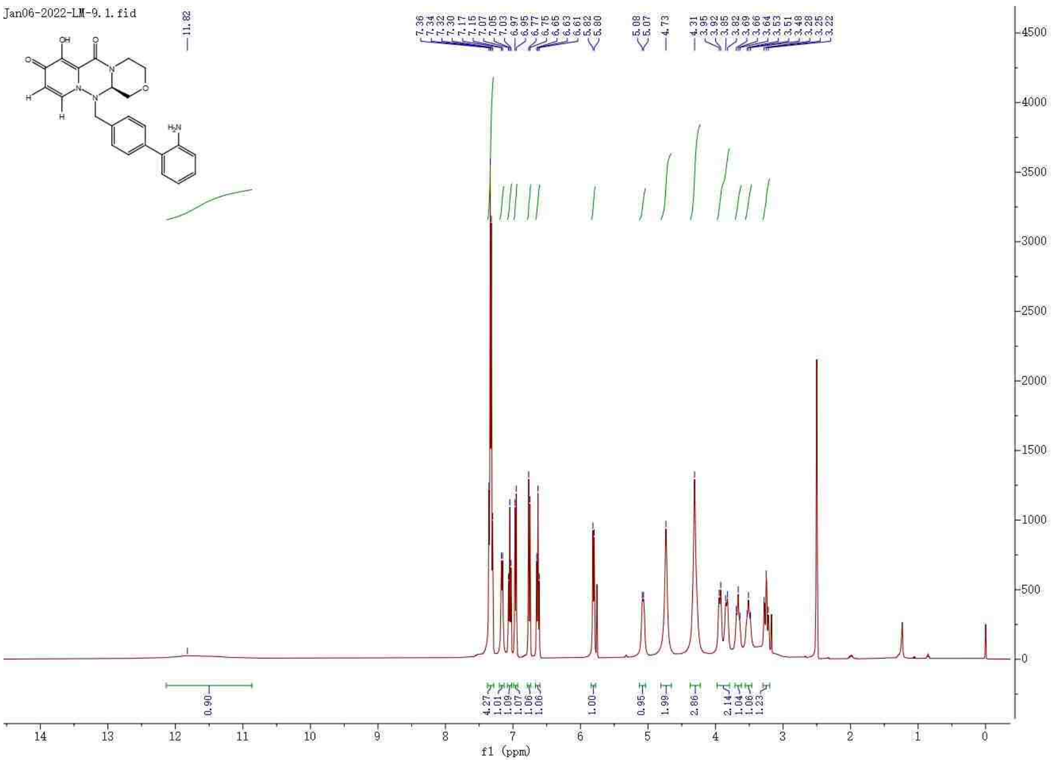

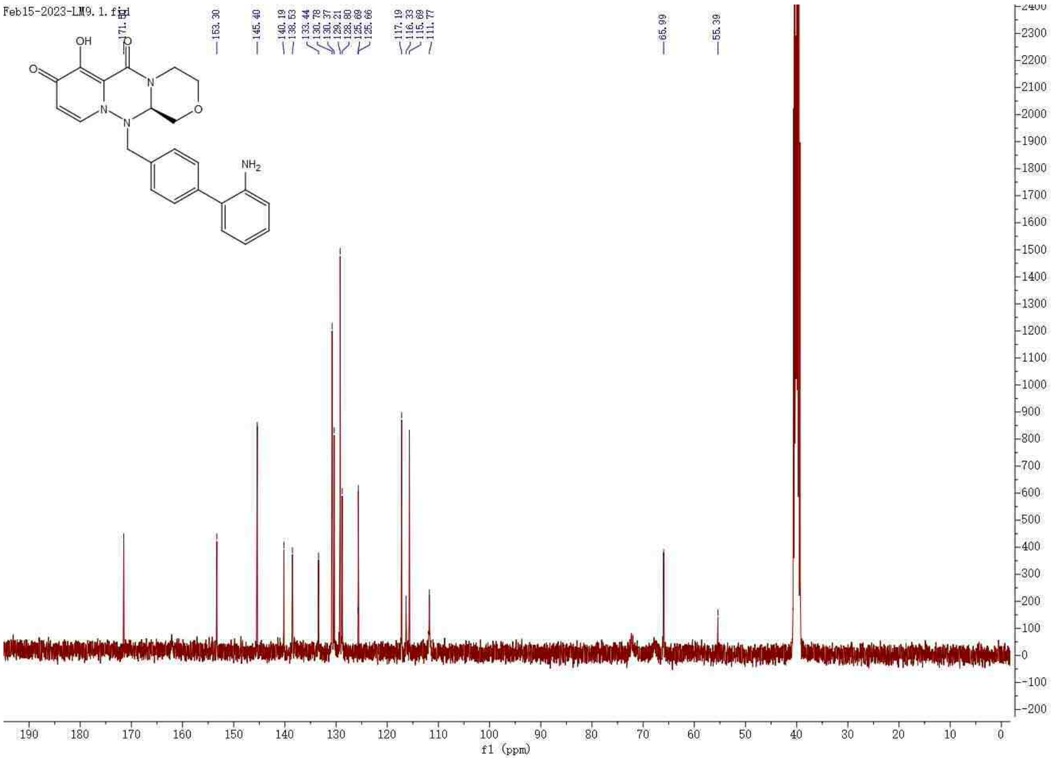

^{1}H and ^{13}C NMR spectra of compound **28**

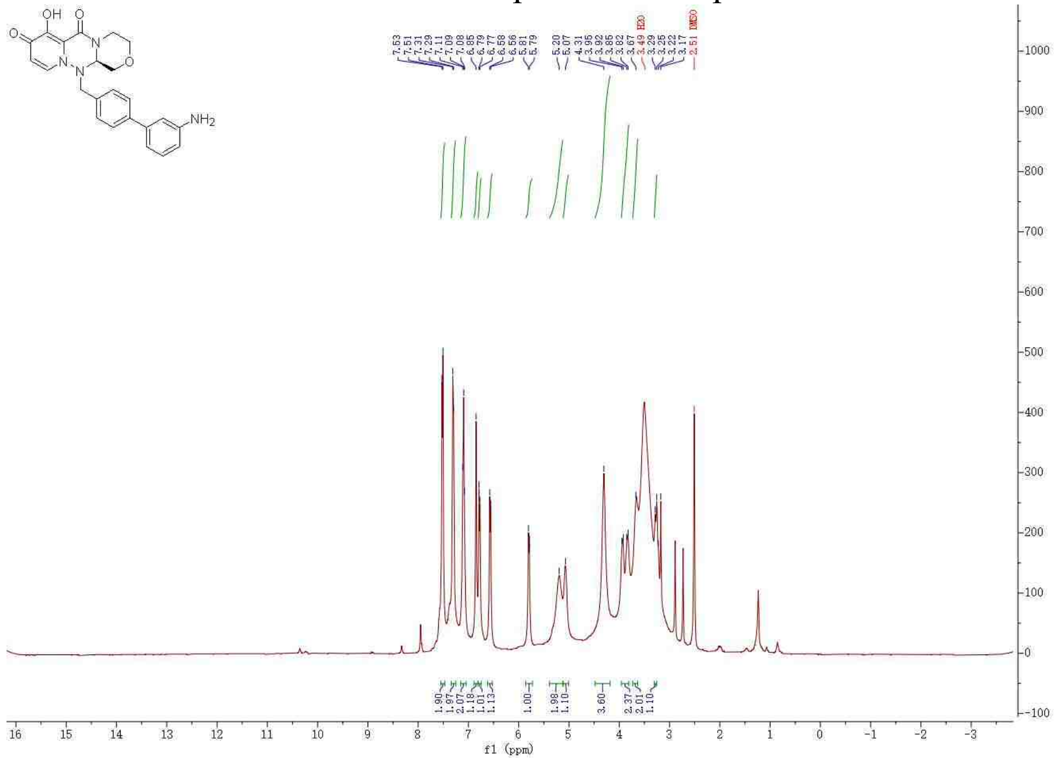

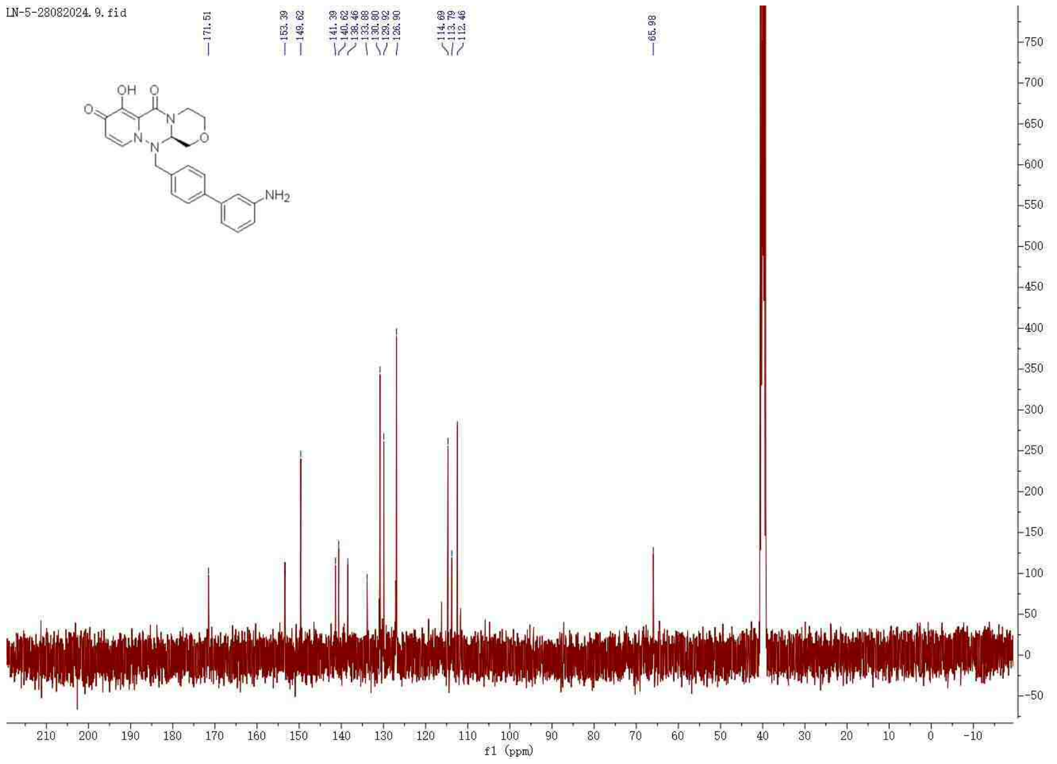

^{1}H and ^{13}C NMR spectra of compound **29**

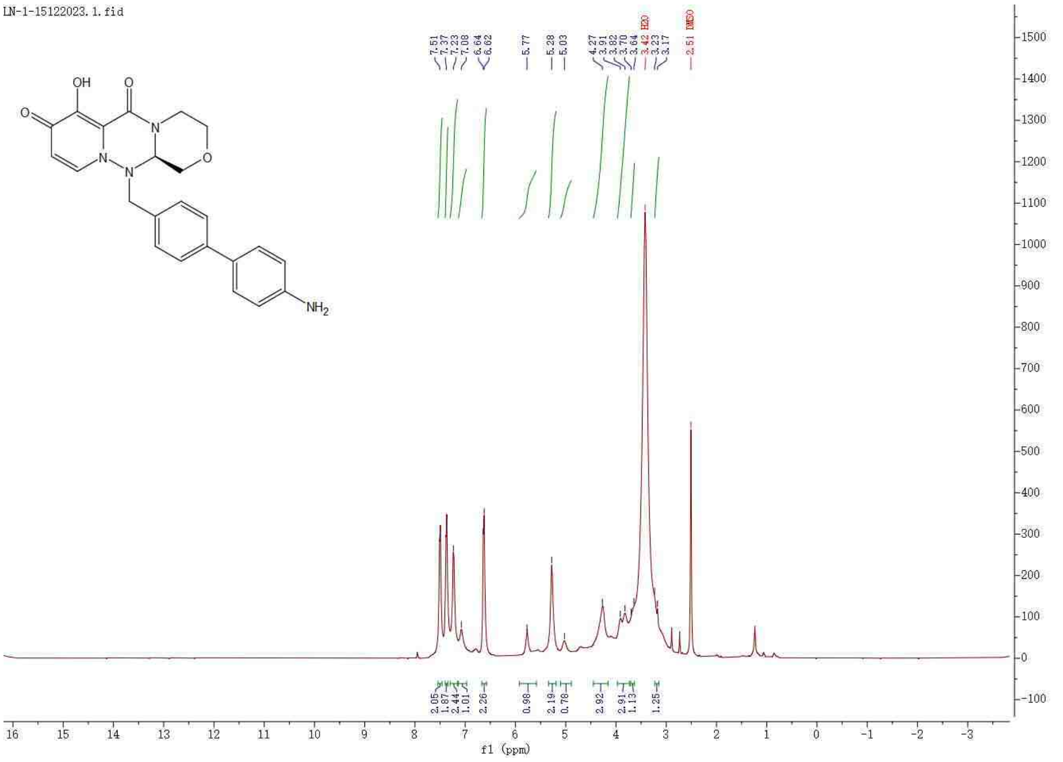

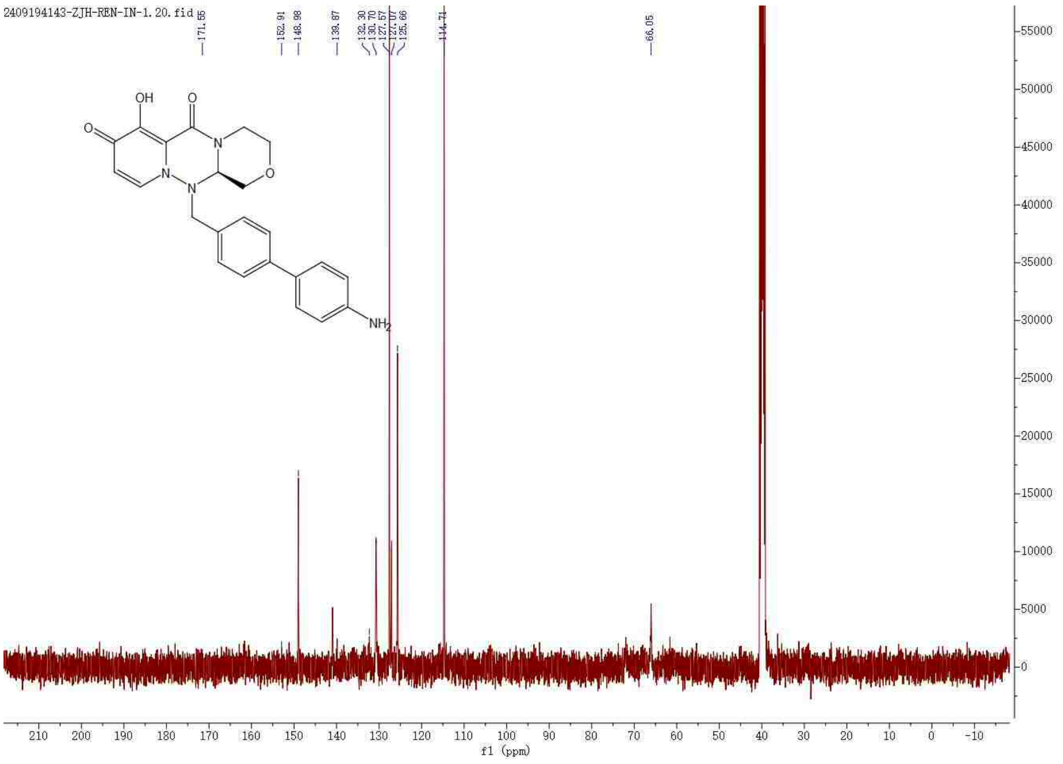

^{1}H and ^{13}C NMR spectra of compound **30**

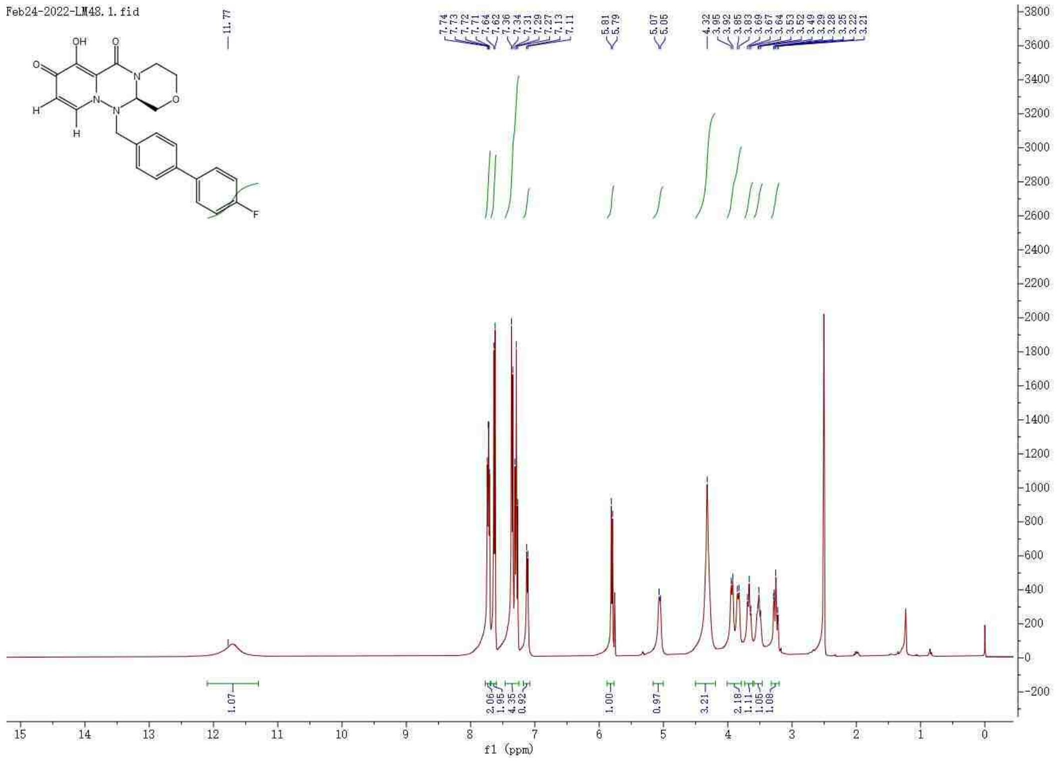

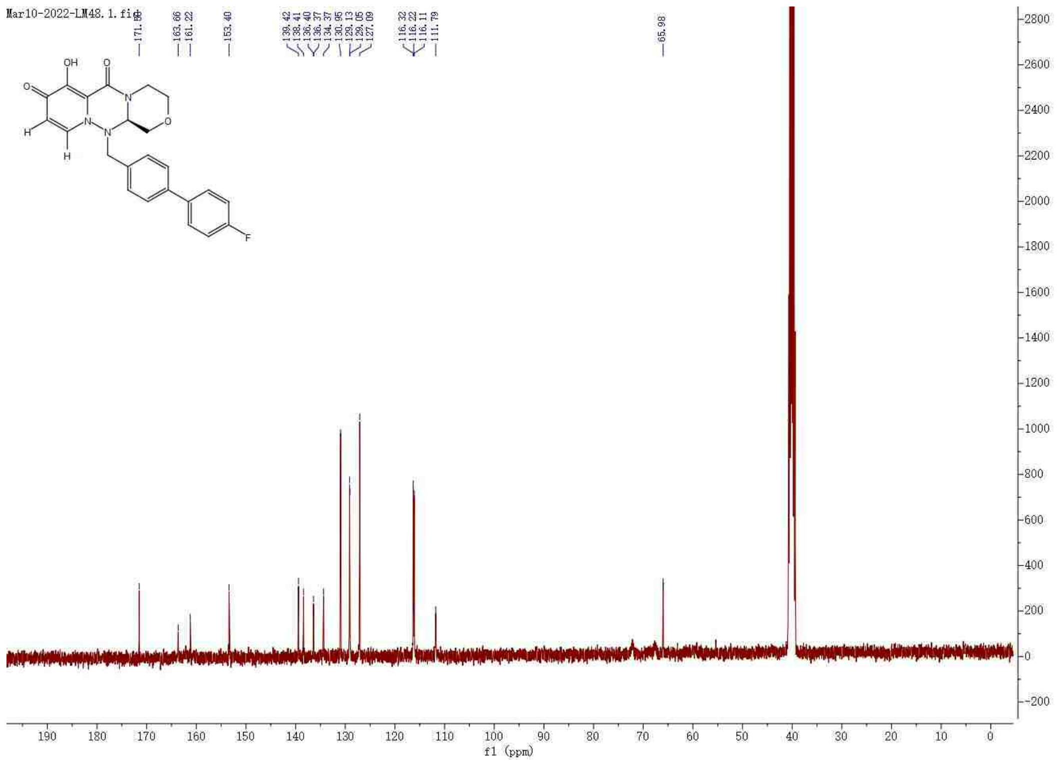

^{1}H and ^{13}C NMR spectra of compound **31**

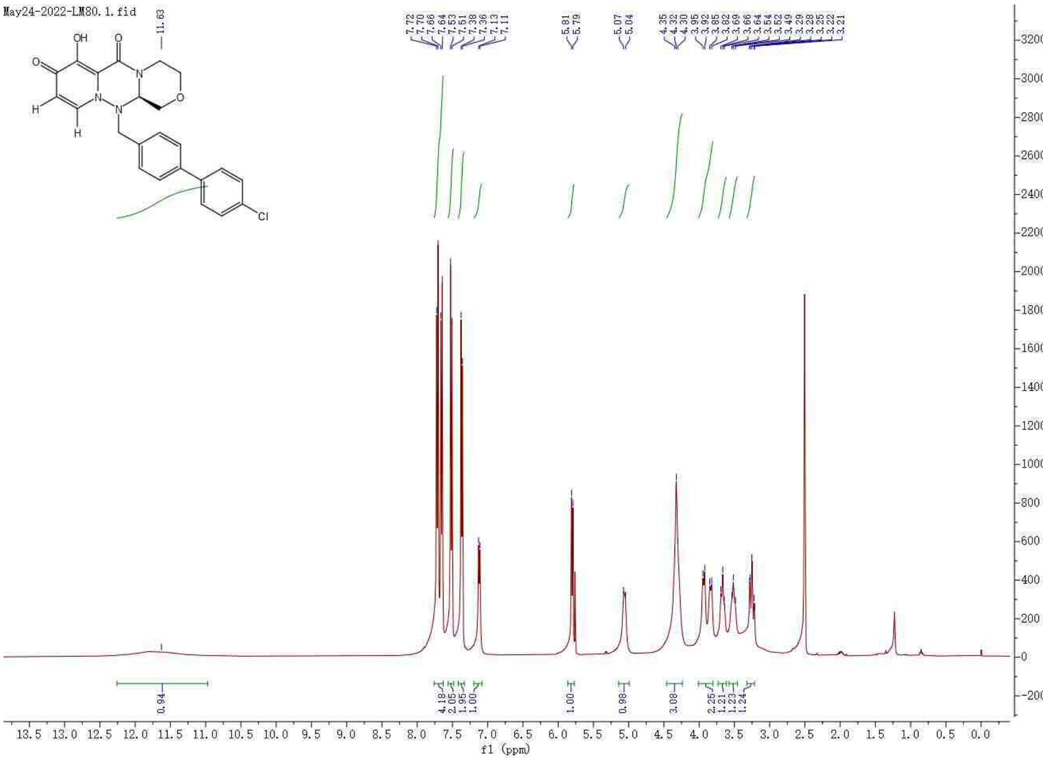

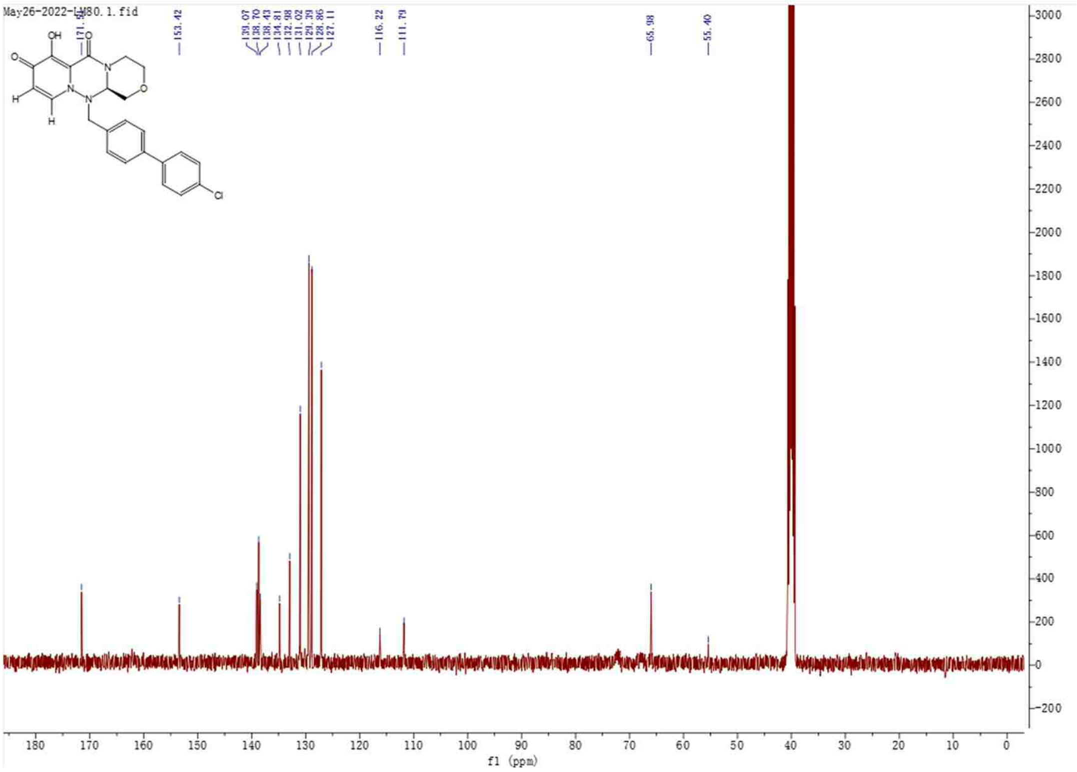

^{1}H and ^{13}C NMR spectra of compound **32**

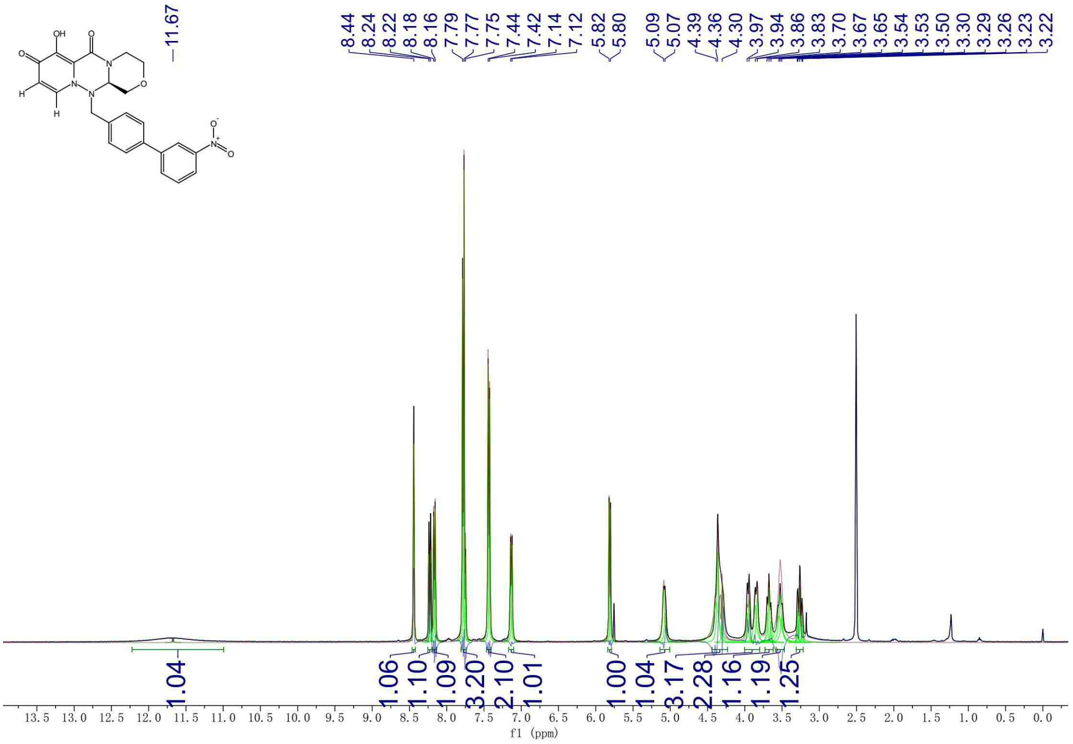

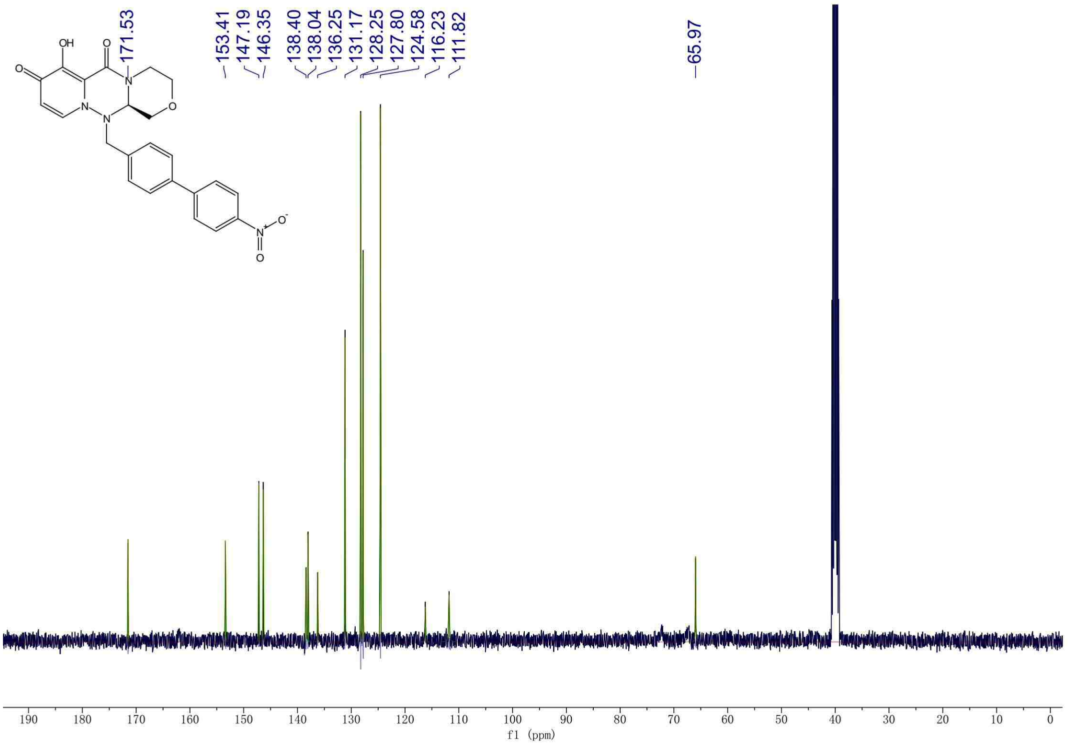

^{1}H and ^{13}C NMR spectra of compound **33**

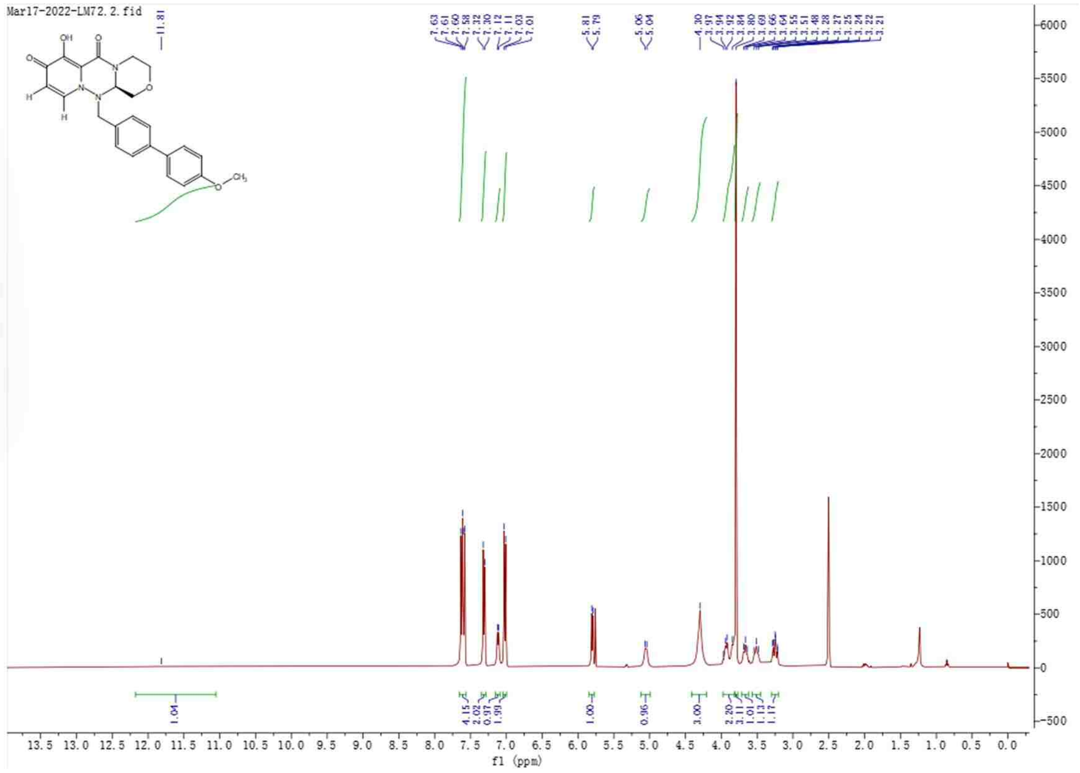

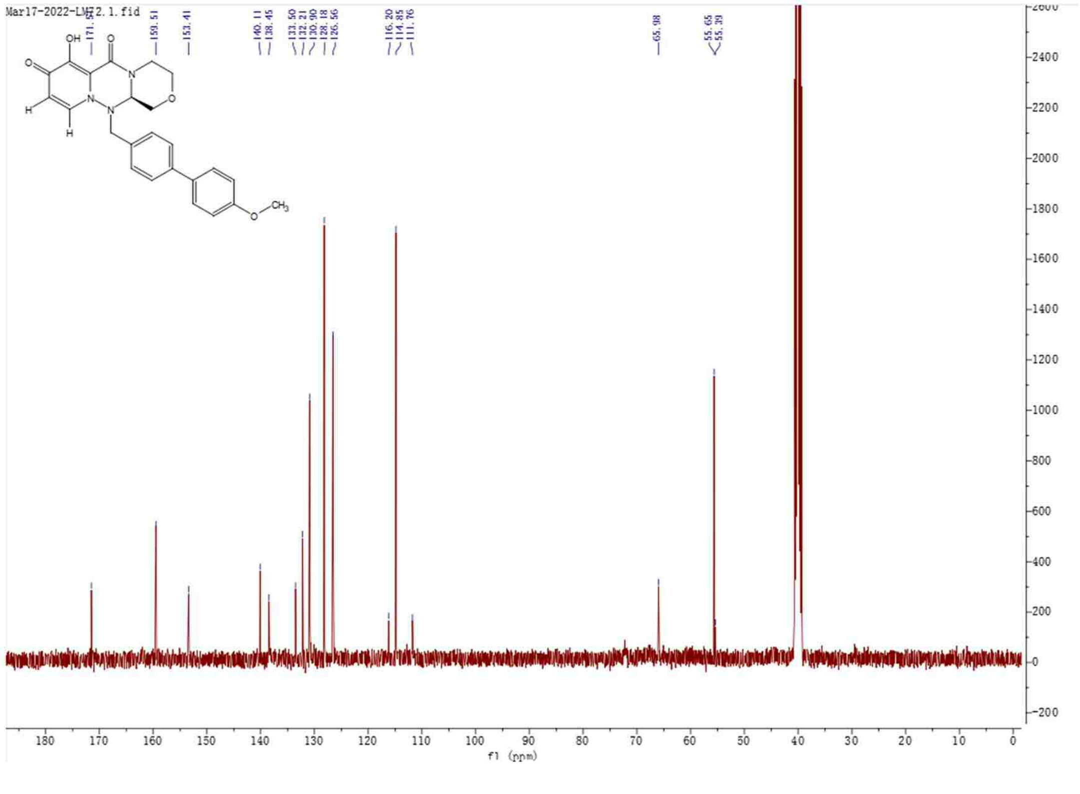

^{1}H and ^{13}C NMR spectra of compound **34**

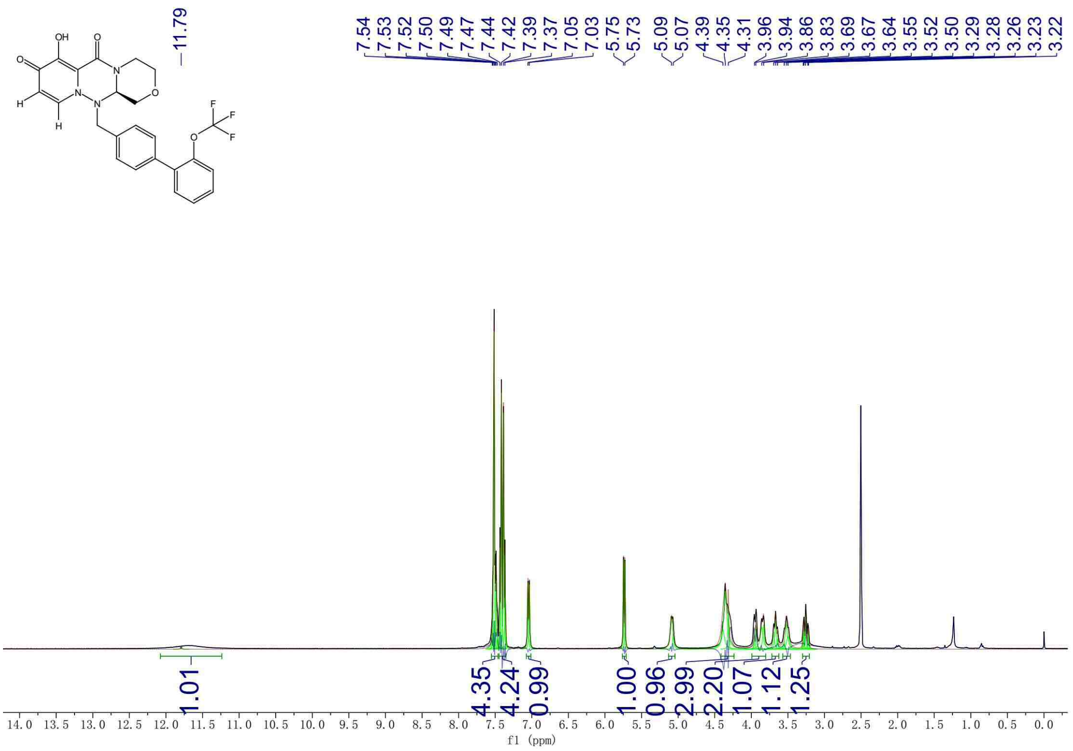

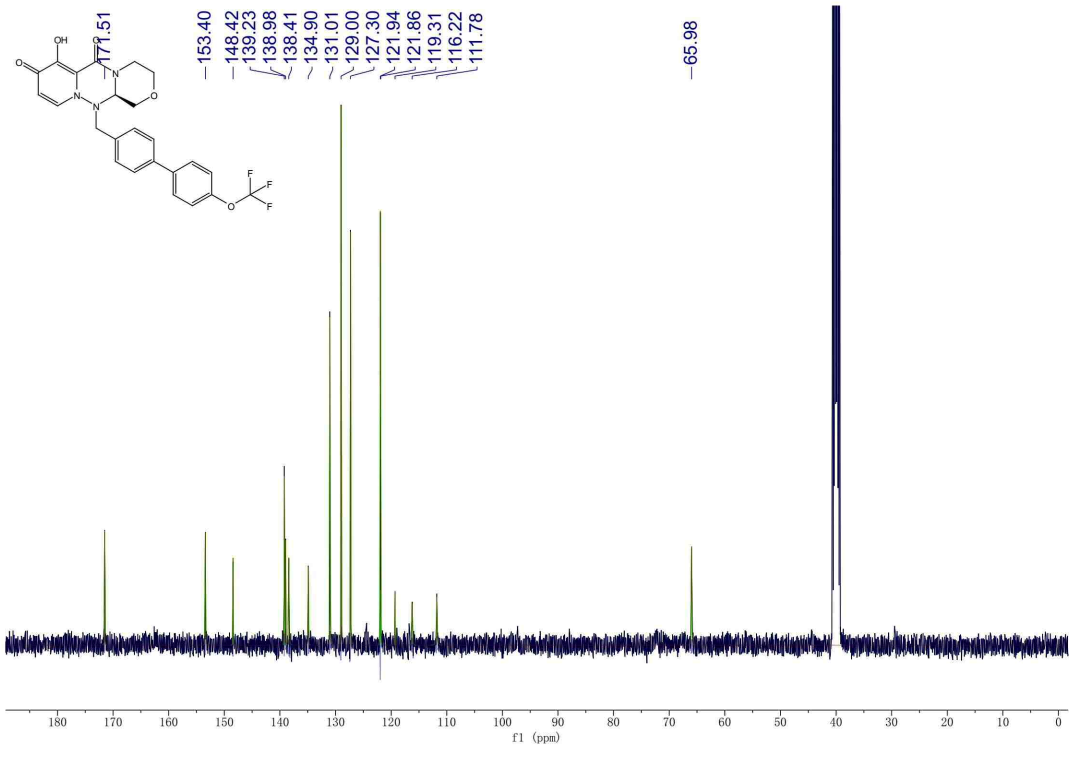

^{1}H and ^{13}C NMR spectra of compound **35**

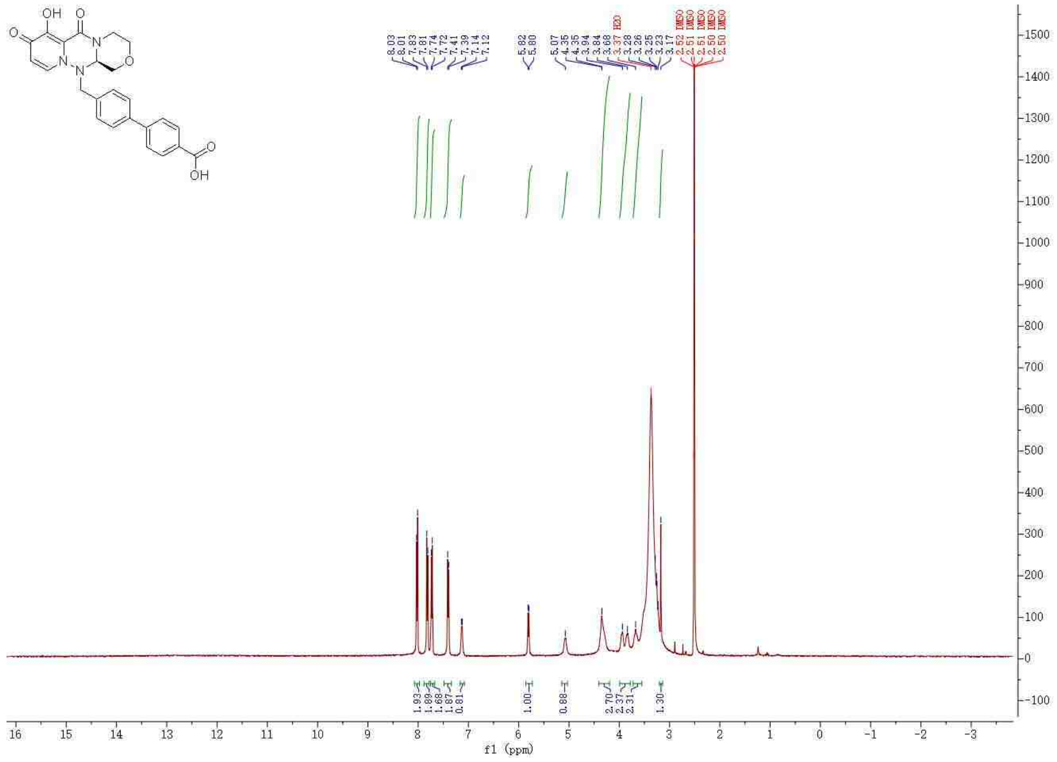

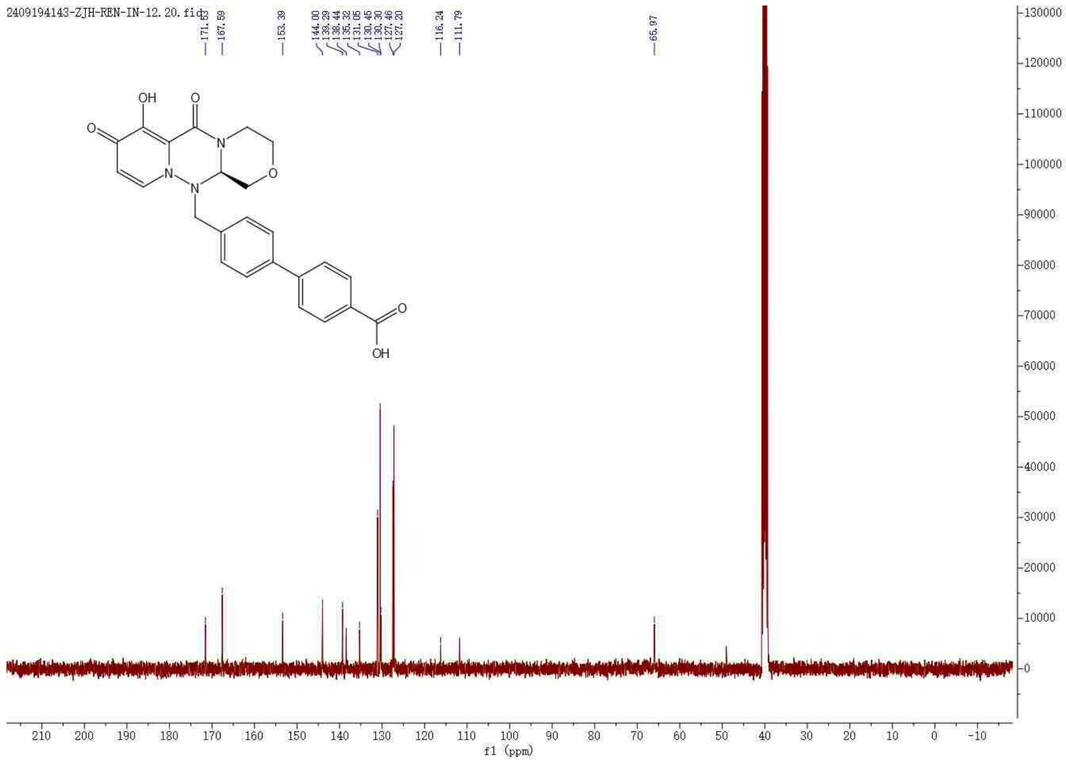

^{1}H and ^{13}C NMR spectra of compound **36**

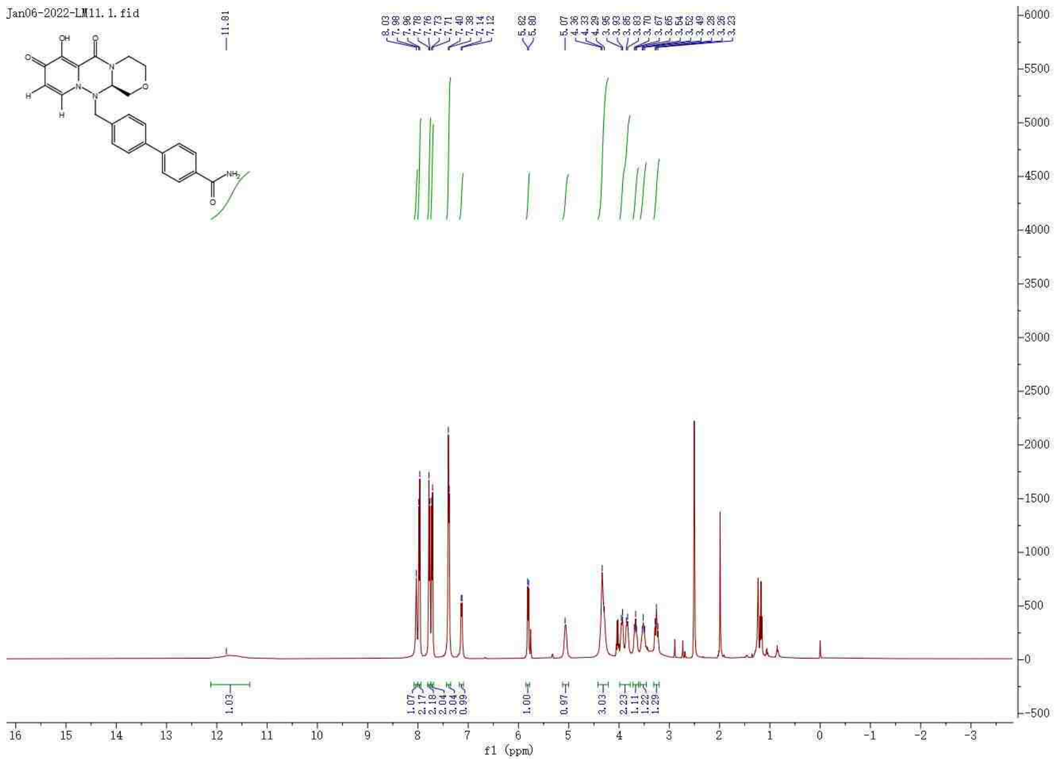

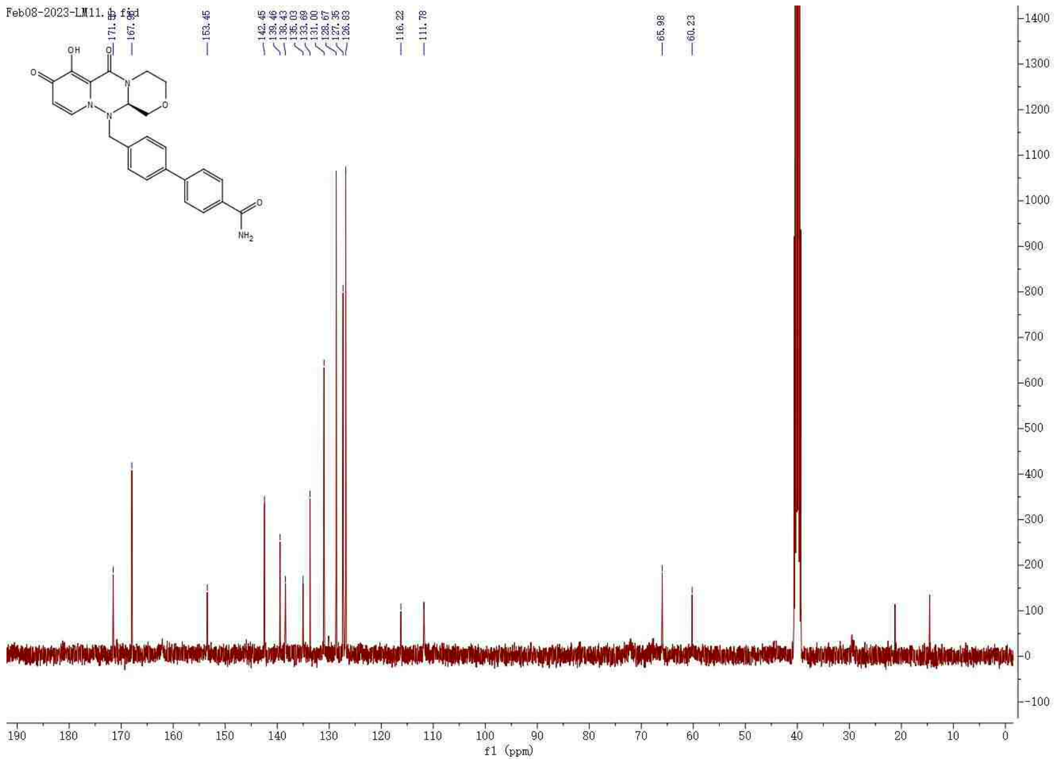

^{1}H and ^{13}C NMR spectra of compound **37**

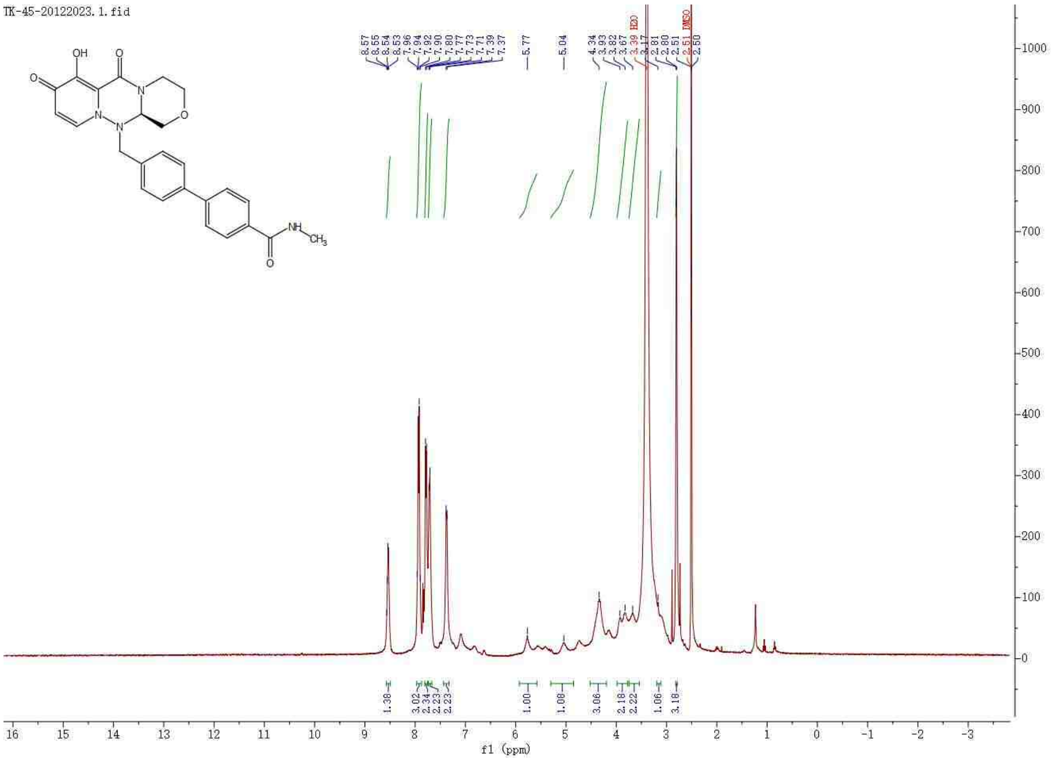

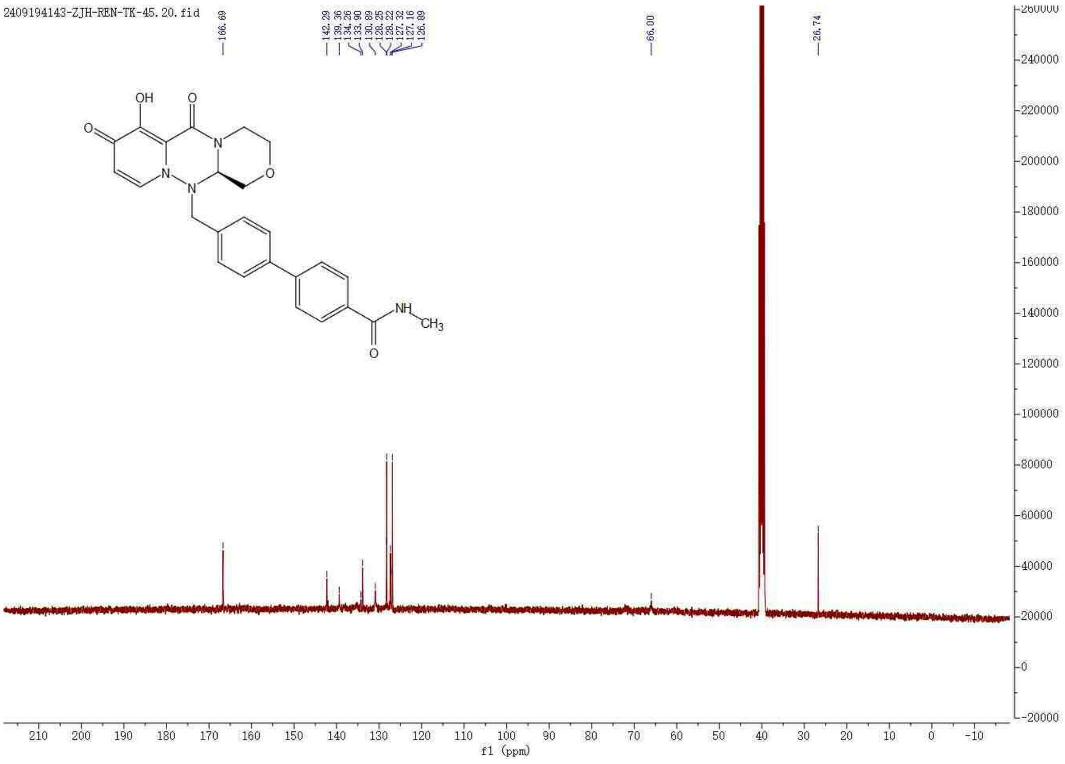

^{1}H and ^{13}C NMR spectra of compound **38**

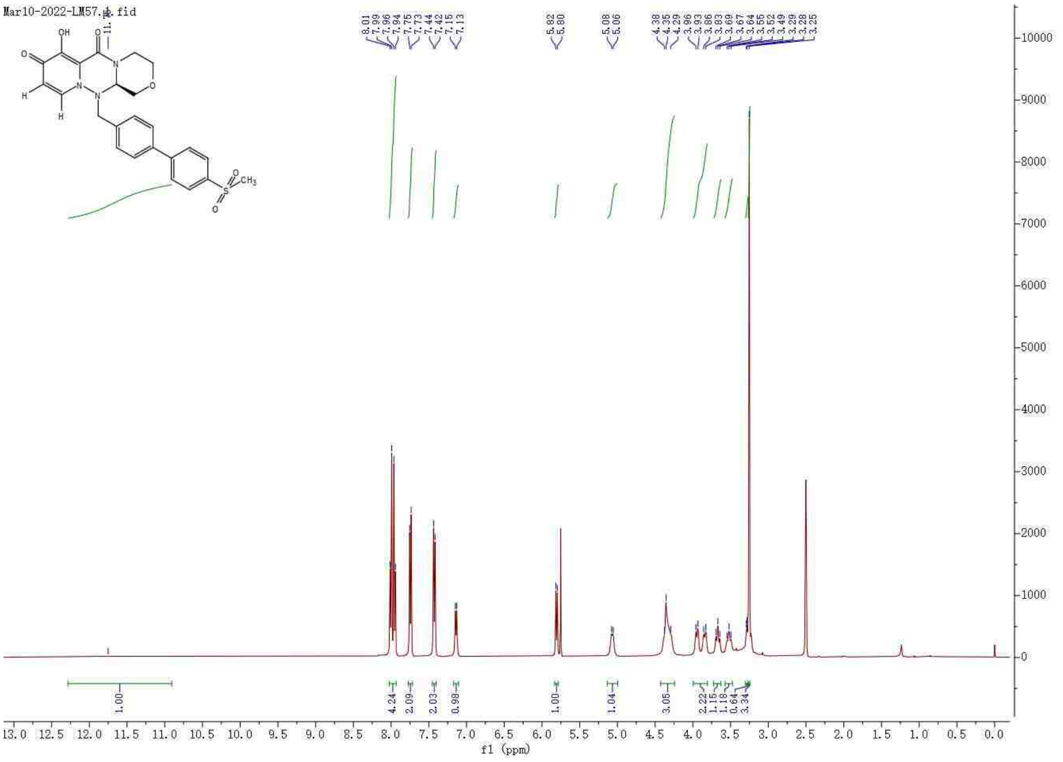

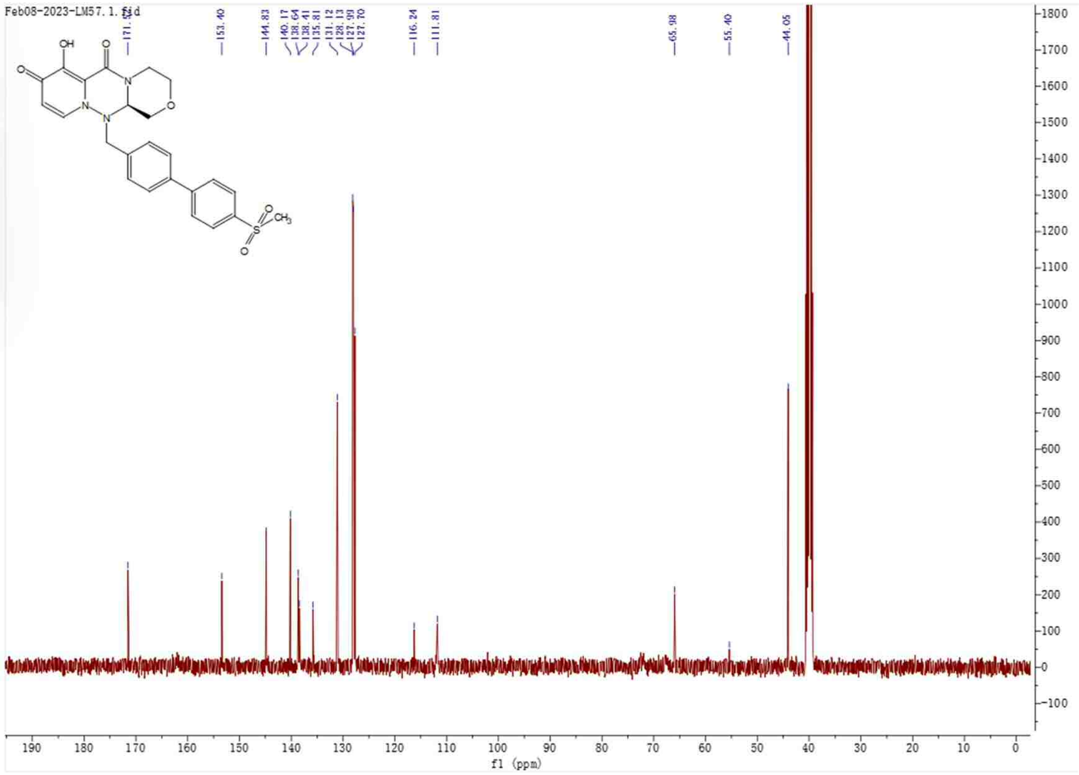

^{1}H and ^{13}C NMR spectra of compound **39**

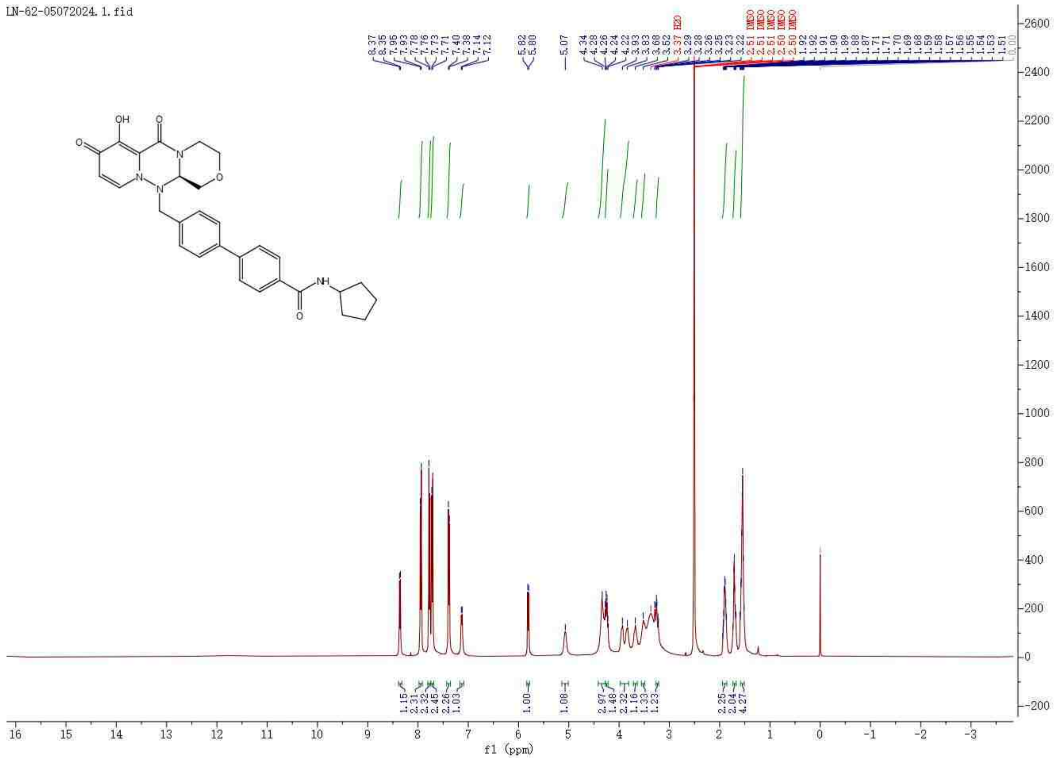

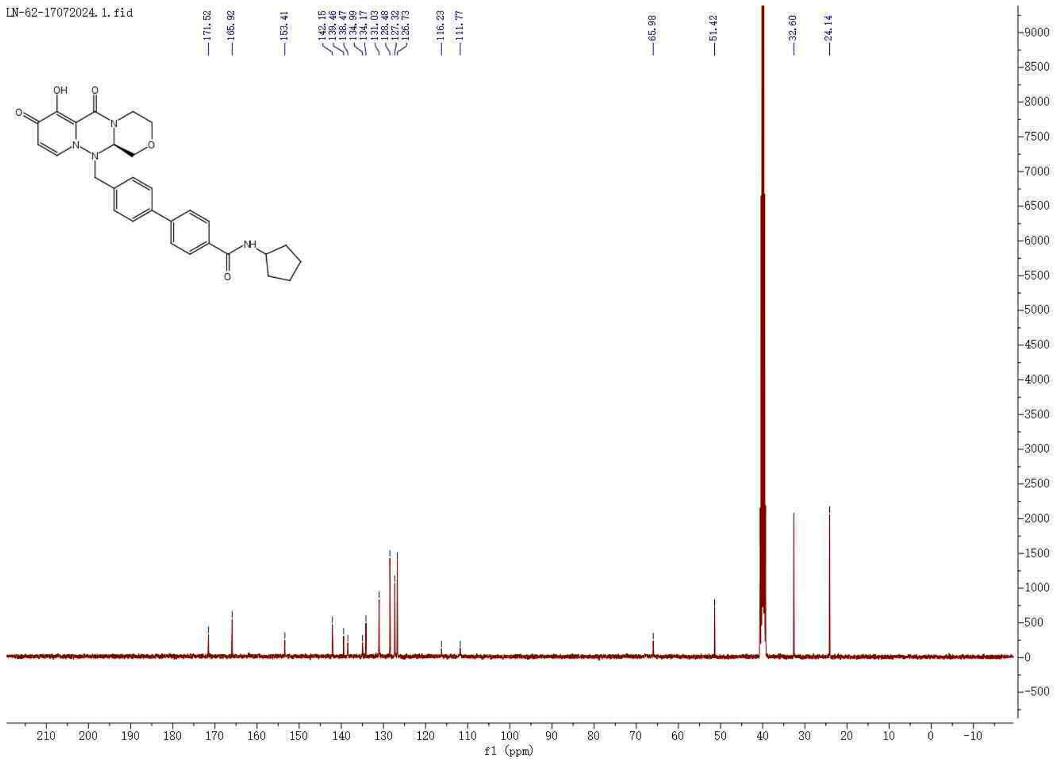

^{1}H and ^{13}C NMR spectra of compound **40**

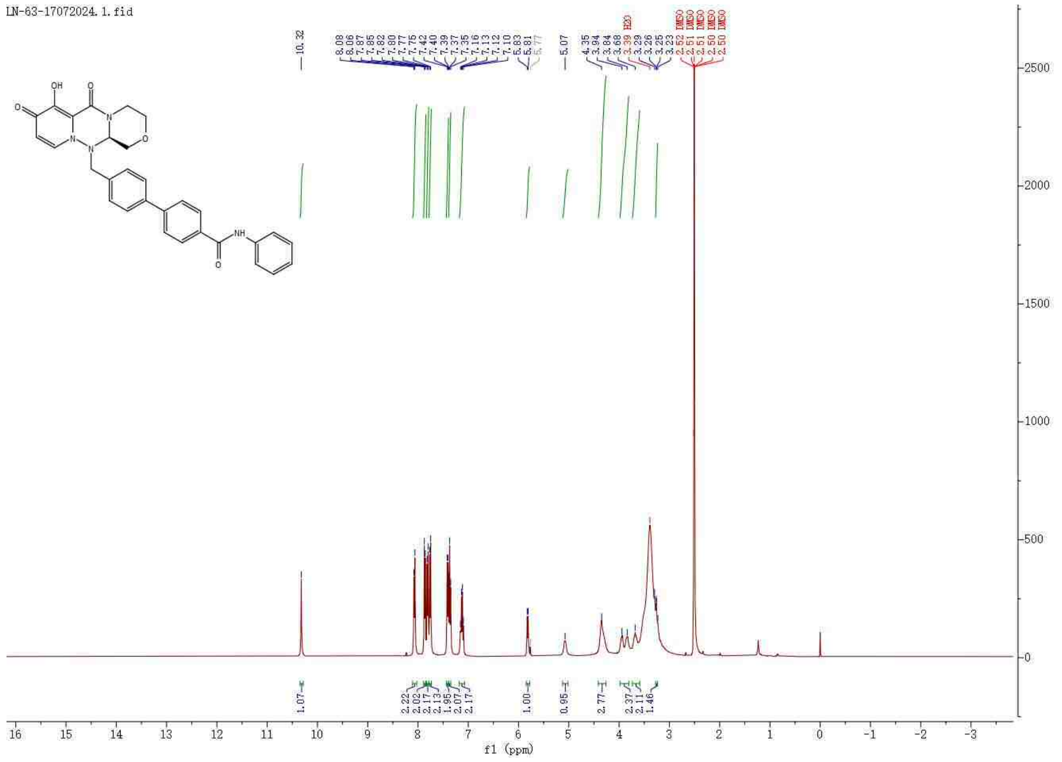

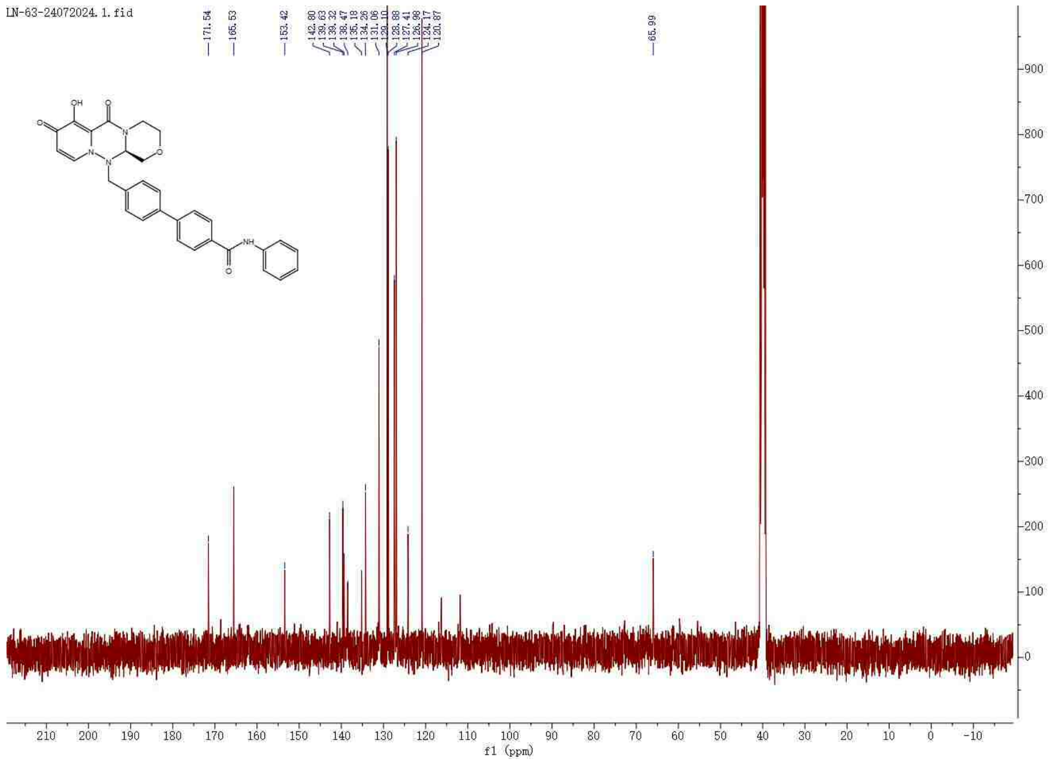

HRMS spectrum of compound **1**,

HRMS (ESI): m/z calcd for $C_{17}H_{15}N_3O_5$ $[M+H]^+$ 342.1090; found 342.1090.

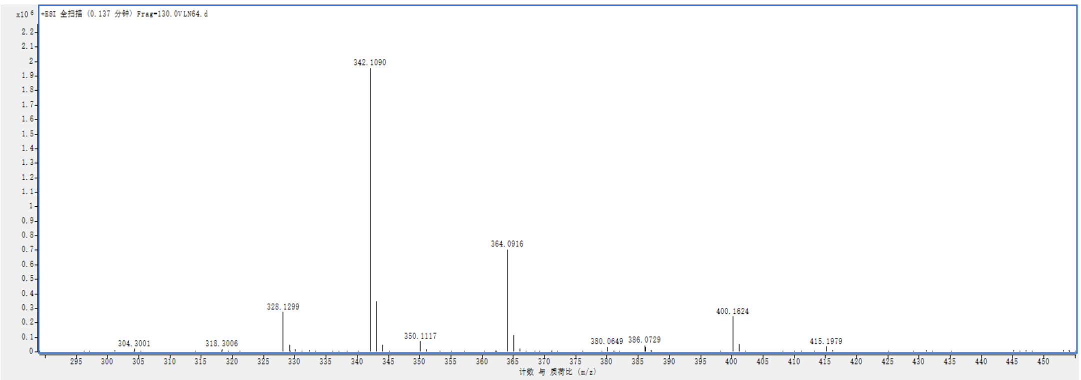

HRMS spectrum of compound **2**,

HRMS (ESI): m/z calcd for $C_{11}H_{13}N_3O_4$ $[M+H]^+$ 252.0984; found 252.0983.

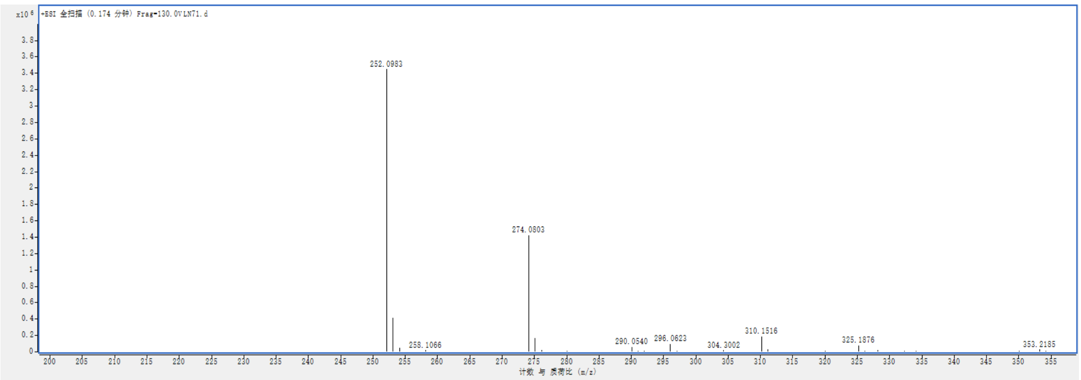

HRMS spectrum of compound **3**,

HRMS (ESI): m/z calcd for $C_{12}H_{15}N_3O_4$ $[M+H]^+$ 266.1141; found 266.1141.

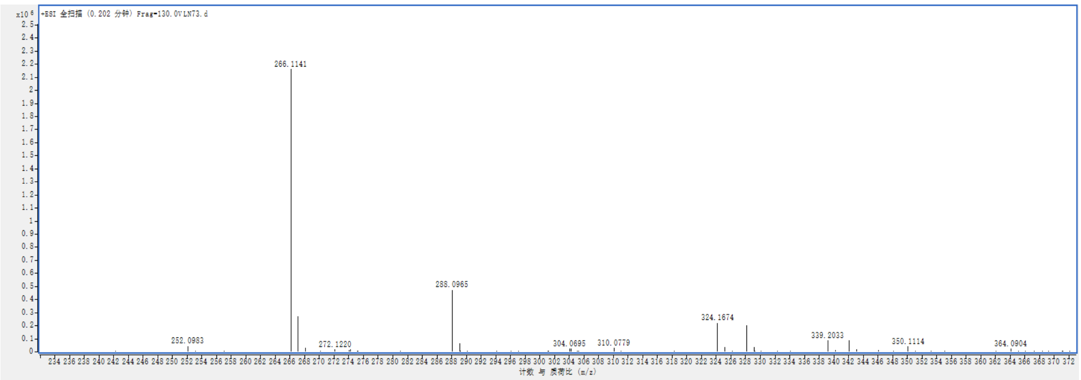

HRMS spectrum of compound **4**,
HRMS (ESI): m/z calcd for $C_{17}H_{17}N_3O_4$ $[M+H]^+$ 328.1297; found 328.1298.

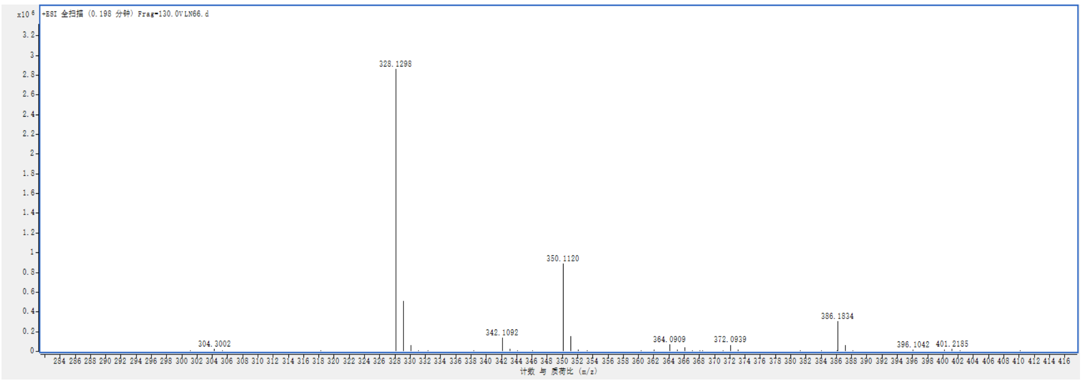

HRMS spectrum of compound **5**,
HRMS (ESI): m/z calcd for $C_{17}H_{16}IN_3O_4$ $[M+H]^+$ 454.0263; found 454.0260.

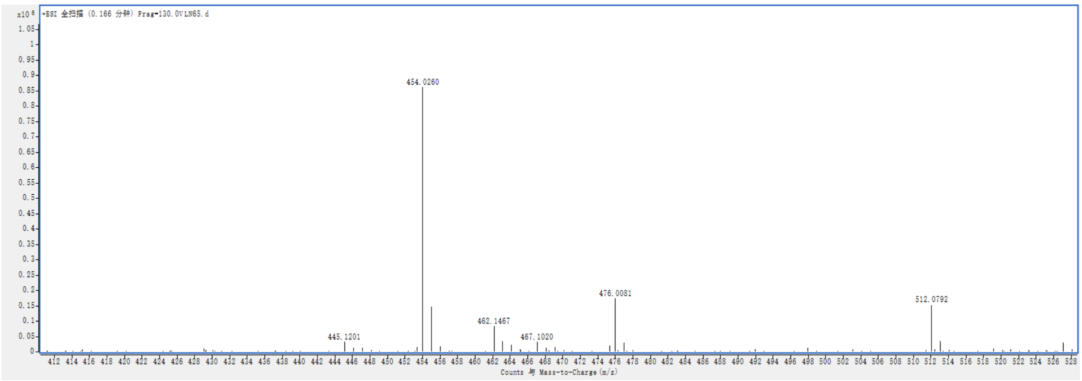

HRMS spectrum of compound **6**,
HRMS (ESI): m/z calcd for $C_{17}H_{16}FN_3O_4$ $[M+H]^+$ 346.1203; found 346.1201.

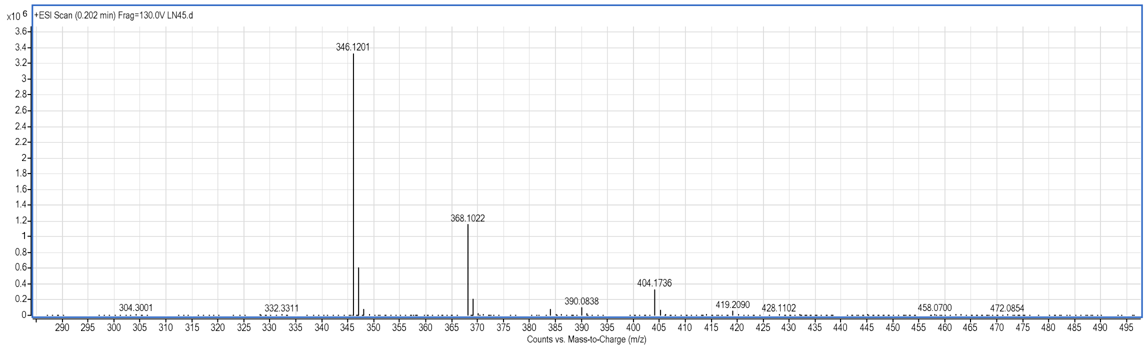

HRMS spectrum of compound **7**,
HRMS (ESI): m/z calcd for $C_{18}H_{16}F_3N_3O_4$ $[M+H]^+$ 396.1171; found 396.1166.

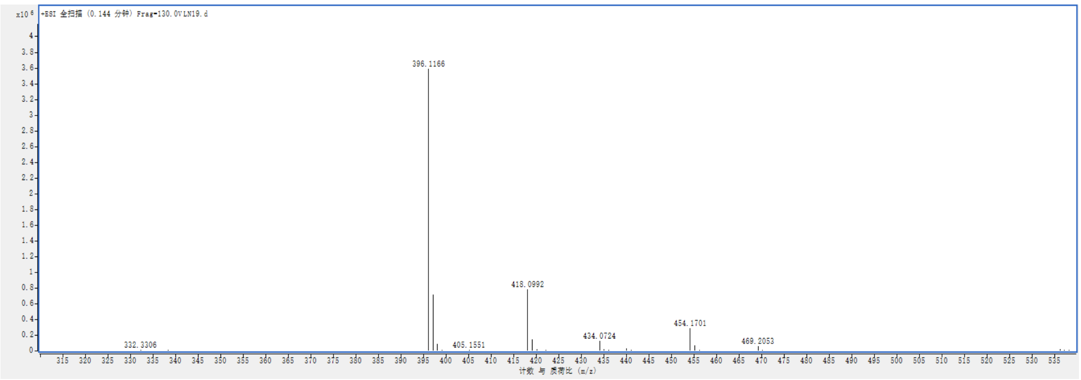

HRMS spectrum of compound **8**,
HRMS (ESI): m/z calcd for $C_{17}H_{16}FN_3O_4$ $[M+H]^+$ 346.1203; found 346.1201.

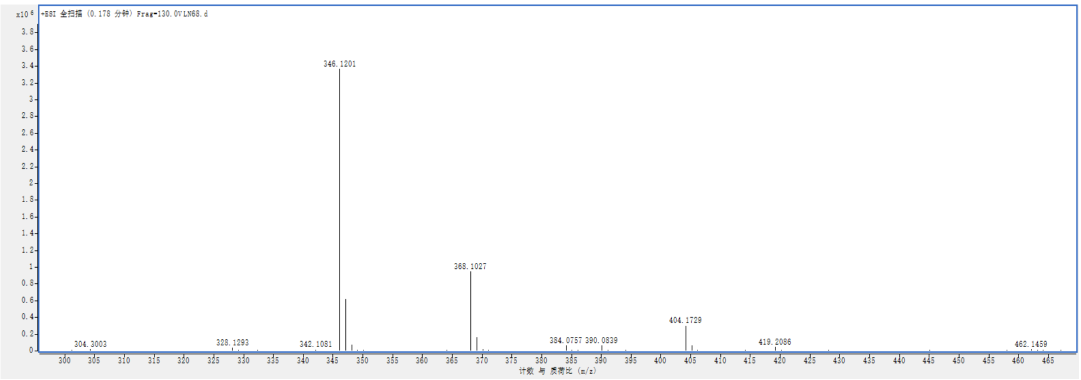

HRMS spectrum of compound **9**,
HRMS (ESI): m/z calcd for $C_{17}H_{16}FN_3O_4$ $[M+Na]^+$ 368.1023; found 368.1023.

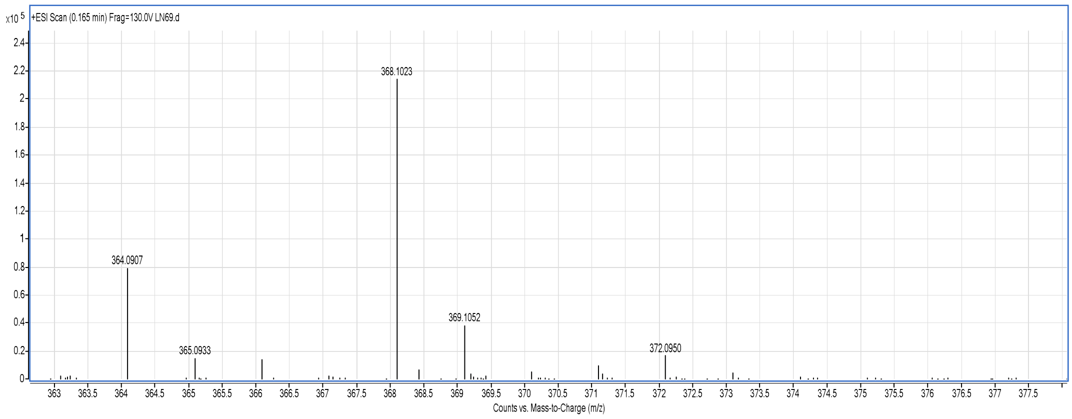

HRMS spectrum of compound **10**,
HRMS (ESI): m/z calcd for $C_{17}H_{15}ClFN_3O_4$ $[M+H]^+$ 380.0813; found 380.0810.

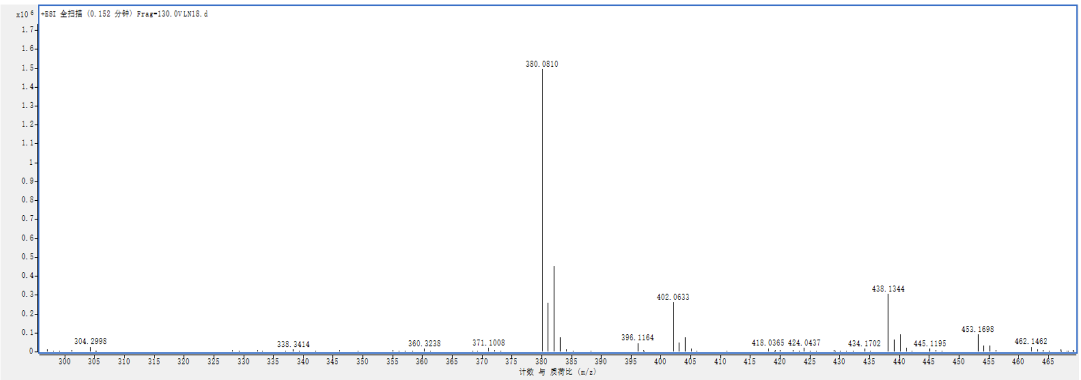

HRMS spectrum of compound **11**,
HRMS (ESI): m/z calcd for $C_{21}H_{19}N_3O_4$ $[M+H]^+$ 378.1454; found 378.1455.

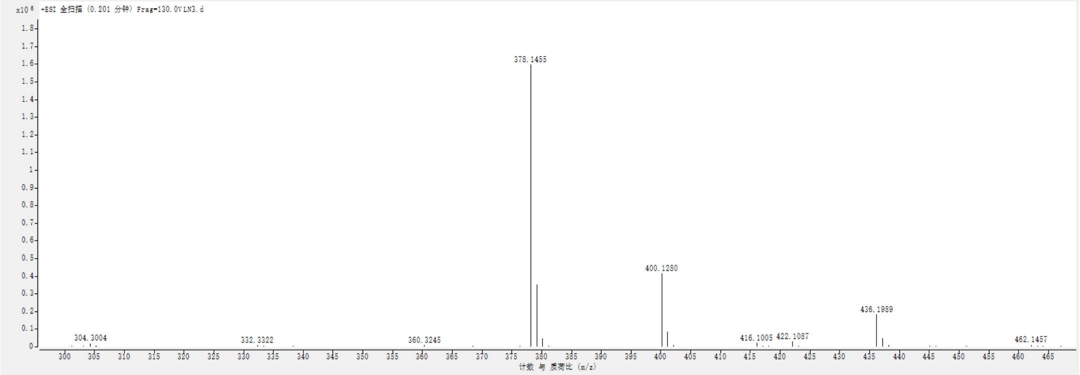

HRMS spectrum of compound **12**,
HRMS (ESI): m/z calcd for $C_{21}H_{19}N_3O_4$ $[M+H]^+$ 452.1610; found 452.1610.

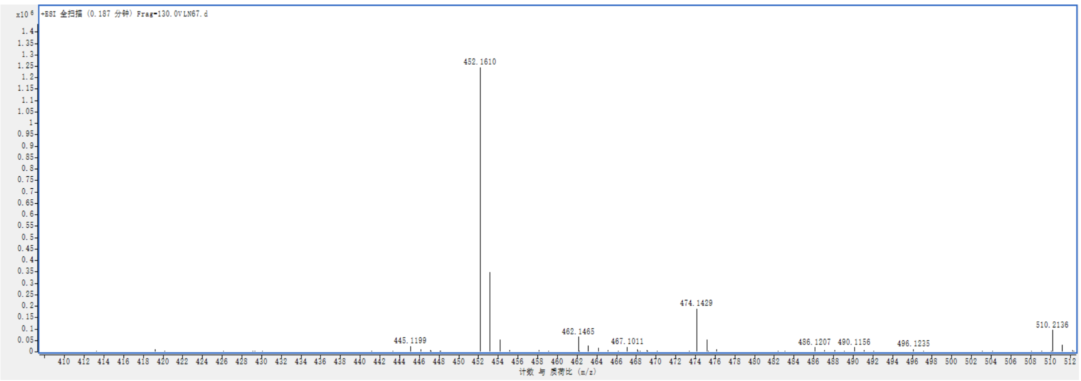

HRMS spectrum of compound **13**,
HRMS (ESI): m/z calcd for $C_{22}H_{20}N_4O_4$ $[M+H]^+$ 405.1563; found 405.1560.

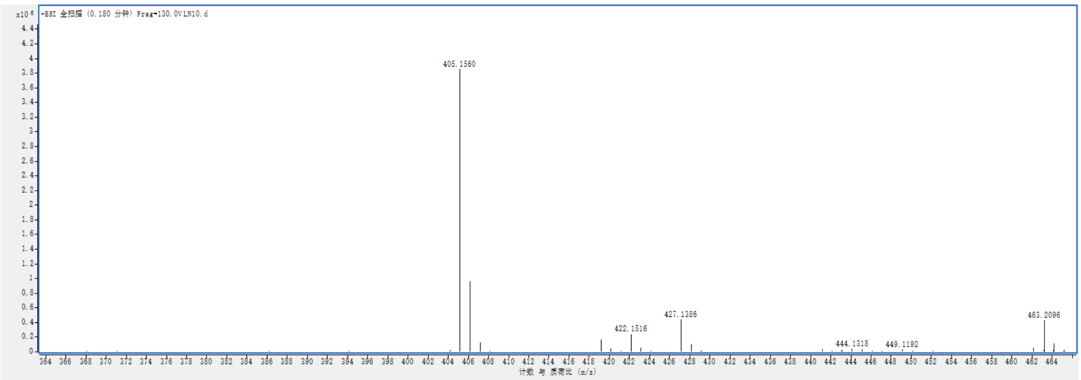

HRMS spectrum of compound **14**,
HRMS (ESI): m/z calcd for $C_{21}H_{19}N_5O_4$ $[M+H]^+$ 406.1515; found 406.1512.

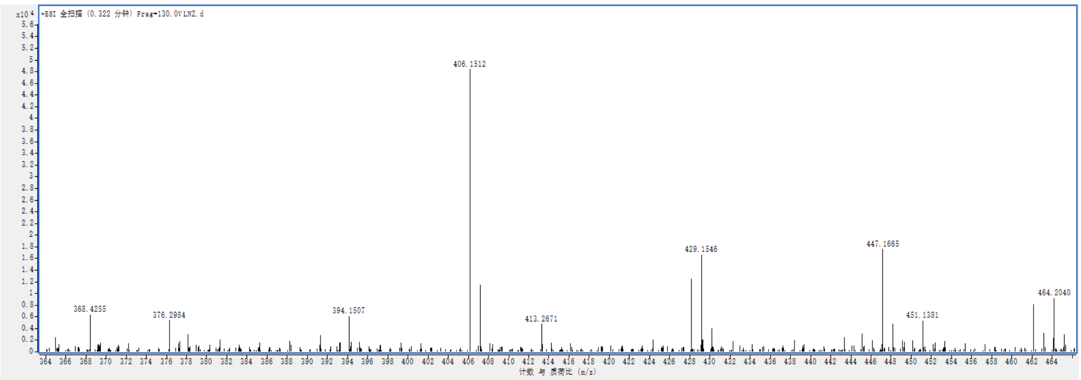

HRMS spectrum of compound **15**,
HRMS (ESI): m/z calcd for $C_{20}H_{19}N_5O_4$ $[M+H]^+$ 394.1515; found 394.1515.

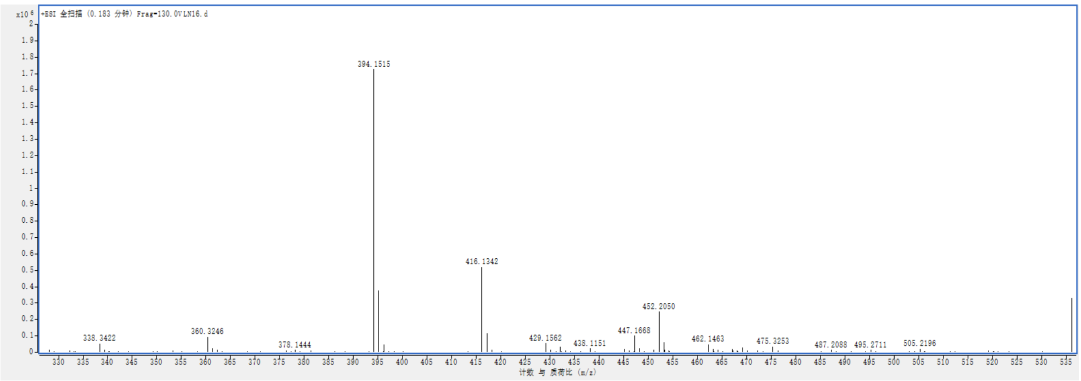

HRMS spectrum of compound **16**,
HRMS (ESI): m/z calcd for $C_{21}H_{19}N_3O_5$ $[M+H]^+$ 394.1403; found 394.1402.

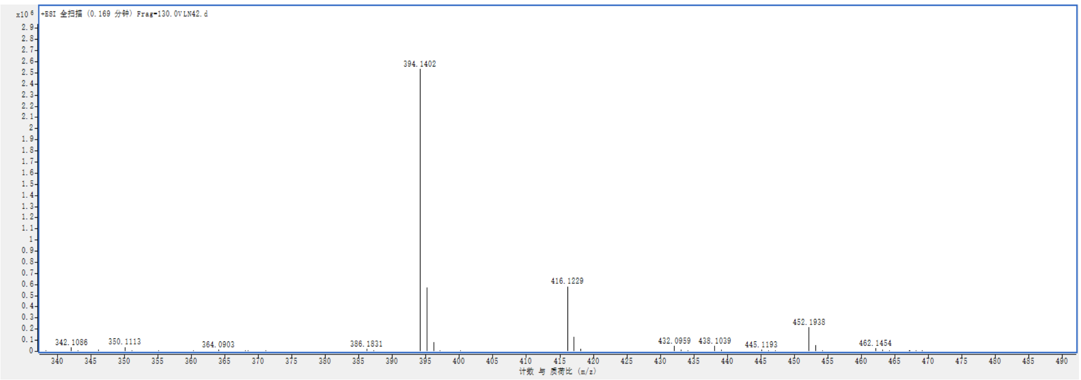

HRMS spectrum of compound **17**,
HRMS (ESI): m/z calcd for $C_{23}H_{21}N_3O_4$ $[M+H]^+$ 404.1610; found 404.1609.

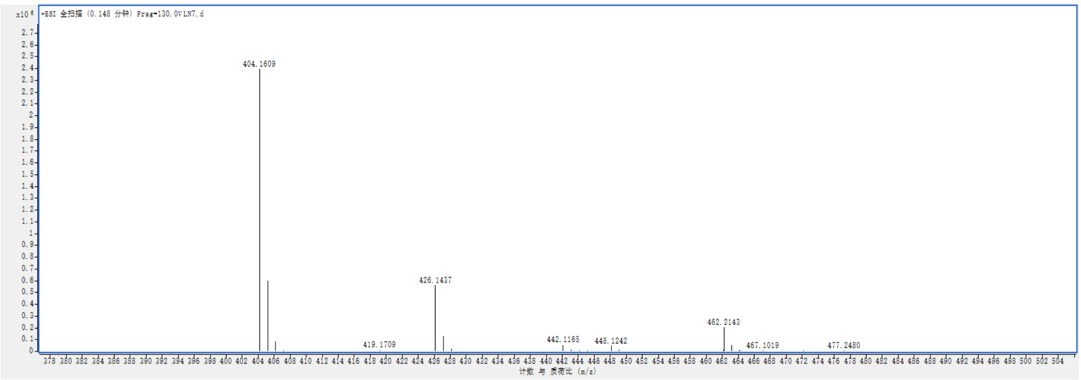

HRMS spectrum of compound **18**,
HRMS (ESI): m/z calcd for $C_{24}H_{21}N_3O_6$ $[M+H]^+$ 448.1508; found 448.1507.

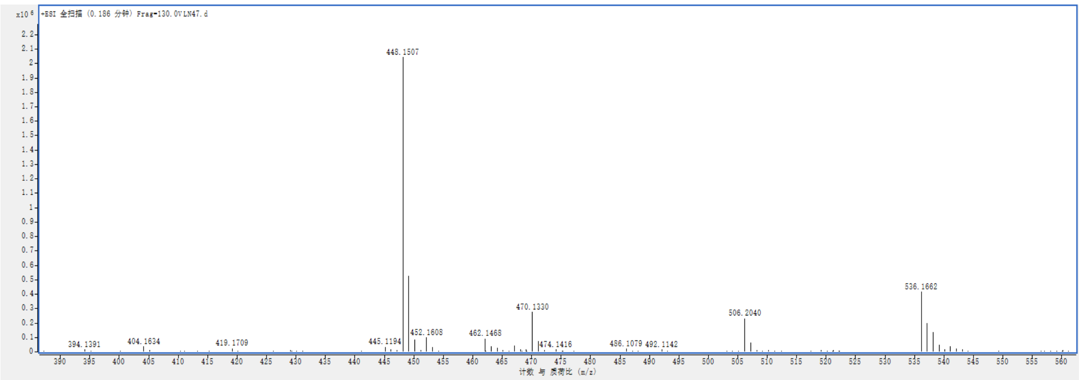

HRMS spectrum of compound **19**,

HRMS (ESI): m/z calcd for $C_{25}H_{22}N_4O_4$ $[M+H]^+$ 443.1719; found 443.1716.

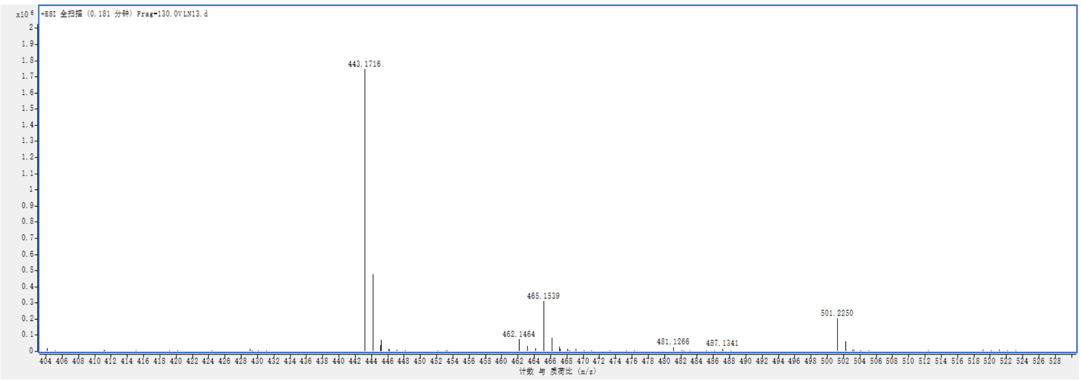

HRMS spectrum of compound **20**,

HRMS (ESI): m/z calcd for $C_{33}H_{25}N_3O_4$ $[M+H]^+$ 528.1923; found 528.1927.

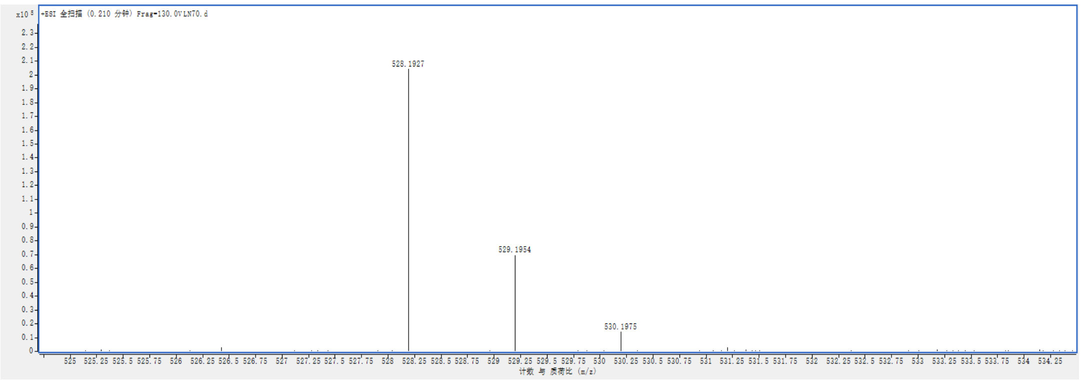

HRMS spectrum of compound **21**,

HRMS (ESI): m/z calcd for $C_{23}H_{21}N_3O_5$ $[M+H]^+$ 420.1559; found 420.1562.

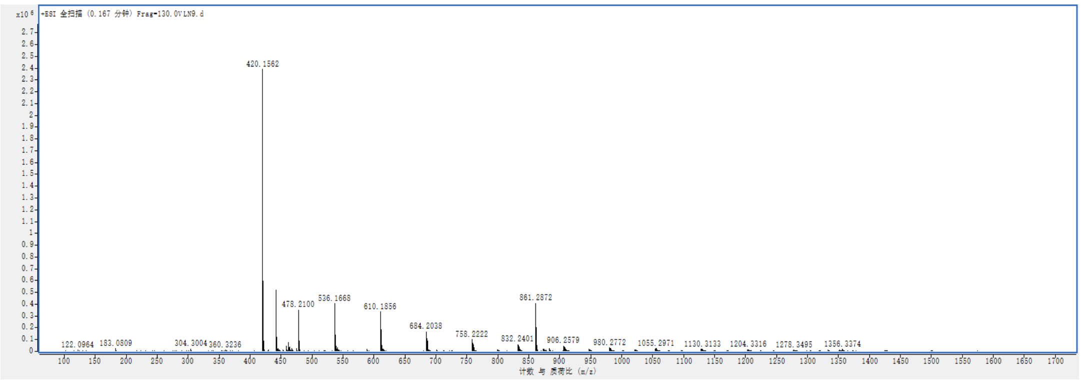

HRMS spectrum of compound **22**,
HRMS (ESI): m/z calcd for $C_{23}H_{21}N_3O_5$ $[M+H]^+$ 420.1559; found 420.1559.

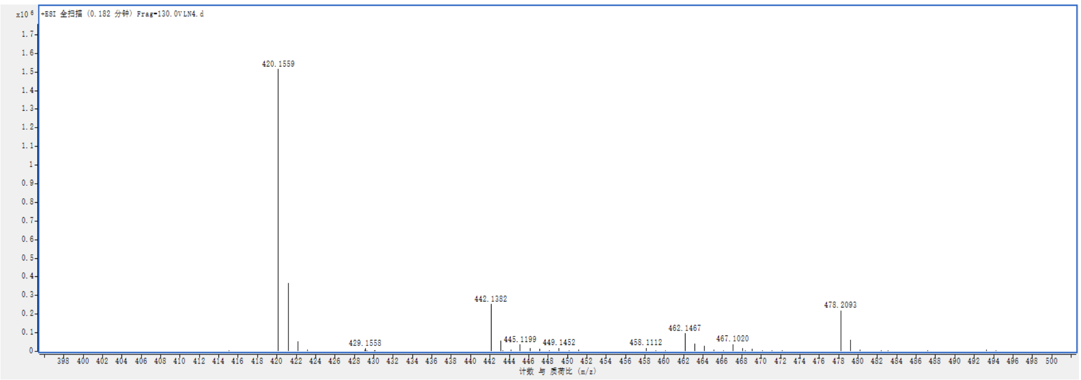

HRMS spectrum of compound **23**,
HRMS (ESI): m/z calcd for $C_{23}H_{21}N_3O_5$ $[M+H]^+$ 420.1559; found 420.1556.

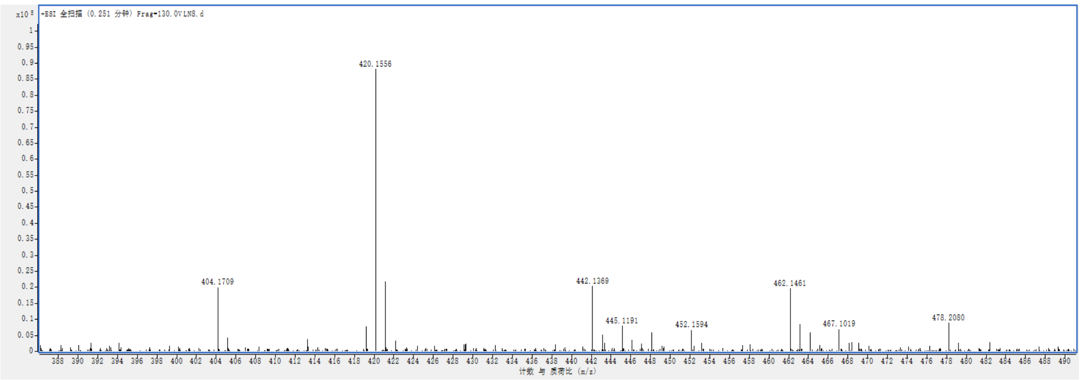

HRMS spectrum of compound **24**,
HRMS (ESI): m/z calcd for $C_{24}H_{20}N_4O_4$ $[M+H]^+$ 429.1563; found 429.1562.

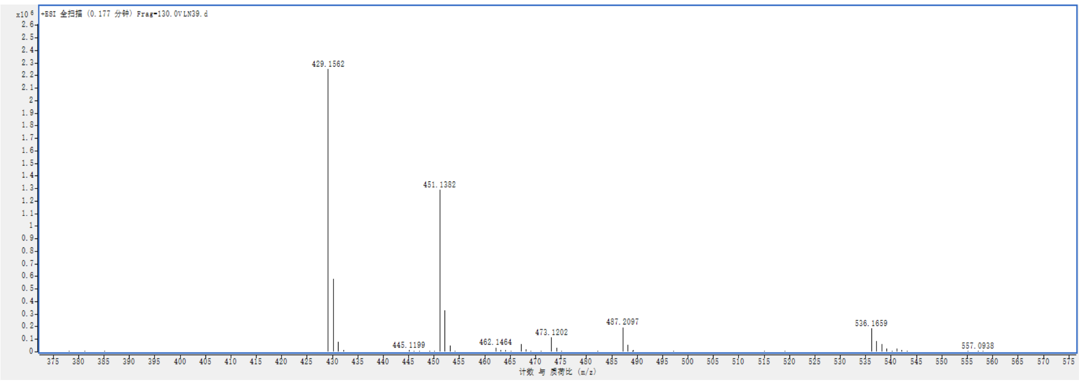

HRMS spectrum of compound **25**,
HRMS (ESI): m/z calcd for $C_{24}H_{20}N_4O_4$ $[M+H]^+$ 429.1563; found 429.1563.

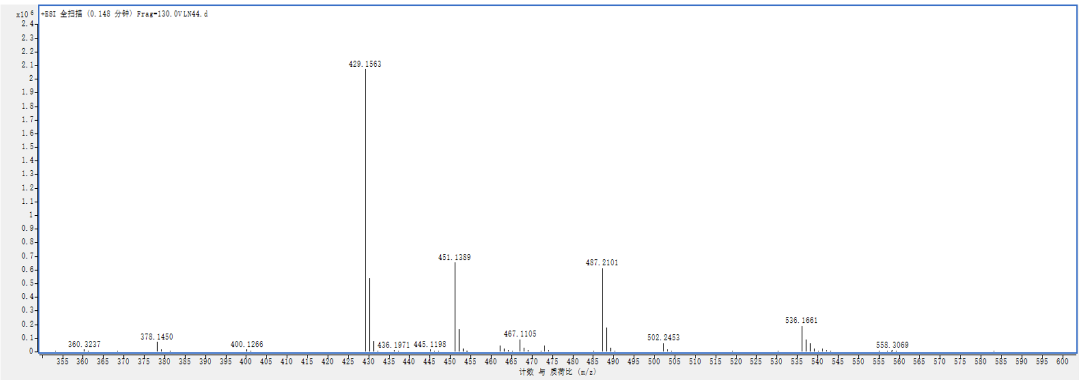

HRMS spectrum of compound **26**,
HRMS (ESI): m/z calcd for $C_{24}H_{20}N_4O_4$ $[M+H]^+$ 429.1563; found 429.1562.

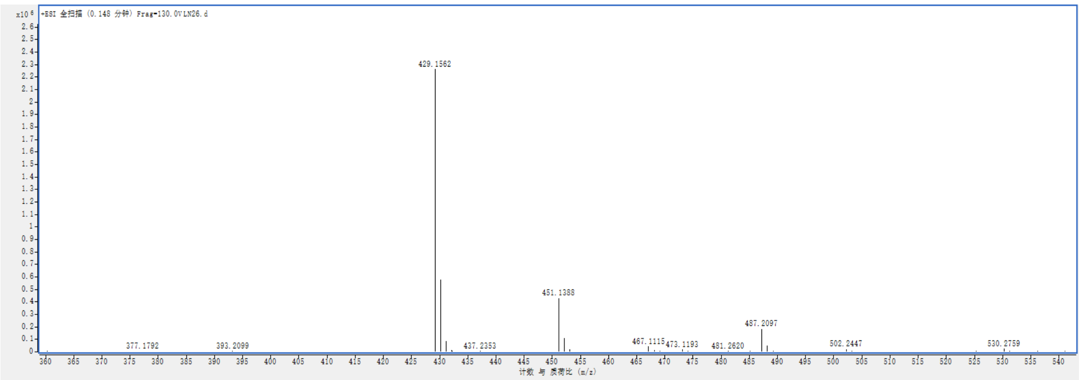

HRMS spectrum of compound **27**,
HRMS (ESI): m/z calcd for $C_{23}H_{22}N_4O_4$ $[M+H]^+$ 419.1719; found 419.1716.

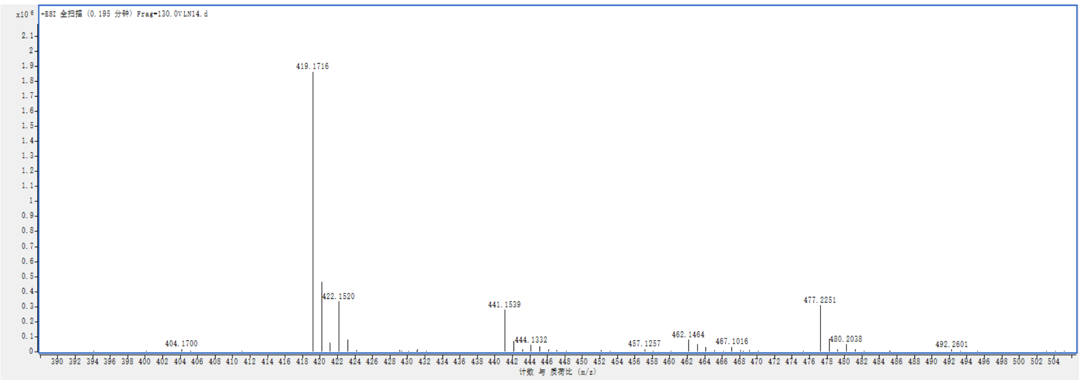

HRMS spectrum of compound **28**,
HRMS (ESI): m/z calcd for $C_{23}H_{22}N_4O_4$ $[M+H]^+$ 419.1719; found 419.1719.

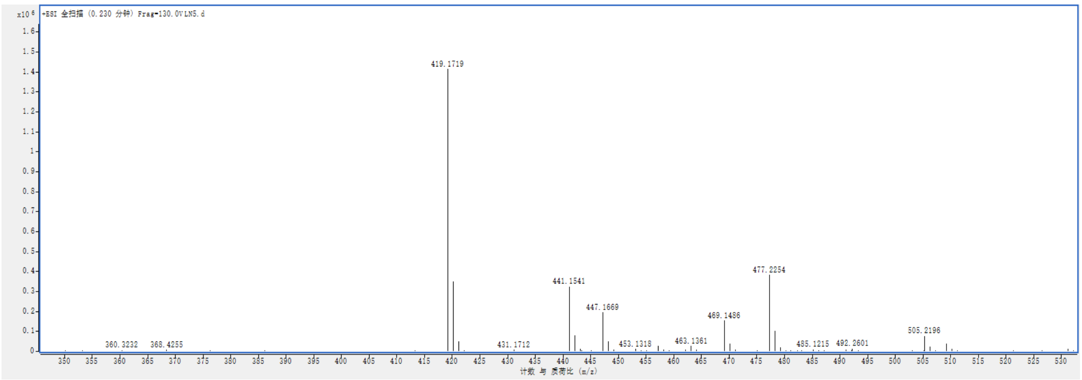

HRMS spectrum of compound **29**,
HRMS (ESI): m/z calcd for $C_{23}H_{22}N_4O_4$ $[M+H]^+$ 419.1719; found 419.1720.

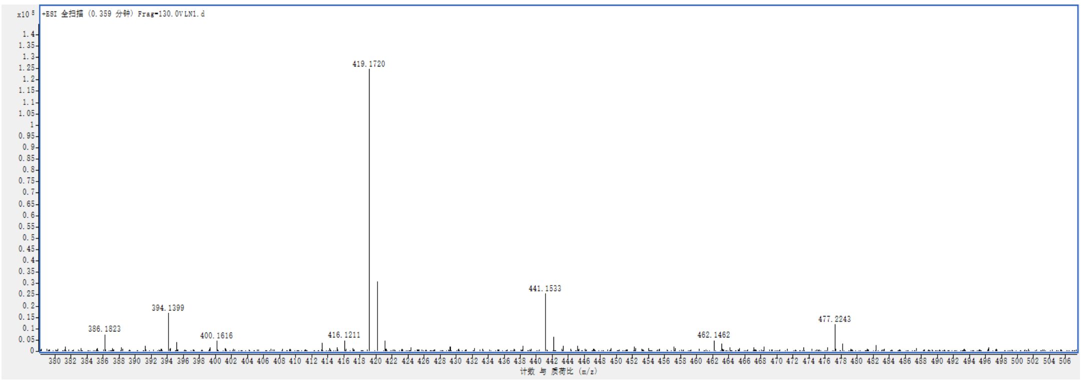

HRMS spectrum of compound **30**,
HRMS (ESI): m/z calcd for $C_{23}H_{20}FN_3O_4$ $[M+H]^+$ 422.1516; found 422.1513.

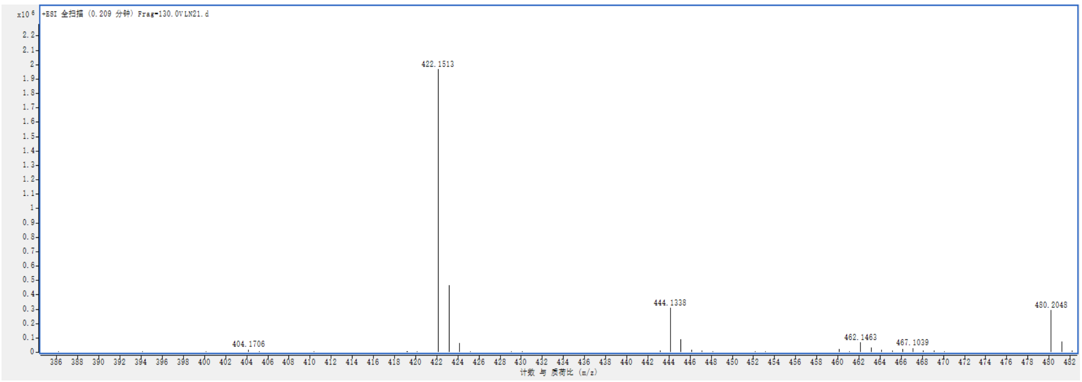

HRMS spectrum of compound **31**,
HRMS (ESI): m/z calcd for $C_{23}H_{20}ClN_3O_4$ $[M+H]^+$ 438.1220; found 438.1220.

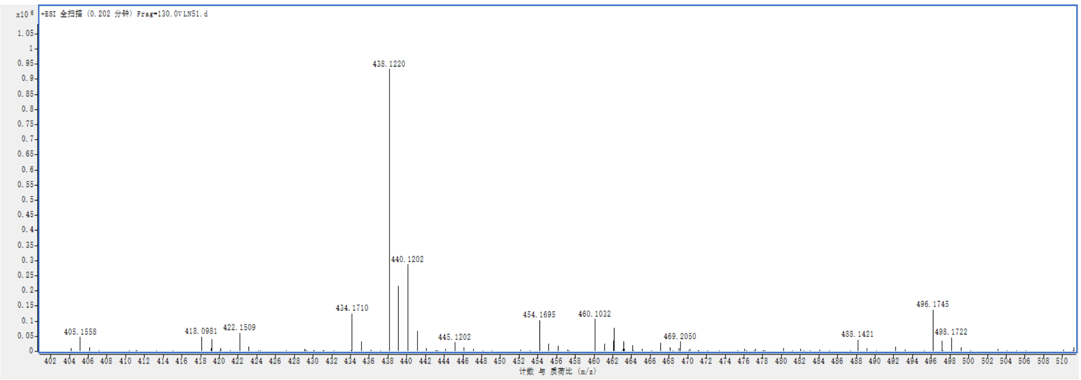

HRMS spectrum of compound **32**,
HRMS (ESI): m/z calcd for $C_{23}H_{20}N_4O_6$ $[M+H]^+$ 449.1461; found 449.1460.

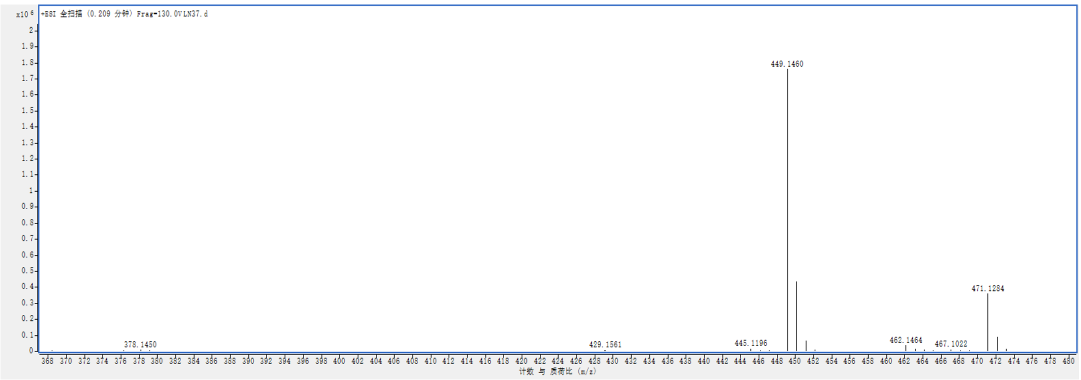

HRMS spectrum of compound **33**,
HRMS (ESI): m/z calcd for $C_{24}H_{23}N_3O_5$ $[M+H]^+$ 434.1716; found 434.1711.

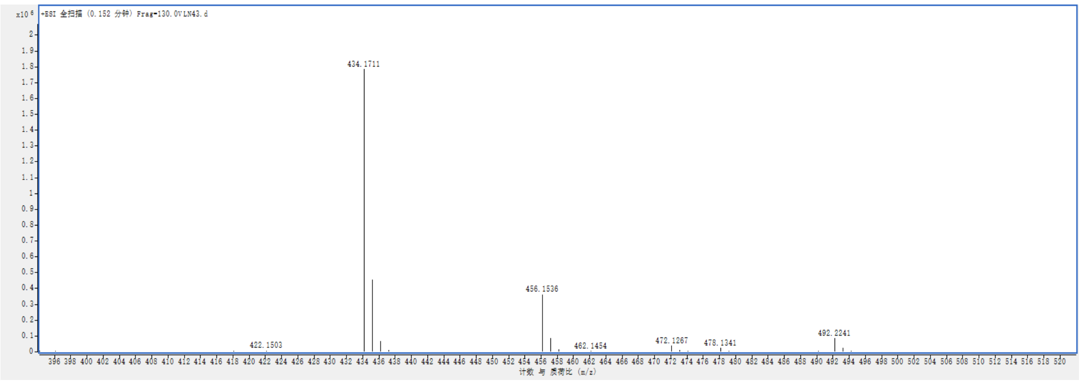

HRMS spectrum of compound **34**,
HRMS (ESI): m/z calcd for $C_{24}H_{20}F_3N_3O_5$ $[M+H]^+$ 488.1433; found 488.1431.

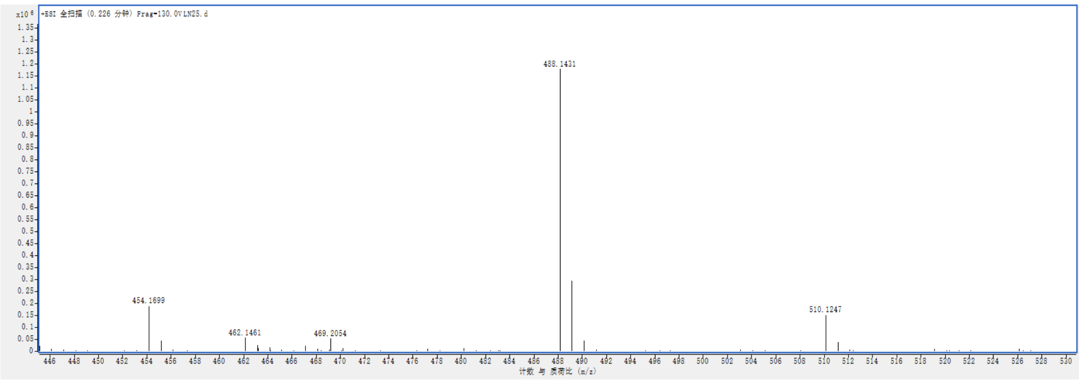

HRMS spectrum of compound **35**,
HRMS (ESI): m/z calcd for $C_{24}H_{21}N_3O_6$ $[M+H]^+$ 448.1508; found 448.1508.

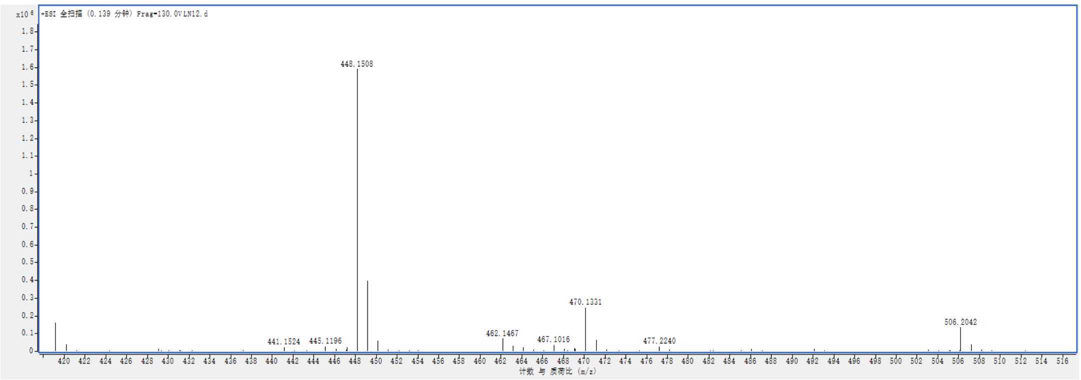

HRMS spectrum of compound **36**,
HRMS (ESI): m/z calcd for $C_{24}H_{22}N_4O_5$ $[M+H]^+$ 447.1668; found 447.1669.

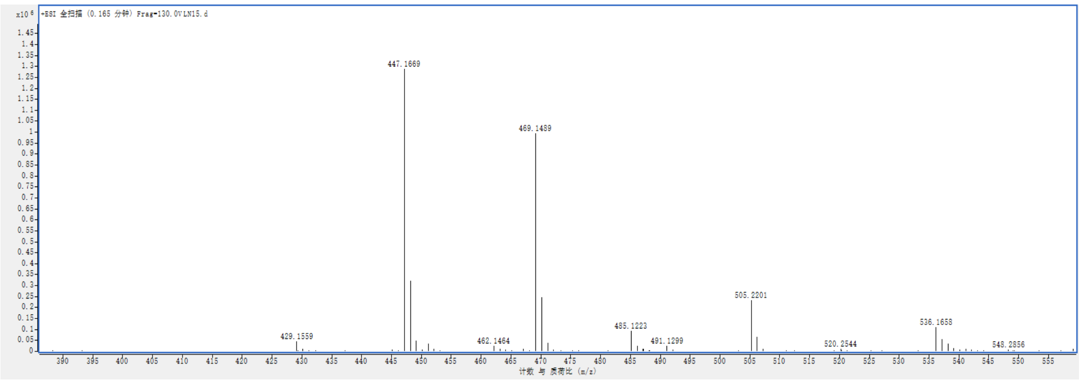

HRMS spectrum of compound **37**,
HRMS (ESI): m/z calcd for $C_{25}H_{24}N_4O_5$ $[M+H]^+$ 461.1825; found 461.1828.

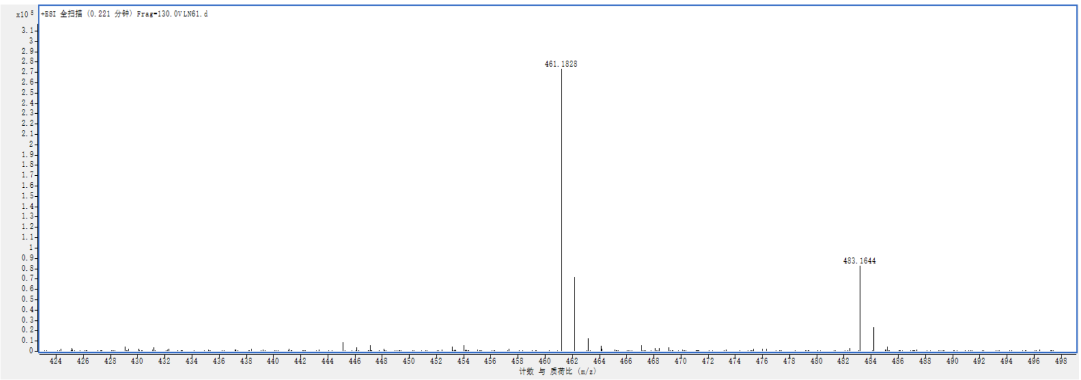

HRMS spectrum of compound **38**,
HRMS (ESI): m/z calcd for $C_{24}H_{23}N_3O_6S$ $[M+H]^+$ 482.1386; found 482.1386.

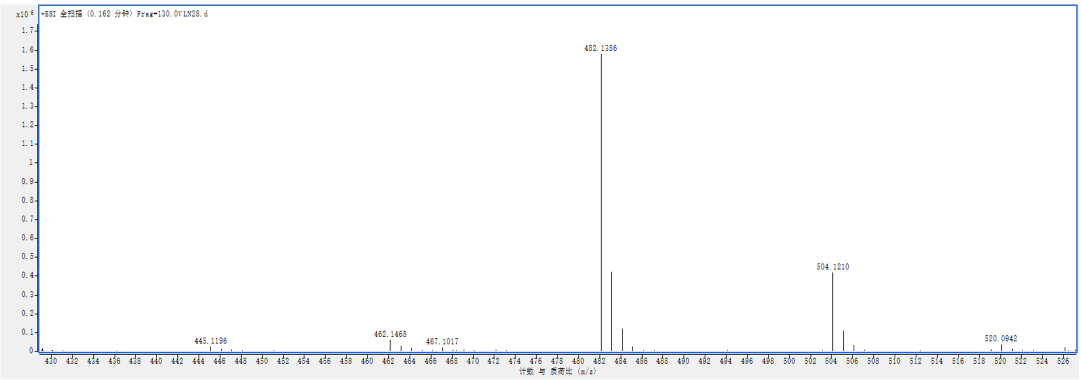

HRMS spectrum of compound **39**,
HRMS (ESI): m/z calcd for $C_{29}H_{30}N_4O_5$ $[M+H]^+$ 515.2294; found 515.2297.

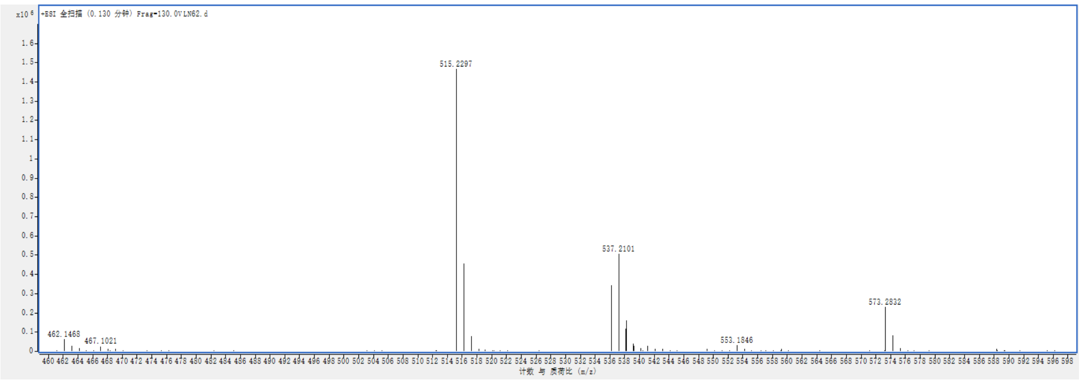

HRMS spectrum of compound **40**,
HRMS (ESI): m/z calcd for $C_{30}H_{26}N_4O_5$ $[M+H]^+$ 523.1981; found 523.1982.

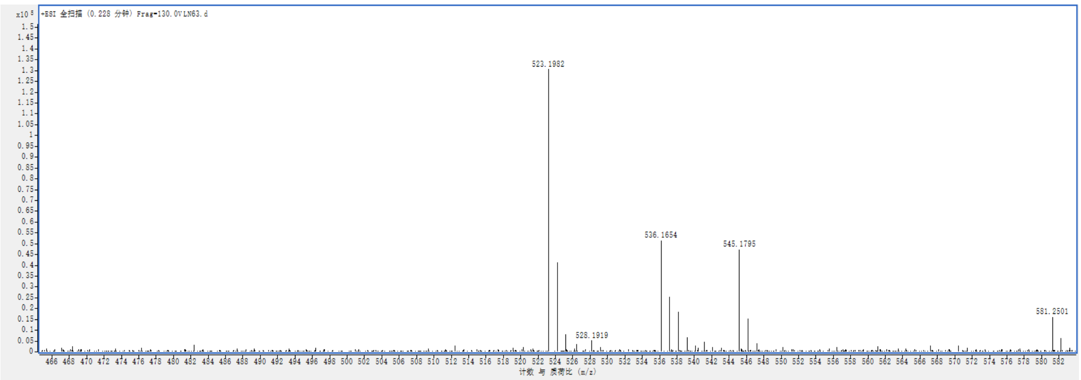

HPLC elution profile and quality control of compound 17 (LN-7)

Instrument	Waters e2695	Column:	Phenomenex column, C-18, 5.0 µm, 4.6*150 mm
Mobile Phase:	Phase A: acetonitrile<br>Phase B: water<br>Gradient elution, v/v<br>0-12min, A: 0%→95%<br>B: 95%→5%<br>12-15min, A/B = 95/5	Flow rate:	1 mL/min
Ret. Time (min):	8.708	Purity (%)	96.19%

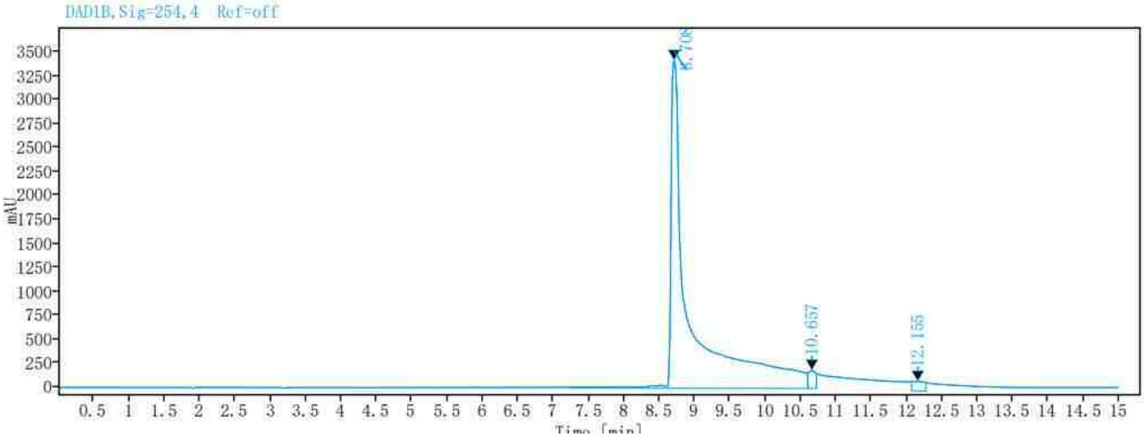

Model building of DNA linear duplexes, DNA hairpins, and their complexes with HSV pUL15C. All-atom double-stranded DNA (dsDNA) models of defined sequences were built in their equilibrium ground state conformations with the aid of the MC-DNA web server [1] a component of the Multiscale Complex Genomics project [2]. The DNA hairpin reported by Masaoka *et al.* [3] was constructed by making use of the RNAstructure [4] and RNAcomposer [5] web servers, which provided comparable secondary structure predictions. The respective 3D models were created by RNAcomposer when given as input the DNA sequence with thymines replaced by uracils. Thereafter the riboses were manually converted to deoxyriboses and the uracils back to thymines. The resulting DNA models were refined by carrying out energy minimization and MD simulations in explicit solvent, as explained below. The HSV pUL15C:dsDNA complexes were built using information from the structural alignment of chain B of PDB entry 4IOX with wild-type and D132N and E188A variants of *Halalkalibacterium halodurans* RNase H co-crystallized with different oligodeoxynucleotides (PDB entries 3EY1, 3EY2, 3EY3, 1ZBI, and 1ZBL) [6,7], as provided by the Dali web server [8].

Energy refinement and molecular dynamics simulations. Energy minimization and unrestrained MD simulations were run in explicit physiological saline solution under periodic boundary conditions, essentially as described [9] using the *gaff2* [10,11] and *ff14SB* [12] AMBER force fields for ligands and macromolecules, respectively, together with PARMBSC1 modifications for DNA [13]. Briefly, an integration step of 2.0 fs was used and the SHAKE algorithm was applied to all bonds involving hydrogens. The cutoff distance for the non-bonded interactions was 9 Å and electrostatic interactions were represented using the smooth particle mesh Ewald method with a grid spacing of 1 Å. The simulation protocol made use of the *pmemd.cuda_SPFP* engine implemented in AMBER 18 [14] running on Nvidia GTX 1080 RTX2080Ti GPUs. Following energy minimization to remove any steric clashes, solvent molecules and counterions were allowed to redistribute around the positionally restrained solute (5 kcal $mol^{-1}Å^{-2}$) during a heating phase from 100 to 300 K (0.1 ns) followed by equilibration at 300 K (2.5 ns) in the presence of weak restraints (0.5 kcal $mol^{-1}Å^{-2}$) on the proteins' Cα atoms. Each system was further simulated in the absence of any restraints for at least 250 ns during which system coordinates were collected every 5 ns for further analysis.

Analysis of the MD trajectories. Distances and angles were monitored by making use of the *cpptraj* module in AmberTools [15] whereas estimations of the solvent-corrected protein-protein and protein-DNA binding energies, as well as their per-residue decompositions into van der Waals, coulombic, apolar, and desolvation contributions were provided by the MM-ISMSA software [16].

REFERENCES

[1] Walther, J.; Orozco, M. "MC_DNA: A web server for the detailed study of the structure and dynamics of DNA and chromatin fibers", http://mmb.irbbarcelona.org/MCDNA/ (accessed October 4th, 2024).

[2] Multiscale Complex Genomics project, https://www.multiscalegenomics.eu/MuGVRE/ (accessed April 2nd, 2025).

[3] Masaoka, T.; Zhao, H.; Hirsch, D. R.; D'Erasmo, M. P.; Meck, C.; Varnado, B.; Gupta, A.; Meyers, M. J.; Baines, J.; Beutler, J. A.; Murelli, R. P.; Tang, L.; Le Grice, S. F. Characterization of the C-terminal nuclease domain of herpes simplex virus pUL15 as a target of nucleotidyltransferase inhibitors. *Biochemistry* **2016**, *55* (5), 809-819.

[4] Reuter, J. S.; Mathews, D. H. RNAstructure: software for RNA secondary structure prediction and analysis. *BMC Bioinformatics* **2010**, *11*, 129.

[5] Popenda, M.; Szachniuk, M.; Antczak, M.; Purzycka, K. J.; Lukasiak, P.; Bartol, N.; Blazewicz, J.; Adamiak, R. W. Automated 3D structure composition for large RNAs. *Nucleic Acids Res.* **2012**, *40* (14), e112.

[6] Nowotny, M.; Gaidamakov, S. A.; Crouch, R. J.; Yang, W. Crystal structures of RNase H bound to an RNA/DNA hybrid: substrate specificity and metal-dependent catalysis. *Cell* **2005**, *121* (7), 1005-1016.

[7] Pallan, P. S.; Prakash, T. P.; Li, F.; Eoff, R. L.; Manoharan, M.; Egli, M. A conformational transition in the structure of a 2'-thiomethyl-modified DNA visualized at high resolution. *Chem. Commun. (Camb)* **2009**, (issue 15), 2017-2019.

[8] Holm, L. Dali server: structural unification of protein families. *Nucleic Acids Res.* **2022**, *50* (W1), W210-W215.

[9] Mills, A.; Gago, F. Atomistic insight into sequence-directed DNA bending and minicircle formation propensity in the absence and presence of phased A-tracts. *J. Comput. Aided Mol. Des*. **2020**, *34* (3), 253-265.

[10] Wang, J.; Wolf, R.; Caldwell, J.; Kollman, P. A.; Case, D. A. Development and test of a general AMBER force field for organic molecules and bio-molecules, *J. Comput. Chem.* **2004**, *25* (9), 1157-1174.

[11] He, X.; Man, V. H.; Yang, W.; Lee, T. S.; Wang, J. A fast and high-quality charge model for the next generation general AMBER force field. *J. Chem. Phys*. **2020**, *153* (11), 114502.

[12] Maier, J. A.; Martinez, C.; Kasavajhala, K.; Wickstrom, L.; Hauser, K. E.; Simmerling, C. ff14SB: Improving the accuracy of protein side chain and backbone parameters from ff99SB. *J. Chem. Theory Comput*. **2015**, *11* (8), 3696-3713.

[13] Ivani, I.; Dans, P. D.; Noy, A.; Pérez, A.; Faustino, I.; Hospital, A.; Walther, J.; Andrio, P.; Goñi, R.; Balaceanu, A.; Portella, G.; Battistini, F.; Gelpí, J. L.; González, C.; Vendruscolo, M.; Laughton, C. A.; Harris, S. A.; Case, D. A.; Orozco, M. Parmbsc1: a refined force field for DNA simulations. *Nat. Methods* **2016**, *13* (1), 55-58.

[14] Case, D. A.; Ben-Shalom, I. Y.; Brozell, S. R.; Cerutti, D. S.; Cheatham III, T. E.; Cruzeiro, V. W. D.; Darden, T. A., Duke, R. E.; Ghoreishi, D.; Gilson, M. K. *et al.* (2018), AMBER 2018, University of California, San Francisco.

[15] Roe, D. R.; Cheatham 3rd, T. E. PTRAJ and CPPTRAJ: software for processing and analysis of molecular dynamics trajectory data. *J. Chem. Theory Comput.* **2013**, *9* (7), 3084-3095.

[16] Klett, J.; Núñez-Salgado, A.; Dos Santos, H. G.; Cortés-Cabrera, Á.; Perona, A.; Gil-Redondo, R.; Abia, D.; Gago, F.; Morreale, A. MM-ISMSA: an ultrafast and accurate scoring function for protein-protein docking. *J. Chem. Theory Comput.* **2012**, *8* (9), 3395-3408.

Other Supplementary Materials for this manuscript:

Data S1. Coordinates of the molecular model of pUL15C bound to LN-7 (Fig. 2A)
Data S2. Coordinates of the molecular model of pUL15C bound to double-stranded DNA (Fig. 2B)
